# Gene set analysis of post-lactational mammary gland involution gene signatures in inflammatory and triple-negative breast cancer

**DOI:** 10.1371/journal.pone.0192689

**Published:** 2018-04-04

**Authors:** Arvind Bambhroliya, Renae D. Van Wyhe, Swaminathan Kumar, Bisrat G. Debeb, Jay P. Reddy, Steve Van Laere, Randa El-Zein, Arvind Rao, Wendy A. Woodward

**Affiliations:** 1 The University of Texas MD Anderson Cancer Center UTHealth Graduate School of Biomedical Sciences, Houston, TX, United States of America; 2 Morgan Welch Inflammatory Breast Cancer Research Program and Clinic, the University of Texas MD Anderson Cancer Center, Houston, TX, United States of America; 3 Baylor College of Medicine, Houston, TX, United States of America; 4 Department of Breast Medical Oncology, the University of Texas MD Anderson Cancer Center, Houston, TX, United States of America; 5 Department of Radiation Oncology, the University of Texas MD Anderson Cancer Center, Houston, TX, United States of America; 6 Department of Oncology, the University of Antwerp, Antwerpen, Belgium; 7 Department of Radiology, Houston Methodist Hospital, Houston, TX, United States of America; 8 Department of Bioinformatics and Computational Biology, the University of Texas MD Anderson Cancer Center, Houston, TX, United States of America; University of Tennessee Health Science Center, UNITED STATES

## Abstract

**Background:**

Epidemiological studies have found that triple-negative breast cancer (TNBC) and TN inflammatory breast cancer (IBC) are associated with lower frequency and duration of breast-feeding compared to non-TNBC and non-TN IBC, respectively. Limited breast-feeding could reflect abrupt or premature involution and contribute to a “primed” stroma that is permissive to the migration of cancer cells typical of IBC. We hypothesized that gene expression related to abrupt mammary gland involution after forced weaning may be enriched in the tissues of IBC patients and, if so, provide a potential correlation between limited breast-feeding and the development of aggressive breast cancer.

**Methods:**

We utilized the Short Time-series Expression Miner (STEM) program to cluster significant signatures from two independent studies that analyzed gene expression at multiple time-points of mouse mammary gland involution. Using 10 significant signatures, we performed gene ontology analysis and gene set enrichment analysis (GSEA) on training and validation sets from human breast cancer gene expression data to identify specific genes that are enriched in IBC compared to non-IBC and in TN compared to non-TN in IBC and non-IBC groups.

**Results:**

Examining the combined data, we identified 10 involution gene clusters (Inv1-10) that share time-dependent regulation after forced weaning. Inv5 was the only cluster significantly enriched in IBC in the training and validation set (nominal p-values <0.05) and only by unadjusted p-values (FDR q-values 0.26 and 0.46 respectively). Eight genes in Inv5 are upregulated in both the training and validation sets in IBC. Combining the training and validation sets, both Inv5 and Inv6 have nominal p-values <0.05 and q-values 0.39 and 0.20, respectively. The time course for both clusters includes genes that change within 12 hours after forced weaning.

**Conclusions:**

Results from this *in silico* study suggest correlation between molecular events during abrupt involution and aggressive breast cancer. Specifically, candidate genes from Inv5 merit functional investigation regarding the role of limited breast-feeding in IBC development.

## Introduction

The mammary gland undergoes various stages of development during the embryonic, pubertal, reproductive, and post-reproductive stages of life. Involution is a term that has been described as the reverse of development [1]. Post-lactational involution is a complex multistage process characterized by regression of the mammary gland epithelium to its non-lactating state through apoptosis and tissue remodeling [2]. Clarkson et al. [3] and Stein et al. [4] conducted gene expression profiling studies of these changes in the mouse mammary gland with the induction of forced weaning at the peak of lactation. Results of these two studies highlight distinct molecular characteristics between the virgin, pregnant, lactating, and involuting states of the mammary gland. It has been shown that the mammary gland microenvironment undergoing tissue remodeling during post-lactational involution mimics that of pathological conditions like wound healing and tumorigenesis [5]. Inflammation and would-healing responses have been found to be associated with tumor growth and progression [6]. Findings from several animal and *in vitro* studies indicate that involution may create a microenvironment that promotes breast cancer growth and progression [7–12].

Breast cancer is the most common cancer and the second leading cause of cancer mortality among women in the United States [13]. In the absence of estrogen receptor (ER) expression, progesterone receptor (PR) expression, and HER2-neu amplification, breast cancer is termed triple-negative breast cancer (TNBC). TNBC accounts for approximately 15% of all breast cancer incidents and has the lowest survival rates among all subtypes of breast cancer [14]. Inflammatory breast cancer (IBC) is a distinct subtype of breast cancer characterized pathologically by the presence of tumor emboli in the dermal lymphatics and clinically by its rapid and diffuse onset with erythematous and edematous presentation of the breast [15]. IBC accounts for approximately 1–5% of all breast cancers but at all stages has significantly lower 5-year survival rates than that for non-IBC [15–17]. Little-to-no breast-feeding has been found to correlate with an increased risk of developing aggressive breast cancer subtypes [18, 19]. In Gaudet et al, TNBC was associated with a shorter duration of breast-feeding in a cohort of 890 young (≤56 years) breast cancer patients [18]. Atkinson et al found also that in a cohort of 224 women with IBC, those that did not breast-feed were more likely to develop TN IBC and luminal IBC [19]. Furthermore, Lyons et al. showed that TN ductal carcinoma in-situ (DCIS) cells exposed to the involuting mammary microenvironment formed large, invasive tumors characterized by abundant fibrillar collagen and high COX-2 expression, which both correlate with a poor prognosis [10]. Additionally, both luminal and myoepithelial lineages in the mammary gland contain long-lived stem cells and stem-like cells, and pregnancy leads to a transient 11-fold increase in their habitation there throughout lactation [11, 12, 20]. In particular, parity-induced mammary epithelial cells (PI-MECs) are an epithelial subpopulation that arise from differentiating cells during the first pregnancy and persist after postlactational remodeling. They later serve as committed alveolar progenitors along the luminal epithelium during subsequent pregnancies and exhibit two important features of multipotent stem cells: self-renewal and contribution to diverse epithelial populations in the ducts and alveoli [20, 21]. PI-MECs have been identified as primary targets of malignant transformation [22]. Thus, it is possible that abrupt involution leaves persistent, likely receptor-negative stem cells or PI-MECs within the mammary gland microenvironment, increasing the chance of an initiating TNBC and TN IBC event.

Given the correlation between minimal breast-feeding and aggressive breast cancers, we hypothesized that gene expression signatures of the abrupt post-lactational involution stage of mammary gland development persist, are complicit in the development and progression of these cancers, and are therefore enriched in TNBC and IBC bulk tumor samples. To test our hypothesis, we identified gene expression signatures for the abrupt post-lactational involution stages of mammary gland development using gene expression data from post-natal mouse mammary gland development and evaluated whether or not these gene expression signatures were enriched in IBC versus non-IBC as well as TNBC versus non-TNBC. We found significant enrichment of one post-lactational involution gene signature in IBC compared to non-IBC. This enriched signature represents genes showing initial up-regulation and later down-regulation during the involution process and significant overlap with genes upregulated in vascular smooth muscle cells (VSMC) by c-Jun N-terminal protein kinase (*JNK1*). Specifically, we identified 3 genes–Involucrin (*IVL*), Cluster of Differentiation 79B (*CD79B*), and leptin (*LEP*)–that were significantly enriched in IBC compared to non-IBC. Thus, it is possible that these genes play a role in IBC development and progression.

## Materials and methods

### Development of involution-specific gene signatures

#### Gene expression dataset on mouse mammary gland involution

In the study by Clarkson et al. [3], genome-wide expression profiles were measured with Affymetrix GeneChip MGU74ver2a arrays at the 12 stages of adult mouse mammary gland development (virgin, 8 week; pregnancy days 5, 10 and 15; lactation days 0, 5 and 10; and involution hours 12, 24, 48, 72 and 96 after forced weaning at 10 days of lactation). In the study by Stein et al. [4], gene expression profiles were measured at the 17 stages of adult mouse mammary gland development (virgin, 10 and 12 weeks; pregnancy days 1, 2, 3, 8.5, 12.5, 14.5 and 17.5; lactation days 1, 3 and 7; and involution days 1, 2, 3, 4 and 20 after forced weaning at 7 days of lactation). The gene expression data for both studies can be downloaded from the webpage of the Mammary Apoptosis and Development Group at the University of Cambridge and from the NCBI Gene Expression Omnibus (GSE12247).

#### Preprocessing of gene expression data

Raw gene expression profiles were preprocessed using Guanine Cytosine Robust Multi-Array (GCRMA) analysis [23] with quantile normalization, and probeset-level signals were summarized in log base 2 scale. We selected a custom Chip Definition File (CDF) MGU74Av2_Mm_ENTREZG version 18 for more accurate probe mapping to the genome [24]. There are 7952 probe sets with a CDF MGU74Av2_Mm_ENTREZG version 18 representing 7882 genes as per the annotation database available for a CDF MGU74Av2_Mm_ENTREZG version 18 at the BrainArray. After preprocessing gene expression data, further analyses were conducted using information available for 7882 probe sets representing 7882 genes with a one probe set–one gene relationship.

#### Identification of differentially expressed genes across time points

Tests of differences in expression were performed with the limma package (version 3.22.1) [25] from the Bioconductor project. The limma package uses the moderated t-statistic. A total of 1,055 genes were identified as the most significantly differentially expressed genes across time points (q-value <0.05) with greater than two-fold changes in at least one pair comparing time points.

#### Clustering analysis of time-series expression data

Ernst et al. presented an algorithm specifically designed for clustering time-series expression data [26] and developed the Short Time-series Expression Miner (STEM) program for analysis of time-series gene expression data [27]. The STEM program was obtained from the website of the Systems Biology Group of the School of Computer Science of Carnegie Mellon University. The STEM program first defines a set of representative model profiles that correspond to possible patterns of gene expression across the conditions examined in the experiment. Each gene is, then, assigned to the closest profile on the basis of correlation coefficients. The expected number of genes for each profile is also computed using random permutation, renormalization, and assignment of original values for each gene to profiles with over 500 repeated permutations. This serves as a basis for the calculation of the statistical significance of each profile. Statistically significant profiles represent the dominant expression profiles in the data set. The parameters used for STEM clustering were set at a maximum of 50 model profiles, a maximum unit change between time points of 3 and a minimum correlation for clustering similar profiles >0.7. Significant expression profiles were identified with a false discovery rate (FDR) <0.05.

#### Ontology analysis of significant clusters

An ontology-based analysis was performed on genes of significant clusters identified through the STEM program. We used gene ontology (GO) annotations for *Mus musculus* gene products available from Mouse Genome Informatics. Enrichment analysis for GO annotations was performed using a hypergeometric distribution in the STEM program, and multiple hypothesis correction was done using a randomization test. For gene-ontology enrichment with this program, p*-*values were corrected with 500 randomizations and were considered significant with an FDR of <0.05.

### Gene set analysis of involution-specific gene signatures

#### Gene expression dataset on IBC and non-IBC cases

Gene expression data for IBC and non-IBC cases were obtained from the NCBI Gene Expression Omnibus (GSE22597) and the EBI ArrayExpress (E-MTAB-1006 and E-MTAB-1547) and collected through the World IBC Consortium [28]. These databases include the largest series of IBC samples ever reported, and tumor samples were obtained from patients treated in three institutions: the Institut Paoli-Calmettes (IPC, Marseille, France: 71 IBC and 139 non-IBC cases), the MD Anderson Cancer Center (MDA, Houston, TX, USA: 25 IBC and 58 non-IBC cases), and the General Hospital Sint-Augustinus (TCRU, Antwerp, Belgium: 41 IBC and 55 non-IBC cases) [28].

We also examined benign-appearing breast tissues from both IBC and non-IBC patients, 44 in total (19 with IBC and 25 with non-IBC) for further analysis. All were treated with neoadjuvant chemotherapy and mastectomy from March 2004 –May 2012. Clinical details for all patients was recorded as part of an institutional database or prospective tumor registry. All patients gave written informed consent to banking surplus tissue for future research prior to study enrollment. This specific study was separately approved by the appropriate institutional review board of The University of Texas MD Anderson Cancer Center to examine these banked tissues and correlate findings to clinical demographics.

#### Preprocessing of gene expression data

Raw gene expression profiles were preprocessed using GCRMA analysis [23] with quantile normalization, and probeset-level signals were summarized in log base 2 scale. We selected custom Chip Definition Files (CDFs) HGU133A_Hs_ENTREZG version 18 for preprocessing GSE22597 data and HGU133Plus2_Hs_ENTREZG version 18 for preprocessing E-MTAB-1006 and E-MTAB-1547 data [24]. There are 12,135 probe sets with a CDF HGU133A_Hs_ENTREZG version 18 with 12,064 probe sets representing 12,064 genes as per the annotation database available at the BrainArray. There are 19,674 probe sets with a CDF HGU133Plus2_Hs_ENTREZG version 18 with 19,544 probe sets representing 19,544 genes as per the annotation database available at the BrainArray. After preprocessing gene expression data, all 3 data sets were merged using common informative probe sets (n = 12,129). To remove the batch effect, we used the removeBatchEffect function from the limma package from the Bioconductor [25]. This function fits a linear model to the data and removes the components due to the batch effects. The principal component analysis plots were generated prior and after removing the batch effect to of the removeBatchEffect function (data not shown). The final merged dataset consisted of 388 samples (137 IBC cases and 251 non-IBC cases) with 12,129 probe sets with 12,063 probe sets representing 12,063 genes with a one probe set–one gene relationship.

### Non-tumor breast gene expression

We employed the previously described methods for gene expression studies from normal adjacent breast tissue [29, 30]. Briefly, RNA was isolated using TRIzol (Invitrogen, Carlsbad, CA, USA) and the RNeasy kit (Qiagen, Valencia, CA, USA). A flouorescently labeled T7 RNA polymerase promotor was used to synthesize cDNA. Reverse transcription was performed and followed by RNA labeling. The labeled RNA samples were hybridized onto U133 Plus2 GeneChips (Affymetrix, Santa Clara, CA). dChip analyzer software was used to estimate expression values, as previously described [29, 30].

#### Involution-specific gene signatures

Involution-specific gene signatures were identified from the results of STEM cluster analysis conducted on post-natal mouse mammary gland development as previously described here. We identified orthologous genes for genes that were found to form significant clusters in STEM cluster analysis by using ENSEMBL gene id on the orthologous data downloaded from the ENSEMBL website. Involution-specific gene signatures have also been reported in the study by Stein et al. [9]. We downloaded those signatures and identified orthologous genes for each signature (S1 Table) and used them to evaluate their enrichment in IBC versus non-IBC and TN versus non-TN subtypes.

#### Gene set enrichment analysis (GSEA) of involution-specific signatures

We used GSEA algorithm as mentioned in [31] to evaluate enrichment of involution-specific gene signatures in IBC cases compared to non-IBC cases and TN BC cases compared to non-TN BC cases. We ranked genes in the GSEA using the student’s t-test, and all other options in the GSEA were kept as default.

#### Training and validation data

A training set is a set of data used to discover potentially predictive relationships, and a validation set is used to assess the strength and utility of said predictive relationships. We divided the merged dataset into the training set to run the GSEA and into the validation set to validate the GSEA results for reproducibility. We used the stratified random sampling method with inclusion of information on IBC status, TN status, and age at diagnosis (<50 years or > = 50 years) to divide the merged dataset into the training and validation sets (Table 1).

**Table 1:** Final merged dataset and training and validation sets.

	Total cases	IBC*			Non-IBC*		
		Total	TN	Non-TN	Total	TN	Non-TN
Merged Dataset	388	137	20	101	251	34	197
Training Set	195	68	10	50	127	18	99
Validation Set	193	69	10	51	124	16	98

*16 cases in IBC and 20 cases in non-IBC groups did not have information available on TN status.

## Results

### Development of involution-specific gene signatures

#### Differentially expressed genes across time points

**Fig 1:**
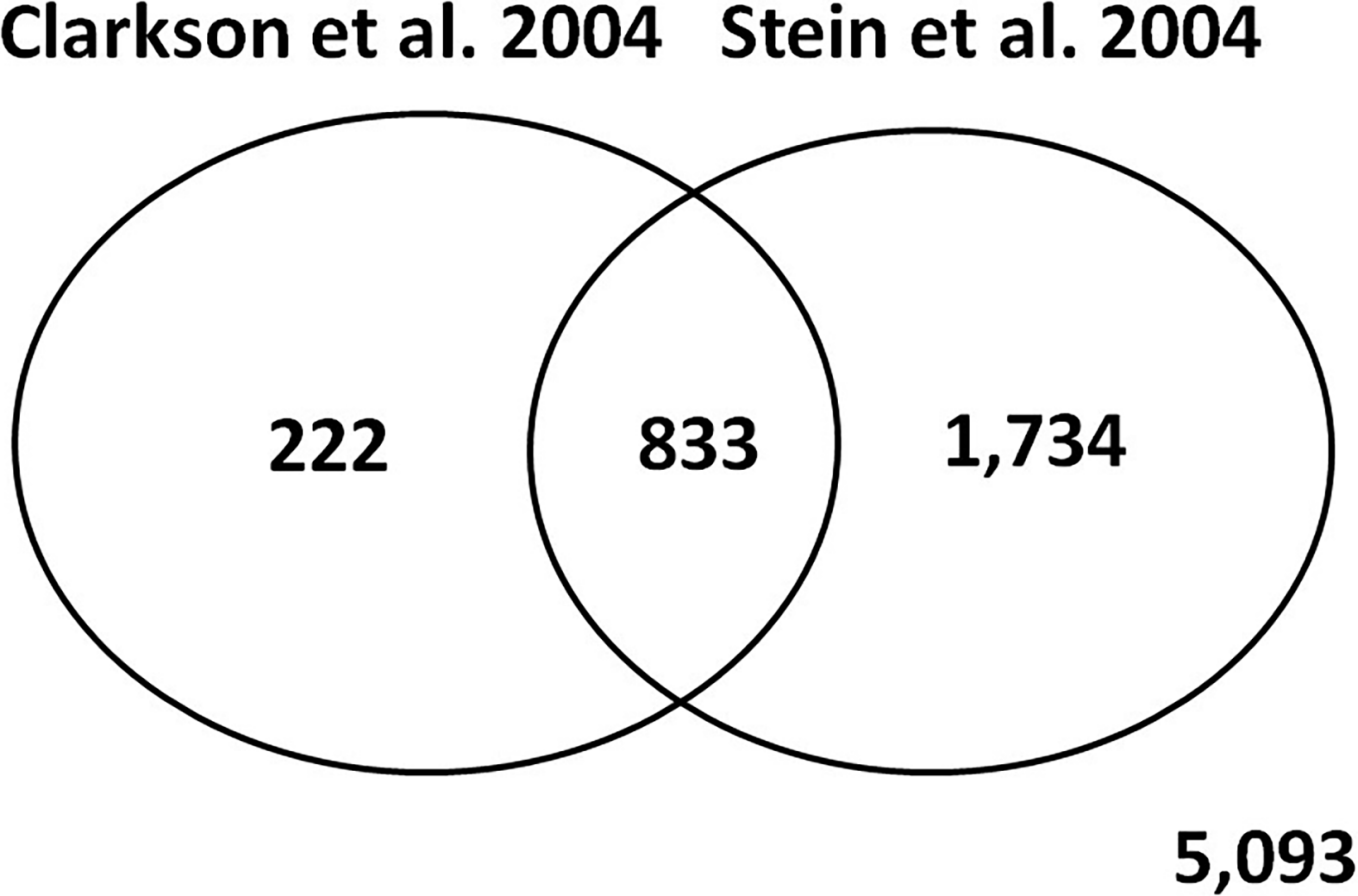
Venn diagram showing the overlap and discrepancies between genes differentially expressed in Clarkson et al. [3] and Stein et al. [4].

We used the limma package [25] from the Bioconductor project to identify differentially expressed genes across time points from lactation day 10 to involution day 4. We identified 1,055 differentially expressed genes across time points from lactation day 10 to involution day 4 in the data from Clarkson et al. [3]. To verify our results, we conducted an analysis for differentially expressed genes using data from Stein et al. [4] and identified 2,567 genes differentially expressed across time points from lactation day 7 to involution day 20. 79% of the genes identified as differentially expressed in the data from both studies. Fig 1 shows the Venn diagram of the overlap and discrepancies between genes differentially expressed in [3] and [4].

#### Clusters of differentially expressed genes

**Fig 2:**
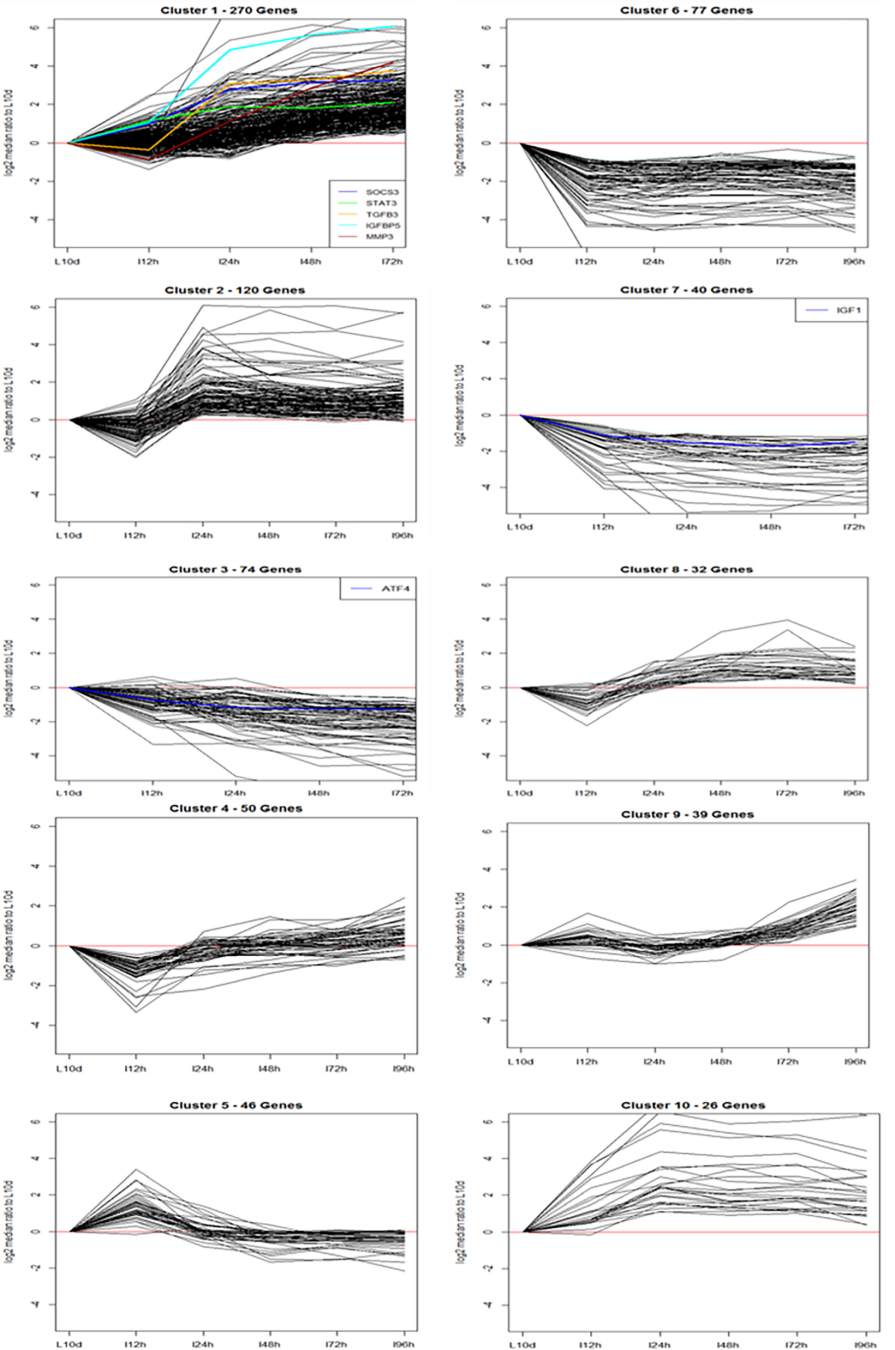
The log2 fold change in gene expression profiles for the ten significant clusters identified through the STEM Clustering. Y-axis represents the relative gene expression levels of involution days 0.5, 1, 2, 3 and 4 days compared lactation day 10 in log base 2 scale. X-axis represents the time points (L10, lactation day 10; I12h, involution day 0.5; I24h, involution day 1; I48h, involution day 2; I72h, involution day 3; I96h, involution day 4). SOCS3, suppressor of cytokine signaling 3; IGF1, insulin-like growth factor 1 (somatomedin C); STAT3, signal transducer and activator of transcription 3 (acute-phase response factor); TGFB3, transforming growth factor, beta 3; ATF4, activating transcription factor 4; IGFBP5, insulin-like growth factor binding protein 5; MMP3, matrix metallopeptidase 3.

Given the limited overlap between the two datasets, we sought to identify relevant gene clusters over time from the two published studies together. We used the STEM algorithm developed by Ernst et al. [26, 27] to cluster genes identified as differentially expressed between the last day of lactation and the last time point of involution. We used c = 3 and m = 50 for input parameters, where c indicates units of change and m the number of candidate profiles. This run significantly clustered 774 genes out of 1,055 differentially expressed genes (73.4%). Table 2 lists the patterns, size, and *p*-value of significant clusters out of 50 possible cluster profiles. Patterns indicate the log2 fold change in expression of genes in clusters compared to the lactation day 10 levels. Fig 2 shows the log2 fold change in gene expression profiles for the ten significant clusters.

**Table 2:** Patterns, size and *p*-values of significant clusters identified through the STEM algorithm using data from Clarkson et al. [3]. The far right column indicates Involution Specific Gene Signatures through the STEM clustering using data from Clarkson et al. [3].

Cluster	Pattern*	Size	*p*-Value	Number of human orthologous genes identified	Signature name
#1	0,1,2,3,4,5	270	1.30E-234	256	Inv1
#2	0,-1,2,1,1,1	120	1.10E-54	118	Inv2
#3	0,-1,-2.-3.-4.-5	74	3.00E-26	69	Inv3
#4	0,-3,-1,-1,0,1	50	5.10E-11	47	Inv4
#5	0,3,0,-3,-3,-2	46	7.30E-08	43	Inv5
#6	0,-3,-4,-2,-3,-3	77	1.20E-07	76	Inv6
#7	0,-3,-4,-5,-6,-3	40	2.30E-05	39	Inv7
#8	0,-2,1,2,4,1	32	1.10E-04	31	Inv8
#9	0,2,1,0,2,5	39	5.70E-04	37	Inv9
#10	0,2,5,3,6,3	26	7.30E-04	26	Inv10

*Pattern indicates the log2 fold change in gene expression levels of lactation day 10 and involution days 0.5, 1, 2, 3 and 4 days compared lactation day 10.

#### Involution-specific gene signatures

To use the involution-specific gene signatures to conduct gene set analysis on human IBC and non-IBC gene expression profiles, we identified human orthologous genes for genes of significant clusters identified through STEM (S1 Table). Ortholog data were downloaded from the ENSEMBL website and orthologous genes were identified using ENSEMBL gene id. See Table 2 for a list of the number of human orthologous genes identified for each of 10 significant clusters.

### Gene set analysis of involution-specific gene signatures

#### Results of GSEA of involution-specific signatures in IBC versus non-IBC

**Fig 3:**
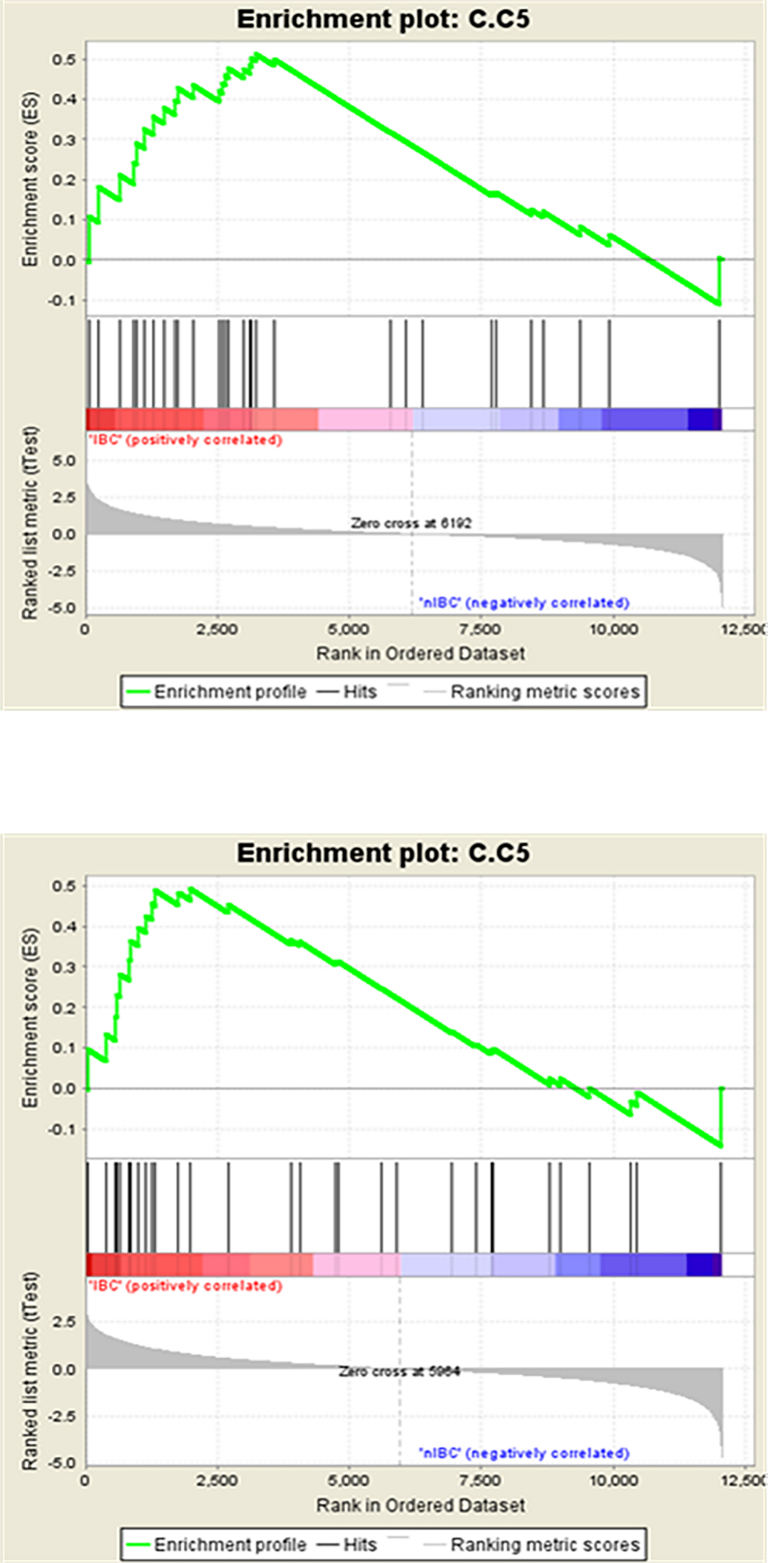
Enrichment plots form GSEA for Inv5 signature for IBC versus non-IBC in the training and validation sets. nIBC, non-Inflammatory breast cancer.

Out of 10 gene signatures developed through the STEM clustering using data from Clarkson et al. [3], 7 signatures have a normalized enrichment score (NES) >1 in the training set of IBC phenotype with 2 signatures, Inv5 and Inv6, having nominal p-values <0.05. In the validation set, we found 9 gene signatures with NES >1 in IBC cases with Inv5 having a nominal p-value of <0.05. Three signatures had NES >1 in non-IBC phenotype with nominal p-values >0.05 and no gene signatures significant at FDR <25% in the training set (Table 3). In the validation set, 1 gene signature had NES >1 in non-IBC phenotype with no gene signature significant at FDR <25% (Table 3). When comparing the results in the training and validation sets, we found that 6 out of 10 gene signatures were enriched in IBC in both training and validation sets. In the merged analysis repeated using the entire data set, we found that 2 out of 10 gene signatures (Inv5 and Inv6) were significantly upregulated in IBC versus non-IBC phenotype at nominal p-value of 0.05 (Table 4). Fig 3 represents the enrichment plot from the GSEA for Inv5 signature in IBC versus non-IBC and Table 5 shows the list of genes in the Inv5 signature as well as genes enriched in IBC. For the involution specific signatures reported by Stein et al. [4], we found that no gene signature was significantly enriched in IBC or non-IBC at FDR <25% or nominal p-value of 0.05 in both the training and validation sets (Table 3) or in the total data set (Table 4).

**Table 3:** GSEA results of involution-specific signatures in IBC versus non-IBC in the training and validation sets.

Gene Signature	Results on Training Set						Results on Validation Set					
	Enriched in IBC vs non-IBC	Size	ES	NES	Nominal p-value	FDR q-value	Enriched in IBC vs non-IBC	Size	ES	NES	Nominal p-value	FDR q-value
Involution specific signatures developed through STEM clustering using data from Clarkson et al. 2004												
Inv1	IBC	205	0.360	1.064	0.415	0.443	IBC	205	0.212	0.643	0.912	0.927
Inv2	Non-IBC	100	-0.139	-0.552	0.933	0.976	IBC	100	0.169	0.674	0.849	1.000
Inv3	Non-IBC	57	-0.299	-0.917	0.625	1.000	IBC	57	0.322	1.030	0.403	0.836
Inv4	IBC	33	0.392	1.108	0.360	0.469	IBC	33	0.307	0.907	0.581	0.835
Inv5	IBC	30	0.514	1.492	0.014	0.263	IBC	30	0.492	1.436	0.028	0.433
Inv6	IBC	62	0.402	1.399	0.043	0.222	IBC	62	0.326	1.122	0.258	1.000
Inv7	IBC	36	0.295	0.843	0.719	0.650	Non-IBC	36	-0.284	-0.810	0.790	0.709
Inv8	Non-IBC	24	-0.294	-0.883	0.647	0.897	IBC	24	0.294	0.878	0.621	0.774
Inv9	IBC	33	0.611	1.363	0.131	0.184	IBC	33	0.409	0.915	0.592	0.975
Inv10	IBC	21	0.296	0.897	0.583	0.654	IBC	21	0.339	1.030	0.414	1.000
Involution specific signatures reported in Stein et al. 2009												
S.C1	IBC	182	0.42	1.16	0.274	0.818	IBC	182	0.35	0.94	0.522	1
S.C2	Non-IBC	205	-0.27	-0.78	0.737	0.746	Non-IBC	205	-0.2	-0.63	0.945	0.962
S.C3	IBC	252	0.2	0.74	0.766	0.922	Non-IBC	252	-0.22	-0.81	0.695	1
S.C4	IBC	258	0.18	0.74	0.753	0.832	Non-IBC	258	-0.29	-1.25	0.215	0.718
S.C5.I3VL7	IBC	117	0.21	0.8	0.743	0.906	IBC	117	0.23	0.83	0.672	1
S.C6	Non-IBC	100	-0.25	-0.94	0.534	0.905	Non-IBC	100	-0.26	-1	0.438	0.715
S.C7	IBC	225	0.29	1.17	0.19	1	IBC	225	0.24	0.97	0.504	1
S.C8	Non-IBC	153	-0.25	-1.01	0.413	1	IBC	153	0.29	1.17	0.171	1
S.C9	Non-IBC	66	-0.25	-0.8	0.818	0.952	Non-IBC	66	-0.39	-1.24	0.149	0.365
S.I1VL7	IBC	495	0.19	0.85	0.693	1	Non-IBC	495	-0.16	-0.7	0.962	1
S.I2VL7	IBC	612	0.21	0.87	0.647	1	IBC	612	0.2	0.79	0.784	0.977
S.I3VL7	IBC	648	0.2	0.83	0.708	0.968	IBC	648	0.19	0.75	0.836	0.779
S.I4VL7	IBC	894	0.23	0.91	0.579	1	IBC	894	0.2	0.78	0.777	0.835

ES, enrichment score; NES, normalized enrichment score.

**Table 4:** GSEA results of involution-specific signatures in IBC versus non-IBC in the merged 3 breast cancer data sets.

Gene Signature	Merged 3 breast cancer data sets IBC = 137 and non-IBC = 251 # Genes = 12064					
	Enriched in IBC versus non-IBC	Size	ES	NES	Nominal p-value	FDR q-value
Involution specific signatures developed through STEM clustering using data from Clarkson et al. 2004						
Inv1	IBC	205	0.29	0.88	0.584	0.869
Inv2	IBC	100	0.13	0.52	0.97	0.988
Inv3	IBC	57	0.25	0.81	0.807	0.902
Inv4	IBC	33	0.32	0.94	0.541	0.873
Inv5	IBC	30	0.51	1.47	0.03	0.392
Inv6	IBC	62	0.42	1.47	0.021	0.204
Inv7	IBC	36	0.26	0.75	0.867	0.896
Inv8	Non-IBC	24	-0.3	-0.92	0.573	0.541
Inv9	IBC	33	0.54	1.22	0.279	0.407
Inv10	IBC	21	0.43	1.34	0.122	0.298
Involution specific signatures reported in Stein et al. 2009						
S.C1	IBC	182	0.4	1.08	0.379	0.614
S.C2	Non-IBC	205	-0.25	-0.77	0.735	0.902
S.C3	Non-IBC	252	-0.19	-0.72	0.783	0.849
S.C4	Non-IBC	258	-0.21	-0.89	0.568	1
S.C5.I3VL7	IBC	117	0.29	1.1	0.315	0.818
S.C6	Non-IBC	100	-0.26	-0.99	0.48	1
S.C7	IBC	225	0.28	1.13	0.26	1
S.C8	Non-IBC	153	-0.22	-0.88	0.717	0.835
S.C9	Non-IBC	66	-0.37	-1.21	0.189	0.847
S.I1VL7	IBC	495	0.19	0.83	0.717	0.639
S.I2VL7	IBC	612	0.21	0.87	0.671	0.798
S.I3VL7	IBC	648	0.21	0.84	0.694	0.729
S.I4VL7	IBC	894	0.23	0.9	0.591	0.882

**Table 5:** List of genes in Inv5 signature and genes enriched in IBC versus non-IBC.

Gene Symbol	Gene Name	Enrichment in IBC (Training Set)	Enrichment in IBC (Validation Set)
CD79B	CD79b molecule, immunoglobulin-associated beta	Yes	Yes
IVL	involucrin	Yes	Yes
KIF2C	kinesin family member 2C	Yes	Yes
NOP2	nucleolar protein 2	Yes	Yes
LDHB	lactate dehydrogenase B	Yes	Yes
LEP	leptin (obesity homolog, mouse)	Yes	Yes
AVIL	advillin	Yes	Yes
DKK2	dickkopf homolog 2 (Xenopus laevis)	Yes	Yes
ARAP3	ankyrin repeat and PH domain 3	Yes	Yes
YBX2	Y box binding protein 2	Yes	No
CEP250	centrosomal protein 250kDa	Yes	No
RPS6KB2	ribosomal protein S6 kinase, 70kDa, polypeptide 2	Yes	No
STX3	syntaxin 3	Yes	No
NFKBIE	nuclear factor of kappa light polypeptide gene enhancer in B-cells inhibitor, epsilon	Yes	No
GSTO1	glutathione S-transferase omega 1	Yes	No
WT1	Wilms tumor 1	Yes	No
DYNC1I1	dynein, cytoplasmic 1, intermediate chain 1	Yes	No
RET	ret proto-oncogene (multiple endocrine neoplasia and medullary thyroid carcinoma 1, Hirschsprung disease)	Yes	No
TIPIN	TIMELESS interacting protein	Yes	No
LLGL2	lethal giant larvae homolog 2 (Drosophila)	No	Yes
TG	thyroglobulin	No	Yes
DDIT4	DNA-damage-inducible transcript 4	No	Yes
HPCA	hippocalcin	No	Yes
GRAMD3	GRAM domain containing 3	No	No
PAX4	paired box gene 4	No	No
KCNJ4	potassium inwardly-rectifying channel, subfamily J, member 4	No	No
NPY	neuropeptide Y	No	No
CHRNA6	cholinergic receptor, nicotinic, alpha 6	No	No
FRAT2	frequently rearranged in advanced T-cell lymphomas 2	No	No
ERBB4	v-erb-a erythroblastic leukemia viral oncogene homolog 4 (avian)	No	No

#### Results of GSEA of involution-specific signatures in TN subtype versus non-TN subtype in IBC and non-IBC

Out of 10 gene signatures developed through STEM clustering using data from Clarkson et al. [3], we found that no gene signature was significantly enriched in TN subtype versus non-TN subtype in IBC cases at FDR <25% or nominal p-value of 0.05 in both the training and validation sets. For involution specific signatures reported by Stein et al. [4], we also did not find any gene signature that was significantly enriched in TN subtype versus non-TN subtype in IBC cases at FDR <25% or nominal p-value of 0.05 in both the training and validation sets. We found similar results when comparing gene signatures in TN subtype versus non-TN subtype in non-IBC cases.

#### GSEA results of involution-specific signatures in IBC versus non-IBC normals

Unfortunately there are not additional samples from which to validate the findings beyond the report above. Nevertheless, we speculated that the relevant signatures might be expressed in gene expression data from histopathologically normal breast isolated from IBC (N = 19) and non-IBC (N = 25) mastectomy patients given our hypothesis that the unique normal tissue changes influence the symptoms of IBC.

Out of 10 gene signatures developed through STEM clustering using data from Clarkson et al. [3], we found that one gene signature (Inv1) was significantly enriched in IBC versus non-IBC Normal samples and one gene signature (Inv5) was significantly enriched in non-IBC normal versus IBC samples at FDR <25% (0.202 and 0.249, respectively). For involution-specific signatures reported by Stein et al. [4], no gene signature that was significantly enriched in IBC versus non-IBC normal samples and in non-IBC normal versus IBC samples at FDR <25%.

This again demonstrates enrichment of involution signatures in human breast tissues from women with breast cancer, but the significant enrichment pattern is different in normal versus tumor. Again, this is limited by a small sample size (19 IBC and 25 non-IBC normals). Of the three genes mentioned from Inv5 tumor data as upregulated, only Leptin is upregulated in IBC in normal breast tissue. Of note, Inv1 genes with 2-fold enrichment are the following: CYP4B1, ACADL, PCK1, RASA3, CDO1, ABCA1 (see S4 Table for details). Interestingly, ABCA1, the primary cholesterol transporter in HDL trafficking previously described by our group as important in mediating the beneficial effects of HDL in IBC patients and pre-clinical studies is upregulated in IBC [32].

#### Ontology analysis for significant clusters

We also conducted ontology analysis on genes of significant clusters identified through the STEM using GO annotations for *mus musculus* gene products available from the Mouse Genome Informatics to understand biologically relevant processes. We did not find any significant biologic processes by the method for the IBC-enriched involution signature (S2 Table).

## Discussion

We reanalyzed the previously published expression profiling data set obtained from mammary glands derived from mice at various stages of post-lactational mammary gland involution (12 gene expression profiles with two hybridizations for each of 10 day lactation time points, and 12, 24, 48, 72 and 96 hour involution time points). We focused on genes that were differentially expressed during time periods spanning from the last day of lactation (day 10) to the fourth day of involution by greater than 2-fold (p <0.05) and performed STEM cluster analysis to discern time-varied expression patterns. We identified 10 time-based gene clusters that represent this time period, Inv1-10. Broadly, many are enriched versus depleted in IBC samples, but only Inv5 is significantly enriched in both the training and validation set based on nominal p-values, and note that FDR adjusted p-values are not significant for this cluster. Nevertheless, given the limitations of the data and the lack of larger datasets or similar extensive gene array studies of the involuting human breast, these hypothesis-generating findings may merit further study.

Up to now, the two most comprehensive studies examining global gene expression on the post-lactational mammary gland have been conducted by Clarkson et al. [3, 5] and Stein et al. [4]. Clarkson et al. [3] used the K means clustering method and Stein et al. used the self-organizing map in [4] and the hierarchical ordered partitioning and collapsing hybrid (HOPACH) method in [9] to find the patterns among differentially expressed genes in the post-lactational involution period. They discovered that apoptotic pathways and immunomodulatory signals are induced during the process of post-lactational involution. During our reanalysis, we used the STEM clustering method on the dataset by Clarkson et al. [3] to find the time-varied patterns among differentially expressed genes in the post-lactational involution period. We identified 10 separate and significant time-varied expression patterns from 774 genes out of 1,055 significantly differentially expressed genes. Gene ontology analyses of these clusters showed that cluster 1, which represented genes showing gradual up-regulation during the first 4 days of involution, had over-representation of numerous biological processes whereas relatively few are noted in other clusters. Representation of fatty acid oxidation and ER and membrane biology in Inv6 may be noteworthy given the nominal significance of Inv6 in analysis of the full data set and relevance of these systems in published IBC studies [28]. The over-representation of biological processes that we found are in general agreement with findings by Clarkson et al. [3], who used a different analytical approach, and with findings by Stein et al. [4, 9], who used a different mouse system and a different analytical approach.

We examined the enrichment of post-lactational mammary gland involution gene expression patterns in TNBC and IBC using the GSEA method. First, we used 10 significant time varied gene expression patterns that we found in the dataset from Clarkson et al. (3). We found that only one gene expression pattern was enriched in IBC compared to non-IBC in both training and validation sets at the nominal p-value. None of these gene expression patterns was enriched in TNBC compared to non-TN BC for both IBC and non-IBC groups in both training and validation sets. Second, we used 13 gene expression patterns on post-lactational involution as reported by Stein et al (9) and found that none of these gene expression patterns was significantly enriched in IBC compared to non-IBC and TN BC compared to non-TN BC in both training and validation sets. To investigate further, we examined the overlap between the involution-specific signatures and the IBC-like signature (79 genes) [28]. We found that there was minimal overlap between the involution-specific signatures and the IBC-like signature, and no gene overlapped between the Inv5 signature and the IBC-like signature (S3 Table).

One gene signature that showed nominal enrichment in IBC compared to non-IBC, Inv5, contained genes that showed initial up-regulation and later down-regulation during the involution process. This might suggest that genes that upregulate during an initial phase of involution after abrupt weaning might not be turning off and, therefore, could be responsible for facilitating an IBC-like phenotype after a tumor-initiating event. We examined the overlap of this gene expression pattern with the existing gene signatures using the Molecular Signatures Database (MSigDB) v4.0 [28]. We found that the genes up-regulated in VSMC by JNK1 [33] showed the most significant overlap (FDR q value = 6.07E-5). Among these overlapped genes, 3 genes–Involucrin (*IVL*), Cluster of Differentiation 79B (*CD79B*), and leptin (*LEP*)–were significantly enriched in IBC compared to non-IBC in both training and validation data sets in our analysis. Involucrin is a transglutaminase substrate protein present in keratinocytes of epidermis and other stratified squamous epithelia [34]. Tsuda et al [35] investigated the expression of Involucrin in breast cancer and found that Involucrin expression was detected in 27% of breast cancer cases and was associated with high-grade atypia, a solid-nest pattern, cancer cell necrosis on histology, and negative ER status. Leptin is a product of the obese (*OB*) gene, an important regulator of energy balance and necessary for normal mammary gland development [36]. In ER-positive breast cancer cell lines, leptin has been shown to stimulate cell growth through activation of multiple signaling pathways including the Janus Kinase/Signal Transducer and Activator of Transcription (JAK/STAT) pathway [37]. Thus, our results along with the published functions of the above-mentioned genes indicate that they play a role within the tumor microenvironment and may merit functional study of their role in promoting IBC development and progression.

A major strength of our study is the use of the largest series of IBC samples ever reported by the World IBC Consortium. Furthermore, this work is novel in part due to the use of updated Chip Definition Files from the BrainArray [25] during preprocessing of gene expression data for accurate probe mapping to the genome. Also, we used the GSEA [31] to examine enrichment of involution signatures in IBC and TNBC phenotype. The GSEA gives more statistical power to detect smaller changes in genes of a gene set compared to other methods of enrichment analysis.

Major limitations of our study include the cross-sectional analysis of enrichment of involution specific signatures in breast cancer and array-based measurement of gene expression profiles, which limit the detection of differentially expressed genes with lower levels of expression. Also, although having the parity status of the patients within the World IBC consortium would be valuable to our study, that information is not recorded or available to us and, as such, we cannot determine or comment on whether parity-related effects persist or were present prior to the time of analysis. Further, there are surprisingly few TNBC in the cohort given the established over-representation of these subtypes in IBC which may influence the findings overall and regarding TNBC versus non-TNBC. Additionally, the studies by Clarkson et al (3) and Stein et al (4) did not include a corresponding group that underwent non-abrupt involution or that was not force weaned, as the authors did not distinguish between abrupt involution versus the normal involution process. Thus, the correlation between limited nursing and abrupt involution signatures remains unstudied here. Although inclusion of such a control group in these studies would be useful to our analysis, we can speculate that Inv5 and *IVL*, *CD79B*, and *LEP* in particular may play in role in IBC development after abrupt or involution. Given the limitations of this study, however, additional research is warranted before a concrete conclusion can be made.

In conclusion, our results provide some evidence that molecular events after abrupt involution are identifiable in IBC patient tissues from the uninvolved breast and tumor; however, they are hypothesis-generating given the potential for false discovery after multiple comparisons as well as the other noted limitations of our study. Whether or not Inv5 or Inv6 related genes or signaling are upregulated in the normal tissues around IBC tumors, and if breast-feeding or abrupt cessation of breast-feeding contributes to the persistence of related genes in the normal breast will be investigated in future studies.

## Supporting information

S1 Table List of orthologous genes identified. (DOCX)

S2 Table Results of ontology analysis for the STEM significant clusters. BP, biological process; CC; cellular components.
(DOCX)

S3 Table Overlap of genes between involution-specific signatures and IBC-like signature. (DOCX)

S4 Table Signature analysis of the breast parenchyma from 19 IBC patients and 25 non-IBC patients. (DOCX)
