# Supplementary material for: Gene set analysis of post-lactational mammary gland involution gene signatures in inflammatory and triple-negative breast cancer
Source: PLoS One. 2018 Apr 4;13(4):e0192689. doi: 10.1371/journal.pone.0192689 (PMC5884491; doi:10.1371/journal.pone.0192689)
Supplement: S1 Table — (DOCX) [file pone.0192689.s001.docx]

| List of orthologous genes identified in Clarkson et al. [3]. | | |
| --- | --- | --- |
| SYMBOL | Gene Singature | GENENAME |
| 0610031j06rik | Inv2 | NA |
| 1190005i06rik | Inv1 | NA |
| 1810030o07rik | Inv2 | NA |
| 2610528a11rik | Inv8 | NA |
| 4933426m11rik | Inv1 | NA |
| Aass | Inv3 | aminoadipate-semialdehyde synthase |
| Abca1 | Inv1 | ATP-binding cassette, sub-family A (ABC1), member 1 |
| Abcf1 | Inv1 | ATP-binding cassette, sub-family F (GCN20), member 1 |
| Abcg1 | Inv9 | ATP-binding cassette, sub-family G (WHITE), member 1 |
| Abcg2 | Inv3 | ATP-binding cassette, sub-family G (WHITE), member 2 |
| Acaa1a | Inv1 | acetyl-Coenzyme A acyltransferase 1A |
| Acaa2 | Inv1 | acetyl-Coenzyme A acyltransferase 2 (mitochondrial 3-oxoacyl-Coenzyme A thiolase) |
| Acadl | Inv1 | acyl-Coenzyme A dehydrogenase, long-chain |
| Acadm | Inv1 | acyl-Coenzyme A dehydrogenase, medium chain |
| Acly | Inv7 | ATP citrate lyase |
| Acot1 | Inv2 | acyl-CoA thioesterase 1 |
| Acp5 | Inv1 | acid phosphatase 5, tartrate resistant |
| Acsl1 | Inv6 | acyl-CoA synthetase long-chain family member 1 |
| Acss1 | Inv6 | acyl-CoA synthetase short-chain family member 1 |
| Acta2 | Inv3 | actin, alpha 2, smooth muscle, aorta |
| Actn1 | Inv2 | actinin, alpha 1 |
| Actn4 | Inv2 | actinin alpha 4 |
| Adamdec1 | Inv9 | ADAM-like, decysin 1 |
| Adrb3 | Inv9 | adrenergic receptor, beta 3 |
| Agpat3 | Inv1 | 1-acylglycerol-3-phosphate O-acyltransferase 3 |
| Agrn | Inv2 | agrin |
| Ahcyl1 | Inv2 | S-adenosylhomocysteine hydrolase-like 1 |
| Ahnak | Inv10 | AHNAK nucleoprotein (desmoyokin) |
| Akap1 | Inv3 | A kinase (PRKA) anchor protein 1 |
| Akirin2 | Inv1 | akirin 2 |
| Akr1a1 | Inv2 | aldo-keto reductase family 1, member A1 (aldehyde reductase) |
| Akr1b8 | Inv10 | aldo-keto reductase family 1, member B8 |
| Alas1 | Inv1 | aminolevulinic acid synthase 1 |
| Aldh18a1 | Inv6 | aldehyde dehydrogenase 18 family, member A1 |
| Aldh1a1 | Inv9 | aldehyde dehydrogenase family 1, subfamily A1 |
| Aldh1a7 | Inv1 | aldehyde dehydrogenase family 1, subfamily A7 |
| Aldoc | Inv6 | aldolase C, fructose-bisphosphate |
| Anapc5 | Inv2 | anaphase-promoting complex subunit 5 |
| Angptl2 | Inv4 | angiopoietin-like 2 |
| Angptl4 | Inv2 | angiopoietin-like 4 |
| Ankrd33b | Inv6 | ankyrin repeat domain 33B |
| Anpep | Inv1 | alanyl (membrane) aminopeptidase |
| Anxa1 | Inv1 | annexin A1 |
| Anxa2 | Inv1 | annexin A2 |
| Anxa3 | Inv2 | annexin A3 |
| Anxa4 | Inv2 | annexin A4 |
| Anxa5 | Inv2 | annexin A5 |
| Aoc3 | Inv1 | amine oxidase, copper containing 3 |
| Ap4s1 | Inv4 | adaptor-related protein complex AP-4, sigma 1 |
| Apbb1ip | Inv1 | amyloid beta (A4) precursor protein-binding, family B, member 1 interacting protein |
| Apobec1 | Inv1 | apolipoprotein B mRNA editing enzyme, catalytic polypeptide 1 |
| Apoe | Inv1 | apolipoprotein E |
| App | Inv8 | amyloid beta (A4) precursor protein |
| Arap3 | Inv5 | ArfGAP with RhoGAP domain, ankyrin repeat and PH domain 3 |
| Arf1 | Inv2 | ADP-ribosylation factor 1 |
| Arf3 | Inv6 | ADP-ribosylation factor 3 |
| Arg2 | Inv3 | arginase type II |
| Arglu1 | Inv4 | arginine and glutamate rich 1 |
| Arhgef3 | Inv4 | Rho guanine nucleotide exchange factor (GEF) 3 |
| Arpc2 | Inv2 | actin related protein 2/3 complex, subunit 2 |
| Arpc3 | Inv1 | actin related protein 2/3 complex, subunit 3 |
| Arxes2 | Inv1 | adipocyte-related X-chromosome expressed sequence 2 |
| Asns | Inv3 | asparagine synthetase |
| Atf4 | Inv3 | activating transcription factor 4 |
| Atl2 | Inv3 | atlastin GTPase 2 |
| Atox1 | Inv2 | ATX1 (antioxidant protein 1) homolog 1 (yeast) |
| Atp11a | Inv1 | ATPase, class VI, type 11A |
| Atp1b3 | Inv2 | ATPase, Na+/K+ transporting, beta 3 polypeptide |
| Atp2b2 | Inv6 | ATPase, Ca++ transporting, plasma membrane 2 |
| Atxn10 | Inv2 | ataxin 10 |
| Au040320 | Inv2 | NA |
| Avil | Inv5 | advillin |
| Azin1 | Inv3 | antizyme inhibitor 1 |
| Baiap2l1 | Inv3 | BAI1-associated protein 2-like 1 |
| Banf1 | Inv2 | barrier to autointegration factor 1 |
| Basp1 | Inv10 | brain abundant, membrane attached signal protein 1 |
| Bc004004 | Inv6 | NA |
| Bcap31 | Inv4 | B cell receptor associated protein 31 |
| Bcar1 | Inv6 | breast cancer anti-estrogen resistance 1 |
| Bckdha | Inv7 | branched chain ketoacid dehydrogenase E1, alpha polypeptide |
| Bgn | Inv1 | biglycan |
| Brk1 | Inv2 | BRICK1, SCAR/WAVE actin-nucleating complex subunit |
| Btbd17 | Inv5 | BTB (POZ) domain containing 17 |
| Btn1a1 | Inv3 | butyrophilin, subfamily 1, member A1 |
| Bud31 | Inv2 | BUD31 homolog (yeast) |
| Bzw2 | Inv2 | basic leucine zipper and W2 domains 2 |
| C1qa | Inv1 | complement component 1, q subcomponent, alpha polypeptide |
| C1qb | Inv1 | complement component 1, q subcomponent, beta polypeptide |
| C1qc | Inv1 | complement component 1, q subcomponent, C chain |
| C3 | Inv1 | complement component 3 |
| C3ar1 | Inv9 | complement component 3a receptor 1 |
| Cab39l | Inv3 | calcium binding protein 39-like |
| Capn5 | Inv6 | calpain 5 |
| Capns1 | Inv2 | calpain, small subunit 1 |
| Capza2 | Inv1 | capping protein (actin filament) muscle Z-line, alpha 2 |
| Casp1 | Inv2 | caspase 1 |
| Casp3 | Inv6 | caspase 3 |
| Ccdc80 | Inv1 | coiled-coil domain containing 80 |
| Cck | Inv6 | cholecystokinin |
| Ccl2 | Inv1 | chemokine (C-C motif) ligand 2 |
| Ccl8 | Inv1 | chemokine (C-C motif) ligand 8 |
| Ccl9 | Inv1 | chemokine (C-C motif) ligand 9 |
| Ccr2 | Inv1 | chemokine (C-C motif) receptor 2 |
| Cct3 | Inv10 | chaperonin containing Tcp1, subunit 3 (gamma) |
| Cct4 | Inv2 | chaperonin containing Tcp1, subunit 4 (delta) |
| Cd14 | Inv10 | CD14 antigen |
| Cd151 | Inv2 | CD151 antigen |
| Cd320 | Inv3 | CD320 antigen |
| Cd47 | Inv1 | CD47 antigen (Rh-related antigen, integrin-associated signal transducer) |
| Cd48 | Inv1 | CD48 antigen |
| Cd53 | Inv1 | CD53 antigen |
| Cd68 | Inv1 | CD68 antigen |
| Cd74 | Inv4 | CD74 antigen (invariant polypeptide of major histocompatibility complex,  class II antigen-associated) |
| Cd79b | Inv5 | CD79B antigen |
| Cd81 | Inv8 | CD81 antigen |
| Cdc5l | Inv4 | cell division cycle 5-like (S. pombe) |
| Cdh1 | Inv10 | cadherin 1 |
| Cdh5 | Inv1 | cadherin 5 |
| Cdk4 | Inv2 | cyclin-dependent kinase 4 |
| Cdo1 | Inv1 | cysteine dioxygenase 1, cytosolic |
| Cebpa | Inv9 | CCAAT/enhancer binding protein (C/EBP), alpha |
| Cecr5 | Inv3 | cat eye syndrome chromosome region, candidate 5 |
| Cel | Inv3 | carboxyl ester lipase |
| Cep250 | Inv5 | centrosomal protein 250 |
| Cfp | Inv1 | complement factor properdin |
| Chchd7 | Inv3 | coiled-coil-helix-coiled-coil-helix domain containing 7 |
| Chd4 | Inv1 | chromodomain helicase DNA binding protein 4 |
| Chil1 | Inv1 | chitinase-like 1 |
| Chmp2a | Inv2 | charged multivesicular body protein 2A |
| Chrna6 | Inv5 | cholinergic receptor, nicotinic, alpha polypeptide 6 |
| Chtop | Inv4 | chromatin target of PRMT1 |
| Cib1 | Inv2 | calcium and integrin binding 1 (calmyrin) |
| Cidea | Inv3 | cell death-inducing DNA fragmentation factor, alpha subunit-like effector A |
| Cidec | Inv9 | cell death-inducing DFFA-like effector c |
| Cited2 | Inv8 | Cbp/p300-interacting transactivator, with Glu/Asp-rich carboxy-terminal domain, 2 |
| Clcn3 | Inv6 | chloride channel 3 |
| Cldn8 | Inv6 | claudin 8 |
| Clic1 | Inv10 | chloride intracellular channel 1 |
| Clk1 | Inv1 | CDC-like kinase 1 |
| Cmbl | Inv1 | carboxymethylenebutenolidase-like (Pseudomonas) |
| Cmtm6 | Inv6 | CKLF-like MARVEL transmembrane domain containing 6 |
| Cnn1 | Inv3 | calponin 1 |
| Cnn3 | Inv1 | calponin 3, acidic |
| Cnpy2 | Inv2 | canopy 2 homolog (zebrafish) |
| Coa3 | Inv2 | cytochrome C oxidase assembly factor 3 |
| Col15a1 | Inv4 | collagen, type XV, alpha 1 |
| Col1a2 | Inv1 | collagen, type I, alpha 2 |
| Col4a1 | Inv1 | collagen, type IV, alpha 1 |
| Col4a2 | Inv1 | collagen, type IV, alpha 2 |
| Col5a2 | Inv1 | collagen, type V, alpha 2 |
| Col6a1 | Inv1 | collagen, type VI, alpha 1 |
| Col6a3 | Inv1 | collagen, type VI, alpha 3 |
| Commd4 | Inv2 | COMM domain containing 4 |
| Commd7 | Inv2 | COMM domain containing 7 |
| Coro1a | Inv1 | coronin, actin binding protein 1A |
| Coro1c | Inv1 | coronin, actin binding protein 1C |
| Cp | Inv2 | ceruloplasmin |
| Cpe | Inv9 | carboxypeptidase E |
| Cpt1a | Inv1 | carnitine palmitoyltransferase 1a, liver |
| Cpt2 | Inv1 | carnitine palmitoyltransferase 2 |
| Creb3l1 | Inv6 | cAMP responsive element binding protein 3-like 1 |
| Crhr1 | Inv6 | corticotropin releasing hormone receptor 1 |
| Cryab | Inv2 | crystallin, alpha B |
| Cs | Inv7 | citrate synthase |
| Csf1r | Inv1 | colony stimulating factor 1 receptor |
| Csnk1a1 | Inv8 | casein kinase 1, alpha 1 |
| Cstb | Inv1 | cystatin B |
| Ctgf | Inv1 | connective tissue growth factor |
| Ctnna1 | Inv1 | catenin (cadherin associated protein), alpha 1 |
| Ctps | Inv2 | cytidine 5'-triphosphate synthase |
| Ctsc | Inv4 | cathepsin C |
| Ctsd | Inv2 | cathepsin D |
| Ctsh | Inv8 | cathepsin H |
| Ctss | Inv1 | cathepsin S |
| Cttn | Inv1 | cortactin |
| Cx3cl1 | Inv1 | chemokine (C-X3-C motif) ligand 1 |
| Cxcl12 | Inv9 | chemokine (C-X-C motif) ligand 12 |
| Cyb5b | Inv6 | cytochrome b5 type B |
| Cyba | Inv1 | cytochrome b-245, alpha polypeptide |
| Cyp1b1 | Inv1 | cytochrome P450, family 1, subfamily b, polypeptide 1 |
| Cyp2d10 | Inv2 | cytochrome P450, family 2, subfamily d, polypeptide 10 |
| Cyp2f2 | Inv9 | cytochrome P450, family 2, subfamily f, polypeptide 2 |
| Cyp4b1 | Inv1 | cytochrome P450, family 4, subfamily b, polypeptide 1 |
| Cyp4v3 | Inv1 | cytochrome P450, family 4, subfamily v, polypeptide 3 |
| Cytip | Inv3 | cytohesin 1 interacting protein |
| D15ertd621e | Inv1 | NA |
| D4wsu53e | Inv1 | NA |
| Dab2 | Inv6 | disabled 2, mitogen-responsive phosphoprotein |
| Dad1 | Inv6 | defender against cell death 1 |
| Dap | Inv6 | death-associated protein |
| Dazap2 | Inv2 | DAZ associated protein 2 |
| Dbnl | Inv2 | drebrin-like |
| Dctn3 | Inv2 | dynactin 3 |
| Dctn6 | Inv1 | dynactin 6 |
| Ddah2 | Inv1 | dimethylarginine dimethylaminohydrolase 2 |
| Ddb1 | Inv2 | damage specific DNA binding protein 1 |
| Ddit4 | Inv5 | DNA-damage-inducible transcript 4 |
| Ddx39b | Inv8 | DEAD (Asp-Glu-Ala-Asp) box polypeptide 39B |
| Decr1 | Inv1 | 2,4-dienoyl CoA reductase 1, mitochondrial |
| Degs1 | Inv1 | degenerative spermatocyte homolog 1 (Drosophila) |
| Dgcr6 | Inv2 | DiGeorge syndrome critical region gene 6 |
| Dhodh | Inv6 | dihydroorotate dehydrogenase |
| Dhrs3 | Inv1 | dehydrogenase/reductase (SDR family) member 3 |
| Dkk2 | Inv5 | dickkopf homolog 2 (Xenopus laevis) |
| Dmrtb1 | Inv5 | DMRT-like family B with proline-rich C-terminal, 1 |
| Dock9 | Inv1 | dedicator of cytokinesis 9 |
| Dpep1 | Inv9 | dipeptidase 1 (renal) |
| Dpt | Inv9 | dermatopontin |
| Dpy30 | Inv1 | dpy-30 homolog (C. elegans) |
| Drd4 | Inv6 | dopamine receptor D4 |
| Dync1h1 | Inv2 | dynein cytoplasmic 1 heavy chain 1 |
| Dync1i1 | Inv5 | dynein cytoplasmic 1 intermediate chain 1 |
| Ear2 | Inv1 | eosinophil-associated, ribonuclease A family, member 2 |
| Ech1 | Inv1 | enoyl coenzyme A hydratase 1, peroxisomal |
| Eci2 | Inv1 | enoyl-Coenzyme A delta isomerase 2 |
| Edem3 | Inv6 | ER degradation enhancer, mannosidase alpha-like 3 |
| Eef2k | Inv6 | eukaryotic elongation factor-2 kinase |
| Eepd1 | Inv6 | endonuclease/exonuclease/phosphatase family domain containing 1 |
| Egf | Inv3 | epidermal growth factor |
| Egr1 | Inv1 | early growth response 1 |
| Ehf | Inv3 | ets homologous factor |
| Ehhadh | Inv7 | enoyl-Coenzyme A, hydratase/3-hydroxyacyl Coenzyme A dehydrogenase |
| Eid1 | Inv4 | EP300 interacting inhibitor of differentiation 1 |
| Eif1 | Inv1 | eukaryotic translation initiation factor 1 |
| Eif3d | Inv2 | eukaryotic translation initiation factor 3, subunit D |
| Elf5 | Inv3 | E74-like factor 5 |
| Ell2 | Inv7 | elongation factor RNA polymerase II 2 |
| Elovl1 | Inv6 | elongation of very long chain fatty acids (FEN1/Elo2, SUR4/Elo3, yeast)-like 1 |
| Elovl5 | Inv7 | ELOVL family member 5, elongation of long chain fatty acids (yeast) |
| Emp1 | Inv1 | epithelial membrane protein 1 |
| Entpd1 | Inv1 | ectonucleoside triphosphate diphosphohydrolase 1 |
| Epb4.1l2 | Inv1 | erythrocyte protein band 4.1-like 2 |
| Erbb4 | Inv5 | v-erb-a erythroblastic leukemia viral oncogene homolog 4 (avian) |
| Errfi1 | Inv1 | ERBB receptor feedback inhibitor 1 |
| Esd | Inv2 | esterase D/formylglutathione hydrolase |
| Etfb | Inv1 | electron transferring flavoprotein, beta polypeptide |
| Ets2 | Inv1 | E26 avian leukemia oncogene 2, 3' domain |
| Evi2a | Inv1 | ecotropic viral integration site 2a |
| F3 | Inv1 | coagulation factor III |
| Fads1 | Inv6 | fatty acid desaturase 1 |
| Fah | Inv3 | fumarylacetoacetate hydrolase |
| Fam101b | Inv1 | family with sequence similarity 101, member B |
| Fam105a | Inv9 | family with sequence similarity 105, member A |
| Fam110a | Inv3 | family with sequence similarity 110, member A |
| Fam134b | Inv3 | family with sequence similarity 134, member B |
| Fam32a | Inv2 | family with sequence similarity 32, member A |
| Fam3c | Inv1 | family with sequence similarity 3, member C |
| Fam76b | Inv1 | family with sequence similarity 76, member B |
| Fbln2 | Inv1 | fibulin 2 |
| Fbn1 | Inv8 | fibrillin 1 |
| Fcer1g | Inv1 | Fc receptor, IgE, high affinity I, gamma polypeptide |
| Fcgr2b | Inv1 | Fc receptor, IgG, low affinity IIb |
| Fermt3 | Inv1 | fermitin family homolog 3 (Drosophila) |
| Fgl2 | Inv1 | fibrinogen-like protein 2 |
| Fkbp11 | Inv6 | FK506 binding protein 11 |
| Fkbp7 | Inv4 | FK506 binding protein 7 |
| Fkbp9 | Inv1 | FK506 binding protein 9 |
| Flot1 | Inv1 | flotillin 1 |
| Fmr1 | Inv1 | fragile X mental retardation syndrome 1 |
| Fn1 | Inv1 | fibronectin 1 |
| Folr1 | Inv3 | folate receptor 1 (adult) |
| Folr2 | Inv9 | folate receptor 2 (fetal) |
| Frat2 | Inv5 | frequently rearranged in advanced T cell lymphomas 2 |
| Fstl1 | Inv1 | follistatin-like 1 |
| Fth1 | Inv1 | ferritin heavy chain 1 |
| Fxyd3 | Inv1 | FXYD domain-containing ion transport regulator 3 |
| G0s2 | Inv7 | G0/G1 switch gene 2 |
| Gadd45a | Inv1 | growth arrest and DNA-damage-inducible 45 alpha |
| Galnt2 | Inv1 | UDP-N-acetyl-alpha-D-galactosamine:polypeptide  N-acetylgalactosaminyltransferase 2 |
| Gar1 | Inv2 | GAR1 ribonucleoprotein homolog (yeast) |
| Gas1 | Inv4 | growth arrest specific 1 |
| Gas6 | Inv1 | growth arrest specific 6 |
| Gbp2 | Inv1 | guanylate binding protein 2 |
| Gja1 | Inv1 | gap junction protein, alpha 1 |
| Gjb1 | Inv3 | gap junction protein, beta 1 |
| Gjb2 | Inv2 | gap junction protein, beta 2 |
| Gldc | Inv6 | glycine decarboxylase |
| Glt25d1 | Inv10 | glycosyltransferase 25 domain containing 1 |
| Gltp | Inv1 | glycolipid transfer protein |
| Gna15 | Inv7 | guanine nucleotide binding protein, alpha 15 |
| Gnai2 | Inv8 | guanine nucleotide binding protein (G protein), alpha inhibiting 2 |
| Gnb4 | Inv3 | guanine nucleotide binding protein (G protein), beta 4 |
| Gne | Inv7 | glucosamine (UDP-N-acetyl)-2-epimerase/N-acetylmannosamine kinase |
| Gnmt | Inv3 | glycine N-methyltransferase |
| Gpd1 | Inv4 | glycerol-3-phosphate dehydrogenase 1 (soluble) |
| Gphn | Inv7 | gephyrin |
| Gpr125 | Inv9 | G protein-coupled receptor 125 |
| Gpr65 | Inv9 | G-protein coupled receptor 65 |
| Gramd3 | Inv5 | GRAM domain containing 3 |
| Grcc10 | Inv4 | gene rich cluster, C10 gene |
| Grn | Inv2 | granulin |
| Gsn | Inv1 | gelsolin |
| Gstm1 | Inv9 | glutathione S-transferase, mu 1 |
| Gsto1 | Inv5 | glutathione S-transferase omega 1 |
| Gtf3a | Inv2 | general transcription factor III A |
| Gtpbp4 | Inv1 | GTP binding protein 4 |
| Gyk | Inv3 | glycerol kinase |
| H2-aa | Inv1 | NA |
| H2-ab1 | Inv1 | NA |
| H2-eb1 | Inv1 | NA |
| H2afv | Inv1 | H2A histone family, member V |
| H3f3b | Inv10 | H3 histone, family 3B |
| Hbs1l | Inv3 | Hbs1-like (S. cerevisiae) |
| Hebp1 | Inv1 | heme binding protein 1 |
| Hexa | Inv1 | hexosaminidase A |
| Hiat1 | Inv6 | hippocampus abundant gene transcript 1 |
| Hk2 | Inv7 | hexokinase 2 |
| Hnrnpm | Inv1 | heterogeneous nuclear ribonucleoprotein M |
| Homer2 | Inv3 | homer homolog 2 (Drosophila) |
| Hp | Inv1 | haptoglobin |
| Hp1bp3 | Inv4 | heterochromatin protein 1, binding protein 3 |
| Hpca | Inv5 | hippocalcin |
| Hpgd | Inv1 | hydroxyprostaglandin dehydrogenase 15 (NAD) |
| Hsd17b11 | Inv1 | hydroxysteroid (17-beta) dehydrogenase 11 |
| Hsd17b4 | Inv1 | hydroxysteroid (17-beta) dehydrogenase 4 |
| Hsd17b7 | Inv6 | hydroxysteroid (17-beta) dehydrogenase 7 |
| Hsp90b1 | Inv2 | heat shock protein 90, beta (Grp94), member 1 |
| Hspa4 | Inv1 | heat shock protein 4 |
| Htra1 | Inv9 | HtrA serine peptidase 1 |
| Ica1 | Inv7 | islet cell autoantigen 1 |
| Idh1 | Inv1 | isocitrate dehydrogenase 1 (NADP+), soluble |
| Ifi27 | Inv8 | interferon, alpha-inducible protein 27 |
| Ifit1 | Inv1 | interferon-induced protein with tetratricopeptide repeats 1 |
| Ifitm3 | Inv1 | interferon induced transmembrane protein 3 |
| Ifnar2 | Inv1 | interferon (alpha and beta) receptor 2 |
| Igf1 | Inv7 | insulin-like growth factor 1 |
| Igfals | Inv6 | insulin-like growth factor binding protein, acid labile subunit |
| Igfbp4 | Inv1 | insulin-like growth factor binding protein 4 |
| Igfbp5 | Inv1 | insulin-like growth factor binding protein 5 |
| Il15 | Inv6 | interleukin 15 |
| Inhbb | Inv1 | inhibin beta-B |
| Inmt | Inv9 | indolethylamine N-methyltransferase |
| Inpp5k | Inv6 | inositol polyphosphate 5-phosphatase K |
| Irf8 | Inv9 | interferon regulatory factor 8 |
| Irgm2 | Inv1 | immunity-related GTPase family M member 2 |
| Isg20 | Inv7 | interferon-stimulated protein |
| Isyna1 | Inv1 | myo-inositol 1-phosphate synthase A1 |
| Itgb2 | Inv9 | integrin beta 2 |
| Itm2a | Inv9 | integral membrane protein 2A |
| Itm2b | Inv1 | integral membrane protein 2B |
| Itm2c | Inv1 | integral membrane protein 2C |
| Ivl | Inv5 | involucrin |
| Jtb | Inv3 | jumping translocation breakpoint |
| Jup | Inv2 | junction plakoglobin |
| Kank3 | Inv1 | KN motif and ankyrin repeat domains 3 |
| Kansl2 | Inv3 | KAT8 regulatory NSL complex subunit 2 |
| Kcnj4 | Inv5 | potassium inwardly-rectifying channel, subfamily J, member 4 |
| Kcnk1 | Inv3 | potassium channel, subfamily K, member 1 |
| Kcnn4 | Inv6 | potassium intermediate/small conductance calcium-activated channel,  subfamily N, member 4 |
| Kctd10 | Inv1 | potassium channel tetramerisation domain containing 10 |
| Kctd12 | Inv1 | potassium channel tetramerisation domain containing 12 |
| Kdm3a | Inv1 | lysine (K)-specific demethylase 3A |
| Kif2c | Inv5 | kinesin family member 2C |
| Kit | Inv1 | kit oncogene |
| Kitl | Inv4 | kit ligand |
| Klf10 | Inv1 | Kruppel-like factor 10 |
| Klrk1 | Inv5 | killer cell lectin-like receptor subfamily K, member 1 |
| Kpnb1 | Inv1 | karyopherin (importin) beta 1 |
| Krt18 | Inv1 | keratin 18 |
| Lalba | Inv3 | lactalbumin, alpha |
| Lamc2 | Inv2 | laminin, gamma 2 |
| Laptm5 | Inv1 | lysosomal-associated protein transmembrane 5 |
| Large | Inv10 | like-glycosyltransferase |
| Lbp | Inv2 | lipopolysaccharide binding protein |
| Lcn4 | Inv5 | lipocalin 4 |
| Lcp2 | Inv9 | lymphocyte cytosolic protein 2 |
| Ldhb | Inv5 | lactate dehydrogenase B |
| Lefty1 | Inv6 | left right determination factor 1 |
| Lep | Inv5 | leptin |
| Lgals1 | Inv1 | lectin, galactose binding, soluble 1 |
| Lgals3 | Inv1 | lectin, galactose binding, soluble 3 |
| Lgals3bp | Inv1 | lectin, galactoside-binding, soluble, 3 binding protein |
| Lgals9 | Inv1 | lectin, galactose binding, soluble 9 |
| Lgi4 | Inv5 | leucine-rich repeat LGI family, member 4 |
| Lgmn | Inv1 | legumain |
| Lima1 | Inv1 | LIM domain and actin binding 1 |
| Litaf | Inv10 | LPS-induced TN factor |
| Llgl2 | Inv5 | lethal giant larvae homolog 2 (Drosophila) |
| Lman1 | Inv6 | lectin, mannose-binding, 1 |
| Lmo2 | Inv6 | LIM domain only 2 |
| Lonp2 | Inv4 | lon peptidase 2, peroxisomal |
| Lpin1 | Inv6 | lipin 1 |
| Lrg1 | Inv1 | leucine-rich alpha-2-glycoprotein 1 |
| Lrp1 | Inv1 | low density lipoprotein receptor-related protein 1 |
| Lsm4 | Inv8 | LSM4 homolog, U6 small nuclear RNA associated (S. cerevisiae) |
| Lsr | Inv2 | lipolysis stimulated lipoprotein receptor |
| Lst1 | Inv1 | leukocyte specific transcript 1 |
| Ltf | Inv8 | lactotransferrin |
| Lum | Inv1 | lumican |
| Ly86 | Inv1 | lymphocyte antigen 86 |
| Lyz2 | Inv1 | lysozyme 2 |
| Man2b1 | Inv6 | mannosidase 2, alpha B1 |
| Mapk3 | Inv2 | mitogen-activated protein kinase 3 |
| Marcks | Inv1 | myristoylated alanine rich protein kinase C substrate |
| Matn2 | Inv4 | matrilin 2 |
| Mbd1 | Inv10 | methyl-CpG binding domain protein 1 |
| Mbnl1 | Inv1 | muscleblind-like 1 (Drosophila) |
| Mcl1 | Inv1 | myeloid cell leukemia sequence 1 |
| Mcoln2 | Inv5 | mucolipin 2 |
| Med27 | Inv3 | mediator complex subunit 27 |
| Mfap5 | Inv1 | microfibrillar associated protein 5 |
| Mfhas1 | Inv6 | malignant fibrous histiocytoma amplified sequence 1 |
| Mfsd1 | Inv2 | major facilitator superfamily domain containing 1 |
| Mfsd4 | Inv6 | major facilitator superfamily domain containing 4 |
| Mgll | Inv1 | monoglyceride lipase |
| Mgp | Inv1 | matrix Gla protein |
| Midn | Inv1 | midnolin |
| Minos1 | Inv4 | mitochondrial inner membrane organizing system 1 |
| Mlph | Inv7 | melanophilin |
| Mmp12 | Inv1 | matrix metallopeptidase 12 |
| Mmp3 | Inv1 | matrix metallopeptidase 3 |
| Mpeg1 | Inv1 | macrophage expressed gene 1 |
| Mpp1 | Inv4 | membrane protein, palmitoylated |
| Mrc1 | Inv1 | mannose receptor, C type 1 |
| Mrpl16 | Inv4 | mitochondrial ribosomal protein L16 |
| Mrpl24 | Inv8 | mitochondrial ribosomal protein L24 |
| Mrpl45 | Inv2 | mitochondrial ribosomal protein L45 |
| Mrpl48 | Inv1 | mitochondrial ribosomal protein L48 |
| Mrps12 | Inv8 | mitochondrial ribosomal protein S12 |
| Mrps21 | Inv8 | mitochondrial ribosomal protein S21 |
| Mt1 | Inv5 | metallothionein 1 |
| Mtpn | Inv4 | myotrophin |
| Myh11 | Inv3 | myosin, heavy polypeptide 11, smooth muscle |
| Myh9 | Inv1 | myosin, heavy polypeptide 9, non-muscle |
| Myl9 | Inv3 | myosin, light polypeptide 9, regulatory |
| Nampt | Inv7 | nicotinamide phosphoribosyltransferase |
| Nans | Inv3 | N-acetylneuraminic acid synthase (sialic acid synthase) |
| Ncl | Inv2 | nucleolin |
| Ndel1 | Inv1 | nuclear distribution gene E-like homolog 1 (A. nidulans) |
| Ndst2 | Inv6 | N-deacetylase/N-sulfotransferase (heparan glucosaminyl) 2 |
| Ndufb10 | Inv4 | NADH dehydrogenase (ubiquinone) 1 beta subcomplex, 10 |
| Ndufv1 | Inv4 | NADH dehydrogenase (ubiquinone) flavoprotein 1 |
| Ndufv3 | Inv6 | NADH dehydrogenase (ubiquinone) flavoprotein 3 |
| Nedd4l | Inv1 | neural precursor cell expressed, developmentally down-regulated gene 4-like |
| Nedd9 | Inv1 | neural precursor cell expressed, developmentally down-regulated gene 9 |
| Nfe2l3 | Inv10 | nuclear factor, erythroid derived 2, like 3 |
| Nfib | Inv8 | nuclear factor I/B |
| Nfix | Inv1 | nuclear factor I/X |
| Nfkb1 | Inv1 | nuclear factor of kappa light polypeptide gene enhancer in B cells 1, p105 |
| Nfkbie | Inv5 | nuclear factor of kappa light polypeptide gene enhancer in B cells inhibitor, epsilon |
| Nhp2 | Inv2 | NHP2 ribonucleoprotein |
| Nisch | Inv1 | nischarin |
| Nkiras1 | Inv5 | NFKB inhibitor interacting Ras-like protein 1 |
| Nkx2-3 | Inv5 | NK2 homeobox 3 |
| Nme3 | Inv8 | NME/NM23 nucleoside diphosphate kinase 3 |
| Nmt1 | Inv2 | N-myristoyltransferase 1 |
| Nnt | Inv7 | nicotinamide nucleotide transhydrogenase |
| Nop2 | Inv5 | NOP2 nucleolar protein |
| Notch1 | Inv1 | notch 1 |
| Nptn | Inv1 | neuroplastin |
| Npy | Inv5 | neuropeptide Y |
| Nr3c1 | Inv6 | nuclear receptor subfamily 3, group C, member 1 |
| Nrep | Inv6 | neuronal regeneration related protein |
| Nrp1 | Inv1 | neuropilin 1 |
| Ntpcr | Inv4 | nucleoside-triphosphatase, cancer-related |
| Nudcd2 | Inv8 | NudC domain containing 2 |
| Nudt4 | Inv7 | nudix (nucleoside diphosphate linked moiety X)-type motif 4 |
| Nudt9 | Inv1 | nudix (nucleoside diphosphate linked moiety X)-type motif 9 |
| Ogn | Inv1 | osteoglycin |
| Osbp | Inv3 | oxysterol binding protein |
| Osbpl5 | Inv5 | oxysterol binding protein-like 5 |
| Osmr | Inv1 | oncostatin M receptor |
| Ostf1 | Inv1 | osteoclast stimulating factor 1 |
| Oxct1 | Inv7 | 3-oxoacid CoA transferase 1 |
| P2rx4 | Inv2 | purinergic receptor P2X, ligand-gated ion channel 4 |
| Pak4 | Inv3 | p21 protein (Cdc42/Rac)-activated kinase 4 |
| Papss1 | Inv4 | 3'-phosphoadenosine 5'-phosphosulfate synthase 1 |
| Pax4 | Inv5 | paired box 4 |
| Paxbp1 | Inv1 | PAX3 and PAX7 binding protein 1 |
| Pck1 | Inv1 | phosphoenolpyruvate carboxykinase 1, cytosolic |
| Pcolce | Inv1 | procollagen C-endopeptidase enhancer protein |
| Pcx | Inv7 | pyruvate carboxylase |
| Pcyox1 | Inv8 | prenylcysteine oxidase 1 |
| Pdgfra | Inv1 | platelet derived growth factor receptor, alpha polypeptide |
| Pdha1 | Inv6 | pyruvate dehydrogenase E1 alpha 1 |
| Pdk1 | Inv3 | pyruvate dehydrogenase kinase, isoenzyme 1 |
| Pdk4 | Inv2 | pyruvate dehydrogenase kinase, isoenzyme 4 |
| Pdlim3 | Inv9 | PDZ and LIM domain 3 |
| Pds5b | Inv6 | PDS5, regulator of cohesion maintenance, homolog B (S. cerevisiae) |
| Pecam1 | Inv4 | platelet/endothelial cell adhesion molecule 1 |
| Pex19 | Inv4 | peroxisomal biogenesis factor 19 |
| Pfdn2 | Inv10 | prefoldin 2 |
| Pgrmc1 | Inv1 | progesterone receptor membrane component 1 |
| Phlda1 | Inv1 | pleckstrin homology-like domain, family A, member 1 |
| Phyh | Inv1 | phytanoyl-CoA hydroxylase |
| Pik3r1 | Inv1 | phosphatidylinositol 3-kinase, regulatory subunit, polypeptide 1 (p85 alpha) |
| Pip | Inv7 | prolactin induced protein |
| Pkd2 | Inv1 | polycystic kidney disease 2 |
| Pla2g12a | Inv3 | phospholipase A2, group XIIA |
| Pla2g7 | Inv1 | phospholipase A2, group VII (platelet-activating factor acetylhydrolase, plasma) |
| Plac8 | Inv1 | placenta-specific 8 |
| Plbd1 | Inv1 | phospholipase B domain containing 1 |
| Plin4 | Inv1 | perilipin 4 |
| Plp2 | Inv2 | proteolipid protein 2 |
| Pls3 | Inv2 | plastin 3 (T-isoform) |
| Plscr2 | Inv1 | phospholipid scramblase 2 |
| Plxnd1 | Inv1 | plexin D1 |
| Pmp22 | Inv1 | peripheral myelin protein 22 |
| Pold4 | Inv1 | polymerase (DNA-directed), delta 4 |
| Polr2g | Inv2 | polymerase (RNA) II (DNA directed) polypeptide G |
| Polr2m | Inv2 | polymerase (RNA) II (DNA directed) polypeptide M |
| Pomp | Inv2 | proteasome maturation protein |
| Postn | Inv8 | periostin, osteoblast specific factor |
| Ppa2 | Inv2 | pyrophosphatase (inorganic) 2 |
| Ppap2b | Inv4 | phosphatidic acid phosphatase type 2B |
| Pparg | Inv9 | peroxisome proliferator activated receptor gamma |
| Ppic | Inv1 | peptidylprolyl isomerase C |
| Ppp1r21 | Inv1 | protein phosphatase 1, regulatory subunit 21 |
| Ppp1r7 | Inv2 | protein phosphatase 1, regulatory (inhibitor) subunit 7 |
| Ppp2cb | Inv10 | protein phosphatase 2 (formerly 2A), catalytic subunit, beta isoform |
| Ppp2r5c | Inv2 | protein phosphatase 2, regulatory subunit B', gamma |
| Pqlc3 | Inv1 | PQ loop repeat containing |
| Prdx4 | Inv3 | peroxiredoxin 4 |
| Prdx5 | Inv8 | peroxiredoxin 5 |
| Prkab1 | Inv4 | protein kinase, AMP-activated, beta 1 non-catalytic subunit |
| Prkar1a | Inv1 | protein kinase, cAMP dependent regulatory, type I, alpha |
| Prkcdbp | Inv1 | protein kinase C, delta binding protein |
| Prkcsh | Inv2 | protein kinase C substrate 80K-H |
| Prlr | Inv6 | prolactin receptor |
| Prm3 | Inv5 | protamine 3 |
| Prnp | Inv1 | prion protein |
| Pros1 | Inv1 | protein S (alpha) |
| Psat1 | Inv7 | phosphoserine aminotransferase 1 |
| Psma1 | Inv2 | proteasome (prosome, macropain) subunit, alpha type 1 |
| Psma2 | Inv2 | proteasome (prosome, macropain) subunit, alpha type 2 |
| Psma6 | Inv2 | proteasome (prosome, macropain) subunit, alpha type 6 |
| Psmb1 | Inv8 | proteasome (prosome, macropain) subunit, beta type 1 |
| Psmb4 | Inv2 | proteasome (prosome, macropain) subunit, beta type 4 |
| Psmb8 | Inv1 | proteasome (prosome, macropain) subunit, beta type 8  (large multifunctional peptidase 7) |
| Psmc2 | Inv2 | proteasome (prosome, macropain) 26S subunit, ATPase 2 |
| Psmc3 | Inv2 | proteasome (prosome, macropain) 26S subunit, ATPase 3 |
| Psmd13 | Inv2 | proteasome (prosome, macropain) 26S subunit, non-ATPase, 13 |
| Psmd14 | Inv2 | proteasome (prosome, macropain) 26S subunit, non-ATPase, 14 |
| Psmd3 | Inv2 | proteasome (prosome, macropain) 26S subunit, non-ATPase, 3 |
| Psmd4 | Inv2 | proteasome (prosome, macropain) 26S subunit, non-ATPase, 4 |
| Psmd6 | Inv2 | proteasome (prosome, macropain) 26S subunit, non-ATPase, 6 |
| Psmd7 | Inv2 | proteasome (prosome, macropain) 26S subunit, non-ATPase, 7 |
| Psme1 | Inv1 | proteasome (prosome, macropain) activator subunit 1 (PA28 alpha) |
| Ptgs1 | Inv6 | prostaglandin-endoperoxide synthase 1 |
| Pthlh | Inv7 | parathyroid hormone-like peptide |
| Ptplad1 | Inv8 | protein tyrosine phosphatase-like A domain containing 1 |
| Qk | Inv1 | quaking |
| Qsox1 | Inv3 | quiescin Q6 sulfhydryl oxidase 1 |
| Rab1 | Inv3 | RAB1, member RAS oncogene family |
| Rab18 | Inv6 | RAB18, member RAS oncogene family |
| Rab34 | Inv1 | RAB34, member of RAS oncogene family |
| Rab4a | Inv7 | RAB4A, member RAS oncogene family |
| Rabggtb | Inv2 | RAB geranylgeranyl transferase, b subunit |
| Rad21 | Inv1 | RAD21 homolog (S. pombe) |
| Ramp1 | Inv4 | receptor (calcitonin) activity modifying protein 1 |
| Rap1gds1 | Inv4 | RAP1, GTP-GDP dissociation stimulator 1 |
| Rasa3 | Inv1 | RAS p21 protein activator 3 |
| Rasa4 | Inv9 | RAS p21 protein activator 4 |
| Rbbp4 | Inv2 | retinoblastoma binding protein 4 |
| Rbp1 | Inv1 | retinol binding protein 1, cellular |
| Rbp4 | Inv9 | retinol binding protein 4, plasma |
| Rcn1 | Inv1 | reticulocalbin 1 |
| Reck | Inv1 | reversion-inducing-cysteine-rich protein with kazal motifs |
| Reep5 | Inv4 | receptor accessory protein 5 |
| Rell1 | Inv3 | RELT-like 1 |
| Ret | Inv5 | ret proto-oncogene |
| Retn | Inv9 | resistin |
| Rfk | Inv6 | riboflavin kinase |
| Rgcc | Inv9 | regulator of cell cycle |
| Rgs10 | Inv9 | regulator of G-protein signalling 10 |
| Rgs2 | Inv1 | regulator of G-protein signaling 2 |
| Rin2 | Inv1 | Ras and Rab interactor 2 |
| Rnase4 | Inv1 | ribonuclease, RNase A family 4 |
| Rnf145 | Inv7 | ring finger protein 145 |
| Rnf181 | Inv4 | ring finger protein 181 |
| Rnf4 | Inv2 | ring finger protein 4 |
| Rnf5 | Inv3 | ring finger protein 5 |
| Rnpepl1 | Inv2 | arginyl aminopeptidase (aminopeptidase B)-like 1 |
| Rogdi | Inv6 | rogdi homolog (Drosophila) |
| Rora | Inv6 | RAR-related orphan receptor alpha |
| Rps6kb2 | Inv5 | ribosomal protein S6 kinase, polypeptide 2 |
| Rragc | Inv2 | Ras-related GTP binding C |
| Rras | Inv10 | Harvey rat sarcoma oncogene, subgroup R |
| Rsad2 | Inv2 | radical S-adenosyl methionine domain containing 2 |
| Rsl1d1 | Inv2 | ribosomal L1 domain containing 1 |
| Rtn4 | Inv10 | reticulon 4 |
| S100a10 | Inv2 | S100 calcium binding protein A10 (calpactin) |
| S100a6 | Inv2 | S100 calcium binding protein A6 (calcyclin) |
| S100a8 | Inv8 | S100 calcium binding protein A8 (calgranulin A) |
| Samhd1 | Inv1 | SAM domain and HD domain, 1 |
| Samm50 | Inv7 | sorting and assembly machinery component 50 homolog (S. cerevisiae) |
| Scarb1 | Inv4 | scavenger receptor class B, member 1 |
| Scarb2 | Inv2 | scavenger receptor class B, member 2 |
| Scd2 | Inv6 | stearoyl-Coenzyme A desaturase 2 |
| Sdhc | Inv4 | succinate dehydrogenase complex, subunit C, integral membrane protein |
| Sec14l1 | Inv6 | SEC14-like 1 (S. cerevisiae) |
| Sec31a | Inv4 | Sec31 homolog A (S. cerevisiae) |
| Sec61a1 | Inv4 | Sec61 alpha 1 subunit (S. cerevisiae) |
| Seh1l | Inv4 | SEH1-like (S. cerevisiae |
| Selk | Inv8 | selenoprotein K |
| Sema7a | Inv6 | sema domain, immunoglobulin domain (Ig), and GPI membrane anchor,  (semaphorin) 7A |
| Serp1 | Inv3 | stress-associated endoplasmic reticulum protein 1 |
| Serpinb6a | Inv1 | serine (or cysteine) peptidase inhibitor, clade B, member 6a |
| Serpinf1 | Inv1 | serine (or cysteine) peptidase inhibitor, clade F, member 1 |
| Serping1 | Inv1 | serine (or cysteine) peptidase inhibitor, clade G, member 1 |
| Sesn1 | Inv9 | sestrin 1 |
| Sgce | Inv9 | sarcoglycan, epsilon |
| Sh3bgrl | Inv1 | SH3-binding domain glutamic acid-rich protein like |
| Sh3bp5 | Inv6 | SH3-domain binding protein 5 (BTK-associated) |
| Shisa5 | Inv1 | shisa homolog 5 (Xenopus laevis) |
| Sidt2 | Inv3 | SID1 transmembrane family, member 2 |
| Sin3b | Inv2 | transcriptional regulator, SIN3B (yeast) |
| Skap2 | Inv1 | src family associated phosphoprotein 2 |
| Slc12a2 | Inv10 | solute carrier family 12, member 2 |
| Slc16a2 | Inv2 | solute carrier family 16 (monocarboxylic acid transporters), member 2 |
| Slc1a4 | Inv6 | solute carrier family 1 (glutamate/neutral amino acid transporter), member 4 |
| Slc1a5 | Inv9 | solute carrier family 1 (neutral amino acid transporter), member 5 |
| Slc22a1 | Inv3 | solute carrier family 22 (organic cation transporter), member 1 |
| Slc25a1 | Inv7 | solute carrier family 25 (mitochondrial carrier, citrate transporter), member 1 |
| Slc25a17 | Inv6 | solute carrier family 25 (mitochondrial carrier, peroxisomal membrane protein),  member 17 |
| Slc25a20 | Inv1 | solute carrier family 25 (mitochondrial carnitine/acylcarnitine translocase),  member 20 |
| Slc25a4 | Inv1 | solute carrier family 25 (mitochondrial carrier, adenine nucleotide translocator),  member 4 |
| Slc27a3 | Inv3 | solute carrier family 27 (fatty acid transporter), member 3 |
| Slc2a1 | Inv7 | solute carrier family 2 (facilitated glucose transporter), member 1 |
| Slc30a4 | Inv3 | solute carrier family 30 (zinc transporter), member 4 |
| Slc34a2 | Inv7 | solute carrier family 34 (sodium phosphate), member 2 |
| Slc35b1 | Inv6 | solute carrier family 35, member B1 |
| Slc35b2 | Inv3 | solute carrier family 35, member B2 |
| Slc39a6 | Inv3 | solute carrier family 39 (metal ion transporter), member 6 |
| Slc41a1 | Inv6 | solute carrier family 41, member 1 |
| Slc50a1 | Inv7 | solute carrier family 50 (sugar transporter), member 1 |
| Slc5a1 | Inv6 | solute carrier family 5 (sodium/glucose cotransporter), member 1 |
| Slc5a6 | Inv7 | solute carrier family 5 (sodium-dependent vitamin transporter), member 6 |
| Slc6a6 | Inv1 | solute carrier family 6 (neurotransmitter transporter, taurine), member 6 |
| Slc7a7 | Inv7 | solute carrier family 7 (cationic amino acid transporter, y+ system), member 7 |
| Slc9a3r1 | Inv3 | solute carrier family 9 (sodium/hydrogen exchanger), member 3 regulator 1 |
| Slfn2 | Inv1 | schlafen 2 |
| Slirp | Inv2 | SRA stem-loop interacting RNA binding protein |
| Slpi | Inv10 | secretory leukocyte peptidase inhibitor |
| Sltm | Inv1 | SAFB-like, transcription modulator |
| Smarca4 | Inv2 | SWI/SNF related, matrix associated, actin dependent regulator of chromatin,  subfamily a, member 4 |
| Smpd1 | Inv7 | sphingomyelin phosphodiesterase 1, acid lysosomal |
| Snapin | Inv10 | SNAP-associated protein |
| Snx6 | Inv1 | sorting nexin 6 |
| Socs3 | Inv1 | suppressor of cytokine signaling 3 |
| Sorbs1 | Inv9 | sorbin and SH3 domain containing 1 |
| Sowahc | Inv9 | sosondowah ankyrin repeat domain family member C |
| Sox4 | Inv10 | SRY (sex determining region Y)-box 4 |
| Spast | Inv6 | spastin |
| Spata5 | Inv3 | spermatogenesis associated 5 |
| Spint1 | Inv2 | serine protease inhibitor, Kunitz type 1 |
| Sptan1 | Inv1 | spectrin alpha, non-erythrocytic 1 |
| Sptbn1 | Inv1 | spectrin beta, non-erythrocytic 1 |
| Sqle | Inv6 | squalene epoxidase |
| Sra1 | Inv4 | steroid receptor RNA activator 1 |
| Srm | Inv3 | spermidine synthase |
| Srms | Inv5 | src-related kinase lacking C-terminal regulatory tyrosine and  N-terminal myristylation sites |
| Srp14 | Inv2 | signal recognition particle 14 |
| Srrm1 | Inv8 | serine/arginine repetitive matrix 1 |
| Srrt | Inv8 | serrate RNA effector molecule homolog (Arabidopsis) |
| Srsf11 | Inv8 | serine/arginine-rich splicing factor 11 |
| Ssr3 | Inv6 | signal sequence receptor, gamma |
| Ssrp1 | Inv2 | structure specific recognition protein 1 |
| St3gal4 | Inv3 | ST3 beta-galactoside alpha-2,3-sialyltransferase 4 |
| St6gal1 | Inv3 | beta galactoside alpha 2,6 sialyltransferase 1 |
| Stat3 | Inv1 | signal transducer and activator of transcription 3 |
| Stat5b | Inv6 | signal transducer and activator of transcription 5B |
| Stx3 | Inv5 | syntaxin 3 |
| Stx4a | Inv1 | syntaxin 4A (placental) |
| Supt5 | Inv1 | suppressor of Ty 5 |
| Swi5 | Inv4 | SWI5 recombination repair homolog (yeast) |
| Synpo | Inv2 | synaptopodin |
| Tagln2 | Inv2 | transgelin 2 |
| Taldo1 | Inv2 | transaldolase 1 |
| Tap1 | Inv1 | transporter 1, ATP-binding cassette, sub-family B (MDR/TAP) |
| Tbc1d14 | Inv6 | TBC1 domain family, member 14 |
| Tbca | Inv8 | tubulin cofactor A |
| Tbxa2r | Inv6 | thromboxane A2 receptor |
| Tcerg1 | Inv1 | transcription elongation regulator 1 (CA150) |
| Tead2 | Inv10 | TEA domain family member 2 |
| Tfrc | Inv6 | transferrin receptor |
| Tg | Inv5 | thyroglobulin |
| Tgfb3 | Inv1 | transforming growth factor, beta 3 |
| Tgfbi | Inv1 | transforming growth factor, beta induced |
| Thbs2 | Inv1 | thrombospondin 2 |
| Thumpd1 | Inv10 | THUMP domain containing 1 |
| Timm17a | Inv8 | translocase of inner mitochondrial membrane 17a |
| Tinagl1 | Inv7 | tubulointerstitial nephritis antigen-like 1 |
| Tipin | Inv5 | timeless interacting protein |
| Tm7sf2 | Inv6 | transmembrane 7 superfamily member 2 |
| Tmc6 | Inv3 | transmembrane channel-like gene family 6 |
| Tmem14c | Inv2 | transmembrane protein 14C |
| Tmem176a | Inv4 | transmembrane protein 176A |
| Tmem176b | Inv4 | transmembrane protein 176B |
| Tmem230 | Inv1 | transmembrane protein 230 |
| Tmem43 | Inv10 | transmembrane protein 43 |
| Tmem55a | Inv2 | transmembrane protein 55A |
| Tmem66 | Inv4 | transmembrane protein 66 |
| Tmsb4x | Inv1 | thymosin, beta 4, X chromosome |
| Tnfrsf11a | Inv6 | tumor necrosis factor receptor superfamily, member 11a |
| Tnfrsf1b | Inv9 | tumor necrosis factor receptor superfamily, member 1b |
| Tob1 | Inv7 | transducer of ErbB-2.1 |
| Tor1b | Inv6 | torsin family 1, member B |
| Tor3a | Inv7 | torsin family 3, member A |
| Tpd52l1 | Inv6 | tumor protein D52-like 1 |
| Traf1 | Inv3 | TNF receptor-associated factor 1 |
| Trafd1 | Inv1 | TRAF type zinc finger domain containing 1 |
| Tram1 | Inv6 | translocating chain-associating membrane protein 1 |
| Trib3 | Inv3 | tribbles homolog 3 (Drosophila) |
| Trim11 | Inv8 | tripartite motif-containing 11 |
| Trim28 | Inv2 | tripartite motif-containing 28 |
| Tsc22d1 | Inv1 | TSC22 domain family, member 1 |
| Tspan4 | Inv1 | tetraspanin 4 |
| Tspan7 | Inv7 | tetraspanin 7 |
| Tspo | Inv1 | translocator protein |
| Ttc7b | Inv3 | tetratricopeptide repeat domain 7B |
| Txndc12 | Inv10 | thioredoxin domain containing 12 (endoplasmic reticulum) |
| Txndc16 | Inv4 | thioredoxin domain containing 16 |
| Txnip | Inv1 | thioredoxin interacting protein |
| Tyro3 | Inv10 | TYRO3 protein tyrosine kinase 3 |
| Tyrobp | Inv1 | TYRO protein tyrosine kinase binding protein |
| Uba2 | Inv2 | ubiquitin-like modifier activating enzyme 2 |
| Uba5 | Inv3 | ubiquitin-like modifier activating enzyme 5 |
| Ube2r2 | Inv2 | ubiquitin-conjugating enzyme E2R 2 |
| Ubxn4 | Inv2 | UBX domain protein 4 |
| Ubxn8 | Inv2 | UBX domain protein 8 |
| Ugcg | Inv7 | UDP-glucose ceramide glucosyltransferase |
| Use1 | Inv1 | unconventional SNARE in the ER 1 homolog (S. cerevisiae) |
| Usp22 | Inv3 | ubiquitin specific peptidase 22 |
| Usp24 | Inv3 | ubiquitin specific peptidase 24 |
| Vcam1 | Inv9 | vascular cell adhesion molecule 1 |
| Vps37b | Inv3 | vacuolar protein sorting 37B (yeast) |
| Wbp1l | Inv1 | WW domain binding protein 1 like |
| Wdr1 | Inv2 | WD repeat domain 1 |
| Wdr77 | Inv2 | WD repeat domain 77 |
| Wfdc2 | Inv1 | WAP four-disulfide core domain 2 |
| Wnt5b | Inv1 | wingless-related MMTV integration site 5B |
| Wt1 | Inv5 | Wilms tumor 1 homolog |
| Xdh | Inv7 | xanthine dehydrogenase |
| Xpo7 | Inv2 | exportin 7 |
| Ybx2 | Inv5 | Y box protein 2 |
| Yipf1 | Inv2 | Yip1 domain family, member 1 |
| Ypel3 | Inv1 | yippee-like 3 (Drosophila) |
| Ywhaq | Inv2 | tyrosine 3-monooxygenase/tryptophan 5-monooxygenase activation protein,  theta polypeptide |
| Zfp36l1 | Inv1 | zinc finger protein 36, C3H type-like 1 |
| Zfp422 | Inv5 | zinc finger protein 422 |
| Zranb2 | Inv4 | zinc finger, RAN-binding domain containing 2 |

| List of orthologous genes identified in Stein et al. [4]. | | |
| --- | --- | --- |
| SYMBOL | Gene Signature | GENENAME |
| 0610010B08Rik | S.c8 | RIKEN cDNA 0610010B08 gene |
| 0610010B08Rik | S.i1vl7 | RIKEN cDNA 0610010B08 gene |
| 0610010B08Rik | S.i4vl7 | RIKEN cDNA 0610010B08 gene |
| 0610010B08Rik | S.i2vl7 | RIKEN cDNA 0610010B08 gene |
| 0610010B08Rik | S.i3vl7 | RIKEN cDNA 0610010B08 gene |
| 0610010K14Rik | S.i3vl7 | RIKEN cDNA 0610010K14 gene |
| 0610010K14Rik | S.i1vl7 | RIKEN cDNA 0610010K14 gene |
| 0610010K14Rik | S.i4vl7 | RIKEN cDNA 0610010K14 gene |
| 0610010K14Rik | S.i2vl7 | RIKEN cDNA 0610010K14 gene |
| 0610010K14Rik | S.c3 | RIKEN cDNA 0610010K14 gene |
| 0610010K14Rik | S.c4 | RIKEN cDNA 0610010K14 gene |
| 1110001J03Rik | S.c7 | RIKEN cDNA 1110001J03 gene |
| 1110001J03Rik | S.i1vl7 | RIKEN cDNA 1110001J03 gene |
| 1110004F10Rik | S.i1vl7 | RIKEN cDNA 1110004F10 gene |
| 1110004F10Rik | S.c4 | RIKEN cDNA 1110004F10 gene |
| 1110007C09Rik | S.i2vl7 | RIKEN cDNA 1110007C09 gene |
| 1110008P14Rik | S.i4vl7 | RIKEN cDNA 1110008P14 gene |
| 1110032A03Rik | S.c8 | RIKEN cDNA 1110032A03 gene |
| 1190002N15Rik | S.c1 | RIKEN cDNA 1190002N15 gene |
| 1190002N15Rik | S.i4vl7 | RIKEN cDNA 1190002N15 gene |
| 1190005I06Rik | S.c2 | RIKEN cDNA 1190005I06 gene |
| 1190005I06Rik | S.i4vl7 | RIKEN cDNA 1190005I06 gene |
| 1190005I06Rik | S.i3vl7 | RIKEN cDNA 1190005I06 gene |
| 1700017B05Rik | S.c2 | RIKEN cDNA 1700017B05 gene |
| 1700037H04Rik | S.c2 | RIKEN cDNA 1700037H04 gene |
| 1700037H04Rik | S.i3vl7 | RIKEN cDNA 1700037H04 gene |
| 1700071K01Rik | S.i1vl7 | RIKEN cDNA 1700071K01 gene |
| 1700071K01Rik | S.c7 | RIKEN cDNA 1700071K01 gene |
| 1810030O07Rik | S.i4vl7 | RIKEN cDNA 1810030O07 gene |
| 1810030O07Rik | S.i2vl7 | RIKEN cDNA 1810030O07 gene |
| 1810030O07Rik | S.c4 | RIKEN cDNA 1810030O07 gene |
| 1810030O07Rik | S.i3vl7 | RIKEN cDNA 1810030O07 gene |
| 2510002D24Rik | S.i4vl7 | RIKEN cDNA 2510002D24 gene |
| 2510002D24Rik | S.c9 | RIKEN cDNA 2510002D24 gene |
| 2610002J02Rik | S.c4 | RIKEN cDNA 2610002J02 gene |
| 2610002J02Rik | S.i2vl7 | RIKEN cDNA 2610002J02 gene |
| 2610002J02Rik | S.i4vl7 | RIKEN cDNA 2610002J02 gene |
| 2610002J02Rik | S.i1vl7 | RIKEN cDNA 2610002J02 gene |
| 2610002J02Rik | S.i3vl7 | RIKEN cDNA 2610002J02 gene |
| 2700094K13Rik | S.i4vl7 | RIKEN cDNA 2700094K13 gene |
| 2700094K13Rik | S.i1vl7 | RIKEN cDNA 2700094K13 gene |
| 2700094K13Rik | S.c6 | RIKEN cDNA 2700094K13 gene |
| 3110043O21Rik | S.c3 | RIKEN cDNA 3110043O21 gene |
| 4833420G17Rik | S.c4 | RIKEN cDNA 4833420G17 gene |
| 4931406C07Rik | S.i4vl7 | RIKEN cDNA 4931406C07 gene |
| 4931406C07Rik | S.c3 | RIKEN cDNA 4931406C07 gene |
| 4933426M11Rik | S.i2vl7 | RIKEN cDNA 4933426M11 gene |
| 4933426M11Rik | S.i1vl7 | RIKEN cDNA 4933426M11 gene |
| 4933426M11Rik | c5.i3vl7 | RIKEN cDNA 4933426M11 gene |
| 4933426M11Rik | S.i3vl7 | RIKEN cDNA 4933426M11 gene |
| 4933426M11Rik | S.i4vl7 | RIKEN cDNA 4933426M11 gene |
| 9530053A07Rik | S.c1 | RIKEN cDNA 9530053A07 gene |
| 9530053A07Rik | S.i4vl7 | RIKEN cDNA 9530053A07 gene |
| 9530068E07Rik | S.i4vl7 | RIKEN cDNA 9530068E07 gene |
| 9530068E07Rik | S.c4 | RIKEN cDNA 9530068E07 gene |
| 9530068E07Rik | S.i2vl7 | RIKEN cDNA 9530068E07 gene |
| 9530068E07Rik | S.i1vl7 | RIKEN cDNA 9530068E07 gene |
| 9530068E07Rik | S.i3vl7 | RIKEN cDNA 9530068E07 gene |
| A4galt | S.i4vl7 | alpha 1,4-galactosyltransferase |
| A4galt | S.c8 | alpha 1,4-galactosyltransferase |
| A4galt | S.i2vl7 | alpha 1,4-galactosyltransferase |
| Aass | S.i1vl7 | aminoadipate-semialdehyde synthase |
| Aass | S.i4vl7 | aminoadipate-semialdehyde synthase |
| Aass | S.i3vl7 | aminoadipate-semialdehyde synthase |
| Aass | S.c8 | aminoadipate-semialdehyde synthase |
| Aass | S.i2vl7 | aminoadipate-semialdehyde synthase |
| Abca1 | S.c2 | ATP-binding cassette, sub-family A (ABC1), member 1 |
| Abca1 | S.i4vl7 | ATP-binding cassette, sub-family A (ABC1), member 1 |
| Abca1 | S.i3vl7 | ATP-binding cassette, sub-family A (ABC1), member 1 |
| Abca1 | S.i2vl7 | ATP-binding cassette, sub-family A (ABC1), member 1 |
| Abcb9 | S.c7 | ATP-binding cassette, sub-family B (MDR/TAP), member 9 |
| Abcd2 | S.i3vl7 | ATP-binding cassette, sub-family D (ALD), member 2 |
| Abcd3 | S.c9 | ATP-binding cassette, sub-family D (ALD), member 3 |
| Abce1 | S.i2vl7 | ATP-binding cassette, sub-family E (OABP), member 1 |
| Abce1 | S.i4vl7 | ATP-binding cassette, sub-family E (OABP), member 1 |
| Abce1 | S.i3vl7 | ATP-binding cassette, sub-family E (OABP), member 1 |
| Abce1 | S.c8 | ATP-binding cassette, sub-family E (OABP), member 1 |
| Abcg1 | S.c1 | ATP-binding cassette, sub-family G (WHITE), member 1 |
| Abcg1 | S.i4vl7 | ATP-binding cassette, sub-family G (WHITE), member 1 |
| Abcg2 | S.c8 | ATP-binding cassette, sub-family G (WHITE), member 2 |
| Abcg2 | S.i1vl7 | ATP-binding cassette, sub-family G (WHITE), member 2 |
| Abcg2 | S.i2vl7 | ATP-binding cassette, sub-family G (WHITE), member 2 |
| Abcg2 | S.i4vl7 | ATP-binding cassette, sub-family G (WHITE), member 2 |
| Abhd14a | S.i3vl7 | abhydrolase domain containing 14A |
| Abhd14a | S.i2vl7 | abhydrolase domain containing 14A |
| Abhd14a | S.c8 | abhydrolase domain containing 14A |
| Abhd14a | S.i4vl7 | abhydrolase domain containing 14A |
| Abhd8 | S.i3vl7 | abhydrolase domain containing 8 |
| Abhd8 | S.i2vl7 | abhydrolase domain containing 8 |
| Abhd8 | S.i1vl7 | abhydrolase domain containing 8 |
| Abhd8 | S.c7 | abhydrolase domain containing 8 |
| Abl1 | S.i4vl7 | c-abl oncogene 1, non-receptor tyrosine kinase |
| Abl1 | S.c3 | c-abl oncogene 1, non-receptor tyrosine kinase |
| Ablim1 | S.c2 | actin-binding LIM protein 1 |
| Ablim1 | S.i4vl7 | actin-binding LIM protein 1 |
| Acaa1a | S.c3 | acetyl-Coenzyme A acyltransferase 1A |
| Acaa1a | S.c4 | acetyl-Coenzyme A acyltransferase 1A |
| Acaa1a | S.i2vl7 | acetyl-Coenzyme A acyltransferase 1A |
| Acaa1a | S.i3vl7 | acetyl-Coenzyme A acyltransferase 1A |
| Acaa1a | S.i4vl7 | acetyl-Coenzyme A acyltransferase 1A |
| Acaa1b | S.i2vl7 | acetyl-Coenzyme A acyltransferase 1B |
| Acaa1b | S.i3vl7 | acetyl-Coenzyme A acyltransferase 1B |
| Acaa1b | S.c3 | acetyl-Coenzyme A acyltransferase 1B |
| Acaa1b | S.i4vl7 | acetyl-Coenzyme A acyltransferase 1B |
| Acaa2 | S.c4 | acetyl-Coenzyme A acyltransferase 2 (mitochondrial 3-oxoacyl-Coenzyme A thiolase) |
| Acaa2 | S.i4vl7 | acetyl-Coenzyme A acyltransferase 2 (mitochondrial 3-oxoacyl-Coenzyme A thiolase) |
| Acadl | S.i4vl7 | acyl-Coenzyme A dehydrogenase, long-chain |
| Acadl | c5.i3vl7 | acyl-Coenzyme A dehydrogenase, long-chain |
| Acadl | S.i1vl7 | acyl-Coenzyme A dehydrogenase, long-chain |
| Acadl | S.i3vl7 | acyl-Coenzyme A dehydrogenase, long-chain |
| Acadl | S.i2vl7 | acyl-Coenzyme A dehydrogenase, long-chain |
| Acadm | S.i3vl7 | acyl-Coenzyme A dehydrogenase, medium chain |
| Acadm | S.i4vl7 | acyl-Coenzyme A dehydrogenase, medium chain |
| Acadm | S.i2vl7 | acyl-Coenzyme A dehydrogenase, medium chain |
| Acadm | S.c3 | acyl-Coenzyme A dehydrogenase, medium chain |
| Acads | S.c2 | acyl-Coenzyme A dehydrogenase, short chain |
| Acat2 | S.c7 | acetyl-Coenzyme A acetyltransferase 2 |
| Acat2 | S.i1vl7 | acetyl-Coenzyme A acetyltransferase 2 |
| Ackr3 | S.c4 | atypical chemokine receptor 3 |
| Acly | S.c7 | ATP citrate lyase |
| Acly | S.i1vl7 | ATP citrate lyase |
| Acot10 | S.c6 | acyl-CoA thioesterase 10 |
| Acot10 | S.i1vl7 | acyl-CoA thioesterase 10 |
| Acot7 | S.c1 | acyl-CoA thioesterase 7 |
| Acot9 | S.c6 | acyl-CoA thioesterase 9 |
| Acot9 | S.i1vl7 | acyl-CoA thioesterase 9 |
| Acox1 | S.i3vl7 | acyl-Coenzyme A oxidase 1, palmitoyl |
| Acox1 | c5.i3vl7 | acyl-Coenzyme A oxidase 1, palmitoyl |
| Acox1 | S.i4vl7 | acyl-Coenzyme A oxidase 1, palmitoyl |
| Acox1 | S.i1vl7 | acyl-Coenzyme A oxidase 1, palmitoyl |
| Acox1 | S.i2vl7 | acyl-Coenzyme A oxidase 1, palmitoyl |
| Acp2 | S.c1 | acid phosphatase 2, lysosomal |
| Acp5 | S.i4vl7 | acid phosphatase 5, tartrate resistant |
| Acp5 | S.c2 | acid phosphatase 5, tartrate resistant |
| Acsf3 | S.c4 | acyl-CoA synthetase family member 3 |
| Acsl1 | S.c7 | acyl-CoA synthetase long-chain family member 1 |
| Acsl1 | S.i2vl7 | acyl-CoA synthetase long-chain family member 1 |
| Acsl1 | S.i1vl7 | acyl-CoA synthetase long-chain family member 1 |
| Acsl4 | c5.i3vl7 | acyl-CoA synthetase long-chain family member 4 |
| Acsl4 | S.i3vl7 | acyl-CoA synthetase long-chain family member 4 |
| Acsl4 | S.i2vl7 | acyl-CoA synthetase long-chain family member 4 |
| Acss1 | S.i3vl7 | acyl-CoA synthetase short-chain family member 1 |
| Acss1 | S.i4vl7 | acyl-CoA synthetase short-chain family member 1 |
| Acss1 | S.c8 | acyl-CoA synthetase short-chain family member 1 |
| Acss1 | S.i2vl7 | acyl-CoA synthetase short-chain family member 1 |
| Acta1 | S.c1 | actin, alpha 1, skeletal muscle |
| Actb | S.i3vl7 | actin, beta |
| Actb | S.c9 | actin, beta |
| Actg1 | S.c4 | actin, gamma, cytoplasmic 1 |
| Actn1 | S.i1vl7 | actinin, alpha 1 |
| Actn1 | S.i3vl7 | actinin, alpha 1 |
| Actn1 | S.c1 | actinin, alpha 1 |
| Actn1 | S.i4vl7 | actinin, alpha 1 |
| Actn1 | S.c4 | actinin, alpha 1 |
| Actn1 | S.i2vl7 | actinin, alpha 1 |
| Actn4 | S.i1vl7 | actinin alpha 4 |
| Actn4 | S.i3vl7 | actinin alpha 4 |
| Actn4 | c5.i3vl7 | actinin alpha 4 |
| Actn4 | S.i2vl7 | actinin alpha 4 |
| Actn4 | S.i4vl7 | actinin alpha 4 |
| Actr2 | S.c6 | ARP2 actin-related protein 2 |
| Actr3 | S.c3 | ARP3 actin-related protein 3 |
| Actr3 | S.i4vl7 | ARP3 actin-related protein 3 |
| Acyp2 | S.c2 | acylphosphatase 2, muscle type |
| Acyp2 | S.i4vl7 | acylphosphatase 2, muscle type |
| Adam10 | S.c6 | a disintegrin and metallopeptidase domain 10 |
| Adam10 | S.c4 | a disintegrin and metallopeptidase domain 10 |
| Adam10 | S.i4vl7 | a disintegrin and metallopeptidase domain 10 |
| Adam28 | S.i4vl7 | a disintegrin and metallopeptidase domain 28 |
| Adam28 | S.i2vl7 | a disintegrin and metallopeptidase domain 28 |
| Adam28 | S.c8 | a disintegrin and metallopeptidase domain 28 |
| Adam8 | S.c1 | a disintegrin and metallopeptidase domain 8 |
| Adam9 | S.c4 | a disintegrin and metallopeptidase domain 9 (meltrin gamma) |
| Adam9 | S.i3vl7 | a disintegrin and metallopeptidase domain 9 (meltrin gamma) |
| Adam9 | S.i1vl7 | a disintegrin and metallopeptidase domain 9 (meltrin gamma) |
| Adam9 | S.i2vl7 | a disintegrin and metallopeptidase domain 9 (meltrin gamma) |
| Adamdec1 | S.c1 | ADAM-like, decysin 1 |
| Adck1 | S.i2vl7 | aarF domain containing kinase 1 |
| Adck1 | S.c2 | aarF domain containing kinase 1 |
| Adck1 | S.i3vl7 | aarF domain containing kinase 1 |
| Adck1 | S.i4vl7 | aarF domain containing kinase 1 |
| Adcy6 | S.c3 | adenylate cyclase 6 |
| Adcy7 | S.c1 | adenylate cyclase 7 |
| Adcy7 | S.i4vl7 | adenylate cyclase 7 |
| Add1 | S.i3vl7 | adducin 1 (alpha) |
| Add1 | S.i1vl7 | adducin 1 (alpha) |
| Add1 | S.c3 | adducin 1 (alpha) |
| Add1 | S.i4vl7 | adducin 1 (alpha) |
| Adig | S.c1 | adipogenin |
| Adipoq | S.c2 | adiponectin, C1Q and collagen domain containing |
| Adipoq | S.i4vl7 | adiponectin, C1Q and collagen domain containing |
| Adipoq | S.i2vl7 | adiponectin, C1Q and collagen domain containing |
| Adk | S.i3vl7 | adenosine kinase |
| Adk | S.c7 | adenosine kinase |
| Adprh | S.i4vl7 | ADP-ribosylarginine hydrolase |
| Adprh | S.c3 | ADP-ribosylarginine hydrolase |
| Adprm | S.i2vl7 | ADP-ribose/CDP-alcohol diphosphatase, manganese dependent |
| Adrb3 | S.i2vl7 | adrenergic receptor, beta 3 |
| Adrb3 | S.c2 | adrenergic receptor, beta 3 |
| Adrb3 | S.c1 | adrenergic receptor, beta 3 |
| Adrb3 | S.i4vl7 | adrenergic receptor, beta 3 |
| Adss | S.c4 | adenylosuccinate synthetase, non muscle |
| Adssl1 | S.i4vl7 | adenylosuccinate synthetase like 1 |
| Adssl1 | S.c1 | adenylosuccinate synthetase like 1 |
| Aebp1 | S.c1 | AE binding protein 1 |
| AF251705 | S.c1 | cDNA sequence AF251705 |
| Agpat3 | S.i2vl7 | 1-acylglycerol-3-phosphate O-acyltransferase 3 |
| Agpat3 | S.i4vl7 | 1-acylglycerol-3-phosphate O-acyltransferase 3 |
| Agr2 | S.i4vl7 | anterior gradient 2 |
| Agr2 | S.c8 | anterior gradient 2 |
| Agrn | S.i2vl7 | agrin |
| Agrn | S.i1vl7 | agrin |
| Agrn | S.c4 | agrin |
| Agrn | S.i3vl7 | agrin |
| Agrn | S.i4vl7 | agrin |
| Ahnak | S.i4vl7 | AHNAK nucleoprotein (desmoyokin) |
| Ahnak | S.i2vl7 | AHNAK nucleoprotein (desmoyokin) |
| Ahnak | S.c4 | AHNAK nucleoprotein (desmoyokin) |
| Ahnak | S.i3vl7 | AHNAK nucleoprotein (desmoyokin) |
| Ahnak | S.i1vl7 | AHNAK nucleoprotein (desmoyokin) |
| Ahr | S.c4 | aryl-hydrocarbon receptor |
| Aif1 | S.c1 | allograft inflammatory factor 1 |
| Aif1 | S.i4vl7 | allograft inflammatory factor 1 |
| Ak1 | S.c1 | adenylate kinase 1 |
| Ak2 | S.i1vl7 | adenylate kinase 2 |
| Ak2 | S.c7 | adenylate kinase 2 |
| Ak3 | S.c8 | adenylate kinase 3 |
| Akirin2 | S.c4 | akirin 2 |
| Akr1a1 | S.c4 | aldo-keto reductase family 1, member A1 (aldehyde reductase) |
| Akr1a1 | S.i2vl7 | aldo-keto reductase family 1, member A1 (aldehyde reductase) |
| Akr1a1 | S.i4vl7 | aldo-keto reductase family 1, member A1 (aldehyde reductase) |
| Akr1a1 | S.i3vl7 | aldo-keto reductase family 1, member A1 (aldehyde reductase) |
| Akr1a1 | S.i1vl7 | aldo-keto reductase family 1, member A1 (aldehyde reductase) |
| Akr1b8 | S.c4 | aldo-keto reductase family 1, member B8 |
| Akr1b8 | S.i4vl7 | aldo-keto reductase family 1, member B8 |
| Akt1 | S.i4vl7 | thymoma viral proto-oncogene 1 |
| Akt1 | S.i3vl7 | thymoma viral proto-oncogene 1 |
| Akt1 | S.i1vl7 | thymoma viral proto-oncogene 1 |
| Akt1 | S.i2vl7 | thymoma viral proto-oncogene 1 |
| Akt1 | S.c7 | thymoma viral proto-oncogene 1 |
| Akt2 | S.c2 | thymoma viral proto-oncogene 2 |
| Akt2 | S.i3vl7 | thymoma viral proto-oncogene 2 |
| Aktip | S.i4vl7 | thymoma viral proto-oncogene 1 interacting protein |
| Aktip | S.c6 | thymoma viral proto-oncogene 1 interacting protein |
| Aktip | S.c8 | thymoma viral proto-oncogene 1 interacting protein |
| Alad | S.i4vl7 | aminolevulinate, delta-, dehydratase |
| Alas1 | S.c2 | aminolevulinic acid synthase 1 |
| Aldh18a1 | S.c7 | aldehyde dehydrogenase 18 family, member A1 |
| Aldh18a1 | S.i4vl7 | aldehyde dehydrogenase 18 family, member A1 |
| Aldh18a1 | S.i1vl7 | aldehyde dehydrogenase 18 family, member A1 |
| Aldh18a1 | S.i2vl7 | aldehyde dehydrogenase 18 family, member A1 |
| Aldh18a1 | S.i3vl7 | aldehyde dehydrogenase 18 family, member A1 |
| Aldh1a1 | S.i4vl7 | aldehyde dehydrogenase family 1, subfamily A1 |
| Aldh1a1 | S.i2vl7 | aldehyde dehydrogenase family 1, subfamily A1 |
| Aldh1a1 | S.c2 | aldehyde dehydrogenase family 1, subfamily A1 |
| Aldh1a7 | S.c1 | aldehyde dehydrogenase family 1, subfamily A7 |
| Aldh1a7 | S.i4vl7 | aldehyde dehydrogenase family 1, subfamily A7 |
| Aldh7a1 | S.i3vl7 | aldehyde dehydrogenase family 7, member A1 |
| Aldh7a1 | S.i2vl7 | aldehyde dehydrogenase family 7, member A1 |
| Aldh7a1 | S.i4vl7 | aldehyde dehydrogenase family 7, member A1 |
| Aldh7a1 | S.c8 | aldehyde dehydrogenase family 7, member A1 |
| Aldoa | S.i4vl7 | aldolase A, fructose-bisphosphate |
| Aldoa | S.c3 | aldolase A, fructose-bisphosphate |
| Aldoart2 | S.i4vl7 | aldolase 1 A, retrogene 2 |
| Aldoc | S.i2vl7 | aldolase C, fructose-bisphosphate |
| Aldoc | S.c7 | aldolase C, fructose-bisphosphate |
| Aldoc | S.i1vl7 | aldolase C, fructose-bisphosphate |
| Aldoc | S.i4vl7 | aldolase C, fructose-bisphosphate |
| Alg3 | S.c7 | asparagine-linked glycosylation 3 (alpha-1,3-mannosyltransferase) |
| Alyref | S.c2 | Aly/REF export factor |
| Amfr | S.i1vl7 | autocrine motility factor receptor |
| Amot | S.c9 | angiomotin |
| Amot | S.i3vl7 | angiomotin |
| Amotl2 | S.i1vl7 | angiomotin-like 2 |
| Amotl2 | S.c6 | angiomotin-like 2 |
| Amotl2 | S.i4vl7 | angiomotin-like 2 |
| Amy1 | S.i4vl7 | amylase 1, salivary |
| Amy1 | S.c3 | amylase 1, salivary |
| Amz2 | S.i1vl7 | archaelysin family metallopeptidase 2 |
| Amz2 | S.c6 | archaelysin family metallopeptidase 2 |
| Amz2 | S.i2vl7 | archaelysin family metallopeptidase 2 |
| Anapc11 | S.i1vl7 | anaphase promoting complex subunit 11 |
| Anapc11 | S.i4vl7 | anaphase promoting complex subunit 11 |
| Anapc11 | S.i2vl7 | anaphase promoting complex subunit 11 |
| Anapc11 | S.i3vl7 | anaphase promoting complex subunit 11 |
| Anapc11 | S.c3 | anaphase promoting complex subunit 11 |
| Anapc13 | S.c7 | anaphase promoting complex subunit 13 |
| Anapc2 | c5.i3vl7 | anaphase promoting complex subunit 2 |
| Anapc2 | S.i1vl7 | anaphase promoting complex subunit 2 |
| Anapc2 | S.i3vl7 | anaphase promoting complex subunit 2 |
| Anapc2 | S.i2vl7 | anaphase promoting complex subunit 2 |
| Anapc4 | S.c4 | anaphase promoting complex subunit 4 |
| Anapc4 | S.i2vl7 | anaphase promoting complex subunit 4 |
| Anapc4 | S.i1vl7 | anaphase promoting complex subunit 4 |
| Anapc4 | S.i4vl7 | anaphase promoting complex subunit 4 |
| Angel2 | S.c4 | angel homolog 2 (Drosophila) |
| Angptl2 | S.c2 | angiopoietin-like 2 |
| Angptl2 | S.i3vl7 | angiopoietin-like 2 |
| Angptl2 | S.i2vl7 | angiopoietin-like 2 |
| Angptl2 | S.i4vl7 | angiopoietin-like 2 |
| Angptl4 | S.i4vl7 | angiopoietin-like 4 |
| Angptl4 | c5.i3vl7 | angiopoietin-like 4 |
| Angptl4 | S.i1vl7 | angiopoietin-like 4 |
| Angptl4 | S.i2vl7 | angiopoietin-like 4 |
| Angptl4 | S.i3vl7 | angiopoietin-like 4 |
| Ankrd42 | S.i3vl7 | ankyrin repeat domain 42 |
| Ankrd42 | S.i4vl7 | ankyrin repeat domain 42 |
| Ankrd42 | S.i2vl7 | ankyrin repeat domain 42 |
| Ankrd42 | S.i1vl7 | ankyrin repeat domain 42 |
| Ankrd42 | S.c4 | ankyrin repeat domain 42 |
| Anp32a | S.c1 | acidic (leucine-rich) nuclear phosphoprotein 32 family, member A |
| Anp32a | S.c7 | acidic (leucine-rich) nuclear phosphoprotein 32 family, member A |
| Anp32b | S.c4 | acidic (leucine-rich) nuclear phosphoprotein 32 family, member B |
| Anpep | c5.i3vl7 | alanyl (membrane) aminopeptidase |
| Anpep | S.i2vl7 | alanyl (membrane) aminopeptidase |
| Anpep | S.i1vl7 | alanyl (membrane) aminopeptidase |
| Anpep | S.i3vl7 | alanyl (membrane) aminopeptidase |
| Anpep | S.i4vl7 | alanyl (membrane) aminopeptidase |
| Antxr2 | S.i4vl7 | anthrax toxin receptor 2 |
| Antxr2 | S.i2vl7 | anthrax toxin receptor 2 |
| Antxr2 | S.c1 | anthrax toxin receptor 2 |
| Antxr2 | S.i3vl7 | anthrax toxin receptor 2 |
| Anxa1 | S.c3 | annexin A1 |
| Anxa1 | S.i3vl7 | annexin A1 |
| Anxa1 | S.i2vl7 | annexin A1 |
| Anxa1 | S.i1vl7 | annexin A1 |
| Anxa1 | S.i4vl7 | annexin A1 |
| Anxa1 | S.c4 | annexin A1 |
| Anxa11 | S.c4 | annexin A11 |
| Anxa11 | S.i4vl7 | annexin A11 |
| Anxa11 | S.i3vl7 | annexin A11 |
| Anxa11 | S.i1vl7 | annexin A11 |
| Anxa2 | S.i2vl7 | annexin A2 |
| Anxa2 | S.i1vl7 | annexin A2 |
| Anxa2 | S.i3vl7 | annexin A2 |
| Anxa2 | S.i4vl7 | annexin A2 |
| Anxa2 | S.c3 | annexin A2 |
| Anxa3 | S.i2vl7 | annexin A3 |
| Anxa3 | S.i4vl7 | annexin A3 |
| Anxa3 | S.c4 | annexin A3 |
| Anxa4 | S.i3vl7 | annexin A4 |
| Anxa4 | S.i4vl7 | annexin A4 |
| Anxa4 | S.i1vl7 | annexin A4 |
| Anxa4 | S.i2vl7 | annexin A4 |
| Anxa4 | S.c4 | annexin A4 |
| Anxa5 | S.i1vl7 | annexin A5 |
| Anxa5 | S.c4 | annexin A5 |
| Anxa5 | S.i2vl7 | annexin A5 |
| Anxa5 | S.i4vl7 | annexin A5 |
| Anxa8 | S.i4vl7 | annexin A8 |
| Anxa8 | S.c3 | annexin A8 |
| Aoc3 | S.c3 | amine oxidase, copper containing 3 |
| Aoc3 | S.i4vl7 | amine oxidase, copper containing 3 |
| Aoc3 | S.i2vl7 | amine oxidase, copper containing 3 |
| Aoc3 | S.i3vl7 | amine oxidase, copper containing 3 |
| Aox1 | S.c1 | aldehyde oxidase 1 |
| Aox1 | S.i3vl7 | aldehyde oxidase 1 |
| Ap1g1 | S.c7 | adaptor protein complex AP-1, gamma 1 subunit |
| Ap3b1 | S.c4 | adaptor-related protein complex 3, beta 1 subunit |
| Apbb1ip | S.i3vl7 | amyloid beta (A4) precursor protein-binding, family B, member 1 interacting protein |
| Apbb1ip | S.c3 | amyloid beta (A4) precursor protein-binding, family B, member 1 interacting protein |
| Apbb1ip | S.i4vl7 | amyloid beta (A4) precursor protein-binding, family B, member 1 interacting protein |
| Apbb1ip | S.i2vl7 | amyloid beta (A4) precursor protein-binding, family B, member 1 interacting protein |
| Apcdd1 | S.c1 | adenomatosis polyposis coli down-regulated 1 |
| Apcdd1 | S.i3vl7 | adenomatosis polyposis coli down-regulated 1 |
| Apcdd1 | S.i4vl7 | adenomatosis polyposis coli down-regulated 1 |
| Apcdd1 | S.i2vl7 | adenomatosis polyposis coli down-regulated 1 |
| Api5 | S.i4vl7 | apoptosis inhibitor 5 |
| Apip | S.i4vl7 | APAF1 interacting protein |
| Apip | S.c7 | APAF1 interacting protein |
| Apip | S.i2vl7 | APAF1 interacting protein |
| Aplp2 | S.i2vl7 | amyloid beta (A4) precursor-like protein 2 |
| Aplp2 | c5.i3vl7 | amyloid beta (A4) precursor-like protein 2 |
| Aplp2 | S.i1vl7 | amyloid beta (A4) precursor-like protein 2 |
| Aplp2 | S.i3vl7 | amyloid beta (A4) precursor-like protein 2 |
| Apobec1 | S.i3vl7 | apolipoprotein B mRNA editing enzyme, catalytic polypeptide 1 |
| Apobec1 | S.i2vl7 | apolipoprotein B mRNA editing enzyme, catalytic polypeptide 1 |
| Apobec1 | S.i4vl7 | apolipoprotein B mRNA editing enzyme, catalytic polypeptide 1 |
| Apobec1 | S.c2 | apolipoprotein B mRNA editing enzyme, catalytic polypeptide 1 |
| Apoc1 | S.c1 | apolipoprotein C-I |
| Apoc2 | S.c1 | apolipoprotein C-II |
| Apod | S.c1 | apolipoprotein D |
| Apod | S.i4vl7 | apolipoprotein D |
| Apoe | S.i4vl7 | apolipoprotein E |
| Apoe | S.c2 | apolipoprotein E |
| Apoe | S.i3vl7 | apolipoprotein E |
| App | S.i2vl7 | amyloid beta (A4) precursor protein |
| App | S.i3vl7 | amyloid beta (A4) precursor protein |
| App | S.c3 | amyloid beta (A4) precursor protein |
| App | S.i4vl7 | amyloid beta (A4) precursor protein |
| Aqp1 | S.c2 | aquaporin 1 |
| Arfgap3 | S.i4vl7 | ADP-ribosylation factor GTPase activating protein 3 |
| Arfgap3 | S.c9 | ADP-ribosylation factor GTPase activating protein 3 |
| Arfrp1 | S.i4vl7 | ADP-ribosylation factor related protein 1 |
| Arfrp1 | S.c4 | ADP-ribosylation factor related protein 1 |
| Arfrp1 | S.i3vl7 | ADP-ribosylation factor related protein 1 |
| Arg1 | S.c3 | arginase, liver |
| Arg2 | S.i2vl7 | arginase type II |
| Arg2 | S.i1vl7 | arginase type II |
| Arg2 | S.c8 | arginase type II |
| Arg2 | S.i4vl7 | arginase type II |
| Arglu1 | S.c3 | arginine and glutamate rich 1 |
| Arglu1 | S.i3vl7 | arginine and glutamate rich 1 |
| Arglu1 | S.i2vl7 | arginine and glutamate rich 1 |
| Arglu1 | S.i4vl7 | arginine and glutamate rich 1 |
| Arhgap1 | S.i4vl7 | Rho GTPase activating protein 1 |
| Arhgap1 | S.i3vl7 | Rho GTPase activating protein 1 |
| Arhgap1 | S.c3 | Rho GTPase activating protein 1 |
| Arhgap1 | S.i2vl7 | Rho GTPase activating protein 1 |
| Arhgdia | S.c2 | Rho GDP dissociation inhibitor (GDI) alpha |
| Arhgef3 | S.c2 | Rho guanine nucleotide exchange factor (GEF) 3 |
| Arid2 | S.c3 | AT rich interactive domain 2 (ARID, RFX-like) |
| Arid2 | S.i3vl7 | AT rich interactive domain 2 (ARID, RFX-like) |
| Arl2 | S.c1 | ADP-ribosylation factor-like 2 |
| Arl4a | S.i3vl7 | ADP-ribosylation factor-like 4A |
| Arl4a | S.c7 | ADP-ribosylation factor-like 4A |
| Arl4c | S.c1 | ADP-ribosylation factor-like 4C |
| Arl6 | S.i4vl7 | ADP-ribosylation factor-like 6 |
| Arl6 | S.c3 | ADP-ribosylation factor-like 6 |
| Arl6ip1 | S.i3vl7 | ADP-ribosylation factor-like 6 interacting protein 1 |
| Arl6ip1 | S.c4 | ADP-ribosylation factor-like 6 interacting protein 1 |
| Arl6ip1 | S.i1vl7 | ADP-ribosylation factor-like 6 interacting protein 1 |
| Arl6ip1 | S.i4vl7 | ADP-ribosylation factor-like 6 interacting protein 1 |
| Arl6ip1 | S.i2vl7 | ADP-ribosylation factor-like 6 interacting protein 1 |
| Armc10 | S.i2vl7 | armadillo repeat containing 10 |
| Armc10 | S.i3vl7 | armadillo repeat containing 10 |
| Armc10 | S.c4 | armadillo repeat containing 10 |
| Armc10 | S.i4vl7 | armadillo repeat containing 10 |
| Armc10 | S.i1vl7 | armadillo repeat containing 10 |
| Arpc2 | S.i3vl7 | actin related protein 2/3 complex, subunit 2 |
| Arpc2 | S.i2vl7 | actin related protein 2/3 complex, subunit 2 |
| Arpc2 | S.i4vl7 | actin related protein 2/3 complex, subunit 2 |
| Arpc2 | S.i1vl7 | actin related protein 2/3 complex, subunit 2 |
| Arpc2 | S.c4 | actin related protein 2/3 complex, subunit 2 |
| Arpc3 | S.i3vl7 | actin related protein 2/3 complex, subunit 3 |
| Arpc3 | S.i1vl7 | actin related protein 2/3 complex, subunit 3 |
| Arpc3 | S.i2vl7 | actin related protein 2/3 complex, subunit 3 |
| Arpc3 | c5.i3vl7 | actin related protein 2/3 complex, subunit 3 |
| Arrb1 | S.c1 | arrestin, beta 1 |
| Arsa | S.i3vl7 | arylsulfatase A |
| Arsa | c5.i3vl7 | arylsulfatase A |
| Arsa | S.i2vl7 | arylsulfatase A |
| Art3 | S.c2 | ADP-ribosyltransferase 3 |
| Art3 | S.i4vl7 | ADP-ribosyltransferase 3 |
| Art3 | S.i3vl7 | ADP-ribosyltransferase 3 |
| Arxes1 | S.c3 | adipocyte-related X-chromosome expressed sequence 1 |
| Arxes1 | S.i4vl7 | adipocyte-related X-chromosome expressed sequence 1 |
| Arxes1 | S.i2vl7 | adipocyte-related X-chromosome expressed sequence 1 |
| Arxes2 | S.i2vl7 | adipocyte-related X-chromosome expressed sequence 2 |
| Arxes2 | S.c3 | adipocyte-related X-chromosome expressed sequence 2 |
| Arxes2 | S.i4vl7 | adipocyte-related X-chromosome expressed sequence 2 |
| Asap1 | S.i3vl7 | ArfGAP with SH3 domain, ankyrin repeat and PH domain1 |
| Asap1 | S.i1vl7 | ArfGAP with SH3 domain, ankyrin repeat and PH domain1 |
| Asap1 | S.i2vl7 | ArfGAP with SH3 domain, ankyrin repeat and PH domain1 |
| Asap1 | S.i4vl7 | ArfGAP with SH3 domain, ankyrin repeat and PH domain1 |
| Asap1 | S.c6 | ArfGAP with SH3 domain, ankyrin repeat and PH domain1 |
| Asgr1 | S.c8 | asialoglycoprotein receptor 1 |
| Asns | S.i4vl7 | asparagine synthetase |
| Asns | S.c7 | asparagine synthetase |
| Asns | S.i2vl7 | asparagine synthetase |
| Atf1 | S.c4 | activating transcription factor 1 |
| Atf1 | S.i1vl7 | activating transcription factor 1 |
| Atf2 | S.i4vl7 | activating transcription factor 2 |
| Atf2 | S.c4 | activating transcription factor 2 |
| Atf3 | S.i4vl7 | activating transcription factor 3 |
| Atf3 | S.c3 | activating transcription factor 3 |
| Atf3 | S.i3vl7 | activating transcription factor 3 |
| Atf4 | S.c8 | activating transcription factor 4 |
| Atf5 | S.c3 | activating transcription factor 5 |
| Atf6b | S.c7 | activating transcription factor 6 beta |
| Atg14 | S.i3vl7 | autophagy related 14 |
| Atg14 | c5.i3vl7 | autophagy related 14 |
| Atox1 | S.i4vl7 | ATX1 (antioxidant protein 1) homolog 1 (yeast) |
| Atox1 | S.i3vl7 | ATX1 (antioxidant protein 1) homolog 1 (yeast) |
| Atox1 | c5.i3vl7 | ATX1 (antioxidant protein 1) homolog 1 (yeast) |
| Atox1 | S.i1vl7 | ATX1 (antioxidant protein 1) homolog 1 (yeast) |
| Atp11a | S.c3 | ATPase, class VI, type 11A |
| Atp11a | S.i2vl7 | ATPase, class VI, type 11A |
| Atp11a | S.i3vl7 | ATPase, class VI, type 11A |
| Atp11a | S.i4vl7 | ATPase, class VI, type 11A |
| Atp13a2 | S.i1vl7 | ATPase type 13A2 |
| Atp13a2 | S.c7 | ATPase type 13A2 |
| Atp1a1 | S.i1vl7 | ATPase, Na+/K+ transporting, alpha 1 polypeptide |
| Atp1a1 | S.c6 | ATPase, Na+/K+ transporting, alpha 1 polypeptide |
| Atp1a2 | S.c2 | ATPase, Na+/K+ transporting, alpha 2 polypeptide |
| Atp1b3 | S.c4 | ATPase, Na+/K+ transporting, beta 3 polypeptide |
| Atp1b3 | S.i4vl7 | ATPase, Na+/K+ transporting, beta 3 polypeptide |
| Atp1b3 | S.i1vl7 | ATPase, Na+/K+ transporting, beta 3 polypeptide |
| Atp1b3 | S.i3vl7 | ATPase, Na+/K+ transporting, beta 3 polypeptide |
| Atp2a3 | S.c1 | ATPase, Ca++ transporting, ubiquitous |
| Atp2b2 | S.i1vl7 | ATPase, Ca++ transporting, plasma membrane 2 |
| Atp2b2 | S.i2vl7 | ATPase, Ca++ transporting, plasma membrane 2 |
| Atp2b2 | S.i4vl7 | ATPase, Ca++ transporting, plasma membrane 2 |
| Atp2b2 | S.i3vl7 | ATPase, Ca++ transporting, plasma membrane 2 |
| Atp2b2 | S.c7 | ATPase, Ca++ transporting, plasma membrane 2 |
| Atp5e | S.c7 | ATP synthase, H+ transporting, mitochondrial F1 complex, epsilon subunit |
| Atp5o | S.c7 | ATP synthase, H+ transporting, mitochondrial F1 complex, O subunit |
| Atp6ap1 | S.i4vl7 | ATPase, H+ transporting, lysosomal accessory protein 1 |
| Atp6ap1 | S.i2vl7 | ATPase, H+ transporting, lysosomal accessory protein 1 |
| Atp6ap1 | S.c8 | ATPase, H+ transporting, lysosomal accessory protein 1 |
| Atp6v0e | S.i1vl7 | ATPase, H+ transporting, lysosomal V0 subunit E |
| Atp6v1a | S.c4 | ATPase, H+ transporting, lysosomal V1 subunit A |
| Atp6v1a | S.i3vl7 | ATPase, H+ transporting, lysosomal V1 subunit A |
| Atp6v1a | S.c6 | ATPase, H+ transporting, lysosomal V1 subunit A |
| Atp6v1a | S.i1vl7 | ATPase, H+ transporting, lysosomal V1 subunit A |
| Atp6v1a | S.i4vl7 | ATPase, H+ transporting, lysosomal V1 subunit A |
| Atp6v1a | S.i2vl7 | ATPase, H+ transporting, lysosomal V1 subunit A |
| Atp6v1b2 | S.c4 | ATPase, H+ transporting, lysosomal V1 subunit B2 |
| Atp6v1b2 | S.i1vl7 | ATPase, H+ transporting, lysosomal V1 subunit B2 |
| Atp6v1b2 | S.i2vl7 | ATPase, H+ transporting, lysosomal V1 subunit B2 |
| Atp6v1b2 | S.i3vl7 | ATPase, H+ transporting, lysosomal V1 subunit B2 |
| Atp6v1b2 | S.i4vl7 | ATPase, H+ transporting, lysosomal V1 subunit B2 |
| Atp6v1f | S.i4vl7 | ATPase, H+ transporting, lysosomal V1 subunit F |
| Atp6v1f | S.c3 | ATPase, H+ transporting, lysosomal V1 subunit F |
| Atp6v1f | S.i2vl7 | ATPase, H+ transporting, lysosomal V1 subunit F |
| Atp6v1f | S.i3vl7 | ATPase, H+ transporting, lysosomal V1 subunit F |
| Atp9a | S.i4vl7 | ATPase, class II, type 9A |
| Atpif1 | S.i4vl7 | ATPase inhibitory factor 1 |
| Atpif1 | c5.i3vl7 | ATPase inhibitory factor 1 |
| Atpif1 | S.i2vl7 | ATPase inhibitory factor 1 |
| Atpif1 | S.i3vl7 | ATPase inhibitory factor 1 |
| Atpif1 | S.i1vl7 | ATPase inhibitory factor 1 |
| Atxn10 | S.i2vl7 | ataxin 10 |
| Atxn10 | S.i4vl7 | ataxin 10 |
| Atxn10 | S.c4 | ataxin 10 |
| Atxn10 | S.i3vl7 | ataxin 10 |
| Atxn10 | S.i1vl7 | ataxin 10 |
| Atxn2 | S.i1vl7 | ataxin 2 |
| Atxn2 | S.i4vl7 | ataxin 2 |
| Atxn2 | S.c4 | ataxin 2 |
| AU040320 | S.i1vl7 | expressed sequence AU040320 |
| AU040320 | S.c6 | expressed sequence AU040320 |
| AU040320 | S.i2vl7 | expressed sequence AU040320 |
| Azin1 | S.i4vl7 | antizyme inhibitor 1 |
| Azin1 | S.i3vl7 | antizyme inhibitor 1 |
| Azin1 | S.i2vl7 | antizyme inhibitor 1 |
| Azin1 | S.c8 | antizyme inhibitor 1 |
| B2m | S.c1 | beta-2 microglobulin |
| B2m | S.i4vl7 | beta-2 microglobulin |
| B3galnt1 | S.c1 | UDP-GalNAc:betaGlcNAc beta 1,3-galactosaminyltransferase, polypeptide 1 |
| B4galnt1 | S.i2vl7 | beta-1,4-N-acetyl-galactosaminyl transferase 1 |
| B4galnt1 | c5.i3vl7 | beta-1,4-N-acetyl-galactosaminyl transferase 1 |
| B4galnt1 | S.i3vl7 | beta-1,4-N-acetyl-galactosaminyl transferase 1 |
| B4galt1 | S.i3vl7 | UDP-Gal:betaGlcNAc beta 1,4- galactosyltransferase, polypeptide 1 |
| B4galt1 | S.i2vl7 | UDP-Gal:betaGlcNAc beta 1,4- galactosyltransferase, polypeptide 1 |
| B4galt1 | S.c8 | UDP-Gal:betaGlcNAc beta 1,4- galactosyltransferase, polypeptide 1 |
| B4galt1 | S.i4vl7 | UDP-Gal:betaGlcNAc beta 1,4- galactosyltransferase, polypeptide 1 |
| B4galt6 | S.i4vl7 | UDP-Gal:betaGlcNAc beta 1,4-galactosyltransferase, polypeptide 6 |
| B4galt6 | S.c4 | UDP-Gal:betaGlcNAc beta 1,4-galactosyltransferase, polypeptide 6 |
| B4galt6 | S.i2vl7 | UDP-Gal:betaGlcNAc beta 1,4-galactosyltransferase, polypeptide 6 |
| Bach1 | S.i3vl7 | BTB and CNC homology 1 |
| Bach1 | S.i2vl7 | BTB and CNC homology 1 |
| Bach1 | S.c4 | BTB and CNC homology 1 |
| Bach1 | S.i4vl7 | BTB and CNC homology 1 |
| Bach1 | S.i1vl7 | BTB and CNC homology 1 |
| Bag2 | S.i4vl7 | BCL2-associated athanogene 2 |
| Bag2 | S.i3vl7 | BCL2-associated athanogene 2 |
| Bag2 | S.i2vl7 | BCL2-associated athanogene 2 |
| Bag2 | S.c8 | BCL2-associated athanogene 2 |
| Baiap2 | S.c3 | brain-specific angiogenesis inhibitor 1-associated protein 2 |
| Baiap2l1 | S.c7 | BAI1-associated protein 2-like 1 |
| Basp1 | S.i1vl7 | brain abundant, membrane attached signal protein 1 |
| Basp1 | S.c4 | brain abundant, membrane attached signal protein 1 |
| Basp1 | c5.i3vl7 | brain abundant, membrane attached signal protein 1 |
| Basp1 | S.i2vl7 | brain abundant, membrane attached signal protein 1 |
| Basp1 | S.i3vl7 | brain abundant, membrane attached signal protein 1 |
| Basp1 | S.i4vl7 | brain abundant, membrane attached signal protein 1 |
| Bax | S.i3vl7 | BCL2-associated X protein |
| Bax | c5.i3vl7 | BCL2-associated X protein |
| BC005537 | S.c3 | cDNA sequence BC005537 |
| BC037034 | S.i3vl7 | cDNA sequence BC037034 |
| BC037034 | S.i1vl7 | cDNA sequence BC037034 |
| BC037034 | S.i4vl7 | cDNA sequence BC037034 |
| BC037034 | S.c3 | cDNA sequence BC037034 |
| Bcan | S.i2vl7 | brevican |
| Bcan | S.i1vl7 | brevican |
| Bcan | S.c8 | brevican |
| Bcan | S.i4vl7 | brevican |
| Bcat2 | S.c7 | branched chain aminotransferase 2, mitochondrial |
| Bckdha | S.c7 | branched chain ketoacid dehydrogenase E1, alpha polypeptide |
| Bckdhb | S.c1 | branched chain ketoacid dehydrogenase E1, beta polypeptide |
| Bckdk | S.c8 | branched chain ketoacid dehydrogenase kinase |
| Bckdk | S.i4vl7 | branched chain ketoacid dehydrogenase kinase |
| Bcl2a1b | S.i4vl7 | B cell leukemia/lymphoma 2 related protein A1b |
| Bcl2a1b | S.c1 | B cell leukemia/lymphoma 2 related protein A1b |
| Bcl2a1c | S.i4vl7 | B cell leukemia/lymphoma 2 related protein A1c |
| Bcl2a1c | S.c1 | B cell leukemia/lymphoma 2 related protein A1c |
| Bcl2l1 | c5.i3vl7 | BCL2-like 1 |
| Bcl2l1 | S.i3vl7 | BCL2-like 1 |
| Bcl2l11 | S.c3 | BCL2-like 11 (apoptosis facilitator) |
| Bcl2l13 | S.c3 | BCL2-like 13 (apoptosis facilitator) |
| Bet1 | S.c8 | blocked early in transport 1 homolog (S. cerevisiae) |
| Bglap | c5.i3vl7 | bone gamma carboxyglutamate protein |
| Bglap | S.i3vl7 | bone gamma carboxyglutamate protein |
| Bglap2 | c5.i3vl7 | bone gamma-carboxyglutamate protein 2 |
| Bglap2 | S.i3vl7 | bone gamma-carboxyglutamate protein 2 |
| Bglap3 | c5.i3vl7 | bone gamma-carboxyglutamate protein 3 |
| Bglap3 | S.i3vl7 | bone gamma-carboxyglutamate protein 3 |
| Bglap3 | S.i1vl7 | bone gamma-carboxyglutamate protein 3 |
| Bgn | S.c2 | biglycan |
| Bhlhe40 | S.i3vl7 | basic helix-loop-helix family, member e40 |
| Bhlhe40 | S.c6 | basic helix-loop-helix family, member e40 |
| Bhlhe40 | S.i2vl7 | basic helix-loop-helix family, member e40 |
| Bhlhe40 | S.i1vl7 | basic helix-loop-helix family, member e40 |
| Birc5 | S.i3vl7 | baculoviral IAP repeat-containing 5 |
| Blnk | S.c4 | B cell linker |
| Blnk | S.c1 | B cell linker |
| Bnip2 | S.i4vl7 | BCL2/adenovirus E1B interacting protein 2 |
| Bnip2 | S.c3 | BCL2/adenovirus E1B interacting protein 2 |
| Bnip2 | S.i2vl7 | BCL2/adenovirus E1B interacting protein 2 |
| Bnip3l | S.c3 | BCL2/adenovirus E1B interacting protein 3-like |
| Bpgm | S.i4vl7 | 2,3-bisphosphoglycerate mutase |
| Brd4 | S.i4vl7 | bromodomain containing 4 |
| Brd4 | S.i3vl7 | bromodomain containing 4 |
| Bri3 | c5.i3vl7 | brain protein I3 |
| Bri3 | S.i4vl7 | brain protein I3 |
| Bri3 | S.i2vl7 | brain protein I3 |
| Bri3 | S.i1vl7 | brain protein I3 |
| Bri3 | S.i3vl7 | brain protein I3 |
| Brox | S.i1vl7 | BRO1 domain and CAAX motif containing |
| Bscl2 | S.c7 | Bernardinelli-Seip congenital lipodystrophy 2 homolog (human) |
| Bscl2 | S.i2vl7 | Bernardinelli-Seip congenital lipodystrophy 2 homolog (human) |
| Bscl2 | S.i1vl7 | Bernardinelli-Seip congenital lipodystrophy 2 homolog (human) |
| Btbd1 | S.i4vl7 | BTB (POZ) domain containing 1 |
| Btbd1 | S.c3 | BTB (POZ) domain containing 1 |
| Btbd1 | S.i2vl7 | BTB (POZ) domain containing 1 |
| Btf3 | S.c4 | basic transcription factor 3 |
| Btg2 | S.c6 | B cell translocation gene 2, anti-proliferative |
| Btg3 | S.i4vl7 | B cell translocation gene 3 |
| Btg3 | S.c7 | B cell translocation gene 3 |
| Btg3 | S.i1vl7 | B cell translocation gene 3 |
| Btg3 | S.i2vl7 | B cell translocation gene 3 |
| Btg3 | S.i3vl7 | B cell translocation gene 3 |
| Btn1a1 | S.c8 | butyrophilin, subfamily 1, member A1 |
| Bub3 | S.c4 | budding uninhibited by benzimidazoles 3 homolog (S. cerevisiae) |
| Bysl | S.c1 | bystin-like |
| Bysl | S.i4vl7 | bystin-like |
| Bzw2 | S.i2vl7 | basic leucine zipper and W2 domains 2 |
| Bzw2 | S.i3vl7 | basic leucine zipper and W2 domains 2 |
| Bzw2 | S.i1vl7 | basic leucine zipper and W2 domains 2 |
| Bzw2 | c5.i3vl7 | basic leucine zipper and W2 domains 2 |
| Bzw2 | S.i4vl7 | basic leucine zipper and W2 domains 2 |
| C1qa | S.c2 | complement component 1, q subcomponent, alpha polypeptide |
| C1qb | S.c2 | complement component 1, q subcomponent, beta polypeptide |
| C1qb | S.i4vl7 | complement component 1, q subcomponent, beta polypeptide |
| C1qb | S.i3vl7 | complement component 1, q subcomponent, beta polypeptide |
| C1qc | S.c2 | complement component 1, q subcomponent, C chain |
| C1qc | S.i4vl7 | complement component 1, q subcomponent, C chain |
| C1qc | S.i3vl7 | complement component 1, q subcomponent, C chain |
| C1ra | S.i4vl7 | complement component 1, r subcomponent A |
| C1ra | S.i3vl7 | complement component 1, r subcomponent A |
| C1ra | S.c1 | complement component 1, r subcomponent A |
| C1rb | S.c1 | complement component 1, r subcomponent B |
| C1rb | S.i3vl7 | complement component 1, r subcomponent B |
| C1rb | S.i4vl7 | complement component 1, r subcomponent B |
| C3 | S.i1vl7 | complement component 3 |
| C3 | S.c3 | complement component 3 |
| C3 | S.i2vl7 | complement component 3 |
| C3 | S.i4vl7 | complement component 3 |
| C3 | S.i3vl7 | complement component 3 |
| C3ar1 | S.c1 | complement component 3a receptor 1 |
| C4b | S.c3 | complement component 4B (Chido blood group) |
| C77080 | S.c6 | expressed sequence C77080 |
| C77080 | S.i1vl7 | expressed sequence C77080 |
| C9 | S.c8 | complement component 9 |
| Cab39l | S.c7 | calcium binding protein 39-like |
| Cacnb3 | S.i2vl7 | calcium channel, voltage-dependent, beta 3 subunit |
| Cadm1 | S.c4 | cell adhesion molecule 1 |
| Cadm1 | S.i2vl7 | cell adhesion molecule 1 |
| Cadm1 | S.i3vl7 | cell adhesion molecule 1 |
| Cadm1 | S.c3 | cell adhesion molecule 1 |
| Cadm1 | S.i4vl7 | cell adhesion molecule 1 |
| Cadm1 | S.i1vl7 | cell adhesion molecule 1 |
| Calm1 | S.i3vl7 | calmodulin 1 |
| Calm1 | S.i4vl7 | calmodulin 1 |
| Calm1 | S.c3 | calmodulin 1 |
| Calm2 | S.i3vl7 | calmodulin 2 |
| Calm2 | S.c3 | calmodulin 2 |
| Calm2 | S.i4vl7 | calmodulin 2 |
| Calm3 | S.c3 | calmodulin 3 |
| Calm3 | S.i3vl7 | calmodulin 3 |
| Calm3 | S.i4vl7 | calmodulin 3 |
| Caml | S.i1vl7 | calcium modulating ligand |
| Capg | S.i3vl7 | capping protein (actin filament), gelsolin-like |
| Capg | S.c4 | capping protein (actin filament), gelsolin-like |
| Capg | S.i4vl7 | capping protein (actin filament), gelsolin-like |
| Capg | S.i1vl7 | capping protein (actin filament), gelsolin-like |
| Capg | S.i2vl7 | capping protein (actin filament), gelsolin-like |
| Capn1 | S.c2 | calpain 1 |
| Capn1 | S.i3vl7 | calpain 1 |
| Capn2 | S.i3vl7 | calpain 2 |
| Capn2 | S.i2vl7 | calpain 2 |
| Capn2 | S.i4vl7 | calpain 2 |
| Capn2 | S.i1vl7 | calpain 2 |
| Capn2 | S.c4 | calpain 2 |
| Capn5 | S.i2vl7 | calpain 5 |
| Capn5 | S.i3vl7 | calpain 5 |
| Capn5 | S.i1vl7 | calpain 5 |
| Capn5 | S.i4vl7 | calpain 5 |
| Capn5 | S.c7 | calpain 5 |
| Capns1 | S.i1vl7 | calpain, small subunit 1 |
| Caprin1 | S.c4 | cell cycle associated protein 1 |
| Capza2 | S.c3 | capping protein (actin filament) muscle Z-line, alpha 2 |
| Capza2 | S.i4vl7 | capping protein (actin filament) muscle Z-line, alpha 2 |
| Capzb | S.c3 | capping protein (actin filament) muscle Z-line, beta |
| Capzb | S.i4vl7 | capping protein (actin filament) muscle Z-line, beta |
| Capzb | S.c1 | capping protein (actin filament) muscle Z-line, beta |
| Car2 | S.i4vl7 | carbonic anhydrase 2 |
| Car2 | S.i1vl7 | carbonic anhydrase 2 |
| Car2 | S.c7 | carbonic anhydrase 2 |
| Car2 | S.i2vl7 | carbonic anhydrase 2 |
| Car3 | S.i3vl7 | carbonic anhydrase 3 |
| Car3 | S.c2 | carbonic anhydrase 3 |
| Car3 | S.i2vl7 | carbonic anhydrase 3 |
| Car3 | S.i4vl7 | carbonic anhydrase 3 |
| Car6 | S.c6 | carbonic anhydrase 6 |
| Casp1 | S.i4vl7 | caspase 1 |
| Casp1 | S.c4 | caspase 1 |
| Casp3 | S.c7 | caspase 3 |
| Casp3 | S.i3vl7 | caspase 3 |
| Casp7 | S.c6 | caspase 7 |
| Casp7 | S.i1vl7 | caspase 7 |
| Cast | S.c3 | calpastatin |
| Cast | S.i1vl7 | calpastatin |
| Cast | S.i4vl7 | calpastatin |
| Cast | S.i3vl7 | calpastatin |
| Cast | S.i2vl7 | calpastatin |
| Cat | S.c3 | catalase |
| Cat | S.i2vl7 | catalase |
| Cav1 | S.c3 | caveolin 1, caveolae protein |
| Cav1 | S.i4vl7 | caveolin 1, caveolae protein |
| Cbfb | S.i3vl7 | core binding factor beta |
| Cbfb | S.c3 | core binding factor beta |
| Cbfb | S.i2vl7 | core binding factor beta |
| Cbfb | S.c4 | core binding factor beta |
| Cbfb | S.i4vl7 | core binding factor beta |
| Cbfb | S.i1vl7 | core binding factor beta |
| Cbx6 | S.c4 | chromobox 6 |
| Cbx6 | S.i4vl7 | chromobox 6 |
| Cbx6 | S.i3vl7 | chromobox 6 |
| Cbx6 | S.i2vl7 | chromobox 6 |
| Cbx6 | S.i1vl7 | chromobox 6 |
| Ccdc12 | S.i4vl7 | coiled-coil domain containing 12 |
| Ccdc12 | S.c4 | coiled-coil domain containing 12 |
| Ccdc12 | S.i1vl7 | coiled-coil domain containing 12 |
| Ccdc50 | S.i2vl7 | coiled-coil domain containing 50 |
| Ccdc50 | S.i3vl7 | coiled-coil domain containing 50 |
| Ccdc50 | S.c3 | coiled-coil domain containing 50 |
| Ccdc50 | S.i4vl7 | coiled-coil domain containing 50 |
| Ccdc80 | S.i2vl7 | coiled-coil domain containing 80 |
| Ccdc80 | S.i4vl7 | coiled-coil domain containing 80 |
| Ccdc80 | S.c3 | coiled-coil domain containing 80 |
| Ccdc90b | S.i1vl7 | coiled-coil domain containing 90B |
| Ccdc90b | S.i3vl7 | coiled-coil domain containing 90B |
| Ccdc90b | S.i4vl7 | coiled-coil domain containing 90B |
| Ccdc90b | S.c4 | coiled-coil domain containing 90B |
| Ccdc90b | S.i2vl7 | coiled-coil domain containing 90B |
| Cck | S.i4vl7 | cholecystokinin |
| Cck | S.c7 | cholecystokinin |
| Cck | S.i2vl7 | cholecystokinin |
| Cck | S.i1vl7 | cholecystokinin |
| Ccl11 | S.c1 | chemokine (C-C motif) ligand 11 |
| Ccl2 | S.i1vl7 | chemokine (C-C motif) ligand 2 |
| Ccl2 | S.c4 | chemokine (C-C motif) ligand 2 |
| Ccl21a | S.c1 | chemokine (C-C motif) ligand 21A (serine) |
| Ccl21b | S.c1 | chemokine (C-C motif) ligand 21B (leucine) |
| Ccl21c | S.c1 | chemokine (C-C motif) ligand 21C (leucine) |
| Ccl8 | S.i2vl7 | chemokine (C-C motif) ligand 8 |
| Ccl8 | S.c2 | chemokine (C-C motif) ligand 8 |
| Ccl8 | S.i4vl7 | chemokine (C-C motif) ligand 8 |
| Ccl9 | S.i4vl7 | chemokine (C-C motif) ligand 9 |
| Ccl9 | S.i2vl7 | chemokine (C-C motif) ligand 9 |
| Ccl9 | S.c4 | chemokine (C-C motif) ligand 9 |
| Ccl9 | S.i1vl7 | chemokine (C-C motif) ligand 9 |
| Ccnb2 | S.c1 | cyclin B2 |
| Ccnd1 | S.c7 | cyclin D1 |
| Ccnd1 | S.i2vl7 | cyclin D1 |
| Ccnd1 | S.i1vl7 | cyclin D1 |
| Ccnd1 | S.i3vl7 | cyclin D1 |
| Ccng1 | S.i4vl7 | cyclin G1 |
| Ccng1 | S.i2vl7 | cyclin G1 |
| Ccng2 | S.c6 | cyclin G2 |
| Ccni | c5.i3vl7 | cyclin I |
| Ccni | S.i2vl7 | cyclin I |
| Ccni | S.i3vl7 | cyclin I |
| Ccni | S.i1vl7 | cyclin I |
| Ccnl2 | S.i4vl7 | cyclin L2 |
| Ccnl2 | S.c3 | cyclin L2 |
| Ccnl2 | S.i2vl7 | cyclin L2 |
| Ccr2 | S.i2vl7 | chemokine (C-C motif) receptor 2 |
| Ccr2 | S.c1 | chemokine (C-C motif) receptor 2 |
| Ccr5 | S.c1 | chemokine (C-C motif) receptor 5 |
| Ccr5 | S.i4vl7 | chemokine (C-C motif) receptor 5 |
| Cct3 | S.i2vl7 | chaperonin containing Tcp1, subunit 3 (gamma) |
| Cct3 | S.c4 | chaperonin containing Tcp1, subunit 3 (gamma) |
| Cct3 | S.i1vl7 | chaperonin containing Tcp1, subunit 3 (gamma) |
| Cct4 | S.i1vl7 | chaperonin containing Tcp1, subunit 4 (delta) |
| Cct4 | S.i2vl7 | chaperonin containing Tcp1, subunit 4 (delta) |
| Cct4 | S.c4 | chaperonin containing Tcp1, subunit 4 (delta) |
| Cct6a | S.c8 | chaperonin containing Tcp1, subunit 6a (zeta) |
| Cct6a | S.i4vl7 | chaperonin containing Tcp1, subunit 6a (zeta) |
| Cct8 | S.i4vl7 | chaperonin containing Tcp1, subunit 8 (theta) |
| Cct8 | S.c4 | chaperonin containing Tcp1, subunit 8 (theta) |
| Cct8 | S.i3vl7 | chaperonin containing Tcp1, subunit 8 (theta) |
| Cd14 | S.i3vl7 | CD14 antigen |
| Cd14 | S.i4vl7 | CD14 antigen |
| Cd14 | c5.i3vl7 | CD14 antigen |
| Cd14 | S.i2vl7 | CD14 antigen |
| Cd14 | S.i1vl7 | CD14 antigen |
| Cd151 | S.i4vl7 | CD151 antigen |
| Cd151 | S.i2vl7 | CD151 antigen |
| Cd151 | S.c4 | CD151 antigen |
| Cd151 | S.i3vl7 | CD151 antigen |
| Cd151 | S.i1vl7 | CD151 antigen |
| Cd1d1 | S.i3vl7 | CD1d1 antigen |
| Cd1d1 | S.i2vl7 | CD1d1 antigen |
| Cd1d1 | S.i4vl7 | CD1d1 antigen |
| Cd1d1 | c5.i3vl7 | CD1d1 antigen |
| Cd1d1 | S.i1vl7 | CD1d1 antigen |
| Cd1d2 | S.c6 | CD1d2 antigen |
| Cd1d2 | S.i2vl7 | CD1d2 antigen |
| Cd1d2 | S.i4vl7 | CD1d2 antigen |
| Cd1d2 | S.i3vl7 | CD1d2 antigen |
| Cd1d2 | S.i1vl7 | CD1d2 antigen |
| Cd1d2 | c5.i3vl7 | CD1d2 antigen |
| Cd200 | S.c1 | CD200 antigen |
| Cd24a | S.i2vl7 | CD24a antigen |
| Cd24a | S.c9 | CD24a antigen |
| Cd24a | S.i4vl7 | CD24a antigen |
| Cd24a | S.i3vl7 | CD24a antigen |
| Cd27 | S.i3vl7 | CD27 antigen |
| Cd27 | S.i2vl7 | CD27 antigen |
| Cd27 | S.i4vl7 | CD27 antigen |
| Cd27 | S.c8 | CD27 antigen |
| Cd276 | S.i2vl7 | CD276 antigen |
| Cd2ap | S.c9 | CD2-associated protein |
| Cd302 | S.c2 | CD302 antigen |
| Cd302 | S.i2vl7 | CD302 antigen |
| Cd302 | S.i4vl7 | CD302 antigen |
| Cd320 | S.i2vl7 | CD320 antigen |
| Cd320 | S.i1vl7 | CD320 antigen |
| Cd320 | S.i3vl7 | CD320 antigen |
| Cd320 | S.c7 | CD320 antigen |
| Cd320 | S.i4vl7 | CD320 antigen |
| Cd34 | S.i3vl7 | CD34 antigen |
| Cd34 | S.i4vl7 | CD34 antigen |
| Cd34 | S.c2 | CD34 antigen |
| Cd36 | S.c7 | CD36 antigen |
| Cd47 | S.i1vl7 | CD47 antigen (Rh-related antigen, integrin-associated signal transducer) |
| Cd47 | S.c4 | CD47 antigen (Rh-related antigen, integrin-associated signal transducer) |
| Cd47 | S.i3vl7 | CD47 antigen (Rh-related antigen, integrin-associated signal transducer) |
| Cd47 | S.i4vl7 | CD47 antigen (Rh-related antigen, integrin-associated signal transducer) |
| Cd47 | S.i2vl7 | CD47 antigen (Rh-related antigen, integrin-associated signal transducer) |
| Cd48 | S.i4vl7 | CD48 antigen |
| Cd48 | S.c1 | CD48 antigen |
| Cd53 | S.c3 | CD53 antigen |
| Cd53 | S.i4vl7 | CD53 antigen |
| Cd55 | S.c9 | CD55 antigen |
| Cd55 | S.i4vl7 | CD55 antigen |
| Cd59a | S.i1vl7 | CD59a antigen |
| Cd59a | S.i3vl7 | CD59a antigen |
| Cd59a | S.i4vl7 | CD59a antigen |
| Cd59a | c5.i3vl7 | CD59a antigen |
| Cd59a | S.i2vl7 | CD59a antigen |
| Cd63 | c5.i3vl7 | CD63 antigen |
| Cd63 | S.i4vl7 | CD63 antigen |
| Cd63 | S.i1vl7 | CD63 antigen |
| Cd63 | S.i3vl7 | CD63 antigen |
| Cd63 | S.i2vl7 | CD63 antigen |
| Cd68 | S.i4vl7 | CD68 antigen |
| Cd68 | S.c3 | CD68 antigen |
| Cd68 | S.i3vl7 | CD68 antigen |
| Cd74 | S.c2 | CD74 antigen (invariant polypeptide of major histocompatibility complex, class II antigen-associated) |
| Cd74 | S.i3vl7 | CD74 antigen (invariant polypeptide of major histocompatibility complex, class II antigen-associated) |
| Cd74 | S.i2vl7 | CD74 antigen (invariant polypeptide of major histocompatibility complex, class II antigen-associated) |
| Cd74 | S.i4vl7 | CD74 antigen (invariant polypeptide of major histocompatibility complex, class II antigen-associated) |
| Cd82 | S.c7 | CD82 antigen |
| Cd9 | S.i1vl7 | CD9 antigen |
| Cd9 | S.c4 | CD9 antigen |
| Cd9 | S.i2vl7 | CD9 antigen |
| Cd9 | S.i3vl7 | CD9 antigen |
| Cd9 | S.i4vl7 | CD9 antigen |
| Cd93 | S.c2 | CD93 antigen |
| Cdc25a | S.c7 | cell division cycle 25A |
| Cdc26 | S.c6 | cell division cycle 26 |
| Cdc34 | S.c9 | cell division cycle 34 |
| Cdc34 | S.i4vl7 | cell division cycle 34 |
| Cdc42 | S.i1vl7 | cell division cycle 42 |
| Cdc42 | S.c4 | cell division cycle 42 |
| Cdc42 | S.i4vl7 | cell division cycle 42 |
| Cdh13 | S.c2 | cadherin 13 |
| Cdh5 | S.i3vl7 | cadherin 5 |
| Cdh5 | S.i4vl7 | cadherin 5 |
| Cdh5 | S.c3 | cadherin 5 |
| Cdip1 | S.i3vl7 | cell death inducing Trp53 target 1 |
| Cdip1 | S.i4vl7 | cell death inducing Trp53 target 1 |
| Cdip1 | S.i2vl7 | cell death inducing Trp53 target 1 |
| Cdip1 | c5.i3vl7 | cell death inducing Trp53 target 1 |
| Cdk18 | S.c7 | cyclin-dependent kinase 18 |
| Cdk4 | S.i2vl7 | cyclin-dependent kinase 4 |
| Cdk4 | S.i1vl7 | cyclin-dependent kinase 4 |
| Cdk4 | S.i3vl7 | cyclin-dependent kinase 4 |
| Cdk4 | S.c4 | cyclin-dependent kinase 4 |
| Cdkn1a | S.i3vl7 | cyclin-dependent kinase inhibitor 1A (P21) |
| Cdkn1a | c5.i3vl7 | cyclin-dependent kinase inhibitor 1A (P21) |
| Cdkn1c | S.i3vl7 | cyclin-dependent kinase inhibitor 1C (P57) |
| Cdkn2c | S.c1 | cyclin-dependent kinase inhibitor 2C (p18, inhibits CDK4) |
| Cdkn2c | S.i1vl7 | cyclin-dependent kinase inhibitor 2C (p18, inhibits CDK4) |
| Cdkn2c | S.i4vl7 | cyclin-dependent kinase inhibitor 2C (p18, inhibits CDK4) |
| Cdo1 | S.c2 | cysteine dioxygenase 1, cytosolic |
| Cdo1 | S.i3vl7 | cysteine dioxygenase 1, cytosolic |
| Cdo1 | S.i4vl7 | cysteine dioxygenase 1, cytosolic |
| Cdo1 | S.i2vl7 | cysteine dioxygenase 1, cytosolic |
| Ceacam2 | S.c6 | carcinoembryonic antigen-related cell adhesion molecule 2 |
| Ceacam2 | S.i1vl7 | carcinoembryonic antigen-related cell adhesion molecule 2 |
| Cebpa | S.i3vl7 | CCAAT/enhancer binding protein (C/EBP), alpha |
| Cebpa | S.i2vl7 | CCAAT/enhancer binding protein (C/EBP), alpha |
| Cebpa | S.i4vl7 | CCAAT/enhancer binding protein (C/EBP), alpha |
| Cebpa | S.c2 | CCAAT/enhancer binding protein (C/EBP), alpha |
| Cebpb | S.i3vl7 | CCAAT/enhancer binding protein (C/EBP), beta |
| Cebpb | S.c2 | CCAAT/enhancer binding protein (C/EBP), beta |
| Cebpb | S.i4vl7 | CCAAT/enhancer binding protein (C/EBP), beta |
| Cebpb | S.i2vl7 | CCAAT/enhancer binding protein (C/EBP), beta |
| Cebpz | S.c3 | CCAAT/enhancer binding protein zeta |
| Cecr5 | S.i3vl7 | cat eye syndrome chromosome region, candidate 5 |
| Cecr5 | S.i4vl7 | cat eye syndrome chromosome region, candidate 5 |
| Cecr5 | S.c8 | cat eye syndrome chromosome region, candidate 5 |
| Cel | S.i4vl7 | carboxyl ester lipase |
| Cel | S.c9 | carboxyl ester lipase |
| Celf2 | S.i4vl7 | CUGBP, Elav-like family member 2 |
| Celf2 | S.i2vl7 | CUGBP, Elav-like family member 2 |
| Celf2 | S.c3 | CUGBP, Elav-like family member 2 |
| Celf2 | S.i3vl7 | CUGBP, Elav-like family member 2 |
| Cenpb | S.i2vl7 | centromere protein B |
| Cenpb | S.i3vl7 | centromere protein B |
| Cenpb | S.c2 | centromere protein B |
| Cerk | S.c1 | ceramide kinase |
| Ces1d | S.c1 | carboxylesterase 1D |
| Ces1d | S.i4vl7 | carboxylesterase 1D |
| Cfd | S.c2 | complement factor D (adipsin) |
| Cfd | S.i4vl7 | complement factor D (adipsin) |
| Cfh | S.i4vl7 | complement component factor h |
| Cfh | S.c1 | complement component factor h |
| Cfl1 | S.i4vl7 | cofilin 1, non-muscle |
| Cfl1 | c5.i3vl7 | cofilin 1, non-muscle |
| Cfl1 | S.i3vl7 | cofilin 1, non-muscle |
| Cfp | S.i4vl7 | complement factor properdin |
| Cfp | S.c2 | complement factor properdin |
| Cfp | S.i2vl7 | complement factor properdin |
| Chchd5 | S.c6 | coiled-coil-helix-coiled-coil-helix domain containing 5 |
| Chd4 | S.c2 | chromodomain helicase DNA binding protein 4 |
| Chd4 | S.i4vl7 | chromodomain helicase DNA binding protein 4 |
| Chil1 | S.i4vl7 | chitinase-like 1 |
| Chil1 | S.c3 | chitinase-like 1 |
| Chil1 | S.i2vl7 | chitinase-like 1 |
| Chil1 | S.i3vl7 | chitinase-like 1 |
| Chka | S.c9 | choline kinase alpha |
| Chka | S.i4vl7 | choline kinase alpha |
| Chmp2b | S.c6 | charged multivesicular body protein 2B |
| Chmp2b | S.i1vl7 | charged multivesicular body protein 2B |
| Chmp2b | S.i2vl7 | charged multivesicular body protein 2B |
| Chmp2b | S.i3vl7 | charged multivesicular body protein 2B |
| Chmp2b | S.i4vl7 | charged multivesicular body protein 2B |
| Chmp4b | S.c4 | charged multivesicular body protein 4B |
| Chmp4b | S.i1vl7 | charged multivesicular body protein 4B |
| Chordc1 | S.c7 | cysteine and histidine-rich domain (CHORD)-containing, zinc-binding protein 1 |
| Chrnb1 | S.c3 | cholinergic receptor, nicotinic, beta polypeptide 1 (muscle) |
| Chrnb1 | S.i3vl7 | cholinergic receptor, nicotinic, beta polypeptide 1 (muscle) |
| Chtop | S.i2vl7 | chromatin target of PRMT1 |
| Chtop | S.i4vl7 | chromatin target of PRMT1 |
| Chtop | S.c3 | chromatin target of PRMT1 |
| Chtop | S.i3vl7 | chromatin target of PRMT1 |
| Cib2 | S.c1 | calcium and integrin binding family member 2 |
| Cib2 | S.i4vl7 | calcium and integrin binding family member 2 |
| Cic | S.i2vl7 | capicua homolog (Drosophila) |
| Cic | S.c2 | capicua homolog (Drosophila) |
| Cidea | S.c9 | cell death-inducing DNA fragmentation factor, alpha subunit-like effector A |
| Cidea | S.i2vl7 | cell death-inducing DNA fragmentation factor, alpha subunit-like effector A |
| Cidea | S.i4vl7 | cell death-inducing DNA fragmentation factor, alpha subunit-like effector A |
| Cidea | S.i1vl7 | cell death-inducing DNA fragmentation factor, alpha subunit-like effector A |
| Cidec | S.c2 | cell death-inducing DFFA-like effector c |
| Cidec | S.i4vl7 | cell death-inducing DFFA-like effector c |
| Cilp2 | S.i3vl7 | cartilage intermediate layer protein 2 |
| Cilp2 | S.c8 | cartilage intermediate layer protein 2 |
| Cilp2 | S.i4vl7 | cartilage intermediate layer protein 2 |
| Cirbp | S.c1 | cold inducible RNA binding protein |
| Clcn3 | S.i3vl7 | chloride channel 3 |
| Clcn3 | S.c9 | chloride channel 3 |
| Clcn3 | S.i4vl7 | chloride channel 3 |
| Clcn7 | S.i3vl7 | chloride channel 7 |
| Cldn3 | S.i2vl7 | claudin 3 |
| Cldn3 | S.i4vl7 | claudin 3 |
| Cldn3 | S.i3vl7 | claudin 3 |
| Cldn3 | c5.i3vl7 | claudin 3 |
| Cldn3 | S.c6 | claudin 3 |
| Cldn3 | S.i1vl7 | claudin 3 |
| Cldn4 | S.c4 | claudin 4 |
| Cldn4 | S.i1vl7 | claudin 4 |
| Cldn5 | S.i4vl7 | claudin 5 |
| Cldn5 | S.i2vl7 | claudin 5 |
| Cldn5 | c5.i3vl7 | claudin 5 |
| Cldn5 | S.i3vl7 | claudin 5 |
| Cldn8 | S.c8 | claudin 8 |
| Cldn8 | S.i3vl7 | claudin 8 |
| Cldn8 | S.i4vl7 | claudin 8 |
| Cldn8 | S.i2vl7 | claudin 8 |
| Clec3b | S.c2 | C-type lectin domain family 3, member b |
| Clec3b | S.i4vl7 | C-type lectin domain family 3, member b |
| Clic1 | S.i1vl7 | chloride intracellular channel 1 |
| Clic1 | S.i3vl7 | chloride intracellular channel 1 |
| Clic1 | S.i4vl7 | chloride intracellular channel 1 |
| Clic1 | S.c4 | chloride intracellular channel 1 |
| Clic1 | S.i2vl7 | chloride intracellular channel 1 |
| Clk3 | S.i2vl7 | CDC-like kinase 3 |
| Cln3 | S.i1vl7 | ceroid lipofuscinosis, neuronal 3, juvenile (Batten, Spielmeyer-Vogt disease) |
| Cln6 | S.c6 | ceroid-lipofuscinosis, neuronal 6 |
| Clptm1l | S.i2vl7 | CLPTM1-like |
| Clptm1l | S.c8 | CLPTM1-like |
| Clptm1l | S.i4vl7 | CLPTM1-like |
| Clptm1l | S.i3vl7 | CLPTM1-like |
| Clu | S.i2vl7 | clusterin |
| Clu | S.i1vl7 | clusterin |
| Clu | S.c6 | clusterin |
| Cma1 | S.c3 | chymase 1, mast cell |
| Cma1 | S.i4vl7 | chymase 1, mast cell |
| Cma1 | S.i2vl7 | chymase 1, mast cell |
| Cma1 | S.i3vl7 | chymase 1, mast cell |
| Cmas | S.c6 | cytidine monophospho-N-acetylneuraminic acid synthetase |
| Cmas | S.i1vl7 | cytidine monophospho-N-acetylneuraminic acid synthetase |
| Cmbl | S.i4vl7 | carboxymethylenebutenolidase-like (Pseudomonas) |
| Cmbl | S.i2vl7 | carboxymethylenebutenolidase-like (Pseudomonas) |
| Cmbl | S.c2 | carboxymethylenebutenolidase-like (Pseudomonas) |
| Cmip | S.i3vl7 | c-Maf inducing protein |
| Cmip | S.i2vl7 | c-Maf inducing protein |
| Cmip | S.c3 | c-Maf inducing protein |
| Cmip | S.i4vl7 | c-Maf inducing protein |
| Cml2 | S.c1 | camello-like 2 |
| Cml2 | S.i4vl7 | camello-like 2 |
| Cmpk2 | S.c7 | cytidine monophosphate (UMP-CMP) kinase 2, mitochondrial |
| Cmpk2 | S.i4vl7 | cytidine monophosphate (UMP-CMP) kinase 2, mitochondrial |
| Cmpk2 | S.i3vl7 | cytidine monophosphate (UMP-CMP) kinase 2, mitochondrial |
| Cmtm3 | S.c4 | CKLF-like MARVEL transmembrane domain containing 3 |
| Cmtm3 | S.i4vl7 | CKLF-like MARVEL transmembrane domain containing 3 |
| Cmtm6 | S.i4vl7 | CKLF-like MARVEL transmembrane domain containing 6 |
| Cmtm6 | S.i3vl7 | CKLF-like MARVEL transmembrane domain containing 6 |
| Cmtm6 | S.c7 | CKLF-like MARVEL transmembrane domain containing 6 |
| Cmtm6 | S.i1vl7 | CKLF-like MARVEL transmembrane domain containing 6 |
| Cmtm6 | S.i2vl7 | CKLF-like MARVEL transmembrane domain containing 6 |
| Cmtm8 | S.c7 | CKLF-like MARVEL transmembrane domain containing 8 |
| Cmtm8 | S.i1vl7 | CKLF-like MARVEL transmembrane domain containing 8 |
| Cmtm8 | S.i2vl7 | CKLF-like MARVEL transmembrane domain containing 8 |
| Cmtm8 | S.i4vl7 | CKLF-like MARVEL transmembrane domain containing 8 |
| Cnbp | S.c7 | cellular nucleic acid binding protein |
| Cnbp | S.i4vl7 | cellular nucleic acid binding protein |
| Cnih1 | S.i1vl7 | cornichon homolog 1 (Drosophila) |
| Cnih1 | S.c4 | cornichon homolog 1 (Drosophila) |
| Cnih1 | S.i4vl7 | cornichon homolog 1 (Drosophila) |
| Cnn1 | S.c9 | calponin 1 |
| Cnn1 | S.i3vl7 | calponin 1 |
| Cnn2 | S.i3vl7 | calponin 2 |
| Cnn2 | S.c3 | calponin 2 |
| Cnn2 | S.i4vl7 | calponin 2 |
| Cnn3 | S.i4vl7 | calponin 3, acidic |
| Cnn3 | S.c4 | calponin 3, acidic |
| Cnn3 | S.i2vl7 | calponin 3, acidic |
| Cnn3 | S.i1vl7 | calponin 3, acidic |
| Cnn3 | S.i3vl7 | calponin 3, acidic |
| Cnpy2 | S.i3vl7 | canopy 2 homolog (zebrafish) |
| Cnpy2 | S.i2vl7 | canopy 2 homolog (zebrafish) |
| Cnpy2 | S.i1vl7 | canopy 2 homolog (zebrafish) |
| Cnpy2 | c5.i3vl7 | canopy 2 homolog (zebrafish) |
| Coa3 | S.c4 | cytochrome C oxidase assembly factor 3 |
| Col14a1 | S.i4vl7 | collagen, type XIV, alpha 1 |
| Col14a1 | S.i2vl7 | collagen, type XIV, alpha 1 |
| Col14a1 | S.i3vl7 | collagen, type XIV, alpha 1 |
| Col14a1 | S.c2 | collagen, type XIV, alpha 1 |
| Col15a1 | S.i2vl7 | collagen, type XV, alpha 1 |
| Col15a1 | S.i3vl7 | collagen, type XV, alpha 1 |
| Col15a1 | S.c3 | collagen, type XV, alpha 1 |
| Col15a1 | S.i4vl7 | collagen, type XV, alpha 1 |
| Col1a1 | S.c3 | collagen, type I, alpha 1 |
| Col1a2 | S.c3 | collagen, type I, alpha 2 |
| Col1a2 | S.i2vl7 | collagen, type I, alpha 2 |
| Col1a2 | S.i4vl7 | collagen, type I, alpha 2 |
| Col3a1 | S.c3 | collagen, type III, alpha 1 |
| Col3a1 | S.i4vl7 | collagen, type III, alpha 1 |
| Col3a1 | S.c2 | collagen, type III, alpha 1 |
| Col3a1 | S.i3vl7 | collagen, type III, alpha 1 |
| Col3a1 | S.i2vl7 | collagen, type III, alpha 1 |
| Col4a1 | S.i4vl7 | collagen, type IV, alpha 1 |
| Col4a1 | S.c3 | collagen, type IV, alpha 1 |
| Col4a2 | S.c2 | collagen, type IV, alpha 2 |
| Col4a2 | S.i4vl7 | collagen, type IV, alpha 2 |
| Col4a5 | S.c6 | collagen, type IV, alpha 5 |
| Col5a1 | S.c2 | collagen, type V, alpha 1 |
| Col5a1 | S.i4vl7 | collagen, type V, alpha 1 |
| Col5a1 | S.c3 | collagen, type V, alpha 1 |
| Col5a2 | S.i2vl7 | collagen, type V, alpha 2 |
| Col5a2 | S.i3vl7 | collagen, type V, alpha 2 |
| Col5a2 | S.c3 | collagen, type V, alpha 2 |
| Col5a2 | S.i4vl7 | collagen, type V, alpha 2 |
| Col5a2 | S.i1vl7 | collagen, type V, alpha 2 |
| Col6a1 | S.c2 | collagen, type VI, alpha 1 |
| Col6a1 | S.i4vl7 | collagen, type VI, alpha 1 |
| Col6a2 | S.c2 | collagen, type VI, alpha 2 |
| Col6a3 | S.i1vl7 | collagen, type VI, alpha 3 |
| Col6a3 | S.i3vl7 | collagen, type VI, alpha 3 |
| Col6a3 | S.c3 | collagen, type VI, alpha 3 |
| Col6a3 | S.i2vl7 | collagen, type VI, alpha 3 |
| Col6a3 | S.i4vl7 | collagen, type VI, alpha 3 |
| Col9a2 | S.c8 | collagen, type IX, alpha 2 |
| Col9a2 | S.i2vl7 | collagen, type IX, alpha 2 |
| Col9a2 | S.i3vl7 | collagen, type IX, alpha 2 |
| Col9a2 | S.i4vl7 | collagen, type IX, alpha 2 |
| Commd4 | S.c3 | COMM domain containing 4 |
| Commd9 | S.c8 | COMM domain containing 9 |
| Commd9 | S.i4vl7 | COMM domain containing 9 |
| Commd9 | S.i2vl7 | COMM domain containing 9 |
| Comt | S.c4 | catechol-O-methyltransferase |
| Comt | S.i4vl7 | catechol-O-methyltransferase |
| Comt | S.i1vl7 | catechol-O-methyltransferase |
| Comt | S.i2vl7 | catechol-O-methyltransferase |
| Comt | S.i3vl7 | catechol-O-methyltransferase |
| Copb2 | S.c8 | coatomer protein complex, subunit beta 2 (beta prime) |
| Copb2 | S.i4vl7 | coatomer protein complex, subunit beta 2 (beta prime) |
| Coprs | S.i3vl7 | coordinator of PRMT5, differentiation stimulator |
| Coprs | S.i2vl7 | coordinator of PRMT5, differentiation stimulator |
| Coprs | S.i4vl7 | coordinator of PRMT5, differentiation stimulator |
| Coprs | S.c7 | coordinator of PRMT5, differentiation stimulator |
| Coprs | S.i1vl7 | coordinator of PRMT5, differentiation stimulator |
| Coprs | S.c8 | coordinator of PRMT5, differentiation stimulator |
| Cops7a | S.i4vl7 | COP9 (constitutive photomorphogenic) homolog, subunit 7a (Arabidopsis thaliana) |
| Cops7a | S.c8 | COP9 (constitutive photomorphogenic) homolog, subunit 7a (Arabidopsis thaliana) |
| Copz1 | S.c7 | coatomer protein complex, subunit zeta 1 |
| Copz1 | S.i2vl7 | coatomer protein complex, subunit zeta 1 |
| Copz1 | S.i3vl7 | coatomer protein complex, subunit zeta 1 |
| Copz1 | S.i1vl7 | coatomer protein complex, subunit zeta 1 |
| Coro1a | S.c2 | coronin, actin binding protein 1A |
| Coro1a | S.i3vl7 | coronin, actin binding protein 1A |
| Coro1a | S.i2vl7 | coronin, actin binding protein 1A |
| Coro1c | S.c2 | coronin, actin binding protein 1C |
| Coro1c | S.i4vl7 | coronin, actin binding protein 1C |
| Coro1c | S.i3vl7 | coronin, actin binding protein 1C |
| Cox6b2 | S.c8 | cytochrome c oxidase subunit VIb polypeptide 2 |
| Cox7a2 | S.i4vl7 | cytochrome c oxidase subunit VIIa 2 |
| Cox7a2 | S.c7 | cytochrome c oxidase subunit VIIa 2 |
| Cox7a2 | S.i3vl7 | cytochrome c oxidase subunit VIIa 2 |
| Cp | c5.i3vl7 | ceruloplasmin |
| Cp | S.i2vl7 | ceruloplasmin |
| Cp | S.i4vl7 | ceruloplasmin |
| Cp | S.i3vl7 | ceruloplasmin |
| Cp | S.i1vl7 | ceruloplasmin |
| Cpa3 | S.c1 | carboxypeptidase A3, mast cell |
| Cpa3 | S.i4vl7 | carboxypeptidase A3, mast cell |
| Cpd | S.c4 | carboxypeptidase D |
| Cpe | S.i4vl7 | carboxypeptidase E |
| Cpe | S.c1 | carboxypeptidase E |
| Cpn1 | S.c7 | carboxypeptidase N, polypeptide 1 |
| Cpt1a | S.i3vl7 | carnitine palmitoyltransferase 1a, liver |
| Cpt1a | c5.i3vl7 | carnitine palmitoyltransferase 1a, liver |
| Cpt1a | S.i4vl7 | carnitine palmitoyltransferase 1a, liver |
| Cpt1a | S.i1vl7 | carnitine palmitoyltransferase 1a, liver |
| Cpt2 | S.i2vl7 | carnitine palmitoyltransferase 2 |
| Cpt2 | S.i1vl7 | carnitine palmitoyltransferase 2 |
| Cpt2 | S.i4vl7 | carnitine palmitoyltransferase 2 |
| Cpt2 | S.c3 | carnitine palmitoyltransferase 2 |
| Cpxm1 | S.c1 | carboxypeptidase X 1 (M14 family) |
| Cr1l | S.i1vl7 | complement component (3b/4b) receptor 1-like |
| Crabp2 | S.c7 | cellular retinoic acid binding protein II |
| Crat | S.c2 | carnitine acetyltransferase |
| Crat | S.i4vl7 | carnitine acetyltransferase |
| Creb3l1 | S.c7 | cAMP responsive element binding protein 3-like 1 |
| Creg1 | S.c1 | cellular repressor of E1A-stimulated genes 1 |
| Creg1 | S.i4vl7 | cellular repressor of E1A-stimulated genes 1 |
| Creld2 | S.c2 | cysteine-rich with EGF-like domains 2 |
| Creld2 | S.i3vl7 | cysteine-rich with EGF-like domains 2 |
| Crip1 | S.i2vl7 | cysteine-rich protein 1 (intestinal) |
| Crip1 | S.i4vl7 | cysteine-rich protein 1 (intestinal) |
| Crip1 | S.c2 | cysteine-rich protein 1 (intestinal) |
| Crip2 | S.c4 | cysteine rich protein 2 |
| Crip2 | S.i2vl7 | cysteine rich protein 2 |
| Cript | S.i1vl7 | cysteine-rich PDZ-binding protein |
| Crlf3 | S.i2vl7 | cytokine receptor-like factor 3 |
| Crlf3 | S.c3 | cytokine receptor-like factor 3 |
| Crlf3 | S.i4vl7 | cytokine receptor-like factor 3 |
| Crtap | S.c3 | cartilage associated protein |
| Cry1 | S.c7 | cryptochrome 1 (photolyase-like) |
| Cryl1 | S.c7 | crystallin, lambda 1 |
| Cs | S.i4vl7 | citrate synthase |
| Cs | S.c7 | citrate synthase |
| Cs | S.i3vl7 | citrate synthase |
| Cs | S.i1vl7 | citrate synthase |
| Cs | S.i2vl7 | citrate synthase |
| Csad | S.i4vl7 | cysteine sulfinic acid decarboxylase |
| Csf1r | S.i4vl7 | colony stimulating factor 1 receptor |
| Csf1r | S.c2 | colony stimulating factor 1 receptor |
| Csf1r | S.i2vl7 | colony stimulating factor 1 receptor |
| Csf1r | S.c8 | colony stimulating factor 1 receptor |
| Csf1r | S.i3vl7 | colony stimulating factor 1 receptor |
| Csf2ra | S.c2 | colony stimulating factor 2 receptor, alpha, low-affinity (granulocyte-macrophage) |
| Csf2rb2 | S.i4vl7 | colony stimulating factor 2 receptor, beta 2, low-affinity (granulocyte-macrophage) |
| Csf2rb2 | S.c1 | colony stimulating factor 2 receptor, beta 2, low-affinity (granulocyte-macrophage) |
| Csnk1a1 | S.i2vl7 | casein kinase 1, alpha 1 |
| Csnk1e | S.c4 | casein kinase 1, epsilon |
| Csrp1 | S.i4vl7 | cysteine and glycine-rich protein 1 |
| Csrp1 | S.c3 | cysteine and glycine-rich protein 1 |
| Csrp1 | S.i1vl7 | cysteine and glycine-rich protein 1 |
| Csrp1 | S.i2vl7 | cysteine and glycine-rich protein 1 |
| Csrp1 | S.i3vl7 | cysteine and glycine-rich protein 1 |
| Cstb | S.c4 | cystatin B |
| Cstb | S.i1vl7 | cystatin B |
| Cstb | S.i3vl7 | cystatin B |
| Cstb | S.i4vl7 | cystatin B |
| Cstb | S.i2vl7 | cystatin B |
| Ctbp2 | S.i2vl7 | C-terminal binding protein 2 |
| Ctbp2 | S.i4vl7 | C-terminal binding protein 2 |
| Ctbp2 | c5.i3vl7 | C-terminal binding protein 2 |
| Ctbp2 | S.i1vl7 | C-terminal binding protein 2 |
| Ctbp2 | S.i3vl7 | C-terminal binding protein 2 |
| Ctgf | S.i2vl7 | connective tissue growth factor |
| Ctgf | S.c3 | connective tissue growth factor |
| Ctgf | S.i3vl7 | connective tissue growth factor |
| Ctgf | S.i4vl7 | connective tissue growth factor |
| Ctnna1 | S.i2vl7 | catenin (cadherin associated protein), alpha 1 |
| Ctnna1 | S.i3vl7 | catenin (cadherin associated protein), alpha 1 |
| Ctnna1 | S.c4 | catenin (cadherin associated protein), alpha 1 |
| Ctnna1 | S.i1vl7 | catenin (cadherin associated protein), alpha 1 |
| Ctnna1 | S.i4vl7 | catenin (cadherin associated protein), alpha 1 |
| Ctnnb1 | S.i3vl7 | catenin (cadherin associated protein), beta 1 |
| Ctnnb1 | S.c4 | catenin (cadherin associated protein), beta 1 |
| Ctnnb1 | S.i2vl7 | catenin (cadherin associated protein), beta 1 |
| Ctnnb1 | S.i1vl7 | catenin (cadherin associated protein), beta 1 |
| Ctnnb1 | S.i4vl7 | catenin (cadherin associated protein), beta 1 |
| Ctnnd1 | S.i1vl7 | catenin (cadherin associated protein), delta 1 |
| Ctps | S.c4 | cytidine 5'-triphosphate synthase |
| Ctps | S.i3vl7 | cytidine 5'-triphosphate synthase |
| Ctps | S.i2vl7 | cytidine 5'-triphosphate synthase |
| Ctps | S.i1vl7 | cytidine 5'-triphosphate synthase |
| Ctps | S.i4vl7 | cytidine 5'-triphosphate synthase |
| Ctr9 | S.i2vl7 | Ctr9, Paf1/RNA polymerase II complex component, homolog (S. cerevisiae) |
| Ctrl | S.c7 | chymotrypsin-like |
| Ctsa | S.i1vl7 | cathepsin A |
| Ctsa | c5.i3vl7 | cathepsin A |
| Ctsa | S.i2vl7 | cathepsin A |
| Ctsa | S.i4vl7 | cathepsin A |
| Ctsa | S.i3vl7 | cathepsin A |
| Ctsb | S.i2vl7 | cathepsin B |
| Ctsb | S.i1vl7 | cathepsin B |
| Ctsb | S.i3vl7 | cathepsin B |
| Ctsb | S.c4 | cathepsin B |
| Ctsb | S.i4vl7 | cathepsin B |
| Ctsb | S.c3 | cathepsin B |
| Ctsd | S.i3vl7 | cathepsin D |
| Ctsd | S.i4vl7 | cathepsin D |
| Ctsd | S.i2vl7 | cathepsin D |
| Ctse | S.c7 | cathepsin E |
| Ctse | S.i4vl7 | cathepsin E |
| Ctse | S.i3vl7 | cathepsin E |
| Ctse | S.i2vl7 | cathepsin E |
| Ctse | S.i1vl7 | cathepsin E |
| Ctsf | S.i2vl7 | cathepsin F |
| Ctsf | S.c2 | cathepsin F |
| Ctsf | S.i4vl7 | cathepsin F |
| Ctsk | S.c1 | cathepsin K |
| Ctsl | S.i1vl7 | cathepsin L |
| Ctsl | S.i2vl7 | cathepsin L |
| Ctsl | S.i3vl7 | cathepsin L |
| Ctsl | c5.i3vl7 | cathepsin L |
| Ctss | S.i2vl7 | cathepsin S |
| Ctss | S.i3vl7 | cathepsin S |
| Ctss | S.i4vl7 | cathepsin S |
| Ctss | S.c2 | cathepsin S |
| Ctsz | S.i4vl7 | cathepsin Z |
| Ctsz | S.c3 | cathepsin Z |
| Cttn | S.i4vl7 | cortactin |
| Cttn | c5.i3vl7 | cortactin |
| Cttn | S.i2vl7 | cortactin |
| Cttn | S.i1vl7 | cortactin |
| Cttn | S.i3vl7 | cortactin |
| Cuta | S.i1vl7 | cutA divalent cation tolerance homolog (E. coli) |
| Cuta | S.i3vl7 | cutA divalent cation tolerance homolog (E. coli) |
| Cuta | S.i2vl7 | cutA divalent cation tolerance homolog (E. coli) |
| Cuta | S.i4vl7 | cutA divalent cation tolerance homolog (E. coli) |
| Cuta | S.c7 | cutA divalent cation tolerance homolog (E. coli) |
| Cwc22 | S.c9 | CWC22 spliceosome-associated protein homolog (S. cerevisiae) |
| Cx3cl1 | S.i2vl7 | chemokine (C-X3-C motif) ligand 1 |
| Cx3cl1 | S.c2 | chemokine (C-X3-C motif) ligand 1 |
| Cx3cl1 | S.i4vl7 | chemokine (C-X3-C motif) ligand 1 |
| Cx3cl1 | S.i3vl7 | chemokine (C-X3-C motif) ligand 1 |
| Cxcl1 | S.c6 | chemokine (C-X-C motif) ligand 1 |
| Cxcl12 | S.i4vl7 | chemokine (C-X-C motif) ligand 12 |
| Cxcl12 | S.c1 | chemokine (C-X-C motif) ligand 12 |
| Cxcl14 | S.i4vl7 | chemokine (C-X-C motif) ligand 14 |
| Cxcl14 | S.i3vl7 | chemokine (C-X-C motif) ligand 14 |
| Cxcl14 | S.c3 | chemokine (C-X-C motif) ligand 14 |
| Cyb561 | S.c6 | cytochrome b-561 |
| Cyb561 | S.i1vl7 | cytochrome b-561 |
| Cyb5b | S.i2vl7 | cytochrome b5 type B |
| Cyb5b | S.c7 | cytochrome b5 type B |
| Cyb5b | S.i3vl7 | cytochrome b5 type B |
| Cyb5b | S.i1vl7 | cytochrome b5 type B |
| Cyb5r4 | S.c6 | cytochrome b5 reductase 4 |
| Cyba | S.i3vl7 | cytochrome b-245, alpha polypeptide |
| Cyba | S.i1vl7 | cytochrome b-245, alpha polypeptide |
| Cyba | S.c3 | cytochrome b-245, alpha polypeptide |
| Cyba | S.i2vl7 | cytochrome b-245, alpha polypeptide |
| Cyba | S.i4vl7 | cytochrome b-245, alpha polypeptide |
| Cyfip1 | S.c4 | cytoplasmic FMR1 interacting protein 1 |
| Cyfip1 | S.i1vl7 | cytoplasmic FMR1 interacting protein 1 |
| Cyfip1 | S.i3vl7 | cytoplasmic FMR1 interacting protein 1 |
| Cyfip1 | S.i4vl7 | cytoplasmic FMR1 interacting protein 1 |
| Cyfip1 | S.i2vl7 | cytoplasmic FMR1 interacting protein 1 |
| Cyhr1 | S.c7 | cysteine and histidine rich 1 |
| Cyhr1 | S.i3vl7 | cysteine and histidine rich 1 |
| Cyhr1 | S.i2vl7 | cysteine and histidine rich 1 |
| Cyhr1 | S.i1vl7 | cysteine and histidine rich 1 |
| Cyp1b1 | S.c1 | cytochrome P450, family 1, subfamily b, polypeptide 1 |
| Cyp1b1 | S.i4vl7 | cytochrome P450, family 1, subfamily b, polypeptide 1 |
| Cyp24a1 | S.i4vl7 | cytochrome P450, family 24, subfamily a, polypeptide 1 |
| Cyp24a1 | S.i2vl7 | cytochrome P450, family 24, subfamily a, polypeptide 1 |
| Cyp24a1 | S.c6 | cytochrome P450, family 24, subfamily a, polypeptide 1 |
| Cyp24a1 | S.i1vl7 | cytochrome P450, family 24, subfamily a, polypeptide 1 |
| Cyp2d10 | S.i2vl7 | cytochrome P450, family 2, subfamily d, polypeptide 10 |
| Cyp2d10 | S.c4 | cytochrome P450, family 2, subfamily d, polypeptide 10 |
| Cyp2d10 | S.i4vl7 | cytochrome P450, family 2, subfamily d, polypeptide 10 |
| Cyp2d12 | S.c3 | cytochrome P450, family 2, subfamily d, polypeptide 12 |
| Cyp2d12 | S.i2vl7 | cytochrome P450, family 2, subfamily d, polypeptide 12 |
| Cyp2d9 | S.c3 | cytochrome P450, family 2, subfamily d, polypeptide 9 |
| Cyp2d9 | S.i2vl7 | cytochrome P450, family 2, subfamily d, polypeptide 9 |
| Cyp2d9 | S.c7 | cytochrome P450, family 2, subfamily d, polypeptide 9 |
| Cyp2e1 | S.c1 | cytochrome P450, family 2, subfamily e, polypeptide 1 |
| Cyp4b1 | S.i4vl7 | cytochrome P450, family 4, subfamily b, polypeptide 1 |
| Cyp4b1 | S.c2 | cytochrome P450, family 4, subfamily b, polypeptide 1 |
| Cyp4v3 | S.i3vl7 | cytochrome P450, family 4, subfamily v, polypeptide 3 |
| Cyp4v3 | S.i4vl7 | cytochrome P450, family 4, subfamily v, polypeptide 3 |
| Cyp4v3 | S.c3 | cytochrome P450, family 4, subfamily v, polypeptide 3 |
| Cyp4v3 | S.i2vl7 | cytochrome P450, family 4, subfamily v, polypeptide 3 |
| Cyp51 | S.i2vl7 | cytochrome P450, family 51 |
| Cyp51 | S.c7 | cytochrome P450, family 51 |
| Cyp51 | S.i4vl7 | cytochrome P450, family 51 |
| Cyth2 | S.i1vl7 | cytohesin 2 |
| Cyth2 | S.c6 | cytohesin 2 |
| Cyth3 | S.i4vl7 | cytohesin 3 |
| Cyth3 | S.c2 | cytohesin 3 |
| Cyth3 | S.i3vl7 | cytohesin 3 |
| Cytip | S.c9 | cytohesin 1 interacting protein |
| Cytip | S.i4vl7 | cytohesin 1 interacting protein |
| Cytip | S.i2vl7 | cytohesin 1 interacting protein |
| D10Jhu81e | S.c7 | DNA segment, Chr 10, Johns Hopkins University 81 expressed |
| D15Ertd621e | S.i4vl7 | DNA segment, Chr 15, ERATO Doi 621, expressed |
| D15Ertd621e | S.c3 | DNA segment, Chr 15, ERATO Doi 621, expressed |
| D15Ertd621e | S.i1vl7 | DNA segment, Chr 15, ERATO Doi 621, expressed |
| D15Ertd621e | S.i3vl7 | DNA segment, Chr 15, ERATO Doi 621, expressed |
| D15Ertd621e | S.i2vl7 | DNA segment, Chr 15, ERATO Doi 621, expressed |
| D17Wsu104e | S.c7 | DNA segment, Chr 17, Wayne State University 104, expressed |
| D17Wsu104e | S.i4vl7 | DNA segment, Chr 17, Wayne State University 104, expressed |
| D17Wsu104e | S.i3vl7 | DNA segment, Chr 17, Wayne State University 104, expressed |
| D2Wsu81e | S.i3vl7 | DNA segment, Chr 2, Wayne State University 81, expressed |
| D2Wsu81e | S.c7 | DNA segment, Chr 2, Wayne State University 81, expressed |
| D2Wsu81e | S.i4vl7 | DNA segment, Chr 2, Wayne State University 81, expressed |
| D2Wsu81e | S.i2vl7 | DNA segment, Chr 2, Wayne State University 81, expressed |
| D4Wsu53e | S.i2vl7 | DNA segment, Chr 4, Wayne State University 53, expressed |
| D4Wsu53e | S.i3vl7 | DNA segment, Chr 4, Wayne State University 53, expressed |
| D4Wsu53e | c5.i3vl7 | DNA segment, Chr 4, Wayne State University 53, expressed |
| Dab2 | S.i2vl7 | disabled 2, mitogen-responsive phosphoprotein |
| Dab2 | S.c7 | disabled 2, mitogen-responsive phosphoprotein |
| Dab2 | S.i3vl7 | disabled 2, mitogen-responsive phosphoprotein |
| Dab2 | S.c8 | disabled 2, mitogen-responsive phosphoprotein |
| Dab2 | S.i4vl7 | disabled 2, mitogen-responsive phosphoprotein |
| Dad1 | S.c7 | defender against cell death 1 |
| Dad1 | S.i3vl7 | defender against cell death 1 |
| Dad1 | S.i4vl7 | defender against cell death 1 |
| Daf2 | S.i4vl7 | decay accelerating factor 2 |
| Daf2 | S.c9 | decay accelerating factor 2 |
| Dag1 | S.i3vl7 | dystroglycan 1 |
| Dag1 | S.i4vl7 | dystroglycan 1 |
| Dag1 | S.c3 | dystroglycan 1 |
| Dap | S.c8 | death-associated protein |
| Dap | S.i3vl7 | death-associated protein |
| Dap | S.i4vl7 | death-associated protein |
| Dap | S.i2vl7 | death-associated protein |
| Dap | S.i1vl7 | death-associated protein |
| Dazap2 | S.i1vl7 | DAZ associated protein 2 |
| Dazap2 | S.c4 | DAZ associated protein 2 |
| Dbt | S.i2vl7 | dihydrolipoamide branched chain transacylase E2 |
| Dbt | S.i3vl7 | dihydrolipoamide branched chain transacylase E2 |
| Dbt | S.i1vl7 | dihydrolipoamide branched chain transacylase E2 |
| Dbt | S.c7 | dihydrolipoamide branched chain transacylase E2 |
| Dcn | S.i2vl7 | decorin |
| Dcn | S.c2 | decorin |
| Dcn | S.i4vl7 | decorin |
| Dcn | S.i3vl7 | decorin |
| Dctn1 | S.c2 | dynactin 1 |
| Dctn2 | S.i1vl7 | dynactin 2 |
| Dctn2 | c5.i3vl7 | dynactin 2 |
| Dctn2 | S.i2vl7 | dynactin 2 |
| Dctn2 | S.i3vl7 | dynactin 2 |
| Ddah2 | S.i2vl7 | dimethylarginine dimethylaminohydrolase 2 |
| Ddah2 | S.i1vl7 | dimethylarginine dimethylaminohydrolase 2 |
| Ddah2 | S.c3 | dimethylarginine dimethylaminohydrolase 2 |
| Ddah2 | S.i3vl7 | dimethylarginine dimethylaminohydrolase 2 |
| Ddah2 | S.i4vl7 | dimethylarginine dimethylaminohydrolase 2 |
| Ddb1 | S.c4 | damage specific DNA binding protein 1 |
| Ddb1 | S.i2vl7 | damage specific DNA binding protein 1 |
| Ddb1 | S.i4vl7 | damage specific DNA binding protein 1 |
| Ddb1 | S.i1vl7 | damage specific DNA binding protein 1 |
| Ddb1 | c5.i3vl7 | damage specific DNA binding protein 1 |
| Ddb1 | S.i3vl7 | damage specific DNA binding protein 1 |
| Ddr1 | S.i1vl7 | discoidin domain receptor family, member 1 |
| Ddr1 | S.c6 | discoidin domain receptor family, member 1 |
| Ddr1 | S.i4vl7 | discoidin domain receptor family, member 1 |
| Ddx17 | S.c2 | DEAD (Asp-Glu-Ala-Asp) box polypeptide 17 |
| Ddx19a | S.i1vl7 | DEAD (Asp-Glu-Ala-Asp) box polypeptide 19a |
| Ddx19a | S.c4 | DEAD (Asp-Glu-Ala-Asp) box polypeptide 19a |
| Ddx19b | S.c8 | DEAD (Asp-Glu-Ala-Asp) box polypeptide 19b |
| Ddx39b | S.i4vl7 | DEAD (Asp-Glu-Ala-Asp) box polypeptide 39B |
| Ddx39b | S.c4 | DEAD (Asp-Glu-Ala-Asp) box polypeptide 39B |
| Ddx39b | S.i1vl7 | DEAD (Asp-Glu-Ala-Asp) box polypeptide 39B |
| Ddx39b | S.i2vl7 | DEAD (Asp-Glu-Ala-Asp) box polypeptide 39B |
| Ddx39b | S.i3vl7 | DEAD (Asp-Glu-Ala-Asp) box polypeptide 39B |
| Decr1 | S.c2 | 2,4-dienoyl CoA reductase 1, mitochondrial |
| Defb1 | S.c6 | defensin beta 1 |
| Degs1 | S.i4vl7 | degenerative spermatocyte homolog 1 (Drosophila) |
| Degs1 | S.c1 | degenerative spermatocyte homolog 1 (Drosophila) |
| Dennd5a | S.i3vl7 | DENN/MADD domain containing 5A |
| Dennd5a | S.i4vl7 | DENN/MADD domain containing 5A |
| Dennd5a | S.i1vl7 | DENN/MADD domain containing 5A |
| Dennd5a | S.i2vl7 | DENN/MADD domain containing 5A |
| Dennd5a | S.c3 | DENN/MADD domain containing 5A |
| Derl2 | S.c8 | Der1-like domain family, member 2 |
| Derl2 | S.i4vl7 | Der1-like domain family, member 2 |
| Dguok | S.c3 | deoxyguanosine kinase |
| Dguok | S.i3vl7 | deoxyguanosine kinase |
| Dguok | S.i4vl7 | deoxyguanosine kinase |
| Dhcr7 | S.c7 | 7-dehydrocholesterol reductase |
| Dhodh | S.c7 | dihydroorotate dehydrogenase |
| Dhrs1 | S.i3vl7 | dehydrogenase/reductase (SDR family) member 1 |
| Dhrs1 | S.c7 | dehydrogenase/reductase (SDR family) member 1 |
| Dhrs1 | S.i1vl7 | dehydrogenase/reductase (SDR family) member 1 |
| Dhrs1 | S.i2vl7 | dehydrogenase/reductase (SDR family) member 1 |
| Dhrs3 | S.i2vl7 | dehydrogenase/reductase (SDR family) member 3 |
| Dhrs3 | S.i3vl7 | dehydrogenase/reductase (SDR family) member 3 |
| Dhrs3 | S.c2 | dehydrogenase/reductase (SDR family) member 3 |
| Dhrs3 | S.i4vl7 | dehydrogenase/reductase (SDR family) member 3 |
| Dhrs7 | S.c2 | dehydrogenase/reductase (SDR family) member 7 |
| Dhx40 | S.i4vl7 | DEAH (Asp-Glu-Ala-His) box polypeptide 40 |
| Dhx40 | S.i2vl7 | DEAH (Asp-Glu-Ala-His) box polypeptide 40 |
| Dhx9 | S.i4vl7 | DEAH (Asp-Glu-Ala-His) box polypeptide 9 |
| Dhx9 | S.c3 | DEAH (Asp-Glu-Ala-His) box polypeptide 9 |
| Diap1 | S.c3 | diaphanous homolog 1 (Drosophila) |
| Diap1 | S.i3vl7 | diaphanous homolog 1 (Drosophila) |
| Diap1 | S.i1vl7 | diaphanous homolog 1 (Drosophila) |
| Diap1 | S.c2 | diaphanous homolog 1 (Drosophila) |
| Diap1 | S.i4vl7 | diaphanous homolog 1 (Drosophila) |
| Diap1 | S.i2vl7 | diaphanous homolog 1 (Drosophila) |
| Dlat | S.c7 | dihydrolipoamide S-acetyltransferase (E2 component of pyruvate dehydrogenase complex) |
| Dlat | S.i1vl7 | dihydrolipoamide S-acetyltransferase (E2 component of pyruvate dehydrogenase complex) |
| Dlat | S.i3vl7 | dihydrolipoamide S-acetyltransferase (E2 component of pyruvate dehydrogenase complex) |
| Dlat | S.i4vl7 | dihydrolipoamide S-acetyltransferase (E2 component of pyruvate dehydrogenase complex) |
| Dlst | S.c3 | dihydrolipoamide S-succinyltransferase (E2 component of 2-oxo-glutarate complex) |
| Dlst | S.i4vl7 | dihydrolipoamide S-succinyltransferase (E2 component of 2-oxo-glutarate complex) |
| Dmwd | S.c7 | dystrophia myotonica-containing WD repeat motif |
| Dmwd | S.i1vl7 | dystrophia myotonica-containing WD repeat motif |
| Dmwd | S.i4vl7 | dystrophia myotonica-containing WD repeat motif |
| Dmwd | S.i2vl7 | dystrophia myotonica-containing WD repeat motif |
| Dnaja1 | S.c4 | DnaJ (Hsp40) homolog, subfamily A, member 1 |
| Dnajb11 | S.c2 | DnaJ (Hsp40) homolog, subfamily B, member 11 |
| Dnajb11 | S.i3vl7 | DnaJ (Hsp40) homolog, subfamily B, member 11 |
| Dnajb11 | S.i4vl7 | DnaJ (Hsp40) homolog, subfamily B, member 11 |
| Dnajb13 | S.c8 | DnaJ (Hsp40) related, subfamily B, member 13 |
| Dnajb13 | S.i4vl7 | DnaJ (Hsp40) related, subfamily B, member 13 |
| Dnajc10 | S.c4 | DnaJ (Hsp40) homolog, subfamily C, member 10 |
| Dnajc10 | S.i1vl7 | DnaJ (Hsp40) homolog, subfamily C, member 10 |
| Dnajc10 | S.i4vl7 | DnaJ (Hsp40) homolog, subfamily C, member 10 |
| Dnajc3 | S.c2 | DnaJ (Hsp40) homolog, subfamily C, member 3 |
| Dnajc8 | S.c4 | DnaJ (Hsp40) homolog, subfamily C, member 8 |
| Dnase1l1 | S.c4 | deoxyribonuclease 1-like 1 |
| Dnm1 | S.c1 | dynamin 1 |
| Dnmt1 | S.i1vl7 | DNA methyltransferase (cytosine-5) 1 |
| Dnmt1 | S.c7 | DNA methyltransferase (cytosine-5) 1 |
| Dnmt1 | S.i2vl7 | DNA methyltransferase (cytosine-5) 1 |
| Dnmt1 | S.i4vl7 | DNA methyltransferase (cytosine-5) 1 |
| Dock9 | S.i4vl7 | dedicator of cytokinesis 9 |
| Dock9 | S.i3vl7 | dedicator of cytokinesis 9 |
| Dock9 | S.i1vl7 | dedicator of cytokinesis 9 |
| Dock9 | S.c4 | dedicator of cytokinesis 9 |
| Dock9 | S.i2vl7 | dedicator of cytokinesis 9 |
| Dohh | S.i4vl7 | deoxyhypusine hydroxylase/monooxygenase |
| Dpagt1 | S.i2vl7 | dolichyl-phosphate (UDP-N-acetylglucosamine) acetylglucosaminephosphotransferase 1 (GlcNAc-1-P transferase) |
| Dpep3 | S.c8 | dipeptidase 3 |
| Dpt | S.i4vl7 | dermatopontin |
| Dpt | S.c2 | dermatopontin |
| Dpt | S.i3vl7 | dermatopontin |
| Dpt | S.i2vl7 | dermatopontin |
| Drc1 | S.c9 | dynein regulatory complex subunit 1 |
| Drd4 | S.c7 | dopamine receptor D4 |
| Drg1 | S.c4 | developmentally regulated GTP binding protein 1 |
| Dscr3 | S.i4vl7 | Down syndrome critical region gene 3 |
| Dscr3 | S.i1vl7 | Down syndrome critical region gene 3 |
| Dscr3 | S.c3 | Down syndrome critical region gene 3 |
| Dscr3 | S.i3vl7 | Down syndrome critical region gene 3 |
| Dscr3 | S.i2vl7 | Down syndrome critical region gene 3 |
| Dsp | S.c1 | desmoplakin |
| Dsp | S.i4vl7 | desmoplakin |
| Dstn | S.i2vl7 | destrin |
| Dstn | S.i4vl7 | destrin |
| Dstn | S.c3 | destrin |
| Dusp16 | S.i3vl7 | dual specificity phosphatase 16 |
| Dusp16 | S.c6 | dual specificity phosphatase 16 |
| Dusp16 | S.i2vl7 | dual specificity phosphatase 16 |
| Dusp6 | S.i4vl7 | dual specificity phosphatase 6 |
| Dusp6 | S.c3 | dual specificity phosphatase 6 |
| Dusp6 | S.i3vl7 | dual specificity phosphatase 6 |
| Dvl2 | S.c8 | dishevelled 2, dsh homolog (Drosophila) |
| Dvl2 | S.i4vl7 | dishevelled 2, dsh homolog (Drosophila) |
| Dync1h1 | S.i4vl7 | dynein cytoplasmic 1 heavy chain 1 |
| Dync1h1 | c5.i3vl7 | dynein cytoplasmic 1 heavy chain 1 |
| Dync1h1 | S.i3vl7 | dynein cytoplasmic 1 heavy chain 1 |
| Dync1h1 | S.i2vl7 | dynein cytoplasmic 1 heavy chain 1 |
| Dync1i2 | S.i2vl7 | dynein cytoplasmic 1 intermediate chain 2 |
| Dync1i2 | S.i1vl7 | dynein cytoplasmic 1 intermediate chain 2 |
| Dync1i2 | S.i4vl7 | dynein cytoplasmic 1 intermediate chain 2 |
| Dync1i2 | S.c4 | dynein cytoplasmic 1 intermediate chain 2 |
| Dynlt1a | S.c3 | dynein light chain Tctex-type 1A |
| Dynlt1a | S.i2vl7 | dynein light chain Tctex-type 1A |
| Dynlt1a | S.i4vl7 | dynein light chain Tctex-type 1A |
| Dynlt1a | S.i3vl7 | dynein light chain Tctex-type 1A |
| Dynlt3 | S.i4vl7 | dynein light chain Tctex-type 3 |
| E130012A19Rik | S.i1vl7 | RIKEN cDNA E130012A19 gene |
| E130012A19Rik | S.i4vl7 | RIKEN cDNA E130012A19 gene |
| E130012A19Rik | S.i2vl7 | RIKEN cDNA E130012A19 gene |
| E130012A19Rik | S.c8 | RIKEN cDNA E130012A19 gene |
| Ear1 | S.c1 | eosinophil-associated, ribonuclease A family, member 1 |
| Ear12 | S.c1 | eosinophil-associated, ribonuclease A family, member 12 |
| Ear12 | S.i4vl7 | eosinophil-associated, ribonuclease A family, member 12 |
| Ear2 | S.c1 | eosinophil-associated, ribonuclease A family, member 2 |
| Ear2 | S.i4vl7 | eosinophil-associated, ribonuclease A family, member 2 |
| Ear3 | S.c1 | eosinophil-associated, ribonuclease A family, member 3 |
| Ear3 | S.i4vl7 | eosinophil-associated, ribonuclease A family, member 3 |
| Ech1 | S.i4vl7 | enoyl coenzyme A hydratase 1, peroxisomal |
| Ech1 | S.i2vl7 | enoyl coenzyme A hydratase 1, peroxisomal |
| Ech1 | S.i3vl7 | enoyl coenzyme A hydratase 1, peroxisomal |
| Ech1 | c5.i3vl7 | enoyl coenzyme A hydratase 1, peroxisomal |
| Ech1 | S.i1vl7 | enoyl coenzyme A hydratase 1, peroxisomal |
| Eci2 | S.i3vl7 | enoyl-Coenzyme A delta isomerase 2 |
| Eci2 | S.i2vl7 | enoyl-Coenzyme A delta isomerase 2 |
| Eci2 | S.i4vl7 | enoyl-Coenzyme A delta isomerase 2 |
| Eci2 | S.i1vl7 | enoyl-Coenzyme A delta isomerase 2 |
| Eci2 | c5.i3vl7 | enoyl-Coenzyme A delta isomerase 2 |
| Edem2 | S.i1vl7 | ER degradation enhancer, mannosidase alpha-like 2 |
| Edem2 | S.c7 | ER degradation enhancer, mannosidase alpha-like 2 |
| Edem2 | S.i4vl7 | ER degradation enhancer, mannosidase alpha-like 2 |
| Edem3 | S.c7 | ER degradation enhancer, mannosidase alpha-like 3 |
| Edem3 | S.i4vl7 | ER degradation enhancer, mannosidase alpha-like 3 |
| Eef2k | S.c7 | eukaryotic elongation factor-2 kinase |
| Efhd1 | S.c8 | EF hand domain containing 1 |
| Efna1 | S.i2vl7 | ephrin A1 |
| Efnb2 | S.c6 | ephrin B2 |
| Egf | S.i1vl7 | epidermal growth factor |
| Egf | S.i4vl7 | epidermal growth factor |
| Egf | S.i2vl7 | epidermal growth factor |
| Egf | S.i3vl7 | epidermal growth factor |
| Egf | S.c8 | epidermal growth factor |
| Egfr | S.i3vl7 | epidermal growth factor receptor |
| Egln1 | S.c3 | EGL nine homolog 1 (C. elegans) |
| Egr1 | S.c2 | early growth response 1 |
| Egr1 | S.i4vl7 | early growth response 1 |
| Egr1 | S.i3vl7 | early growth response 1 |
| Egr2 | S.i4vl7 | early growth response 2 |
| Ehhadh | S.c8 | enoyl-Coenzyme A, hydratase/3-hydroxyacyl Coenzyme A dehydrogenase |
| Ehmt2 | c5.i3vl7 | euchromatic histone lysine N-methyltransferase 2 |
| Ehmt2 | S.i3vl7 | euchromatic histone lysine N-methyltransferase 2 |
| Ehmt2 | S.i2vl7 | euchromatic histone lysine N-methyltransferase 2 |
| Eif1b | S.i1vl7 | eukaryotic translation initiation factor 1B |
| Eif1b | S.c4 | eukaryotic translation initiation factor 1B |
| Eif2b4 | S.c9 | eukaryotic translation initiation factor 2B, subunit 4 delta |
| Eif2b4 | S.i3vl7 | eukaryotic translation initiation factor 2B, subunit 4 delta |
| Eif2b4 | S.i4vl7 | eukaryotic translation initiation factor 2B, subunit 4 delta |
| Eif3e | S.c4 | eukaryotic translation initiation factor 3, subunit E |
| Eif4ebp1 | S.i3vl7 | eukaryotic translation initiation factor 4E binding protein 1 |
| Eif4ebp1 | S.c4 | eukaryotic translation initiation factor 4E binding protein 1 |
| Eif4ebp1 | S.i4vl7 | eukaryotic translation initiation factor 4E binding protein 1 |
| Eif4g1 | S.c8 | eukaryotic translation initiation factor 4, gamma 1 |
| Eif4g1 | S.i4vl7 | eukaryotic translation initiation factor 4, gamma 1 |
| Elf3 | c5.i3vl7 | E74-like factor 3 |
| Elf3 | S.i3vl7 | E74-like factor 3 |
| Elf5 | S.i4vl7 | E74-like factor 5 |
| Elf5 | S.c9 | E74-like factor 5 |
| Ell | S.c3 | elongation factor RNA polymerase II |
| Ell2 | S.i4vl7 | elongation factor RNA polymerase II 2 |
| Ell2 | S.i2vl7 | elongation factor RNA polymerase II 2 |
| Ell2 | S.i1vl7 | elongation factor RNA polymerase II 2 |
| Ell2 | S.c8 | elongation factor RNA polymerase II 2 |
| Ell2 | S.i3vl7 | elongation factor RNA polymerase II 2 |
| Ell2 | S.c7 | elongation factor RNA polymerase II 2 |
| Eln | S.c3 | elastin |
| Elovl1 | S.c7 | elongation of very long chain fatty acids (FEN1/Elo2, SUR4/Elo3, yeast)-like 1 |
| Elovl1 | S.i4vl7 | elongation of very long chain fatty acids (FEN1/Elo2, SUR4/Elo3, yeast)-like 1 |
| Elovl1 | S.i1vl7 | elongation of very long chain fatty acids (FEN1/Elo2, SUR4/Elo3, yeast)-like 1 |
| Elovl5 | S.i4vl7 | ELOVL family member 5, elongation of long chain fatty acids (yeast) |
| Elovl5 | S.c7 | ELOVL family member 5, elongation of long chain fatty acids (yeast) |
| Elovl5 | S.i2vl7 | ELOVL family member 5, elongation of long chain fatty acids (yeast) |
| Elovl5 | S.i3vl7 | ELOVL family member 5, elongation of long chain fatty acids (yeast) |
| Elovl5 | S.i1vl7 | ELOVL family member 5, elongation of long chain fatty acids (yeast) |
| Elovl6 | S.c7 | ELOVL family member 6, elongation of long chain fatty acids (yeast) |
| Emcn | S.c1 | endomucin |
| Emp1 | S.i1vl7 | epithelial membrane protein 1 |
| Emp1 | S.i2vl7 | epithelial membrane protein 1 |
| Emp1 | S.c3 | epithelial membrane protein 1 |
| Emp1 | S.i3vl7 | epithelial membrane protein 1 |
| Emp1 | S.i4vl7 | epithelial membrane protein 1 |
| Emp3 | S.i4vl7 | epithelial membrane protein 3 |
| Emp3 | S.c3 | epithelial membrane protein 3 |
| Emp3 | S.i3vl7 | epithelial membrane protein 3 |
| Emp3 | S.i2vl7 | epithelial membrane protein 3 |
| Emp3 | S.c7 | epithelial membrane protein 3 |
| Eng | S.c2 | endoglin |
| Enpep | S.i4vl7 | glutamyl aminopeptidase |
| Enpep | S.c3 | glutamyl aminopeptidase |
| Enpp2 | S.c1 | ectonucleotide pyrophosphatase/phosphodiesterase 2 |
| Enpp2 | S.i4vl7 | ectonucleotide pyrophosphatase/phosphodiesterase 2 |
| Epb4.1 | S.i4vl7 | erythrocyte protein band 4.1 |
| Epcam | S.i1vl7 | epithelial cell adhesion molecule |
| Epcam | S.c6 | epithelial cell adhesion molecule |
| Epcam | S.i2vl7 | epithelial cell adhesion molecule |
| Epcam | S.i3vl7 | epithelial cell adhesion molecule |
| Ephb6 | S.c8 | Eph receptor B6 |
| Ephx1 | S.i3vl7 | epoxide hydrolase 1, microsomal |
| Ephx1 | c5.i3vl7 | epoxide hydrolase 1, microsomal |
| Ephx1 | S.i4vl7 | epoxide hydrolase 1, microsomal |
| Ephx1 | S.i1vl7 | epoxide hydrolase 1, microsomal |
| Ephx1 | S.i2vl7 | epoxide hydrolase 1, microsomal |
| Ephx2 | S.c2 | epoxide hydrolase 2, cytoplasmic |
| Ephx2 | S.i4vl7 | epoxide hydrolase 2, cytoplasmic |
| Eps15 | S.i2vl7 | epidermal growth factor receptor pathway substrate 15 |
| Eps15 | S.c3 | epidermal growth factor receptor pathway substrate 15 |
| Eps15 | S.i1vl7 | epidermal growth factor receptor pathway substrate 15 |
| Eps15 | S.i4vl7 | epidermal growth factor receptor pathway substrate 15 |
| Eps15 | S.i3vl7 | epidermal growth factor receptor pathway substrate 15 |
| Ergic1 | S.c7 | endoplasmic reticulum-golgi intermediate compartment (ERGIC) 1 |
| Erh | S.i1vl7 | enhancer of rudimentary homolog (Drosophila) |
| Ermp1 | S.i4vl7 | endoplasmic reticulum metallopeptidase 1 |
| Ermp1 | S.i3vl7 | endoplasmic reticulum metallopeptidase 1 |
| Ermp1 | S.c3 | endoplasmic reticulum metallopeptidase 1 |
| Errfi1 | S.c3 | ERBB receptor feedback inhibitor 1 |
| Errfi1 | S.i3vl7 | ERBB receptor feedback inhibitor 1 |
| Errfi1 | S.c2 | ERBB receptor feedback inhibitor 1 |
| Esd | S.c4 | esterase D/formylglutathione hydrolase |
| Esyt1 | S.i2vl7 | extended synaptotagmin-like protein 1 |
| Esyt1 | S.c4 | extended synaptotagmin-like protein 1 |
| Esyt1 | S.i3vl7 | extended synaptotagmin-like protein 1 |
| Esyt1 | S.i4vl7 | extended synaptotagmin-like protein 1 |
| Esyt1 | S.i1vl7 | extended synaptotagmin-like protein 1 |
| Etfb | S.c3 | electron transferring flavoprotein, beta polypeptide |
| Etfb | S.i4vl7 | electron transferring flavoprotein, beta polypeptide |
| Etfb | S.i2vl7 | electron transferring flavoprotein, beta polypeptide |
| Etfb | S.i3vl7 | electron transferring flavoprotein, beta polypeptide |
| Etfdh | S.i2vl7 | electron transferring flavoprotein, dehydrogenase |
| Etfdh | S.i4vl7 | electron transferring flavoprotein, dehydrogenase |
| Etfdh | S.c4 | electron transferring flavoprotein, dehydrogenase |
| Ethe1 | S.c4 | ethylmalonic encephalopathy 1 |
| Ets2 | S.c3 | E26 avian leukemia oncogene 2, 3' domain |
| Ets2 | S.i2vl7 | E26 avian leukemia oncogene 2, 3' domain |
| Ets2 | S.i3vl7 | E26 avian leukemia oncogene 2, 3' domain |
| Ets2 | S.i4vl7 | E26 avian leukemia oncogene 2, 3' domain |
| Etv4 | S.c8 | ets variant gene 4 (E1A enhancer binding protein, E1AF) |
| Etv4 | S.i3vl7 | ets variant gene 4 (E1A enhancer binding protein, E1AF) |
| Etv4 | S.i4vl7 | ets variant gene 4 (E1A enhancer binding protein, E1AF) |
| Evi2a | S.i4vl7 | ecotropic viral integration site 2a |
| Evi2a | S.c1 | ecotropic viral integration site 2a |
| Evl | S.c1 | Ena-vasodilator stimulated phosphoprotein |
| Evl | S.i4vl7 | Ena-vasodilator stimulated phosphoprotein |
| Extl3 | S.i4vl7 | exostoses (multiple)-like 3 |
| Ezh1 | S.c4 | enhancer of zeste homolog 1 (Drosophila) |
| Ezr | S.c4 | ezrin |
| Ezr | S.i2vl7 | ezrin |
| Ezr | S.i1vl7 | ezrin |
| Ezr | S.i3vl7 | ezrin |
| F11r | S.c6 | F11 receptor |
| F11r | S.i2vl7 | F11 receptor |
| F11r | S.i3vl7 | F11 receptor |
| F11r | S.i1vl7 | F11 receptor |
| F2r | S.i1vl7 | coagulation factor II (thrombin) receptor |
| F2r | S.i4vl7 | coagulation factor II (thrombin) receptor |
| F2rl1 | S.i1vl7 | coagulation factor II (thrombin) receptor-like 1 |
| F2rl1 | S.c6 | coagulation factor II (thrombin) receptor-like 1 |
| F3 | S.i4vl7 | coagulation factor III |
| F3 | S.c4 | coagulation factor III |
| F3 | S.i2vl7 | coagulation factor III |
| Fabp3 | S.i2vl7 | fatty acid binding protein 3, muscle and heart |
| Fabp3 | S.i4vl7 | fatty acid binding protein 3, muscle and heart |
| Fabp3 | S.c9 | fatty acid binding protein 3, muscle and heart |
| Fabp3 | S.i1vl7 | fatty acid binding protein 3, muscle and heart |
| Fads1 | S.i2vl7 | fatty acid desaturase 1 |
| Fads1 | S.c7 | fatty acid desaturase 1 |
| Fads1 | S.i3vl7 | fatty acid desaturase 1 |
| Fads1 | S.i1vl7 | fatty acid desaturase 1 |
| Fads1 | S.i4vl7 | fatty acid desaturase 1 |
| Fah | S.i1vl7 | fumarylacetoacetate hydrolase |
| Fah | S.i2vl7 | fumarylacetoacetate hydrolase |
| Fah | S.i4vl7 | fumarylacetoacetate hydrolase |
| Fah | S.c7 | fumarylacetoacetate hydrolase |
| Fah | S.i3vl7 | fumarylacetoacetate hydrolase |
| Fahd1 | S.i3vl7 | fumarylacetoacetate hydrolase domain containing 1 |
| Fahd1 | S.c8 | fumarylacetoacetate hydrolase domain containing 1 |
| Fam101b | S.c3 | family with sequence similarity 101, member B |
| Fam101b | S.i4vl7 | family with sequence similarity 101, member B |
| Fam102a | S.c4 | family with sequence similarity 102, member A |
| Fam102a | S.i1vl7 | family with sequence similarity 102, member A |
| Fam105a | S.c1 | family with sequence similarity 105, member A |
| Fam107b | S.c4 | family with sequence similarity 107, member B |
| Fam110a | S.i3vl7 | family with sequence similarity 110, member A |
| Fam110a | S.i2vl7 | family with sequence similarity 110, member A |
| Fam110a | S.c7 | family with sequence similarity 110, member A |
| Fam110a | S.i4vl7 | family with sequence similarity 110, member A |
| Fam110a | S.i1vl7 | family with sequence similarity 110, member A |
| Fam132a | S.c1 | family with sequence similarity 132, member A |
| Fam132a | S.i1vl7 | family with sequence similarity 132, member A |
| Fam134b | S.i3vl7 | family with sequence similarity 134, member B |
| Fam134b | S.i1vl7 | family with sequence similarity 134, member B |
| Fam134b | S.c8 | family with sequence similarity 134, member B |
| Fam134b | S.i2vl7 | family with sequence similarity 134, member B |
| Fam134b | S.i4vl7 | family with sequence similarity 134, member B |
| Fam13b | S.i4vl7 | family with sequence similarity 13, member B |
| Fam13b | S.c3 | family with sequence similarity 13, member B |
| Fam171a1 | S.c3 | family with sequence similarity 171, member A1 |
| Fam195b | c5.i3vl7 | family with sequence similarity 195, member B |
| Fam195b | S.i4vl7 | family with sequence similarity 195, member B |
| Fam195b | S.i3vl7 | family with sequence similarity 195, member B |
| Fam195b | S.i2vl7 | family with sequence similarity 195, member B |
| Fam195b | S.i1vl7 | family with sequence similarity 195, member B |
| Fam20c | S.i4vl7 | family with sequence similarity 20, member C |
| Fam20c | S.c9 | family with sequence similarity 20, member C |
| Fam21 | S.i3vl7 | family with sequence similarity 21 |
| Fam21 | S.i1vl7 | family with sequence similarity 21 |
| Fam21 | S.i2vl7 | family with sequence similarity 21 |
| Fam21 | S.c4 | family with sequence similarity 21 |
| Fam21 | S.i4vl7 | family with sequence similarity 21 |
| Fam213a | S.c7 | family with sequence similarity 213, member A |
| Fam3c | S.i3vl7 | family with sequence similarity 3, member C |
| Fam3c | S.c3 | family with sequence similarity 3, member C |
| Fam3c | S.i4vl7 | family with sequence similarity 3, member C |
| Fam3c | S.i2vl7 | family with sequence similarity 3, member C |
| Fam46c | S.i2vl7 | family with sequence similarity 46, member C |
| Fam46c | S.i3vl7 | family with sequence similarity 46, member C |
| Fam46c | S.c7 | family with sequence similarity 46, member C |
| Fam46c | S.i1vl7 | family with sequence similarity 46, member C |
| Fam46c | S.i4vl7 | family with sequence similarity 46, member C |
| Fam49b | S.i1vl7 | family with sequence similarity 49, member B |
| Fam49b | S.i4vl7 | family with sequence similarity 49, member B |
| Fam49b | S.c4 | family with sequence similarity 49, member B |
| Fam76b | S.i4vl7 | family with sequence similarity 76, member B |
| Fam76b | S.c3 | family with sequence similarity 76, member B |
| Fam76b | S.i3vl7 | family with sequence similarity 76, member B |
| Fam83h | S.i1vl7 | family with sequence similarity 83, member H |
| Fam83h | S.c6 | family with sequence similarity 83, member H |
| Fam89b | S.i1vl7 | family with sequence similarity 89, member B |
| Fam89b | S.c4 | family with sequence similarity 89, member B |
| Fap | S.c2 | fibroblast activation protein |
| Farsa | S.i1vl7 | phenylalanyl-tRNA synthetase, alpha subunit |
| Fasn | S.c7 | fatty acid synthase |
| Fasn | S.i1vl7 | fatty acid synthase |
| Fbln1 | S.c1 | fibulin 1 |
| Fbln2 | S.i4vl7 | fibulin 2 |
| Fbln2 | S.c2 | fibulin 2 |
| Fbn1 | S.i4vl7 | fibrillin 1 |
| Fbn1 | S.c3 | fibrillin 1 |
| Fbn1 | S.i3vl7 | fibrillin 1 |
| Fbn1 | S.i2vl7 | fibrillin 1 |
| Fbxo3 | S.c6 | F-box protein 3 |
| Fbxo38 | S.c4 | F-box protein 38 |
| Fbxo38 | S.i4vl7 | F-box protein 38 |
| Fbxo38 | S.i1vl7 | F-box protein 38 |
| Fbxo38 | S.i3vl7 | F-box protein 38 |
| Fbxo6 | S.i2vl7 | F-box protein 6 |
| Fbxo6 | S.i1vl7 | F-box protein 6 |
| Fbxw2 | S.i1vl7 | F-box and WD-40 domain protein 2 |
| Fbxw2 | c5.i3vl7 | F-box and WD-40 domain protein 2 |
| Fbxw2 | S.i4vl7 | F-box and WD-40 domain protein 2 |
| Fbxw2 | S.i2vl7 | F-box and WD-40 domain protein 2 |
| Fbxw2 | S.i3vl7 | F-box and WD-40 domain protein 2 |
| Fbxw4 | S.c6 | F-box and WD-40 domain protein 4 |
| Fbxw4 | S.i1vl7 | F-box and WD-40 domain protein 4 |
| Fbxw7 | S.i2vl7 | F-box and WD-40 domain protein 7 |
| Fcgbp | S.c2 | Fc fragment of IgG binding protein |
| Fcgbp | S.c1 | Fc fragment of IgG binding protein |
| Fcgbp | S.i4vl7 | Fc fragment of IgG binding protein |
| Fcgbp | S.i3vl7 | Fc fragment of IgG binding protein |
| Fcgr1 | S.i4vl7 | Fc receptor, IgG, high affinity I |
| Fcgr1 | S.c1 | Fc receptor, IgG, high affinity I |
| Fcgr2b | S.i4vl7 | Fc receptor, IgG, low affinity IIb |
| Fcgr2b | S.c1 | Fc receptor, IgG, low affinity IIb |
| Fdft1 | S.c9 | farnesyl diphosphate farnesyl transferase 1 |
| Fdps | S.i2vl7 | farnesyl diphosphate synthetase |
| Fdps | S.c7 | farnesyl diphosphate synthetase |
| Fdps | S.i1vl7 | farnesyl diphosphate synthetase |
| Fdps | S.c8 | farnesyl diphosphate synthetase |
| Fdps | S.i3vl7 | farnesyl diphosphate synthetase |
| Fdx1 | S.i3vl7 | ferredoxin 1 |
| Fdx1 | S.i4vl7 | ferredoxin 1 |
| Fdx1 | S.i2vl7 | ferredoxin 1 |
| Fdx1 | S.c7 | ferredoxin 1 |
| Fen1 | S.c7 | flap structure specific endonuclease 1 |
| Fermt3 | S.c1 | fermitin family homolog 3 (Drosophila) |
| Fgf11 | S.c7 | fibroblast growth factor 11 |
| Fgfr2 | S.i4vl7 | fibroblast growth factor receptor 2 |
| Fgl2 | S.c1 | fibrinogen-like protein 2 |
| Fgl2 | S.i4vl7 | fibrinogen-like protein 2 |
| Fhl2 | S.c1 | four and a half LIM domains 2 |
| Fibp | S.i1vl7 | fibroblast growth factor (acidic) intracellular binding protein |
| Fignl1 | S.c7 | fidgetin-like 1 |
| Fignl1 | S.i3vl7 | fidgetin-like 1 |
| Fignl1 | S.i4vl7 | fidgetin-like 1 |
| Fkbp11 | S.i1vl7 | FK506 binding protein 11 |
| Fkbp11 | S.i4vl7 | FK506 binding protein 11 |
| Fkbp11 | S.c8 | FK506 binding protein 11 |
| Fkbp1a | S.i2vl7 | FK506 binding protein 1a |
| Fkbp1a | S.i4vl7 | FK506 binding protein 1a |
| Fkbp1a | S.i1vl7 | FK506 binding protein 1a |
| Fkbp1a | S.c4 | FK506 binding protein 1a |
| Fkbp5 | S.c7 | FK506 binding protein 5 |
| Fkbp7 | S.c3 | FK506 binding protein 7 |
| Fkbp9 | S.i3vl7 | FK506 binding protein 9 |
| Fkbp9 | S.c3 | FK506 binding protein 9 |
| Fkbp9 | S.i2vl7 | FK506 binding protein 9 |
| Fkbp9 | S.i4vl7 | FK506 binding protein 9 |
| Flii | S.i3vl7 | flightless I homolog (Drosophila) |
| Flii | S.c4 | flightless I homolog (Drosophila) |
| Flii | S.i4vl7 | flightless I homolog (Drosophila) |
| Flii | S.i2vl7 | flightless I homolog (Drosophila) |
| Flii | S.c3 | flightless I homolog (Drosophila) |
| Flii | S.i1vl7 | flightless I homolog (Drosophila) |
| Flot1 | S.i1vl7 | flotillin 1 |
| Flot1 | S.i4vl7 | flotillin 1 |
| Flot1 | S.c4 | flotillin 1 |
| Flot1 | S.i2vl7 | flotillin 1 |
| Flot1 | S.i3vl7 | flotillin 1 |
| Fmo1 | S.c1 | flavin containing monooxygenase 1 |
| Fmo1 | S.i4vl7 | flavin containing monooxygenase 1 |
| Fmod | S.i4vl7 | fibromodulin |
| Fmod | S.c8 | fibromodulin |
| Fmr1 | S.c3 | fragile X mental retardation syndrome 1 |
| Fn1 | S.c2 | fibronectin 1 |
| Fn1 | S.i4vl7 | fibronectin 1 |
| Fndc1 | S.c1 | fibronectin type III domain containing 1 |
| Folr1 | S.i1vl7 | folate receptor 1 (adult) |
| Folr1 | S.i2vl7 | folate receptor 1 (adult) |
| Folr1 | S.c8 | folate receptor 1 (adult) |
| Folr1 | S.i3vl7 | folate receptor 1 (adult) |
| Folr1 | S.i4vl7 | folate receptor 1 (adult) |
| Folr2 | S.c1 | folate receptor 2 (fetal) |
| Fos | S.c3 | FBJ osteosarcoma oncogene |
| Foxh1 | S.c7 | forkhead box H1 |
| Foxk2 | S.c7 | forkhead box K2 |
| Frg1 | S.c6 | FSHD region gene 1 |
| Frk | S.i1vl7 | fyn-related kinase |
| Frmd6 | S.i4vl7 | FERM domain containing 6 |
| Frrs1 | S.c8 | ferric-chelate reductase 1 |
| Frrs1 | S.i2vl7 | ferric-chelate reductase 1 |
| Frrs1 | S.i3vl7 | ferric-chelate reductase 1 |
| Frrs1 | S.i4vl7 | ferric-chelate reductase 1 |
| Fstl1 | S.i3vl7 | follistatin-like 1 |
| Fstl1 | S.i4vl7 | follistatin-like 1 |
| Fstl1 | S.c3 | follistatin-like 1 |
| Fstl1 | S.i2vl7 | follistatin-like 1 |
| Ftl1 | S.i4vl7 | ferritin light chain 1 |
| Fubp1 | S.i1vl7 | far upstream element (FUSE) binding protein 1 |
| Fuca1 | S.i4vl7 | fucosidase, alpha-L- 1, tissue |
| Fuca1 | S.c3 | fucosidase, alpha-L- 1, tissue |
| Fxyd1 | S.c1 | FXYD domain-containing ion transport regulator 1 |
| Fxyd1 | S.i4vl7 | FXYD domain-containing ion transport regulator 1 |
| Fxyd2 | S.c1 | FXYD domain-containing ion transport regulator 2 |
| Fxyd3 | S.i1vl7 | FXYD domain-containing ion transport regulator 3 |
| Fxyd3 | S.i3vl7 | FXYD domain-containing ion transport regulator 3 |
| Fxyd3 | c5.i3vl7 | FXYD domain-containing ion transport regulator 3 |
| Fxyd3 | S.i2vl7 | FXYD domain-containing ion transport regulator 3 |
| Fxyd3 | S.i4vl7 | FXYD domain-containing ion transport regulator 3 |
| Fxyd5 | S.i4vl7 | FXYD domain-containing ion transport regulator 5 |
| Fxyd5 | S.c1 | FXYD domain-containing ion transport regulator 5 |
| Fyn | S.c1 | Fyn proto-oncogene |
| G0s2 | S.i2vl7 | G0/G1 switch gene 2 |
| G0s2 | S.c7 | G0/G1 switch gene 2 |
| G0s2 | S.i4vl7 | G0/G1 switch gene 2 |
| G0s2 | S.i3vl7 | G0/G1 switch gene 2 |
| G3bp2 | S.i4vl7 | GTPase activating protein (SH3 domain) binding protein 2 |
| G3bp2 | S.c3 | GTPase activating protein (SH3 domain) binding protein 2 |
| G3bp2 | S.i3vl7 | GTPase activating protein (SH3 domain) binding protein 2 |
| G6pd2 | S.c7 | glucose-6-phosphate dehydrogenase 2 |
| G6pd2 | S.i1vl7 | glucose-6-phosphate dehydrogenase 2 |
| G6pdx | S.i3vl7 | glucose-6-phosphate dehydrogenase X-linked |
| G6pdx | S.i1vl7 | glucose-6-phosphate dehydrogenase X-linked |
| G6pdx | S.c7 | glucose-6-phosphate dehydrogenase X-linked |
| G6pdx | S.i2vl7 | glucose-6-phosphate dehydrogenase X-linked |
| Gaa | S.c7 | glucosidase, alpha, acid |
| Gab1 | S.i2vl7 | growth factor receptor bound protein 2-associated protein 1 |
| Gab1 | S.i4vl7 | growth factor receptor bound protein 2-associated protein 1 |
| Gab1 | S.c4 | growth factor receptor bound protein 2-associated protein 1 |
| Gab1 | S.i3vl7 | growth factor receptor bound protein 2-associated protein 1 |
| Gab1 | S.i1vl7 | growth factor receptor bound protein 2-associated protein 1 |
| Gabpa | S.c3 | GA repeat binding protein, alpha |
| Gadd45a | S.i1vl7 | growth arrest and DNA-damage-inducible 45 alpha |
| Gadd45a | S.i3vl7 | growth arrest and DNA-damage-inducible 45 alpha |
| Gadd45a | S.i2vl7 | growth arrest and DNA-damage-inducible 45 alpha |
| Gadd45a | S.i4vl7 | growth arrest and DNA-damage-inducible 45 alpha |
| Gadd45a | S.c3 | growth arrest and DNA-damage-inducible 45 alpha |
| Galnt1 | S.c8 | UDP-N-acetyl-alpha-D-galactosamine:polypeptide N-acetylgalactosaminyltransferase 1 |
| Galnt1 | S.i3vl7 | UDP-N-acetyl-alpha-D-galactosamine:polypeptide N-acetylgalactosaminyltransferase 1 |
| Galnt2 | S.i1vl7 | UDP-N-acetyl-alpha-D-galactosamine:polypeptide N-acetylgalactosaminyltransferase 2 |
| Galnt2 | S.i3vl7 | UDP-N-acetyl-alpha-D-galactosamine:polypeptide N-acetylgalactosaminyltransferase 2 |
| Galnt2 | S.i4vl7 | UDP-N-acetyl-alpha-D-galactosamine:polypeptide N-acetylgalactosaminyltransferase 2 |
| Galnt2 | S.i2vl7 | UDP-N-acetyl-alpha-D-galactosamine:polypeptide N-acetylgalactosaminyltransferase 2 |
| Galnt2 | c5.i3vl7 | UDP-N-acetyl-alpha-D-galactosamine:polypeptide N-acetylgalactosaminyltransferase 2 |
| Gas1 | S.c2 | growth arrest specific 1 |
| Gas1 | S.i3vl7 | growth arrest specific 1 |
| Gas1 | S.i4vl7 | growth arrest specific 1 |
| Gas6 | S.c3 | growth arrest specific 6 |
| Gas6 | S.i2vl7 | growth arrest specific 6 |
| Gas6 | S.i3vl7 | growth arrest specific 6 |
| Gas6 | S.i4vl7 | growth arrest specific 6 |
| Gata3 | S.i2vl7 | GATA binding protein 3 |
| Gatm | S.c2 | glycine amidinotransferase (L-arginine:glycine amidinotransferase) |
| Gatm | S.i3vl7 | glycine amidinotransferase (L-arginine:glycine amidinotransferase) |
| Gatm | S.i4vl7 | glycine amidinotransferase (L-arginine:glycine amidinotransferase) |
| Gbf1 | S.i1vl7 | golgi-specific brefeldin A-resistance factor 1 |
| Gbf1 | S.c7 | golgi-specific brefeldin A-resistance factor 1 |
| Gbf1 | S.i4vl7 | golgi-specific brefeldin A-resistance factor 1 |
| Gbp2 | S.c4 | guanylate binding protein 2 |
| Gbp2 | S.i4vl7 | guanylate binding protein 2 |
| Gbp2 | S.i2vl7 | guanylate binding protein 2 |
| Gbp2 | S.i3vl7 | guanylate binding protein 2 |
| Gcdh | S.i4vl7 | glutaryl-Coenzyme A dehydrogenase |
| Gcdh | S.i3vl7 | glutaryl-Coenzyme A dehydrogenase |
| Gch1 | c5.i3vl7 | GTP cyclohydrolase 1 |
| Gch1 | S.i3vl7 | GTP cyclohydrolase 1 |
| Gch1 | S.i1vl7 | GTP cyclohydrolase 1 |
| Gch1 | S.i2vl7 | GTP cyclohydrolase 1 |
| Gclm | S.c6 | glutamate-cysteine ligase, modifier subunit |
| Gclm | S.i1vl7 | glutamate-cysteine ligase, modifier subunit |
| Gcsh | S.c7 | glycine cleavage system protein H (aminomethyl carrier) |
| Gcsh | S.i2vl7 | glycine cleavage system protein H (aminomethyl carrier) |
| Gcsh | S.i3vl7 | glycine cleavage system protein H (aminomethyl carrier) |
| Gdi1 | S.i3vl7 | guanosine diphosphate (GDP) dissociation inhibitor 1 |
| Gdpd5 | S.c7 | glycerophosphodiester phosphodiesterase domain containing 5 |
| Gdpd5 | S.i2vl7 | glycerophosphodiester phosphodiesterase domain containing 5 |
| Gdpd5 | S.i4vl7 | glycerophosphodiester phosphodiesterase domain containing 5 |
| Gdpd5 | S.i1vl7 | glycerophosphodiester phosphodiesterase domain containing 5 |
| Gdpd5 | S.i3vl7 | glycerophosphodiester phosphodiesterase domain containing 5 |
| Ggct | S.c8 | gamma-glutamyl cyclotransferase |
| Ggh | S.c1 | gamma-glutamyl hydrolase |
| Ggt1 | S.c7 | gamma-glutamyltransferase 1 |
| Ggt5 | S.c1 | gamma-glutamyltransferase 5 |
| Ghitm | S.i1vl7 | growth hormone inducible transmembrane protein |
| Ghr | S.c2 | growth hormone receptor |
| Ghr | S.i4vl7 | growth hormone receptor |
| Ghr | S.i3vl7 | growth hormone receptor |
| Gimap4 | S.c1 | GTPase, IMAP family member 4 |
| Ginm1 | S.i2vl7 | glycoprotein integral membrane 1 |
| Ginm1 | S.i1vl7 | glycoprotein integral membrane 1 |
| Ginm1 | S.i4vl7 | glycoprotein integral membrane 1 |
| Ginm1 | S.c4 | glycoprotein integral membrane 1 |
| Gipc1 | S.c7 | GIPC PDZ domain containing family, member 1 |
| Gipc1 | S.i4vl7 | GIPC PDZ domain containing family, member 1 |
| Gipc1 | S.i3vl7 | GIPC PDZ domain containing family, member 1 |
| Gipc1 | S.i2vl7 | GIPC PDZ domain containing family, member 1 |
| Gja1 | S.i3vl7 | gap junction protein, alpha 1 |
| Gja1 | S.c3 | gap junction protein, alpha 1 |
| Gja1 | S.c1 | gap junction protein, alpha 1 |
| Gja1 | S.i4vl7 | gap junction protein, alpha 1 |
| Gjb1 | S.i2vl7 | gap junction protein, beta 1 |
| Gjb1 | S.i3vl7 | gap junction protein, beta 1 |
| Gjb1 | S.i4vl7 | gap junction protein, beta 1 |
| Gjb1 | S.i1vl7 | gap junction protein, beta 1 |
| Gjb1 | S.c7 | gap junction protein, beta 1 |
| Gjb2 | S.c6 | gap junction protein, beta 2 |
| Gkap1 | S.c6 | G kinase anchoring protein 1 |
| Glce | S.i4vl7 | glucuronyl C5-epimerase |
| Gldc | S.i3vl7 | glycine decarboxylase |
| Gldc | S.i1vl7 | glycine decarboxylase |
| Gldc | S.i4vl7 | glycine decarboxylase |
| Gldc | S.c7 | glycine decarboxylase |
| Gldc | S.i2vl7 | glycine decarboxylase |
| Glg1 | S.c2 | golgi apparatus protein 1 |
| Glg1 | S.i2vl7 | golgi apparatus protein 1 |
| Glrx | S.i4vl7 | glutaredoxin |
| Glrx | S.c9 | glutaredoxin |
| Glrx | S.i2vl7 | glutaredoxin |
| Glrx | S.i3vl7 | glutaredoxin |
| Glt25d1 | S.i1vl7 | glycosyltransferase 25 domain containing 1 |
| Glt25d1 | S.i4vl7 | glycosyltransferase 25 domain containing 1 |
| Glt25d1 | S.i2vl7 | glycosyltransferase 25 domain containing 1 |
| Glt25d1 | c5.i3vl7 | glycosyltransferase 25 domain containing 1 |
| Glt25d1 | S.i3vl7 | glycosyltransferase 25 domain containing 1 |
| Gltp | S.c9 | glycolipid transfer protein |
| Gltp | S.i4vl7 | glycolipid transfer protein |
| Gltp | S.i3vl7 | glycolipid transfer protein |
| Gltp | S.i2vl7 | glycolipid transfer protein |
| Gltp | S.c2 | glycolipid transfer protein |
| Glud1 | S.c3 | glutamate dehydrogenase 1 |
| Glud1 | S.i4vl7 | glutamate dehydrogenase 1 |
| Glud1 | S.i2vl7 | glutamate dehydrogenase 1 |
| Glul | S.c1 | glutamate-ammonia ligase (glutamine synthetase) |
| Gm10257 | S.i3vl7 | predicted gene 10257 |
| Gm10257 | S.i1vl7 | predicted gene 10257 |
| Gm10257 | S.c4 | predicted gene 10257 |
| Gm10349 | S.c4 | predicted gene 10349 |
| Gm10480 | S.c3 | predicted gene 10480 |
| Gm10591 | S.c1 | predicted gene 10591 |
| Gm12657 | S.i1vl7 | predicted gene 12657 |
| Gm12657 | S.i3vl7 | predicted gene 12657 |
| Gm12657 | S.c4 | predicted gene 12657 |
| Gm12854 | S.i2vl7 | predicted gene 12854 |
| Gm12854 | S.i4vl7 | predicted gene 12854 |
| Gm12854 | S.i1vl7 | predicted gene 12854 |
| Gm12854 | S.i3vl7 | predicted gene 12854 |
| Gm12854 | S.c4 | predicted gene 12854 |
| Gm13304 | S.c1 | predicted gene 13304 |
| Gm14308 | S.i3vl7 | predicted gene 14308 |
| Gm14308 | S.i1vl7 | predicted gene 14308 |
| Gm14308 | S.i2vl7 | predicted gene 14308 |
| Gm14308 | S.i4vl7 | predicted gene 14308 |
| Gm14308 | S.c8 | predicted gene 14308 |
| Gm14326 | S.i3vl7 | predicted gene 14326 |
| Gm14326 | S.i4vl7 | predicted gene 14326 |
| Gm14326 | S.i2vl7 | predicted gene 14326 |
| Gm14326 | S.i1vl7 | predicted gene 14326 |
| Gm14326 | S.c8 | predicted gene 14326 |
| Gm14430 | S.i4vl7 | predicted gene 14430 |
| Gm14430 | S.i1vl7 | predicted gene 14430 |
| Gm14430 | S.i2vl7 | predicted gene 14430 |
| Gm14430 | S.c8 | predicted gene 14430 |
| Gm14430 | S.i3vl7 | predicted gene 14430 |
| Gm14434 | S.c8 | predicted gene 14434 |
| Gm14434 | S.i2vl7 | predicted gene 14434 |
| Gm14434 | S.i3vl7 | predicted gene 14434 |
| Gm14434 | S.i1vl7 | predicted gene 14434 |
| Gm14434 | S.i4vl7 | predicted gene 14434 |
| Gm14548 | S.c2 | predicted gene 14548 |
| Gm14548 | S.i3vl7 | predicted gene 14548 |
| Gm15448 | S.i3vl7 | predicted gene 15448 |
| Gm15448 | S.c2 | predicted gene 15448 |
| Gm15453 | S.i1vl7 | predicted gene 15453 |
| Gm15466 | S.c3 | predicted gene 15466 |
| Gm15466 | S.i4vl7 | predicted gene 15466 |
| Gm15466 | S.i2vl7 | predicted gene 15466 |
| Gm15466 | S.i1vl7 | predicted gene 15466 |
| Gm15466 | S.i3vl7 | predicted gene 15466 |
| Gm1862 | S.c4 | predicted pseudogene 1862 |
| Gm1862 | S.i1vl7 | predicted pseudogene 1862 |
| Gm1987 | S.c1 | predicted gene 1987 |
| Gm20746 | S.i4vl7 | predicted gene, 20746 |
| Gm21399 | S.i1vl7 | peroxiredoxin 1 pseudogene |
| Gm21541 | S.c1 | predicted gene, 21541 |
| Gm21559 | S.c2 | predicted gene, 21559 |
| Gm21596 | S.i4vl7 | predicted gene, 21596 |
| Gm21596 | S.c3 | predicted gene, 21596 |
| Gm21596 | S.i2vl7 | predicted gene, 21596 |
| Gm2260 | S.i3vl7 | predicted gene 2260 |
| Gm2260 | S.c4 | predicted gene 2260 |
| Gm2260 | S.i1vl7 | predicted gene 2260 |
| Gm2260 | S.i4vl7 | predicted gene 2260 |
| Gm2274 | S.i4vl7 | predicted gene 2274 |
| Gm2274 | S.i3vl7 | predicted gene 2274 |
| Gm2274 | S.c4 | predicted gene 2274 |
| Gm2274 | S.i1vl7 | predicted gene 2274 |
| Gm2444 | S.c4 | predicted gene 2444 |
| Gm2444 | S.i1vl7 | predicted gene 2444 |
| Gm2904 | S.c4 | predicted pseudogene 2904 |
| Gm2a | S.i2vl7 | GM2 ganglioside activator protein |
| Gm2a | S.i4vl7 | GM2 ganglioside activator protein |
| Gm2a | S.i1vl7 | GM2 ganglioside activator protein |
| Gm2a | S.i3vl7 | GM2 ganglioside activator protein |
| Gm2a | S.c7 | GM2 ganglioside activator protein |
| Gm3258 | c5.i3vl7 | predicted gene 3258 |
| Gm3258 | S.i1vl7 | predicted gene 3258 |
| Gm3258 | S.i2vl7 | predicted gene 3258 |
| Gm3258 | S.i4vl7 | predicted gene 3258 |
| Gm3258 | S.i3vl7 | predicted gene 3258 |
| Gm3837 | S.i1vl7 | predicted gene 3837 |
| Gm3837 | S.i2vl7 | predicted gene 3837 |
| Gm3837 | S.c4 | predicted gene 3837 |
| Gm4705 | S.i2vl7 | predicted gene 4705 |
| Gm4705 | S.c8 | predicted gene 4705 |
| Gm4705 | S.i4vl7 | predicted gene 4705 |
| Gm4724 | S.i2vl7 | predicted gene 4724 |
| Gm4724 | S.i4vl7 | predicted gene 4724 |
| Gm4724 | S.i3vl7 | predicted gene 4724 |
| Gm4724 | S.c8 | predicted gene 4724 |
| Gm4724 | S.i1vl7 | predicted gene 4724 |
| Gm4788 | S.c1 | predicted gene 4788 |
| Gm5068 | S.i2vl7 | predicted gene 5068 |
| Gm5068 | S.i4vl7 | predicted gene 5068 |
| Gm5068 | S.i1vl7 | predicted gene 5068 |
| Gm5068 | S.i3vl7 | predicted gene 5068 |
| Gm5068 | S.c4 | predicted gene 5068 |
| Gm5161 | S.c2 | predicted pseudogene 5161 |
| Gm5553 | S.i4vl7 | predicted gene 5553 |
| Gm5553 | S.i3vl7 | predicted gene 5553 |
| Gm5553 | S.c7 | predicted gene 5553 |
| Gm6115 | S.i2vl7 | predicted gene 6115 |
| Gm6115 | S.i4vl7 | predicted gene 6115 |
| Gm6115 | S.c3 | predicted gene 6115 |
| Gm6404 | S.i2vl7 | predicted gene 6404 |
| Gm6404 | S.c8 | predicted gene 6404 |
| Gm6404 | S.i4vl7 | predicted gene 6404 |
| Gm6749 | S.i1vl7 | predicted pseudogene 6749 |
| Gm6749 | S.c4 | predicted pseudogene 6749 |
| Gm6749 | S.i3vl7 | predicted pseudogene 6749 |
| Gm6750 | S.i4vl7 | predicted gene 6750 |
| Gm6750 | S.c4 | predicted gene 6750 |
| Gm6750 | S.i1vl7 | predicted gene 6750 |
| Gm7334 | S.i4vl7 | B-cell translocation gene 3 pseudogene |
| Gm7334 | S.c7 | B-cell translocation gene 3 pseudogene |
| Gm7334 | S.i3vl7 | B-cell translocation gene 3 pseudogene |
| Gm7334 | S.i1vl7 | B-cell translocation gene 3 pseudogene |
| Gm7334 | S.i2vl7 | B-cell translocation gene 3 pseudogene |
| Gm7931 | S.i1vl7 | predicted pseudogene 7931 |
| Gm7931 | S.i4vl7 | predicted pseudogene 7931 |
| Gm7931 | S.c4 | predicted pseudogene 7931 |
| Gm9386 | S.c4 | predicted pseudogene 9386 |
| Gm9525 | S.i1vl7 | predicted gene 9525 |
| Gm9525 | S.i4vl7 | predicted gene 9525 |
| Gm9525 | S.c4 | predicted gene 9525 |
| Gm9769 | S.c3 | predicted pseudogene 9769 |
| Gm9780 | S.c2 | predicted gene 9780 |
| Gm9835 | S.i3vl7 | predicted pseudogene 9835 |
| Gmfg | S.i3vl7 | glia maturation factor, gamma |
| Gmppb | S.c7 | GDP-mannose pyrophosphorylase B |
| Gna12 | S.c2 | guanine nucleotide binding protein, alpha 12 |
| Gna12 | S.i4vl7 | guanine nucleotide binding protein, alpha 12 |
| Gna12 | S.i3vl7 | guanine nucleotide binding protein, alpha 12 |
| Gna14 | S.c6 | guanine nucleotide binding protein, alpha 14 |
| Gnai1 | S.i4vl7 | guanine nucleotide binding protein (G protein), alpha inhibiting 1 |
| Gnai1 | S.c2 | guanine nucleotide binding protein (G protein), alpha inhibiting 1 |
| Gnai2 | S.i2vl7 | guanine nucleotide binding protein (G protein), alpha inhibiting 2 |
| Gnai2 | S.i1vl7 | guanine nucleotide binding protein (G protein), alpha inhibiting 2 |
| Gnai2 | S.c2 | guanine nucleotide binding protein (G protein), alpha inhibiting 2 |
| Gnai2 | S.i4vl7 | guanine nucleotide binding protein (G protein), alpha inhibiting 2 |
| Gnai2 | S.i3vl7 | guanine nucleotide binding protein (G protein), alpha inhibiting 2 |
| Gnai2 | S.c3 | guanine nucleotide binding protein (G protein), alpha inhibiting 2 |
| Gnb1 | S.i3vl7 | guanine nucleotide binding protein (G protein), beta 1 |
| Gnb1 | S.i2vl7 | guanine nucleotide binding protein (G protein), beta 1 |
| Gnb1 | c5.i3vl7 | guanine nucleotide binding protein (G protein), beta 1 |
| Gnb4 | S.i2vl7 | guanine nucleotide binding protein (G protein), beta 4 |
| Gnb4 | S.c7 | guanine nucleotide binding protein (G protein), beta 4 |
| Gnb4 | S.i4vl7 | guanine nucleotide binding protein (G protein), beta 4 |
| Gnb4 | S.i1vl7 | guanine nucleotide binding protein (G protein), beta 4 |
| Gnb4 | S.i3vl7 | guanine nucleotide binding protein (G protein), beta 4 |
| Gne | S.i3vl7 | glucosamine (UDP-N-acetyl)-2-epimerase/N-acetylmannosamine kinase |
| Gne | S.i4vl7 | glucosamine (UDP-N-acetyl)-2-epimerase/N-acetylmannosamine kinase |
| Gne | S.i1vl7 | glucosamine (UDP-N-acetyl)-2-epimerase/N-acetylmannosamine kinase |
| Gne | S.i2vl7 | glucosamine (UDP-N-acetyl)-2-epimerase/N-acetylmannosamine kinase |
| Gne | S.c7 | glucosamine (UDP-N-acetyl)-2-epimerase/N-acetylmannosamine kinase |
| Gng10 | S.i4vl7 | guanine nucleotide binding protein (G protein), gamma 10 |
| Gng10 | S.c3 | guanine nucleotide binding protein (G protein), gamma 10 |
| Gng10 | S.i3vl7 | guanine nucleotide binding protein (G protein), gamma 10 |
| Gng12 | S.i2vl7 | guanine nucleotide binding protein (G protein), gamma 12 |
| Gng12 | S.c4 | guanine nucleotide binding protein (G protein), gamma 12 |
| Gng12 | S.i1vl7 | guanine nucleotide binding protein (G protein), gamma 12 |
| Gng12 | S.i3vl7 | guanine nucleotide binding protein (G protein), gamma 12 |
| Gng12 | S.i4vl7 | guanine nucleotide binding protein (G protein), gamma 12 |
| Gnmt | S.i1vl7 | glycine N-methyltransferase |
| Gnmt | S.c7 | glycine N-methyltransferase |
| Gnmt | S.i3vl7 | glycine N-methyltransferase |
| Gnmt | S.i4vl7 | glycine N-methyltransferase |
| Gnmt | S.i2vl7 | glycine N-methyltransferase |
| Gnpda1 | S.i1vl7 | glucosamine-6-phosphate deaminase 1 |
| Gnpda1 | S.c4 | glucosamine-6-phosphate deaminase 1 |
| Gnpnat1 | S.i3vl7 | glucosamine-phosphate N-acetyltransferase 1 |
| Gnpnat1 | S.i4vl7 | glucosamine-phosphate N-acetyltransferase 1 |
| Gnpnat1 | S.c9 | glucosamine-phosphate N-acetyltransferase 1 |
| Gnpnat1 | S.i2vl7 | glucosamine-phosphate N-acetyltransferase 1 |
| Gns | S.i3vl7 | glucosamine (N-acetyl)-6-sulfatase |
| Gns | S.i2vl7 | glucosamine (N-acetyl)-6-sulfatase |
| Gns | S.c3 | glucosamine (N-acetyl)-6-sulfatase |
| Gns | S.i4vl7 | glucosamine (N-acetyl)-6-sulfatase |
| Golph3 | S.i3vl7 | golgi phosphoprotein 3 |
| Golph3 | S.i2vl7 | golgi phosphoprotein 3 |
| Golph3 | S.c7 | golgi phosphoprotein 3 |
| Golph3 | S.i4vl7 | golgi phosphoprotein 3 |
| Gosr2 | S.i4vl7 | golgi SNAP receptor complex member 2 |
| Gosr2 | S.c9 | golgi SNAP receptor complex member 2 |
| Gp49a | S.c1 | glycoprotein 49 A |
| Gp49a | S.i4vl7 | glycoprotein 49 A |
| Gpaa1 | S.c7 | GPI anchor attachment protein 1 |
| Gpaa1 | S.i1vl7 | GPI anchor attachment protein 1 |
| Gpam | S.c3 | glycerol-3-phosphate acyltransferase, mitochondrial |
| Gpam | S.i3vl7 | glycerol-3-phosphate acyltransferase, mitochondrial |
| Gpam | S.i4vl7 | glycerol-3-phosphate acyltransferase, mitochondrial |
| Gpcpd1 | S.i4vl7 | glycerophosphocholine phosphodiesterase GDE1 homolog (S. cerevisiae) |
| Gpcpd1 | S.i2vl7 | glycerophosphocholine phosphodiesterase GDE1 homolog (S. cerevisiae) |
| Gpcpd1 | S.c8 | glycerophosphocholine phosphodiesterase GDE1 homolog (S. cerevisiae) |
| Gpd1 | S.c1 | glycerol-3-phosphate dehydrogenase 1 (soluble) |
| Gpd2 | S.i2vl7 | glycerol phosphate dehydrogenase 2, mitochondrial |
| Gpd2 | S.i1vl7 | glycerol phosphate dehydrogenase 2, mitochondrial |
| Gpd2 | S.c4 | glycerol phosphate dehydrogenase 2, mitochondrial |
| Gphn | S.i3vl7 | gephyrin |
| Gphn | S.c7 | gephyrin |
| Gphn | S.i4vl7 | gephyrin |
| Gphn | S.i2vl7 | gephyrin |
| Gphn | S.i1vl7 | gephyrin |
| Gpi1 | S.i1vl7 | glucose phosphate isomerase 1 |
| Gpi1 | S.i4vl7 | glucose phosphate isomerase 1 |
| Gpi1 | S.i3vl7 | glucose phosphate isomerase 1 |
| Gpi1 | S.i2vl7 | glucose phosphate isomerase 1 |
| Gpi1 | c5.i3vl7 | glucose phosphate isomerase 1 |
| Gpn1 | S.i2vl7 | GPN-loop GTPase 1 |
| Gpn1 | S.c4 | GPN-loop GTPase 1 |
| Gpn1 | S.i4vl7 | GPN-loop GTPase 1 |
| Gpn1 | S.i3vl7 | GPN-loop GTPase 1 |
| Gpr133 | S.i4vl7 | G protein-coupled receptor 133 |
| Gpr133 | S.c1 | G protein-coupled receptor 133 |
| Gpr137b | S.c1 | G protein-coupled receptor 137B |
| Gpr137b | S.i4vl7 | G protein-coupled receptor 137B |
| Gps1 | S.c8 | G protein pathway suppressor 1 |
| Gps1 | S.i4vl7 | G protein pathway suppressor 1 |
| Gps1 | S.i3vl7 | G protein pathway suppressor 1 |
| Gps1 | S.i2vl7 | G protein pathway suppressor 1 |
| Gpx3 | S.i4vl7 | glutathione peroxidase 3 |
| Gpx3 | S.c2 | glutathione peroxidase 3 |
| Gpx4 | S.i1vl7 | glutathione peroxidase 4 |
| Gpx4 | S.i2vl7 | glutathione peroxidase 4 |
| Gramd3 | S.i1vl7 | GRAM domain containing 3 |
| Gramd3 | S.c6 | GRAM domain containing 3 |
| Grina | S.c2 | glutamate receptor, ionotropic, N-methyl D-aspartate-associated protein 1 (glutamate binding) |
| Grina | S.i2vl7 | glutamate receptor, ionotropic, N-methyl D-aspartate-associated protein 1 (glutamate binding) |
| Grina | S.i3vl7 | glutamate receptor, ionotropic, N-methyl D-aspartate-associated protein 1 (glutamate binding) |
| Grn | S.i4vl7 | granulin |
| Grn | S.i2vl7 | granulin |
| Grn | S.i3vl7 | granulin |
| Grn | S.i1vl7 | granulin |
| Grn | S.c3 | granulin |
| Grpel2 | S.c6 | GrpE-like 2, mitochondrial |
| Gsn | S.c2 | gelsolin |
| Gsr | S.i3vl7 | glutathione reductase |
| Gsr | S.c4 | glutathione reductase |
| Gsr | S.i1vl7 | glutathione reductase |
| Gstk1 | S.i1vl7 | glutathione S-transferase kappa 1 |
| Gstk1 | S.c7 | glutathione S-transferase kappa 1 |
| Gstz1 | S.i2vl7 | glutathione transferase zeta 1 (maleylacetoacetate isomerase) |
| Gstz1 | S.c2 | glutathione transferase zeta 1 (maleylacetoacetate isomerase) |
| Gtf2a1 | S.i2vl7 | general transcription factor II A, 1 |
| Gtf2a1 | S.c4 | general transcription factor II A, 1 |
| Gtf2a1 | S.i3vl7 | general transcription factor II A, 1 |
| Gtf2a1 | S.i4vl7 | general transcription factor II A, 1 |
| Gtf2e2 | S.c6 | general transcription factor II E, polypeptide 2 (beta subunit) |
| Gtpbp4 | S.c4 | GTP binding protein 4 |
| Guca1a | S.i3vl7 | guanylate cyclase activator 1a (retina) |
| Guca1a | S.i2vl7 | guanylate cyclase activator 1a (retina) |
| Guca1a | S.i4vl7 | guanylate cyclase activator 1a (retina) |
| Gusb | S.c4 | glucuronidase, beta |
| Gusb | S.i4vl7 | glucuronidase, beta |
| Gyg | S.i1vl7 | glycogenin |
| Gyg | S.i3vl7 | glycogenin |
| Gyg | S.i4vl7 | glycogenin |
| Gyg | S.c4 | glycogenin |
| Gyg | S.i2vl7 | glycogenin |
| Gyk | S.c8 | glycerol kinase |
| Gyk | S.i1vl7 | glycerol kinase |
| Gyk | S.i2vl7 | glycerol kinase |
| Gyk | S.i4vl7 | glycerol kinase |
| Gyk | S.i3vl7 | glycerol kinase |
| H1foo | S.c8 | H1 histone family, member O, oocyte-specific |
| H2-Aa | S.c2 | histocompatibility 2, class II antigen A, alpha |
| H2-Aa | S.i3vl7 | histocompatibility 2, class II antigen A, alpha |
| H2-Aa | S.i4vl7 | histocompatibility 2, class II antigen A, alpha |
| H2-Ab1 | S.i4vl7 | histocompatibility 2, class II antigen A, beta 1 |
| H2-Ab1 | S.c2 | histocompatibility 2, class II antigen A, beta 1 |
| H2-Ab1 | S.i3vl7 | histocompatibility 2, class II antigen A, beta 1 |
| H2-DMb1 | S.c2 | histocompatibility 2, class II, locus Mb1 |
| H2-Eb1 | S.i4vl7 | histocompatibility 2, class II antigen E beta |
| H2-Eb1 | S.c2 | histocompatibility 2, class II antigen E beta |
| H2-Eb1 | S.i3vl7 | histocompatibility 2, class II antigen E beta |
| H2-Q5 | S.i4vl7 | histocompatibility 2, Q region locus 5 |
| H2afv | S.i3vl7 | H2A histone family, member V |
| H2afv | S.i1vl7 | H2A histone family, member V |
| H2afv | S.i2vl7 | H2A histone family, member V |
| H2afv | S.i4vl7 | H2A histone family, member V |
| H2afv | S.c3 | H2A histone family, member V |
| H2afx | S.i4vl7 | H2A histone family, member X |
| H2afx | S.i3vl7 | H2A histone family, member X |
| H2afx | c5.i3vl7 | H2A histone family, member X |
| H2afy | S.i2vl7 | H2A histone family, member Y |
| H2afy | S.c3 | H2A histone family, member Y |
| H2afy | S.i3vl7 | H2A histone family, member Y |
| H2afy | S.i4vl7 | H2A histone family, member Y |
| H2afy | c5.i3vl7 | H2A histone family, member Y |
| H3f3a | S.c4 | H3 histone, family 3A |
| H3f3a | S.i1vl7 | H3 histone, family 3A |
| H3f3a | S.i3vl7 | H3 histone, family 3A |
| H3f3b | S.i3vl7 | H3 histone, family 3B |
| H3f3b | S.i1vl7 | H3 histone, family 3B |
| H3f3b | S.c4 | H3 histone, family 3B |
| H3f3c | S.i3vl7 | H3 histone, family 3C |
| H3f3c | S.i1vl7 | H3 histone, family 3C |
| H3f3c | S.c4 | H3 histone, family 3C |
| H6pd | S.c1 | hexose-6-phosphate dehydrogenase (glucose 1-dehydrogenase) |
| Hadh | S.c3 | hydroxyacyl-Coenzyme A dehydrogenase |
| Hadhb | S.i1vl7 | hydroxyacyl-Coenzyme A dehydrogenase/3-ketoacyl-Coenzyme A thiolase/enoyl-Coenzyme A hydratase (trifunctional protein), beta subunit |
| Hadhb | S.i2vl7 | hydroxyacyl-Coenzyme A dehydrogenase/3-ketoacyl-Coenzyme A thiolase/enoyl-Coenzyme A hydratase (trifunctional protein), beta subunit |
| Hadhb | S.c4 | hydroxyacyl-Coenzyme A dehydrogenase/3-ketoacyl-Coenzyme A thiolase/enoyl-Coenzyme A hydratase (trifunctional protein), beta subunit |
| Hadhb | S.i3vl7 | hydroxyacyl-Coenzyme A dehydrogenase/3-ketoacyl-Coenzyme A thiolase/enoyl-Coenzyme A hydratase (trifunctional protein), beta subunit |
| Hadhb | S.i4vl7 | hydroxyacyl-Coenzyme A dehydrogenase/3-ketoacyl-Coenzyme A thiolase/enoyl-Coenzyme A hydratase (trifunctional protein), beta subunit |
| Hal | S.i4vl7 | histidine ammonia lyase |
| Hal | S.c2 | histidine ammonia lyase |
| Hat1 | S.c8 | histone aminotransferase 1 |
| Haus3 | S.c1 | HAUS augmin-like complex, subunit 3 |
| Hbs1l | S.c9 | Hbs1-like (S. cerevisiae) |
| Hbs1l | S.i4vl7 | Hbs1-like (S. cerevisiae) |
| Hck | S.i4vl7 | hemopoietic cell kinase |
| Hck | S.c1 | hemopoietic cell kinase |
| Hcls1 | S.i4vl7 | hematopoietic cell specific Lyn substrate 1 |
| Hdac3 | S.i1vl7 | histone deacetylase 3 |
| Hdac3 | S.c6 | histone deacetylase 3 |
| Hdac5 | S.c2 | histone deacetylase 5 |
| Hdac5 | S.i3vl7 | histone deacetylase 5 |
| Hdac5 | S.i2vl7 | histone deacetylase 5 |
| Hdac5 | S.i4vl7 | histone deacetylase 5 |
| Hdac6 | S.i1vl7 | histone deacetylase 6 |
| Hdac6 | S.c6 | histone deacetylase 6 |
| Hddc2 | S.c9 | HD domain containing 2 |
| Helb | S.c3 | helicase (DNA) B |
| Herc1 | S.i4vl7 | hect (homologous to the E6-AP (UBE3A) carboxyl terminus) domain and RCC1 (CHC1)-like domain (RLD) 1 |
| Herpud1 | S.c2 | homocysteine-inducible, endoplasmic reticulum stress-inducible, ubiquitin-like domain member 1 |
| Hes5 | S.i2vl7 | hairy and enhancer of split 5 (Drosophila) |
| Hes5 | S.i3vl7 | hairy and enhancer of split 5 (Drosophila) |
| Hes5 | S.c8 | hairy and enhancer of split 5 (Drosophila) |
| Hes5 | S.i4vl7 | hairy and enhancer of split 5 (Drosophila) |
| Hexa | S.c2 | hexosaminidase A |
| Hexa | S.i4vl7 | hexosaminidase A |
| Hfe | S.c1 | hemochromatosis |
| Higd1a | S.c7 | HIG1 domain family, member 1A |
| Hipk1 | S.i2vl7 | homeodomain interacting protein kinase 1 |
| Hipk1 | S.i1vl7 | homeodomain interacting protein kinase 1 |
| Hipk1 | S.i4vl7 | homeodomain interacting protein kinase 1 |
| Hipk1 | S.c7 | homeodomain interacting protein kinase 1 |
| Hk2 | S.c7 | hexokinase 2 |
| Hk2 | S.i1vl7 | hexokinase 2 |
| Hk2 | S.i2vl7 | hexokinase 2 |
| Hmbox1 | S.i4vl7 | homeobox containing 1 |
| Hmbox1 | S.c4 | homeobox containing 1 |
| Hmbox1 | S.i2vl7 | homeobox containing 1 |
| Hmbox1 | S.i3vl7 | homeobox containing 1 |
| Hmbox1 | S.i1vl7 | homeobox containing 1 |
| Hmces | S.i4vl7 | 5-hydroxymethylcytosine (hmC) binding, ES cell specific |
| Hmces | c5.i3vl7 | 5-hydroxymethylcytosine (hmC) binding, ES cell specific |
| Hmces | S.i3vl7 | 5-hydroxymethylcytosine (hmC) binding, ES cell specific |
| Hmces | S.i1vl7 | 5-hydroxymethylcytosine (hmC) binding, ES cell specific |
| Hmces | S.i2vl7 | 5-hydroxymethylcytosine (hmC) binding, ES cell specific |
| Hmgb1 | S.i4vl7 | high mobility group box 1 |
| Hmgb1 | S.c3 | high mobility group box 1 |
| Hmgb1 | S.i2vl7 | high mobility group box 1 |
| Hmgb3 | S.c4 | high mobility group box 3 |
| Hmgb3 | S.i1vl7 | high mobility group box 3 |
| Hmgn1 | S.i2vl7 | high mobility group nucleosomal binding domain 1 |
| Hmgn1 | S.i1vl7 | high mobility group nucleosomal binding domain 1 |
| Hmgn1 | S.c6 | high mobility group nucleosomal binding domain 1 |
| Hmgn2 | S.c4 | high mobility group nucleosomal binding domain 2 |
| Hmgn2 | S.i1vl7 | high mobility group nucleosomal binding domain 2 |
| Hmgn2 | S.i4vl7 | high mobility group nucleosomal binding domain 2 |
| Hmgn5 | S.i1vl7 | high-mobility group nucleosome binding domain 5 |
| Hmgn5 | S.i4vl7 | high-mobility group nucleosome binding domain 5 |
| Hmgn5 | S.c7 | high-mobility group nucleosome binding domain 5 |
| Hmgn5 | S.i2vl7 | high-mobility group nucleosome binding domain 5 |
| Hmgn5 | S.i3vl7 | high-mobility group nucleosome binding domain 5 |
| Hmha1 | S.c1 | histocompatibility (minor) HA-1 |
| Hmox1 | S.i4vl7 | heme oxygenase (decycling) 1 |
| Hn1 | S.i4vl7 | hematological and neurological expressed sequence 1 |
| Hn1 | S.i2vl7 | hematological and neurological expressed sequence 1 |
| Hn1 | S.i3vl7 | hematological and neurological expressed sequence 1 |
| Hn1 | S.c4 | hematological and neurological expressed sequence 1 |
| Hn1 | S.i1vl7 | hematological and neurological expressed sequence 1 |
| Hnrnpa2b1 | S.i4vl7 | heterogeneous nuclear ribonucleoprotein A2/B1 |
| Hnrnpa2b1 | S.c2 | heterogeneous nuclear ribonucleoprotein A2/B1 |
| Hnrnpc | S.c3 | heterogeneous nuclear ribonucleoprotein C |
| Hnrnph1 | S.c4 | heterogeneous nuclear ribonucleoprotein H1 |
| Hnrnpk | S.i2vl7 | heterogeneous nuclear ribonucleoprotein K |
| Hnrnpk | S.c4 | heterogeneous nuclear ribonucleoprotein K |
| Hnrnpk | S.i3vl7 | heterogeneous nuclear ribonucleoprotein K |
| Hnrnpk | S.i4vl7 | heterogeneous nuclear ribonucleoprotein K |
| Hnrnpm | S.i4vl7 | heterogeneous nuclear ribonucleoprotein M |
| Hnrnpm | S.c4 | heterogeneous nuclear ribonucleoprotein M |
| Hnrnpr | S.c3 | heterogeneous nuclear ribonucleoprotein R |
| Hnrnpr | S.i4vl7 | heterogeneous nuclear ribonucleoprotein R |
| Homer2 | S.i4vl7 | homer homolog 2 (Drosophila) |
| Homer2 | S.i3vl7 | homer homolog 2 (Drosophila) |
| Homer2 | S.c8 | homer homolog 2 (Drosophila) |
| Homer2 | S.i2vl7 | homer homolog 2 (Drosophila) |
| Hoxb3 | S.i4vl7 | homeobox B3 |
| Hp | S.i4vl7 | haptoglobin |
| Hp | S.i3vl7 | haptoglobin |
| Hp | S.c2 | haptoglobin |
| Hp1bp3 | S.i3vl7 | heterochromatin protein 1, binding protein 3 |
| Hp1bp3 | S.c4 | heterochromatin protein 1, binding protein 3 |
| Hp1bp3 | S.i4vl7 | heterochromatin protein 1, binding protein 3 |
| Hp1bp3 | S.i2vl7 | heterochromatin protein 1, binding protein 3 |
| Hpd | S.i4vl7 | 4-hydroxyphenylpyruvic acid dioxygenase |
| Hpgd | S.c1 | hydroxyprostaglandin dehydrogenase 15 (NAD) |
| Hprt | S.i4vl7 | hypoxanthine guanine phosphoribosyl transferase |
| Hprt | S.c4 | hypoxanthine guanine phosphoribosyl transferase |
| Hsd17b11 | S.c4 | hydroxysteroid (17-beta) dehydrogenase 11 |
| Hsd17b11 | S.i1vl7 | hydroxysteroid (17-beta) dehydrogenase 11 |
| Hsd17b7 | S.c7 | hydroxysteroid (17-beta) dehydrogenase 7 |
| Hsdl2 | S.i2vl7 | hydroxysteroid dehydrogenase like 2 |
| Hsdl2 | S.i1vl7 | hydroxysteroid dehydrogenase like 2 |
| Hsdl2 | S.i3vl7 | hydroxysteroid dehydrogenase like 2 |
| Hsdl2 | S.i4vl7 | hydroxysteroid dehydrogenase like 2 |
| Hsdl2 | c5.i3vl7 | hydroxysteroid dehydrogenase like 2 |
| Hsp90ab1 | S.c8 | heat shock protein 90 alpha (cytosolic), class B member 1 |
| Hsp90ab1 | S.i1vl7 | heat shock protein 90 alpha (cytosolic), class B member 1 |
| Hsp90ab1 | S.i4vl7 | heat shock protein 90 alpha (cytosolic), class B member 1 |
| Hsp90b1 | S.i3vl7 | heat shock protein 90, beta (Grp94), member 1 |
| Hsp90b1 | S.c2 | heat shock protein 90, beta (Grp94), member 1 |
| Hspa4 | S.c2 | heat shock protein 4 |
| Hspa4l | S.i2vl7 | heat shock protein 4 like |
| Hspa4l | S.i4vl7 | heat shock protein 4 like |
| Hspa4l | S.c9 | heat shock protein 4 like |
| Hspa5 | S.c2 | heat shock protein 5 |
| Hspa5 | S.i3vl7 | heat shock protein 5 |
| Hspb11 | S.c9 | heat shock protein family B (small), member 11 |
| Hspb8 | S.i2vl7 | heat shock protein 8 |
| Hspd1 | S.c4 | heat shock protein 1 (chaperonin) |
| Htatip2 | S.i1vl7 | HIV-1 tat interactive protein 2, homolog (human) |
| Htatip2 | S.i3vl7 | HIV-1 tat interactive protein 2, homolog (human) |
| Htatip2 | S.i4vl7 | HIV-1 tat interactive protein 2, homolog (human) |
| Htatip2 | S.i2vl7 | HIV-1 tat interactive protein 2, homolog (human) |
| Htatip2 | c5.i3vl7 | HIV-1 tat interactive protein 2, homolog (human) |
| Htra1 | S.c2 | HtrA serine peptidase 1 |
| Htra1 | S.i4vl7 | HtrA serine peptidase 1 |
| Hypk | S.c4 | huntingtin interacting protein K |
| Hypk | S.i1vl7 | huntingtin interacting protein K |
| Iars2 | S.i4vl7 | isoleucine-tRNA synthetase 2, mitochondrial |
| Ica1 | S.i4vl7 | islet cell autoantigen 1 |
| Ica1 | S.c8 | islet cell autoantigen 1 |
| Ica1 | S.i3vl7 | islet cell autoantigen 1 |
| Icam1 | S.c1 | intercellular adhesion molecule 1 |
| Icam1 | S.i4vl7 | intercellular adhesion molecule 1 |
| Id1 | S.c1 | inhibitor of DNA binding 1 |
| Id1 | S.i4vl7 | inhibitor of DNA binding 1 |
| Id3 | S.c1 | inhibitor of DNA binding 3 |
| Idh1 | S.i3vl7 | isocitrate dehydrogenase 1 (NADP+), soluble |
| Idh1 | S.c3 | isocitrate dehydrogenase 1 (NADP+), soluble |
| Idh1 | S.i2vl7 | isocitrate dehydrogenase 1 (NADP+), soluble |
| Idh1 | S.i4vl7 | isocitrate dehydrogenase 1 (NADP+), soluble |
| Idh3a | S.i4vl7 | isocitrate dehydrogenase 3 (NAD+) alpha |
| Idh3a | S.c1 | isocitrate dehydrogenase 3 (NAD+) alpha |
| Idi1 | S.i3vl7 | isopentenyl-diphosphate delta isomerase |
| Idi1 | S.i2vl7 | isopentenyl-diphosphate delta isomerase |
| Idi1 | S.i4vl7 | isopentenyl-diphosphate delta isomerase |
| Idi1 | S.c7 | isopentenyl-diphosphate delta isomerase |
| Idi1 | S.i1vl7 | isopentenyl-diphosphate delta isomerase |
| Ier2 | S.c2 | immediate early response 2 |
| Ifi204 | S.i4vl7 | interferon activated gene 204 |
| Ifi204 | S.c3 | interferon activated gene 204 |
| Ifi204 | S.i3vl7 | interferon activated gene 204 |
| Ifi205 | S.c3 | interferon activated gene 205 |
| Ifi205 | S.i4vl7 | interferon activated gene 205 |
| Ifi27 | S.i2vl7 | interferon, alpha-inducible protein 27 |
| Ifi27 | S.c3 | interferon, alpha-inducible protein 27 |
| Ifi27 | S.i3vl7 | interferon, alpha-inducible protein 27 |
| Ifi27 | S.i4vl7 | interferon, alpha-inducible protein 27 |
| Ifi30 | S.i4vl7 | interferon gamma inducible protein 30 |
| Ifi30 | S.c9 | interferon gamma inducible protein 30 |
| Ifih1 | S.i4vl7 | interferon induced with helicase C domain 1 |
| Ifih1 | S.c4 | interferon induced with helicase C domain 1 |
| Ifih1 | S.i1vl7 | interferon induced with helicase C domain 1 |
| Ifih1 | S.i2vl7 | interferon induced with helicase C domain 1 |
| Ifitm3 | S.c3 | interferon induced transmembrane protein 3 |
| Ifitm3 | S.i2vl7 | interferon induced transmembrane protein 3 |
| Ifitm3 | S.i4vl7 | interferon induced transmembrane protein 3 |
| Ifnar1 | S.i4vl7 | interferon (alpha and beta) receptor 1 |
| Ifnar1 | S.i1vl7 | interferon (alpha and beta) receptor 1 |
| Ifnar1 | S.i2vl7 | interferon (alpha and beta) receptor 1 |
| Ifnar1 | S.i3vl7 | interferon (alpha and beta) receptor 1 |
| Ifnar1 | S.c2 | interferon (alpha and beta) receptor 1 |
| Ifnar2 | S.c3 | interferon (alpha and beta) receptor 2 |
| Ifnar2 | S.i4vl7 | interferon (alpha and beta) receptor 2 |
| Ifngr2 | S.i2vl7 | interferon gamma receptor 2 |
| Ifngr2 | S.i1vl7 | interferon gamma receptor 2 |
| Ifngr2 | S.i3vl7 | interferon gamma receptor 2 |
| Ifngr2 | S.c6 | interferon gamma receptor 2 |
| Ifrd1 | S.c3 | interferon-related developmental regulator 1 |
| Ift20 | S.i1vl7 | intraflagellar transport 20 |
| Ift20 | S.c7 | intraflagellar transport 20 |
| Ift27 | S.c3 | intraflagellar transport 27 |
| Ift46 | S.c6 | intraflagellar transport 46 |
| Igf1 | S.c7 | insulin-like growth factor 1 |
| Igfals | S.c9 | insulin-like growth factor binding protein, acid labile subunit |
| Igfals | S.i4vl7 | insulin-like growth factor binding protein, acid labile subunit |
| Igfbp2 | S.c9 | insulin-like growth factor binding protein 2 |
| Igfbp3 | S.c7 | insulin-like growth factor binding protein 3 |
| Igfbp4 | S.i2vl7 | insulin-like growth factor binding protein 4 |
| Igfbp4 | S.c2 | insulin-like growth factor binding protein 4 |
| Igfbp4 | S.i3vl7 | insulin-like growth factor binding protein 4 |
| Igfbp5 | S.i3vl7 | insulin-like growth factor binding protein 5 |
| Igfbp5 | c5.i3vl7 | insulin-like growth factor binding protein 5 |
| Igfbp5 | S.i4vl7 | insulin-like growth factor binding protein 5 |
| Igfbp5 | S.i1vl7 | insulin-like growth factor binding protein 5 |
| Igfbp5 | S.i2vl7 | insulin-like growth factor binding protein 5 |
| Igfbp6 | S.c2 | insulin-like growth factor binding protein 6 |
| Igfbpl1 | S.i3vl7 | insulin-like growth factor binding protein-like 1 |
| Igfbpl1 | S.i4vl7 | insulin-like growth factor binding protein-like 1 |
| Igfbpl1 | S.i2vl7 | insulin-like growth factor binding protein-like 1 |
| Igh-VJ558 | S.c2 | immunoglobulin heavy chain (J558 family) |
| Igh-VJ558 | S.i2vl7 | immunoglobulin heavy chain (J558 family) |
| Igh-VJ558 | S.i3vl7 | immunoglobulin heavy chain (J558 family) |
| Igh-VJ558 | S.i4vl7 | immunoglobulin heavy chain (J558 family) |
| Igj | S.c2 | immunoglobulin joining chain |
| Igsf10 | S.i2vl7 | immunoglobulin superfamily, member 10 |
| Il10rb | S.i4vl7 | interleukin 10 receptor, beta |
| Il10rb | S.i3vl7 | interleukin 10 receptor, beta |
| Il10rb | S.c4 | interleukin 10 receptor, beta |
| Il10rb | S.i2vl7 | interleukin 10 receptor, beta |
| Il10rb | S.i1vl7 | interleukin 10 receptor, beta |
| Il1rn | S.c1 | interleukin 1 receptor antagonist |
| Il6st | S.i2vl7 | interleukin 6 signal transducer |
| Il6st | S.c2 | interleukin 6 signal transducer |
| Il6st | S.i4vl7 | interleukin 6 signal transducer |
| Il6st | S.i3vl7 | interleukin 6 signal transducer |
| Ilf3 | S.c4 | interleukin enhancer binding factor 3 |
| Ilf3 | S.i1vl7 | interleukin enhancer binding factor 3 |
| Ilf3 | S.i3vl7 | interleukin enhancer binding factor 3 |
| Ilf3 | S.i4vl7 | interleukin enhancer binding factor 3 |
| Ilf3 | S.i2vl7 | interleukin enhancer binding factor 3 |
| Ilkap | S.i2vl7 | integrin-linked kinase-associated serine/threonine phosphatase 2C |
| Ilkap | S.i4vl7 | integrin-linked kinase-associated serine/threonine phosphatase 2C |
| Ilkap | S.i3vl7 | integrin-linked kinase-associated serine/threonine phosphatase 2C |
| Ilkap | S.c4 | integrin-linked kinase-associated serine/threonine phosphatase 2C |
| Ilvbl | S.c3 | ilvB (bacterial acetolactate synthase)-like |
| Immt | S.i4vl7 | inner membrane protein, mitochondrial |
| Immt | S.c8 | inner membrane protein, mitochondrial |
| Impdh1 | S.c7 | inosine 5'-phosphate dehydrogenase 1 |
| Inhbb | c5.i3vl7 | inhibin beta-B |
| Inhbb | S.i1vl7 | inhibin beta-B |
| Inhbb | S.i3vl7 | inhibin beta-B |
| Inhbb | S.i4vl7 | inhibin beta-B |
| Inhbb | S.i2vl7 | inhibin beta-B |
| Ins2 | S.i4vl7 | insulin II |
| Ins2 | S.c8 | insulin II |
| Ins2 | S.i2vl7 | insulin II |
| Ipo11 | S.i1vl7 | importin 11 |
| Ipo11 | S.c6 | importin 11 |
| Iqgap1 | S.c4 | IQ motif containing GTPase activating protein 1 |
| Iqgap1 | c5.i3vl7 | IQ motif containing GTPase activating protein 1 |
| Iqgap1 | S.i1vl7 | IQ motif containing GTPase activating protein 1 |
| Iqgap1 | S.i3vl7 | IQ motif containing GTPase activating protein 1 |
| Iqgap1 | S.i4vl7 | IQ motif containing GTPase activating protein 1 |
| Iqgap1 | S.i2vl7 | IQ motif containing GTPase activating protein 1 |
| Irak1 | S.c7 | interleukin-1 receptor-associated kinase 1 |
| Irak1 | S.i1vl7 | interleukin-1 receptor-associated kinase 1 |
| Irak1 | S.i2vl7 | interleukin-1 receptor-associated kinase 1 |
| Irf5 | S.c1 | interferon regulatory factor 5 |
| Irf8 | S.i4vl7 | interferon regulatory factor 8 |
| Irf8 | S.c1 | interferon regulatory factor 8 |
| Irf9 | S.c4 | interferon regulatory factor 9 |
| Isg15 | S.c4 | ISG15 ubiquitin-like modifier |
| Isg20 | S.i1vl7 | interferon-stimulated protein |
| Isg20 | S.c7 | interferon-stimulated protein |
| Isg20 | S.i2vl7 | interferon-stimulated protein |
| Isg20 | S.i3vl7 | interferon-stimulated protein |
| Isg20 | S.i4vl7 | interferon-stimulated protein |
| Islr | S.c2 | immunoglobulin superfamily containing leucine-rich repeat |
| Islr | S.i2vl7 | immunoglobulin superfamily containing leucine-rich repeat |
| Isoc1 | S.i4vl7 | isochorismatase domain containing 1 |
| Isoc1 | S.c3 | isochorismatase domain containing 1 |
| Isoc1 | S.i3vl7 | isochorismatase domain containing 1 |
| Isyna1 | S.c2 | myo-inositol 1-phosphate synthase A1 |
| Isyna1 | S.i4vl7 | myo-inositol 1-phosphate synthase A1 |
| Isyna1 | S.i3vl7 | myo-inositol 1-phosphate synthase A1 |
| Itga3 | S.i3vl7 | integrin alpha 3 |
| Itga6 | S.c6 | integrin alpha 6 |
| Itga6 | S.i3vl7 | integrin alpha 6 |
| Itga6 | S.i1vl7 | integrin alpha 6 |
| Itga8 | c5.i3vl7 | integrin alpha 8 |
| Itga8 | S.i3vl7 | integrin alpha 8 |
| Itga8 | S.i1vl7 | integrin alpha 8 |
| Itgax | S.i4vl7 | integrin alpha X |
| Itgax | S.c1 | integrin alpha X |
| Itgb1 | S.i4vl7 | integrin beta 1 (fibronectin receptor beta) |
| Itgb1 | S.i3vl7 | integrin beta 1 (fibronectin receptor beta) |
| Itgb1 | S.i2vl7 | integrin beta 1 (fibronectin receptor beta) |
| Itgb1 | S.i1vl7 | integrin beta 1 (fibronectin receptor beta) |
| Itgb1 | S.c4 | integrin beta 1 (fibronectin receptor beta) |
| Itgb2 | S.c1 | integrin beta 2 |
| Itgb4 | S.i4vl7 | integrin beta 4 |
| Itgb4 | S.i1vl7 | integrin beta 4 |
| Itgb4 | S.i3vl7 | integrin beta 4 |
| Itgb4 | S.i2vl7 | integrin beta 4 |
| Itgb4 | c5.i3vl7 | integrin beta 4 |
| Itgb5 | S.i4vl7 | integrin beta 5 |
| Itgb5 | S.c2 | integrin beta 5 |
| Itih5 | S.i3vl7 | inter-alpha (globulin) inhibitor H5 |
| Itih5 | S.i4vl7 | inter-alpha (globulin) inhibitor H5 |
| Itih5 | S.c1 | inter-alpha (globulin) inhibitor H5 |
| Itm2a | S.c1 | integral membrane protein 2A |
| Itm2a | S.i4vl7 | integral membrane protein 2A |
| Itm2b | S.i1vl7 | integral membrane protein 2B |
| Itm2b | S.i2vl7 | integral membrane protein 2B |
| Itm2b | S.c3 | integral membrane protein 2B |
| Itm2b | S.i3vl7 | integral membrane protein 2B |
| Itm2b | S.i4vl7 | integral membrane protein 2B |
| Itm2c | S.i1vl7 | integral membrane protein 2C |
| Itm2c | S.c3 | integral membrane protein 2C |
| Itm2c | S.i2vl7 | integral membrane protein 2C |
| Itm2c | S.i4vl7 | integral membrane protein 2C |
| Itm2c | S.i3vl7 | integral membrane protein 2C |
| Itpk1 | S.c2 | inositol 1,3,4-triphosphate 5/6 kinase |
| Itpr2 | S.i3vl7 | inositol 1,4,5-triphosphate receptor 2 |
| Itpr2 | S.i2vl7 | inositol 1,4,5-triphosphate receptor 2 |
| Itpr2 | S.i4vl7 | inositol 1,4,5-triphosphate receptor 2 |
| Itpr2 | S.i1vl7 | inositol 1,4,5-triphosphate receptor 2 |
| Itpr2 | c5.i3vl7 | inositol 1,4,5-triphosphate receptor 2 |
| Ivd | S.i1vl7 | isovaleryl coenzyme A dehydrogenase |
| Ivd | S.c7 | isovaleryl coenzyme A dehydrogenase |
| Jmjd6 | S.c9 | jumonji domain containing 6 |
| Jtb | S.c8 | jumping translocation breakpoint |
| Junb | S.i3vl7 | jun B proto-oncogene |
| Junb | S.c2 | jun B proto-oncogene |
| Jund | S.c8 | jun D proto-oncogene |
| Kank3 | S.i3vl7 | KN motif and ankyrin repeat domains 3 |
| Kank3 | S.c3 | KN motif and ankyrin repeat domains 3 |
| Kank3 | S.i2vl7 | KN motif and ankyrin repeat domains 3 |
| Kank3 | S.i4vl7 | KN motif and ankyrin repeat domains 3 |
| Kcnj4 | S.i3vl7 | potassium inwardly-rectifying channel, subfamily J, member 4 |
| Kcnj4 | S.i2vl7 | potassium inwardly-rectifying channel, subfamily J, member 4 |
| Kcnj4 | S.i4vl7 | potassium inwardly-rectifying channel, subfamily J, member 4 |
| Kcnj9 | S.c8 | potassium inwardly-rectifying channel, subfamily J, member 9 |
| Kcnj9 | S.i4vl7 | potassium inwardly-rectifying channel, subfamily J, member 9 |
| Kcnk1 | S.i1vl7 | potassium channel, subfamily K, member 1 |
| Kcnk1 | S.i2vl7 | potassium channel, subfamily K, member 1 |
| Kcnk1 | S.c8 | potassium channel, subfamily K, member 1 |
| Kcnk1 | S.i3vl7 | potassium channel, subfamily K, member 1 |
| Kcnk1 | S.i4vl7 | potassium channel, subfamily K, member 1 |
| Kcnk2 | S.c1 | potassium channel, subfamily K, member 2 |
| Kcnn4 | S.c8 | potassium intermediate/small conductance calcium-activated channel, subfamily N, member 4 |
| Kctd10 | S.c3 | potassium channel tetramerisation domain containing 10 |
| Kctd10 | S.i2vl7 | potassium channel tetramerisation domain containing 10 |
| Kctd10 | S.i4vl7 | potassium channel tetramerisation domain containing 10 |
| Kctd10 | S.i3vl7 | potassium channel tetramerisation domain containing 10 |
| Kctd12 | S.i3vl7 | potassium channel tetramerisation domain containing 12 |
| Kctd12 | S.i4vl7 | potassium channel tetramerisation domain containing 12 |
| Kctd12 | S.i2vl7 | potassium channel tetramerisation domain containing 12 |
| Kctd12 | S.c3 | potassium channel tetramerisation domain containing 12 |
| Kdm3b | S.i4vl7 | KDM3B lysine (K)-specific demethylase 3B |
| Kifap3 | S.i4vl7 | kinesin-associated protein 3 |
| Kifap3 | S.i1vl7 | kinesin-associated protein 3 |
| Kifap3 | S.i2vl7 | kinesin-associated protein 3 |
| Kifap3 | S.c4 | kinesin-associated protein 3 |
| Kit | S.i3vl7 | kit oncogene |
| Kit | S.i2vl7 | kit oncogene |
| Kit | S.i4vl7 | kit oncogene |
| Kit | S.c4 | kit oncogene |
| Kit | S.i1vl7 | kit oncogene |
| Kitl | S.i4vl7 | kit ligand |
| Kitl | S.c2 | kit ligand |
| Klc1 | S.i3vl7 | kinesin light chain 1 |
| Klc1 | c5.i3vl7 | kinesin light chain 1 |
| Klc1 | S.i4vl7 | kinesin light chain 1 |
| Klc1 | S.i1vl7 | kinesin light chain 1 |
| Klc1 | S.i2vl7 | kinesin light chain 1 |
| Klf10 | S.i4vl7 | Kruppel-like factor 10 |
| Klf10 | S.c3 | Kruppel-like factor 10 |
| Klf10 | S.i2vl7 | Kruppel-like factor 10 |
| Klf10 | S.i3vl7 | Kruppel-like factor 10 |
| Klf4 | S.i4vl7 | Kruppel-like factor 4 (gut) |
| Klf4 | S.c3 | Kruppel-like factor 4 (gut) |
| Klf4 | S.i3vl7 | Kruppel-like factor 4 (gut) |
| Klf4 | S.i2vl7 | Kruppel-like factor 4 (gut) |
| Klf6 | S.i1vl7 | Kruppel-like factor 6 |
| Klf6 | S.c6 | Kruppel-like factor 6 |
| Klhdc2 | S.c6 | kelch domain containing 2 |
| Klhdc2 | S.i1vl7 | kelch domain containing 2 |
| Kmt2e | S.i4vl7 | lysine (K)-specific methyltransferase 2E |
| Kmt2e | S.i2vl7 | lysine (K)-specific methyltransferase 2E |
| Kmt2e | S.c3 | lysine (K)-specific methyltransferase 2E |
| Kmt2e | S.i3vl7 | lysine (K)-specific methyltransferase 2E |
| Knop1 | S.c6 | lysine rich nucleolar protein 1 |
| Kpna3 | S.i1vl7 | karyopherin (importin) alpha 3 |
| Kpna3 | S.i3vl7 | karyopherin (importin) alpha 3 |
| Kpna3 | S.i4vl7 | karyopherin (importin) alpha 3 |
| Kpna3 | S.c4 | karyopherin (importin) alpha 3 |
| Kpna3 | S.i2vl7 | karyopherin (importin) alpha 3 |
| Kpnb1 | S.i4vl7 | karyopherin (importin) beta 1 |
| Kpnb1 | S.c2 | karyopherin (importin) beta 1 |
| Krt18 | S.i2vl7 | keratin 18 |
| Krt18 | S.i1vl7 | keratin 18 |
| Krt18 | S.i3vl7 | keratin 18 |
| Krt18 | c5.i3vl7 | keratin 18 |
| Krt18 | S.i4vl7 | keratin 18 |
| Krt19 | S.i3vl7 | keratin 19 |
| Krt19 | S.c3 | keratin 19 |
| Krt19 | S.i2vl7 | keratin 19 |
| Krt19 | S.i4vl7 | keratin 19 |
| Krt25 | S.c7 | keratin 25 |
| Krt35 | S.c8 | keratin 35 |
| Krt7 | S.c4 | keratin 7 |
| Krt7 | S.i1vl7 | keratin 7 |
| Krt7 | S.i2vl7 | keratin 7 |
| Krt7 | S.i4vl7 | keratin 7 |
| Krt8 | S.c4 | keratin 8 |
| Krt8 | S.i1vl7 | keratin 8 |
| Krt8 | S.i2vl7 | keratin 8 |
| Krt8 | S.i3vl7 | keratin 8 |
| Krt8 | S.i4vl7 | keratin 8 |
| Krt81 | S.i1vl7 | keratin 81 |
| Krt81 | S.i4vl7 | keratin 81 |
| Krt81 | S.i2vl7 | keratin 81 |
| Krt81 | S.c8 | keratin 81 |
| Krt81 | S.i3vl7 | keratin 81 |
| Krt83 | S.c8 | keratin 83 |
| Krt83 | S.i4vl7 | keratin 83 |
| Krt83 | S.i2vl7 | keratin 83 |
| Krt83 | S.i3vl7 | keratin 83 |
| Krt83 | S.i1vl7 | keratin 83 |
| Krtcap2 | S.c7 | keratinocyte associated protein 2 |
| Krtcap2 | S.i4vl7 | keratinocyte associated protein 2 |
| Krtcap2 | S.i1vl7 | keratinocyte associated protein 2 |
| Lalba | S.i4vl7 | lactalbumin, alpha |
| Lalba | S.c9 | lactalbumin, alpha |
| Lama2 | S.c1 | laminin, alpha 2 |
| Lama3 | S.i3vl7 | laminin, alpha 3 |
| Lama3 | S.i2vl7 | laminin, alpha 3 |
| Lama3 | S.c2 | laminin, alpha 3 |
| Lama4 | S.i2vl7 | laminin, alpha 4 |
| Lama4 | S.c2 | laminin, alpha 4 |
| Lama4 | S.i4vl7 | laminin, alpha 4 |
| Lama4 | S.i3vl7 | laminin, alpha 4 |
| Lamb3 | S.c4 | laminin, beta 3 |
| Lamc2 | S.i1vl7 | laminin, gamma 2 |
| Lamc2 | S.i3vl7 | laminin, gamma 2 |
| Lamc2 | S.c4 | laminin, gamma 2 |
| Lamp1 | S.c3 | lysosomal-associated membrane protein 1 |
| Lamp2 | c5.i3vl7 | lysosomal-associated membrane protein 2 |
| Lamp2 | S.i2vl7 | lysosomal-associated membrane protein 2 |
| Lamp2 | S.i1vl7 | lysosomal-associated membrane protein 2 |
| Lamp2 | S.i3vl7 | lysosomal-associated membrane protein 2 |
| Lamtor2 | S.i1vl7 | late endosomal/lysosomal adaptor, MAPK and MTOR activator 2 |
| Lamtor2 | S.c7 | late endosomal/lysosomal adaptor, MAPK and MTOR activator 2 |
| Laptm4a | S.i3vl7 | lysosomal-associated protein transmembrane 4A |
| Laptm4a | c5.i3vl7 | lysosomal-associated protein transmembrane 4A |
| Laptm4a | S.i4vl7 | lysosomal-associated protein transmembrane 4A |
| Laptm4b | S.i2vl7 | lysosomal-associated protein transmembrane 4B |
| Laptm4b | S.i4vl7 | lysosomal-associated protein transmembrane 4B |
| Laptm4b | S.i1vl7 | lysosomal-associated protein transmembrane 4B |
| Laptm4b | S.i3vl7 | lysosomal-associated protein transmembrane 4B |
| Laptm4b | S.c3 | lysosomal-associated protein transmembrane 4B |
| Laptm5 | S.i4vl7 | lysosomal-associated protein transmembrane 5 |
| Laptm5 | S.c1 | lysosomal-associated protein transmembrane 5 |
| Laptm5 | S.c2 | lysosomal-associated protein transmembrane 5 |
| Las1l | S.i1vl7 | LAS1-like (S. cerevisiae) |
| Lasp1 | S.c4 | LIM and SH3 protein 1 |
| Lasp1 | S.i1vl7 | LIM and SH3 protein 1 |
| Lasp1 | S.i3vl7 | LIM and SH3 protein 1 |
| Lasp1 | S.i2vl7 | LIM and SH3 protein 1 |
| Lasp1 | S.i4vl7 | LIM and SH3 protein 1 |
| Lat2 | S.c1 | linker for activation of T cells family, member 2 |
| Lat2 | S.i4vl7 | linker for activation of T cells family, member 2 |
| Lats2 | S.c4 | large tumor suppressor 2 |
| Lbp | S.i1vl7 | lipopolysaccharide binding protein |
| Lbp | c5.i3vl7 | lipopolysaccharide binding protein |
| Lbp | S.i2vl7 | lipopolysaccharide binding protein |
| Lbp | S.i3vl7 | lipopolysaccharide binding protein |
| Lcp1 | S.i4vl7 | lymphocyte cytosolic protein 1 |
| Lcp1 | S.i2vl7 | lymphocyte cytosolic protein 1 |
| Lcp1 | S.i3vl7 | lymphocyte cytosolic protein 1 |
| Lcp1 | S.c3 | lymphocyte cytosolic protein 1 |
| Ldhb | S.c7 | lactate dehydrogenase B |
| Ldhb | S.i1vl7 | lactate dehydrogenase B |
| Ldlr | S.c7 | low density lipoprotein receptor |
| Lep | S.i4vl7 | leptin |
| Lep | S.i2vl7 | leptin |
| Lep | S.i1vl7 | leptin |
| Lep | S.i3vl7 | leptin |
| Leprot | S.i4vl7 | leptin receptor overlapping transcript |
| Leprot | S.i1vl7 | leptin receptor overlapping transcript |
| Leprot | S.i3vl7 | leptin receptor overlapping transcript |
| Leprot | S.i2vl7 | leptin receptor overlapping transcript |
| Leprot | S.c4 | leptin receptor overlapping transcript |
| Lgals1 | S.i1vl7 | lectin, galactose binding, soluble 1 |
| Lgals1 | S.c3 | lectin, galactose binding, soluble 1 |
| Lgals1 | S.i2vl7 | lectin, galactose binding, soluble 1 |
| Lgals1 | S.i4vl7 | lectin, galactose binding, soluble 1 |
| Lgals1 | S.i3vl7 | lectin, galactose binding, soluble 1 |
| Lgals3 | S.i4vl7 | lectin, galactose binding, soluble 3 |
| Lgals3 | S.c2 | lectin, galactose binding, soluble 3 |
| Lgals3bp | S.c2 | lectin, galactoside-binding, soluble, 3 binding protein |
| Lgals3bp | S.i4vl7 | lectin, galactoside-binding, soluble, 3 binding protein |
| Lgals3bp | S.i2vl7 | lectin, galactoside-binding, soluble, 3 binding protein |
| Lgals3bp | S.i3vl7 | lectin, galactoside-binding, soluble, 3 binding protein |
| Lgals9 | S.c4 | lectin, galactose binding, soluble 9 |
| Lgals9 | S.i4vl7 | lectin, galactose binding, soluble 9 |
| Lgals9 | S.i1vl7 | lectin, galactose binding, soluble 9 |
| Lgalsl | S.i3vl7 | lectin, galactoside binding-like |
| Lgalsl | S.i2vl7 | lectin, galactoside binding-like |
| Lgalsl | c5.i3vl7 | lectin, galactoside binding-like |
| Lgmn | S.i3vl7 | legumain |
| Lgmn | S.i4vl7 | legumain |
| Lgmn | S.c3 | legumain |
| Lgmn | S.i2vl7 | legumain |
| Lifr | S.c7 | leukemia inhibitory factor receptor |
| Lifr | S.i3vl7 | leukemia inhibitory factor receptor |
| Lifr | S.i4vl7 | leukemia inhibitory factor receptor |
| Lifr | S.i2vl7 | leukemia inhibitory factor receptor |
| Lilra6 | S.i3vl7 | leukocyte immunoglobulin-like receptor, subfamily A (with TM domain), member 6 |
| Lilra6 | S.c2 | leukocyte immunoglobulin-like receptor, subfamily A (with TM domain), member 6 |
| Lilrb4 | S.i4vl7 | leukocyte immunoglobulin-like receptor, subfamily B, member 4 |
| Lilrb4 | S.c1 | leukocyte immunoglobulin-like receptor, subfamily B, member 4 |
| Lima1 | S.i2vl7 | LIM domain and actin binding 1 |
| Lima1 | S.i4vl7 | LIM domain and actin binding 1 |
| Lima1 | S.i3vl7 | LIM domain and actin binding 1 |
| Lima1 | c5.i3vl7 | LIM domain and actin binding 1 |
| Lima1 | S.i1vl7 | LIM domain and actin binding 1 |
| Lims1 | S.i4vl7 | LIM and senescent cell antigen-like domains 1 |
| Lims1 | S.c4 | LIM and senescent cell antigen-like domains 1 |
| Lims1 | S.i2vl7 | LIM and senescent cell antigen-like domains 1 |
| Lin9 | S.c6 | lin-9 homolog (C. elegans) |
| Lipe | S.i4vl7 | lipase, hormone sensitive |
| Lipe | S.c2 | lipase, hormone sensitive |
| Lipe | S.i3vl7 | lipase, hormone sensitive |
| Litaf | S.i1vl7 | LPS-induced TN factor |
| Litaf | c5.i3vl7 | LPS-induced TN factor |
| Litaf | S.i3vl7 | LPS-induced TN factor |
| Litaf | S.i4vl7 | LPS-induced TN factor |
| Litaf | S.i2vl7 | LPS-induced TN factor |
| Llgl1 | S.c7 | lethal giant larvae homolog 1 (Drosophila) |
| Llgl2 | S.c8 | lethal giant larvae homolog 2 (Drosophila) |
| Llgl2 | S.i2vl7 | lethal giant larvae homolog 2 (Drosophila) |
| Llgl2 | S.i3vl7 | lethal giant larvae homolog 2 (Drosophila) |
| Llgl2 | S.i4vl7 | lethal giant larvae homolog 2 (Drosophila) |
| Lman1 | S.i2vl7 | lectin, mannose-binding, 1 |
| Lman1 | S.i1vl7 | lectin, mannose-binding, 1 |
| Lman1 | S.i4vl7 | lectin, mannose-binding, 1 |
| Lman1 | S.i3vl7 | lectin, mannose-binding, 1 |
| Lman1 | S.c7 | lectin, mannose-binding, 1 |
| Lman2 | S.c8 | lectin, mannose-binding 2 |
| Lmna | S.i3vl7 | lamin A |
| Lmna | S.c3 | lamin A |
| Lmna | S.i2vl7 | lamin A |
| Lmna | c5.i3vl7 | lamin A |
| Lmo2 | S.i3vl7 | LIM domain only 2 |
| Lmo2 | S.c7 | LIM domain only 2 |
| Lmo2 | S.i2vl7 | LIM domain only 2 |
| Lmo7 | S.c4 | LIM domain only 7 |
| Lmo7 | S.i4vl7 | LIM domain only 7 |
| LOC100041504 | S.c1 | c-C motif chemokine 21c-like |
| LOC100041593 | S.c1 | c-C motif chemokine 21c-like |
| LOC100862446 | S.i4vl7 | ferritin light chain 1-like |
| LOC101056061 | S.i1vl7 | enhancer of rudimentary homolog |
| Lonp2 | S.i4vl7 | lon peptidase 2, peroxisomal |
| Lox | S.c3 | lysyl oxidase |
| Lox | S.i4vl7 | lysyl oxidase |
| Lpar1 | S.i3vl7 | lysophosphatidic acid receptor 1 |
| Lpgat1 | S.c4 | lysophosphatidylglycerol acyltransferase 1 |
| Lpin1 | S.c7 | lipin 1 |
| Lpl | S.i2vl7 | lipoprotein lipase |
| Lpl | S.c7 | lipoprotein lipase |
| Lpl | S.i1vl7 | lipoprotein lipase |
| Lrch4 | S.c2 | leucine-rich repeats and calponin homology (CH) domain containing 4 |
| Lrg1 | c5.i3vl7 | leucine-rich alpha-2-glycoprotein 1 |
| Lrg1 | S.i2vl7 | leucine-rich alpha-2-glycoprotein 1 |
| Lrg1 | S.i1vl7 | leucine-rich alpha-2-glycoprotein 1 |
| Lrg1 | S.i3vl7 | leucine-rich alpha-2-glycoprotein 1 |
| Lrg1 | S.i4vl7 | leucine-rich alpha-2-glycoprotein 1 |
| Lrp1 | S.i4vl7 | low density lipoprotein receptor-related protein 1 |
| Lrp1 | S.c1 | low density lipoprotein receptor-related protein 1 |
| Lrp10 | S.c2 | low-density lipoprotein receptor-related protein 10 |
| Lrp10 | S.i3vl7 | low-density lipoprotein receptor-related protein 10 |
| Lrpap1 | S.c1 | low density lipoprotein receptor-related protein associated protein 1 |
| Lrpprc | S.i1vl7 | leucine-rich PPR-motif containing |
| Lsm14a | S.c3 | LSM14 homolog A (SCD6, S. cerevisiae) |
| Lsm3 | S.c4 | LSM3 homolog, U6 small nuclear RNA associated (S. cerevisiae) |
| Lsm3 | S.i1vl7 | LSM3 homolog, U6 small nuclear RNA associated (S. cerevisiae) |
| Lsm8 | S.i3vl7 | LSM8 homolog, U6 small nuclear RNA associated (S. cerevisiae) |
| Lsm8 | S.c4 | LSM8 homolog, U6 small nuclear RNA associated (S. cerevisiae) |
| Lsm8 | S.i2vl7 | LSM8 homolog, U6 small nuclear RNA associated (S. cerevisiae) |
| Lsm8 | S.i1vl7 | LSM8 homolog, U6 small nuclear RNA associated (S. cerevisiae) |
| Lsm8 | S.i4vl7 | LSM8 homolog, U6 small nuclear RNA associated (S. cerevisiae) |
| Lsp1 | S.i3vl7 | lymphocyte specific 1 |
| Lsp1 | S.i2vl7 | lymphocyte specific 1 |
| Lsp1 | S.c2 | lymphocyte specific 1 |
| Lsr | S.i2vl7 | lipolysis stimulated lipoprotein receptor |
| Lsr | c5.i3vl7 | lipolysis stimulated lipoprotein receptor |
| Lsr | S.i4vl7 | lipolysis stimulated lipoprotein receptor |
| Lsr | S.i3vl7 | lipolysis stimulated lipoprotein receptor |
| Lsr | S.i1vl7 | lipolysis stimulated lipoprotein receptor |
| Lss | S.c7 | lanosterol synthase |
| Lst1 | S.i4vl7 | leukocyte specific transcript 1 |
| Lst1 | S.i3vl7 | leukocyte specific transcript 1 |
| Lst1 | S.c2 | leukocyte specific transcript 1 |
| Lta4h | S.i4vl7 | leukotriene A4 hydrolase |
| Lta4h | S.i1vl7 | leukotriene A4 hydrolase |
| Lta4h | S.c4 | leukotriene A4 hydrolase |
| Ltbp4 | S.c1 | latent transforming growth factor beta binding protein 4 |
| Ltbr | S.i4vl7 | lymphotoxin B receptor |
| Ltbr | S.i3vl7 | lymphotoxin B receptor |
| Ltbr | c5.i3vl7 | lymphotoxin B receptor |
| Ltc4s | S.c1 | leukotriene C4 synthase |
| Ltf | c5.i3vl7 | lactotransferrin |
| Ltf | S.i1vl7 | lactotransferrin |
| Ltf | S.i4vl7 | lactotransferrin |
| Ltf | S.i3vl7 | lactotransferrin |
| Ltf | S.i2vl7 | lactotransferrin |
| Lum | S.i3vl7 | lumican |
| Lum | S.c3 | lumican |
| Lum | S.i4vl7 | lumican |
| Lxn | S.i1vl7 | latexin |
| Lxn | S.c4 | latexin |
| Ly6e | S.i3vl7 | lymphocyte antigen 6 complex, locus E |
| Ly6e | S.c8 | lymphocyte antigen 6 complex, locus E |
| Ly6e | S.i1vl7 | lymphocyte antigen 6 complex, locus E |
| Ly6e | S.c3 | lymphocyte antigen 6 complex, locus E |
| Ly6e | S.i4vl7 | lymphocyte antigen 6 complex, locus E |
| Ly86 | S.c1 | lymphocyte antigen 86 |
| Ly86 | S.i4vl7 | lymphocyte antigen 86 |
| Lyl1 | S.i4vl7 | lymphoblastomic leukemia 1 |
| Lyl1 | S.c1 | lymphoblastomic leukemia 1 |
| Lyrm2 | S.i2vl7 | LYR motif containing 2 |
| Lyrm2 | S.i3vl7 | LYR motif containing 2 |
| Lyrm2 | S.i4vl7 | LYR motif containing 2 |
| Lyrm2 | S.i1vl7 | LYR motif containing 2 |
| Lyrm2 | S.c6 | LYR motif containing 2 |
| Lysmd2 | S.c1 | LysM, putative peptidoglycan-binding, domain containing 2 |
| Lyz1 | S.i3vl7 | lysozyme 1 |
| Lyz1 | S.i4vl7 | lysozyme 1 |
| Lyz1 | S.c2 | lysozyme 1 |
| Lyz2 | S.i3vl7 | lysozyme 2 |
| Lyz2 | S.c2 | lysozyme 2 |
| Lyz2 | S.i4vl7 | lysozyme 2 |
| Lyz2 | S.i2vl7 | lysozyme 2 |
| M6pr | S.i2vl7 | mannose-6-phosphate receptor, cation dependent |
| M6pr | S.c4 | mannose-6-phosphate receptor, cation dependent |
| M6pr | S.i3vl7 | mannose-6-phosphate receptor, cation dependent |
| M6pr | S.i4vl7 | mannose-6-phosphate receptor, cation dependent |
| Mad2l1bp | S.c9 | MAD2L1 binding protein |
| Mafb | S.c2 | v-maf musculoaponeurotic fibrosarcoma oncogene family, protein B (avian) |
| Maged1 | S.c2 | melanoma antigen, family D, 1 |
| Maged1 | S.i4vl7 | melanoma antigen, family D, 1 |
| Magoh | S.c4 | mago-nashi homolog, proliferation-associated (Drosophila) |
| Man1a | S.c2 | mannosidase 1, alpha |
| Man1a | S.i4vl7 | mannosidase 1, alpha |
| Man1a | S.i2vl7 | mannosidase 1, alpha |
| Man1a | S.i3vl7 | mannosidase 1, alpha |
| Man1b1 | S.c3 | mannosidase, alpha, class 1B, member 1 |
| Man2b1 | S.i4vl7 | mannosidase 2, alpha B1 |
| Man2b1 | S.c2 | mannosidase 2, alpha B1 |
| Man2b1 | S.i3vl7 | mannosidase 2, alpha B1 |
| Manf | S.c2 | mesencephalic astrocyte-derived neurotrophic factor |
| Map1lc3a | S.i1vl7 | microtubule-associated protein 1 light chain 3 alpha |
| Map1lc3b | S.c6 | microtubule-associated protein 1 light chain 3 beta |
| Map1lc3b | S.i1vl7 | microtubule-associated protein 1 light chain 3 beta |
| Map3k1 | S.c6 | mitogen-activated protein kinase kinase kinase 1 |
| Map4k3 | S.i4vl7 | mitogen-activated protein kinase kinase kinase kinase 3 |
| Map4k3 | S.c3 | mitogen-activated protein kinase kinase kinase kinase 3 |
| Map4k4 | S.i4vl7 | mitogen-activated protein kinase kinase kinase kinase 4 |
| Map4k4 | c5.i3vl7 | mitogen-activated protein kinase kinase kinase kinase 4 |
| Map4k4 | S.i2vl7 | mitogen-activated protein kinase kinase kinase kinase 4 |
| Map4k4 | S.i1vl7 | mitogen-activated protein kinase kinase kinase kinase 4 |
| Map4k4 | S.i3vl7 | mitogen-activated protein kinase kinase kinase kinase 4 |
| Map4k4 | S.c4 | mitogen-activated protein kinase kinase kinase kinase 4 |
| Map7d1 | S.c7 | MAP7 domain containing 1 |
| Mapk1 | S.c9 | mitogen-activated protein kinase 1 |
| Mapk14 | S.c4 | mitogen-activated protein kinase 14 |
| Mapk8ip1 | S.i4vl7 | mitogen-activated protein kinase 8 interacting protein 1 |
| Mapkapk2 | S.i4vl7 | MAP kinase-activated protein kinase 2 |
| Mapkapk2 | S.i2vl7 | MAP kinase-activated protein kinase 2 |
| Mapkapk2 | c5.i3vl7 | MAP kinase-activated protein kinase 2 |
| Mapkapk2 | S.c3 | MAP kinase-activated protein kinase 2 |
| Mapkapk2 | S.i3vl7 | MAP kinase-activated protein kinase 2 |
| Mapre1 | S.c4 | microtubule-associated protein, RP/EB family, member 1 |
| Mapre1 | S.i3vl7 | microtubule-associated protein, RP/EB family, member 1 |
| Mapre1 | S.i4vl7 | microtubule-associated protein, RP/EB family, member 1 |
| Mapre1 | S.i2vl7 | microtubule-associated protein, RP/EB family, member 1 |
| Mapre1 | S.i1vl7 | microtubule-associated protein, RP/EB family, member 1 |
| 6-Mar | S.i4vl7 | membrane-associated ring finger (C3HC4) 6 |
| 6-Mar | S.i3vl7 | membrane-associated ring finger (C3HC4) 6 |
| 6-Mar | S.c3 | membrane-associated ring finger (C3HC4) 6 |
| 7-Mar | S.c3 | membrane-associated ring finger (C3HC4) 7 |
| Marcks | S.c3 | myristoylated alanine rich protein kinase C substrate |
| Marcks | S.i4vl7 | myristoylated alanine rich protein kinase C substrate |
| Marcksl1 | S.i4vl7 | MARCKS-like 1 |
| Marcksl1 | S.i2vl7 | MARCKS-like 1 |
| Marcksl1 | c5.i3vl7 | MARCKS-like 1 |
| Marcksl1 | S.i3vl7 | MARCKS-like 1 |
| Marcksl1 | S.i1vl7 | MARCKS-like 1 |
| Mark2 | S.i2vl7 | MAP/microtubule affinity-regulating kinase 2 |
| Mark2 | S.i3vl7 | MAP/microtubule affinity-regulating kinase 2 |
| Mark2 | S.i4vl7 | MAP/microtubule affinity-regulating kinase 2 |
| Mark2 | S.c2 | MAP/microtubule affinity-regulating kinase 2 |
| Mark3 | S.i4vl7 | MAP/microtubule affinity-regulating kinase 3 |
| Matn2 | S.c1 | matrilin 2 |
| Matr3 | S.i4vl7 | matrin 3 |
| Matr3 | S.c4 | matrin 3 |
| Matr3 | S.i3vl7 | matrin 3 |
| Max | S.i4vl7 | Max protein |
| Max | c5.i3vl7 | Max protein |
| Max | S.i3vl7 | Max protein |
| Mbd1 | S.i1vl7 | methyl-CpG binding domain protein 1 |
| Mbnl1 | S.c3 | muscleblind-like 1 (Drosophila) |
| Mbnl1 | S.i4vl7 | muscleblind-like 1 (Drosophila) |
| Mbnl1 | S.i2vl7 | muscleblind-like 1 (Drosophila) |
| Mbnl1 | S.i3vl7 | muscleblind-like 1 (Drosophila) |
| Mbnl1 | S.i1vl7 | muscleblind-like 1 (Drosophila) |
| Mbp | S.c6 | myelin basic protein |
| Mcg1038069 | S.c4 | ubiquitin-conjugating enzyme E2 N-like |
| Mcm7 | S.c3 | minichromosome maintenance deficient 7 (S. cerevisiae) |
| Mcrs1 | S.c4 | microspherule protein 1 |
| Mcrs1 | S.i4vl7 | microspherule protein 1 |
| Mdfi | S.c8 | MyoD family inhibitor |
| Mdfi | S.i2vl7 | MyoD family inhibitor |
| Mdfi | S.i1vl7 | MyoD family inhibitor |
| Mdfi | S.i3vl7 | MyoD family inhibitor |
| Mdfi | S.i4vl7 | MyoD family inhibitor |
| Mdk | S.c1 | midkine |
| Me1 | S.c7 | malic enzyme 1, NADP(+)-dependent, cytosolic |
| Med10 | S.c4 | mediator complex subunit 10 |
| Med10 | S.i1vl7 | mediator complex subunit 10 |
| Med12 | S.i1vl7 | mediator complex subunit 12 |
| Med12 | S.i3vl7 | mediator complex subunit 12 |
| Med12 | c5.i3vl7 | mediator complex subunit 12 |
| Med12 | S.i2vl7 | mediator complex subunit 12 |
| Med12l | S.i2vl7 | mediator complex subunit 12-like |
| Med22 | S.c7 | mediator complex subunit 22 |
| Med27 | S.c8 | mediator complex subunit 27 |
| Med28 | S.c3 | mediator complex subunit 28 |
| Med28 | S.i3vl7 | mediator complex subunit 28 |
| Med28 | S.i2vl7 | mediator complex subunit 28 |
| Med28 | S.i4vl7 | mediator complex subunit 28 |
| Med28 | S.i1vl7 | mediator complex subunit 28 |
| Mef2a | S.c4 | myocyte enhancer factor 2A |
| Mef2d | S.c3 | myocyte enhancer factor 2D |
| Men1 | S.c7 | multiple endocrine neoplasia 1 |
| Mep1b | S.c8 | meprin 1 beta |
| Mep1b | S.i2vl7 | meprin 1 beta |
| Mep1b | S.i4vl7 | meprin 1 beta |
| Mettl9 | S.i1vl7 | methyltransferase like 9 |
| Mettl9 | S.c6 | methyltransferase like 9 |
| Mfap2 | S.c1 | microfibrillar-associated protein 2 |
| Mfap5 | S.i4vl7 | microfibrillar associated protein 5 |
| Mfap5 | S.c4 | microfibrillar associated protein 5 |
| Mfap5 | S.i2vl7 | microfibrillar associated protein 5 |
| Mfhas1 | S.c7 | malignant fibrous histiocytoma amplified sequence 1 |
| Mfhas1 | S.i3vl7 | malignant fibrous histiocytoma amplified sequence 1 |
| Mfhas1 | S.i4vl7 | malignant fibrous histiocytoma amplified sequence 1 |
| Mfhas1 | S.i2vl7 | malignant fibrous histiocytoma amplified sequence 1 |
| Mfhas1 | S.i1vl7 | malignant fibrous histiocytoma amplified sequence 1 |
| Mfsd1 | S.i1vl7 | major facilitator superfamily domain containing 1 |
| Mfsd1 | S.i3vl7 | major facilitator superfamily domain containing 1 |
| Mfsd1 | S.c4 | major facilitator superfamily domain containing 1 |
| Mfsd1 | S.i4vl7 | major facilitator superfamily domain containing 1 |
| Mfsd4 | S.i4vl7 | major facilitator superfamily domain containing 4 |
| Mfsd4 | S.c8 | major facilitator superfamily domain containing 4 |
| Mgll | S.c3 | monoglyceride lipase |
| Mgll | S.i4vl7 | monoglyceride lipase |
| Mgll | S.i3vl7 | monoglyceride lipase |
| Mgll | S.i2vl7 | monoglyceride lipase |
| Mgp | S.i3vl7 | matrix Gla protein |
| Mgp | S.c2 | matrix Gla protein |
| Mgp | S.c1 | matrix Gla protein |
| Mgst1 | S.i2vl7 | microsomal glutathione S-transferase 1 |
| Mgst3 | S.c3 | microsomal glutathione S-transferase 3 |
| Mgst3 | S.i2vl7 | microsomal glutathione S-transferase 3 |
| Mgst3 | S.i4vl7 | microsomal glutathione S-transferase 3 |
| Mgst3 | S.i3vl7 | microsomal glutathione S-transferase 3 |
| Mid1ip1 | S.c1 | Mid1 interacting protein 1 (gastrulation specific G12-like (zebrafish)) |
| Midn | c5.i3vl7 | midnolin |
| Midn | S.i3vl7 | midnolin |
| Mien1 | S.i4vl7 | migration and invasion enhancer 1 |
| Mien1 | S.c8 | migration and invasion enhancer 1 |
| Miip | S.c8 | migration and invasion inhibitory protein |
| Miip | S.i4vl7 | migration and invasion inhibitory protein |
| Mki67 | S.c7 | antigen identified by monoclonal antibody Ki 67 |
| Mki67 | S.i2vl7 | antigen identified by monoclonal antibody Ki 67 |
| Mki67 | S.i4vl7 | antigen identified by monoclonal antibody Ki 67 |
| Mki67 | S.i1vl7 | antigen identified by monoclonal antibody Ki 67 |
| Mki67 | S.i3vl7 | antigen identified by monoclonal antibody Ki 67 |
| Mknk2 | S.c7 | MAP kinase-interacting serine/threonine kinase 2 |
| Mkrn1 | S.i2vl7 | makorin, ring finger protein, 1 |
| Mkrn1 | c5.i3vl7 | makorin, ring finger protein, 1 |
| Mkrn1 | S.i3vl7 | makorin, ring finger protein, 1 |
| Mkrn1 | S.i4vl7 | makorin, ring finger protein, 1 |
| Mkrn1 | S.i1vl7 | makorin, ring finger protein, 1 |
| Mlph | S.c7 | melanophilin |
| Mlst8 | S.c8 | MTOR associated protein, LST8 homolog (S. cerevisiae) |
| Mlx | S.c8 | MAX-like protein X |
| Mmp11 | S.c1 | matrix metallopeptidase 11 |
| Mmp12 | S.i4vl7 | matrix metallopeptidase 12 |
| Mmp12 | S.c1 | matrix metallopeptidase 12 |
| Mmp14 | S.c2 | matrix metallopeptidase 14 (membrane-inserted) |
| Mmp3 | S.c2 | matrix metallopeptidase 3 |
| Mmp3 | S.i4vl7 | matrix metallopeptidase 3 |
| Mmp3 | S.i3vl7 | matrix metallopeptidase 3 |
| Mmp3 | S.i2vl7 | matrix metallopeptidase 3 |
| Mnda | S.c3 | myeloid cell nuclear differentiation antigen |
| Mnda | S.i4vl7 | myeloid cell nuclear differentiation antigen |
| Mndal | S.c3 | myeloid nuclear differentiation antigen like |
| Mndal | S.i4vl7 | myeloid nuclear differentiation antigen like |
| Mob1a | S.i3vl7 | MOB kinase activator 1A |
| Mob1a | S.c2 | MOB kinase activator 1A |
| Mob1a | S.i1vl7 | MOB kinase activator 1A |
| Mob1a | S.c3 | MOB kinase activator 1A |
| Mob1a | S.i4vl7 | MOB kinase activator 1A |
| Mob1a | S.i2vl7 | MOB kinase activator 1A |
| Mob1b | S.i4vl7 | MOB kinase activator 1B |
| Mob1b | S.c8 | MOB kinase activator 1B |
| Mob2 | S.i2vl7 | MOB kinase activator 2 |
| Mob2 | S.c9 | MOB kinase activator 2 |
| Mob2 | S.i4vl7 | MOB kinase activator 2 |
| Mocos | S.c6 | molybdenum cofactor sulfurase |
| Mocos | S.i1vl7 | molybdenum cofactor sulfurase |
| Mpdu1 | S.i1vl7 | mannose-P-dolichol utilization defect 1 |
| Mpdu1 | S.c6 | mannose-P-dolichol utilization defect 1 |
| Mpeg1 | S.c2 | macrophage expressed gene 1 |
| Mpeg1 | S.i3vl7 | macrophage expressed gene 1 |
| Mpeg1 | S.i4vl7 | macrophage expressed gene 1 |
| Mphosph10 | S.c8 | M-phase phosphoprotein 10 (U3 small nucleolar ribonucleoprotein) |
| Mrap | S.i2vl7 | melanocortin 2 receptor accessory protein |
| Mrap | S.i3vl7 | melanocortin 2 receptor accessory protein |
| Mrap | S.i4vl7 | melanocortin 2 receptor accessory protein |
| Mrap | S.c3 | melanocortin 2 receptor accessory protein |
| Mrc1 | S.i4vl7 | mannose receptor, C type 1 |
| Mrc1 | S.c2 | mannose receptor, C type 1 |
| Mroh1 | S.i2vl7 | maestro heat-like repeat family member 1 |
| Mroh1 | S.i3vl7 | maestro heat-like repeat family member 1 |
| Mrpl12 | S.c8 | mitochondrial ribosomal protein L12 |
| Mrpl12 | S.i3vl7 | mitochondrial ribosomal protein L12 |
| Mrpl12 | S.i4vl7 | mitochondrial ribosomal protein L12 |
| Mrpl27 | S.c4 | mitochondrial ribosomal protein L27 |
| Mrpl27 | S.i1vl7 | mitochondrial ribosomal protein L27 |
| Mrpl27 | S.i4vl7 | mitochondrial ribosomal protein L27 |
| Mrpl36 | S.c3 | mitochondrial ribosomal protein L36 |
| Mrpl36 | S.i4vl7 | mitochondrial ribosomal protein L36 |
| Mrpl46 | S.c8 | mitochondrial ribosomal protein L46 |
| Mrpl9 | S.c6 | mitochondrial ribosomal protein L9 |
| Mrpl9 | S.i1vl7 | mitochondrial ribosomal protein L9 |
| Mrps15 | S.i3vl7 | mitochondrial ribosomal protein S15 |
| Mrps15 | S.i4vl7 | mitochondrial ribosomal protein S15 |
| Mrps15 | S.c3 | mitochondrial ribosomal protein S15 |
| Mrps18a | S.i4vl7 | mitochondrial ribosomal protein S18A |
| Mrps18a | S.i2vl7 | mitochondrial ribosomal protein S18A |
| Mrps18a | S.i3vl7 | mitochondrial ribosomal protein S18A |
| Mrps18a | S.c8 | mitochondrial ribosomal protein S18A |
| Ms4a6c | S.i4vl7 | membrane-spanning 4-domains, subfamily A, member 6C |
| Ms4a6c | S.c2 | membrane-spanning 4-domains, subfamily A, member 6C |
| Msn | S.i2vl7 | moesin |
| Msn | S.i3vl7 | moesin |
| Msn | S.c4 | moesin |
| Msn | S.i1vl7 | moesin |
| Msn | S.i4vl7 | moesin |
| Mt1 | S.c6 | metallothionein 1 |
| Mt1 | S.i1vl7 | metallothionein 1 |
| Mt2 | S.c4 | metallothionein 2 |
| Mtf2 | S.i4vl7 | metal response element binding transcription factor 2 |
| Mtf2 | S.c8 | metal response element binding transcription factor 2 |
| Mthfs | S.i4vl7 | 5, 10-methenyltetrahydrofolate synthetase |
| Mthfs | S.c1 | 5, 10-methenyltetrahydrofolate synthetase |
| Muc1 | S.c9 | mucin 1, transmembrane |
| Mvd | S.c7 | mevalonate (diphospho) decarboxylase |
| Mvk | S.c7 | mevalonate kinase |
| Mvk | S.i3vl7 | mevalonate kinase |
| Mvp | S.i3vl7 | major vault protein |
| Mvp | S.c4 | major vault protein |
| Mvp | S.i2vl7 | major vault protein |
| Mxi1 | S.i1vl7 | Max interacting protein 1 |
| Mxi1 | S.c6 | Max interacting protein 1 |
| Myadm | S.i4vl7 | myeloid-associated differentiation marker |
| Myadm | S.c3 | myeloid-associated differentiation marker |
| Myadm | S.i3vl7 | myeloid-associated differentiation marker |
| Myd88 | S.c4 | myeloid differentiation primary response gene 88 |
| Myh4 | S.c1 | myosin, heavy polypeptide 4, skeletal muscle |
| Myh9 | S.c2 | myosin, heavy polypeptide 9, non-muscle |
| Myh9 | S.i4vl7 | myosin, heavy polypeptide 9, non-muscle |
| Myh9 | S.i2vl7 | myosin, heavy polypeptide 9, non-muscle |
| Myh9 | S.c3 | myosin, heavy polypeptide 9, non-muscle |
| Myh9 | S.i1vl7 | myosin, heavy polypeptide 9, non-muscle |
| Myh9 | S.i3vl7 | myosin, heavy polypeptide 9, non-muscle |
| Myl1 | S.c1 | myosin, light polypeptide 1 |
| Myl12a | S.c4 | myosin, light chain 12A, regulatory, non-sarcomeric |
| Myl12a | S.i2vl7 | myosin, light chain 12A, regulatory, non-sarcomeric |
| Myl12a | S.i1vl7 | myosin, light chain 12A, regulatory, non-sarcomeric |
| Myl12a | S.i3vl7 | myosin, light chain 12A, regulatory, non-sarcomeric |
| Myl12a | S.i4vl7 | myosin, light chain 12A, regulatory, non-sarcomeric |
| Myl12b | S.i4vl7 | myosin, light chain 12B, regulatory |
| Myl12b | S.i3vl7 | myosin, light chain 12B, regulatory |
| Myl12b | S.i1vl7 | myosin, light chain 12B, regulatory |
| Myl12b | S.c4 | myosin, light chain 12B, regulatory |
| Myl12b | S.i2vl7 | myosin, light chain 12B, regulatory |
| Mylk | S.c6 | myosin, light polypeptide kinase |
| Myo10 | S.i4vl7 | myosin X |
| Myo10 | S.c3 | myosin X |
| Myo10 | S.i2vl7 | myosin X |
| Myo1f | S.c1 | myosin IF |
| Myo5b | S.c6 | myosin VB |
| Myo5b | S.i1vl7 | myosin VB |
| Myog | S.c7 | myogenin |
| Myt1l | S.i3vl7 | myelin transcription factor 1-like |
| Myt1l | S.i2vl7 | myelin transcription factor 1-like |
| Myt1l | S.i4vl7 | myelin transcription factor 1-like |
| Myt1l | S.i1vl7 | myelin transcription factor 1-like |
| Myt1l | S.c7 | myelin transcription factor 1-like |
| Nab2 | S.c2 | Ngfi-A binding protein 2 |
| Nab2 | S.i3vl7 | Ngfi-A binding protein 2 |
| Nabp1 | S.c6 | nucleic acid binding protein 1 |
| Nacc2 | S.c3 | nucleus accumbens associated 2, BEN and BTB (POZ) domain containing |
| Nae1 | S.c6 | NEDD8 activating enzyme E1 subunit 1 |
| Nampt | S.i2vl7 | nicotinamide phosphoribosyltransferase |
| Nampt | S.c7 | nicotinamide phosphoribosyltransferase |
| Nampt | S.i4vl7 | nicotinamide phosphoribosyltransferase |
| Nampt | S.i3vl7 | nicotinamide phosphoribosyltransferase |
| Nans | S.i1vl7 | N-acetylneuraminic acid synthase (sialic acid synthase) |
| Nans | S.c8 | N-acetylneuraminic acid synthase (sialic acid synthase) |
| Nans | S.i4vl7 | N-acetylneuraminic acid synthase (sialic acid synthase) |
| Nap1l1 | S.c4 | nucleosome assembly protein 1-like 1 |
| Nap1l1 | S.i4vl7 | nucleosome assembly protein 1-like 1 |
| Nap1l1 | S.i3vl7 | nucleosome assembly protein 1-like 1 |
| Nap1l1 | S.i1vl7 | nucleosome assembly protein 1-like 1 |
| Nap1l1 | S.i2vl7 | nucleosome assembly protein 1-like 1 |
| Nbl1 | S.c1 | neuroblastoma, suppression of tumorigenicity 1 |
| Nbr1 | S.i4vl7 | neighbor of Brca1 gene 1 |
| Nbr1 | S.c8 | neighbor of Brca1 gene 1 |
| Nbr1 | S.i3vl7 | neighbor of Brca1 gene 1 |
| Nbr1 | S.i1vl7 | neighbor of Brca1 gene 1 |
| Ncam1 | S.c8 | neural cell adhesion molecule 1 |
| Ncam1 | S.i2vl7 | neural cell adhesion molecule 1 |
| Ncam1 | S.i3vl7 | neural cell adhesion molecule 1 |
| Ncam1 | S.i4vl7 | neural cell adhesion molecule 1 |
| Ncdn | S.i1vl7 | neurochondrin |
| Ncdn | S.i2vl7 | neurochondrin |
| Ncf2 | S.c1 | neutrophil cytosolic factor 2 |
| Ncf4 | S.i4vl7 | neutrophil cytosolic factor 4 |
| Ncf4 | S.c1 | neutrophil cytosolic factor 4 |
| Nckap1 | S.c4 | NCK-associated protein 1 |
| Nckap1 | S.i1vl7 | NCK-associated protein 1 |
| Ncl | S.i3vl7 | nucleolin |
| Ncl | S.i1vl7 | nucleolin |
| Ncl | S.c3 | nucleolin |
| Ncl | S.i4vl7 | nucleolin |
| Ncl | S.i2vl7 | nucleolin |
| Ncoa4 | S.i4vl7 | nuclear receptor coactivator 4 |
| Ncoa4 | S.c2 | nuclear receptor coactivator 4 |
| Ncor2 | S.c2 | nuclear receptor co-repressor 2 |
| Nde1 | S.i4vl7 | nuclear distribution gene E homolog 1 (A nidulans) |
| Nde1 | S.c2 | nuclear distribution gene E homolog 1 (A nidulans) |
| Nde1 | S.i2vl7 | nuclear distribution gene E homolog 1 (A nidulans) |
| Ndel1 | c5.i3vl7 | nuclear distribution gene E-like homolog 1 (A. nidulans) |
| Ndel1 | S.i3vl7 | nuclear distribution gene E-like homolog 1 (A. nidulans) |
| Ndel1 | S.i4vl7 | nuclear distribution gene E-like homolog 1 (A. nidulans) |
| Ndn | S.i4vl7 | necdin |
| Ndn | S.i2vl7 | necdin |
| Ndn | S.i3vl7 | necdin |
| Ndn | S.c3 | necdin |
| Ndrg2 | S.c1 | N-myc downstream regulated gene 2 |
| Ndufa1 | S.c7 | NADH dehydrogenase (ubiquinone) 1 alpha subcomplex, 1 |
| Ndufa6 | S.i2vl7 | NADH dehydrogenase (ubiquinone) 1 alpha subcomplex, 6 (B14) |
| Ndufaf4 | S.c7 | NADH dehydrogenase (ubiquinone) 1 alpha subcomplex, assembly factor 4 |
| Ndufaf4 | S.i2vl7 | NADH dehydrogenase (ubiquinone) 1 alpha subcomplex, assembly factor 4 |
| Ndufb2 | S.i3vl7 | NADH dehydrogenase (ubiquinone) 1 beta subcomplex, 2 |
| Ndufb2 | S.c7 | NADH dehydrogenase (ubiquinone) 1 beta subcomplex, 2 |
| Ndufb8 | S.c7 | NADH dehydrogenase (ubiquinone) 1 beta subcomplex 8 |
| Ndufb8 | S.i1vl7 | NADH dehydrogenase (ubiquinone) 1 beta subcomplex 8 |
| Ndufv3 | S.c7 | NADH dehydrogenase (ubiquinone) flavoprotein 3 |
| Ndufv3 | S.i1vl7 | NADH dehydrogenase (ubiquinone) flavoprotein 3 |
| Necap1 | S.c9 | NECAP endocytosis associated 1 |
| Necap2 | S.c3 | NECAP endocytosis associated 2 |
| Nedd4 | S.i2vl7 | neural precursor cell expressed, developmentally down-regulated 4 |
| Nedd4 | S.c4 | neural precursor cell expressed, developmentally down-regulated 4 |
| Nedd4 | S.i4vl7 | neural precursor cell expressed, developmentally down-regulated 4 |
| Nedd4 | S.i1vl7 | neural precursor cell expressed, developmentally down-regulated 4 |
| Nedd4 | S.i3vl7 | neural precursor cell expressed, developmentally down-regulated 4 |
| Nedd4l | S.i2vl7 | neural precursor cell expressed, developmentally down-regulated gene 4-like |
| Nedd9 | S.c3 | neural precursor cell expressed, developmentally down-regulated gene 9 |
| Neu1 | S.c6 | neuraminidase 1 |
| Neu1 | S.i2vl7 | neuraminidase 1 |
| Neu1 | S.i1vl7 | neuraminidase 1 |
| Nfe2l1 | S.c2 | nuclear factor, erythroid derived 2,-like 1 |
| Nfe2l2 | S.c4 | nuclear factor, erythroid derived 2, like 2 |
| Nfe2l2 | S.i1vl7 | nuclear factor, erythroid derived 2, like 2 |
| Nfe2l3 | S.i2vl7 | nuclear factor, erythroid derived 2, like 3 |
| Nfe2l3 | S.i1vl7 | nuclear factor, erythroid derived 2, like 3 |
| Nfe2l3 | S.i3vl7 | nuclear factor, erythroid derived 2, like 3 |
| Nfe2l3 | c5.i3vl7 | nuclear factor, erythroid derived 2, like 3 |
| Nfix | S.i3vl7 | nuclear factor I/X |
| Nfix | S.i4vl7 | nuclear factor I/X |
| Nfix | S.i2vl7 | nuclear factor I/X |
| Nfix | S.c2 | nuclear factor I/X |
| Nfkb1 | S.i4vl7 | nuclear factor of kappa light polypeptide gene enhancer in B cells 1, p105 |
| Nfkb1 | S.i1vl7 | nuclear factor of kappa light polypeptide gene enhancer in B cells 1, p105 |
| Nfkb1 | S.i2vl7 | nuclear factor of kappa light polypeptide gene enhancer in B cells 1, p105 |
| Nfkb1 | S.c3 | nuclear factor of kappa light polypeptide gene enhancer in B cells 1, p105 |
| Nfkb1 | S.i3vl7 | nuclear factor of kappa light polypeptide gene enhancer in B cells 1, p105 |
| Nfkb2 | S.c1 | nuclear factor of kappa light polypeptide gene enhancer in B cells 2, p49/p100 |
| Nfkbia | S.c7 | nuclear factor of kappa light polypeptide gene enhancer in B cells inhibitor, alpha |
| Ngrn | S.i4vl7 | neugrin, neurite outgrowth associated |
| Ngrn | S.c3 | neugrin, neurite outgrowth associated |
| Nid1 | S.i4vl7 | nidogen 1 |
| Nid1 | S.c2 | nidogen 1 |
| Ninj1 | S.i4vl7 | ninjurin 1 |
| Ninj1 | S.c1 | ninjurin 1 |
| Nipbl | S.i2vl7 | Nipped-B homolog (Drosophila) |
| Nipbl | S.c3 | Nipped-B homolog (Drosophila) |
| Nipbl | S.i3vl7 | Nipped-B homolog (Drosophila) |
| Nisch | S.i2vl7 | nischarin |
| Nisch | S.i4vl7 | nischarin |
| Nisch | S.c3 | nischarin |
| Nisch | S.i3vl7 | nischarin |
| Nkiras1 | S.i4vl7 | NFKB inhibitor interacting Ras-like protein 1 |
| Nkiras1 | S.i3vl7 | NFKB inhibitor interacting Ras-like protein 1 |
| Nkiras1 | S.i2vl7 | NFKB inhibitor interacting Ras-like protein 1 |
| Nkiras1 | S.c8 | NFKB inhibitor interacting Ras-like protein 1 |
| Nkiras1 | S.i1vl7 | NFKB inhibitor interacting Ras-like protein 1 |
| Nmd3 | S.c3 | NMD3 homolog (S. cerevisiae) |
| Nme6 | S.c8 | NME/NM23 nucleoside diphosphate kinase 6 |
| Nme6 | S.i4vl7 | NME/NM23 nucleoside diphosphate kinase 6 |
| Nme6 | S.i3vl7 | NME/NM23 nucleoside diphosphate kinase 6 |
| Nmt1 | S.i2vl7 | N-myristoyltransferase 1 |
| Nmt1 | c5.i3vl7 | N-myristoyltransferase 1 |
| Nmt1 | S.i1vl7 | N-myristoyltransferase 1 |
| Nmt1 | S.i3vl7 | N-myristoyltransferase 1 |
| Nnmt | S.c1 | nicotinamide N-methyltransferase |
| Nono | S.i2vl7 | non-POU-domain-containing, octamer binding protein |
| Nono | S.i3vl7 | non-POU-domain-containing, octamer binding protein |
| Nono | S.c4 | non-POU-domain-containing, octamer binding protein |
| Nono | S.i4vl7 | non-POU-domain-containing, octamer binding protein |
| Nono | S.i1vl7 | non-POU-domain-containing, octamer binding protein |
| Nop16 | S.i4vl7 | NOP16 nucleolar protein |
| Nop16 | S.c3 | NOP16 nucleolar protein |
| Nop16 | S.i3vl7 | NOP16 nucleolar protein |
| Nop16 | S.i2vl7 | NOP16 nucleolar protein |
| Notch1 | S.i4vl7 | notch 1 |
| Notch1 | S.i2vl7 | notch 1 |
| Notch1 | S.i3vl7 | notch 1 |
| Notch1 | S.c3 | notch 1 |
| Notch4 | S.c8 | notch 4 |
| Npc1 | S.i4vl7 | Niemann Pick type C1 |
| Npc1 | S.i1vl7 | Niemann Pick type C1 |
| Npc2 | S.i3vl7 | Niemann Pick type C2 |
| Npepps | S.i2vl7 | aminopeptidase puromycin sensitive |
| Npepps | S.i4vl7 | aminopeptidase puromycin sensitive |
| Npl | S.i4vl7 | N-acetylneuraminate pyruvate lyase |
| Npl | S.c1 | N-acetylneuraminate pyruvate lyase |
| Npm1 | S.c4 | nucleophosmin 1 |
| Npnt | S.c7 | nephronectin |
| Nptn | S.i2vl7 | neuroplastin |
| Nptn | S.c2 | neuroplastin |
| Nptn | S.i4vl7 | neuroplastin |
| Nptn | S.i3vl7 | neuroplastin |
| Nr1h3 | S.i2vl7 | nuclear receptor subfamily 1, group H, member 3 |
| Nr1h3 | S.i4vl7 | nuclear receptor subfamily 1, group H, member 3 |
| Nr1h3 | S.c2 | nuclear receptor subfamily 1, group H, member 3 |
| Nras | S.i4vl7 | neuroblastoma ras oncogene |
| Nras | S.i1vl7 | neuroblastoma ras oncogene |
| Nras | S.i2vl7 | neuroblastoma ras oncogene |
| Nras | S.i3vl7 | neuroblastoma ras oncogene |
| Nras | S.c4 | neuroblastoma ras oncogene |
| Nrep | S.i4vl7 | neuronal regeneration related protein |
| Nrep | S.c9 | neuronal regeneration related protein |
| Nrp1 | S.c2 | neuropilin 1 |
| Nrp1 | S.i2vl7 | neuropilin 1 |
| Nrp1 | S.i4vl7 | neuropilin 1 |
| Nrp1 | S.i3vl7 | neuropilin 1 |
| Nsdhl | S.i1vl7 | NAD(P) dependent steroid dehydrogenase-like |
| Nsdhl | S.c2 | NAD(P) dependent steroid dehydrogenase-like |
| Nsdhl | S.i2vl7 | NAD(P) dependent steroid dehydrogenase-like |
| Nsdhl | S.c7 | NAD(P) dependent steroid dehydrogenase-like |
| Nsdhl | S.i3vl7 | NAD(P) dependent steroid dehydrogenase-like |
| Nsmce1 | S.i1vl7 | non-SMC element 1 homolog (S. cerevisiae) |
| Nsmce1 | S.c4 | non-SMC element 1 homolog (S. cerevisiae) |
| Nsmce4a | S.c3 | non-SMC element 4 homolog A (S. cerevisiae) |
| Nt5dc3 | S.c7 | 5'-nucleotidase domain containing 3 |
| Ntpcr | S.c4 | nucleoside-triphosphatase, cancer-related |
| Ntpcr | S.i4vl7 | nucleoside-triphosphatase, cancer-related |
| Nub1 | S.c3 | negative regulator of ubiquitin-like proteins 1 |
| Nucb1 | S.i3vl7 | nucleobindin 1 |
| Nucb1 | S.c2 | nucleobindin 1 |
| Nucb1 | S.i2vl7 | nucleobindin 1 |
| Nucb1 | S.i4vl7 | nucleobindin 1 |
| Nucb2 | S.i4vl7 | nucleobindin 2 |
| Nucb2 | S.c9 | nucleobindin 2 |
| Nucks1 | S.i4vl7 | nuclear casein kinase and cyclin-dependent kinase substrate 1 |
| Nucks1 | S.c4 | nuclear casein kinase and cyclin-dependent kinase substrate 1 |
| Nudc | S.i4vl7 | nuclear distribution gene C homolog (Aspergillus) |
| Nudc | S.c8 | nuclear distribution gene C homolog (Aspergillus) |
| Nudc | S.i1vl7 | nuclear distribution gene C homolog (Aspergillus) |
| Nudc | S.i2vl7 | nuclear distribution gene C homolog (Aspergillus) |
| Nudt9 | S.c4 | nudix (nucleoside diphosphate linked moiety X)-type motif 9 |
| Nudt9 | S.i4vl7 | nudix (nucleoside diphosphate linked moiety X)-type motif 9 |
| Nupr1 | S.i3vl7 | nuclear protein transcription regulator 1 |
| Nupr1 | S.c9 | nuclear protein transcription regulator 1 |
| Nupr1 | S.i1vl7 | nuclear protein transcription regulator 1 |
| Nupr1 | S.i4vl7 | nuclear protein transcription regulator 1 |
| Nutf2 | S.c4 | nuclear transport factor 2 |
| Nutf2-ps1 | S.c4 | nuclear transport factor 2, pseudogene 1 |
| Oat | S.i2vl7 | ornithine aminotransferase |
| Oat | S.c4 | ornithine aminotransferase |
| Odc1 | S.i2vl7 | ornithine decarboxylase, structural 1 |
| Odc1 | S.c7 | ornithine decarboxylase, structural 1 |
| Odc1 | S.i4vl7 | ornithine decarboxylase, structural 1 |
| Odc1 | S.i1vl7 | ornithine decarboxylase, structural 1 |
| Odc1 | S.i3vl7 | ornithine decarboxylase, structural 1 |
| Odf2 | S.c8 | outer dense fiber of sperm tails 2 |
| Odf2 | S.i4vl7 | outer dense fiber of sperm tails 2 |
| Ogdh | S.i4vl7 | oxoglutarate (alpha-ketoglutarate) dehydrogenase (lipoamide) |
| Ogdh | S.i2vl7 | oxoglutarate (alpha-ketoglutarate) dehydrogenase (lipoamide) |
| Ogdh | S.c3 | oxoglutarate (alpha-ketoglutarate) dehydrogenase (lipoamide) |
| Ogdh | S.i3vl7 | oxoglutarate (alpha-ketoglutarate) dehydrogenase (lipoamide) |
| Ogn | S.i4vl7 | osteoglycin |
| Ogn | S.c1 | osteoglycin |
| Ogn | S.c2 | osteoglycin |
| Orc5 | S.c6 | origin recognition complex, subunit 5 |
| Orc5 | S.c7 | origin recognition complex, subunit 5 |
| Orc5 | S.i2vl7 | origin recognition complex, subunit 5 |
| Orc5 | S.i1vl7 | origin recognition complex, subunit 5 |
| Orc5 | S.i4vl7 | origin recognition complex, subunit 5 |
| Orc5 | S.i3vl7 | origin recognition complex, subunit 5 |
| Orc6 | S.i1vl7 | origin recognition complex, subunit 6 |
| Orm1 | S.i3vl7 | orosomucoid 1 |
| Orm1 | S.c9 | orosomucoid 1 |
| Orm1 | S.i4vl7 | orosomucoid 1 |
| Orm2 | S.c6 | orosomucoid 2 |
| Orm2 | S.i1vl7 | orosomucoid 2 |
| Orm2 | S.i4vl7 | orosomucoid 2 |
| Orm2 | S.c9 | orosomucoid 2 |
| Ormdl1 | S.c6 | ORM1-like 1 (S. cerevisiae) |
| Ormdl2 | S.c7 | ORM1-like 2 (S. cerevisiae) |
| Ormdl2 | S.i4vl7 | ORM1-like 2 (S. cerevisiae) |
| Ormdl2 | S.i3vl7 | ORM1-like 2 (S. cerevisiae) |
| Os9 | S.i4vl7 | amplified in osteosarcoma |
| Osbpl11 | S.c1 | oxysterol binding protein-like 11 |
| Osbpl1a | S.c7 | oxysterol binding protein-like 1A |
| Osbpl1a | S.i1vl7 | oxysterol binding protein-like 1A |
| Osbpl1a | S.i3vl7 | oxysterol binding protein-like 1A |
| Osbpl1a | S.i4vl7 | oxysterol binding protein-like 1A |
| Osbpl1a | S.i2vl7 | oxysterol binding protein-like 1A |
| Osmr | S.i4vl7 | oncostatin M receptor |
| Osmr | S.i2vl7 | oncostatin M receptor |
| Osmr | S.i3vl7 | oncostatin M receptor |
| Osmr | c5.i3vl7 | oncostatin M receptor |
| Ostf1 | S.c4 | osteoclast stimulating factor 1 |
| Ostf1 | S.i1vl7 | osteoclast stimulating factor 1 |
| Ostf1 | S.i4vl7 | osteoclast stimulating factor 1 |
| Ostf1 | S.i3vl7 | osteoclast stimulating factor 1 |
| Otud5 | S.i2vl7 | OTU domain containing 5 |
| Otud5 | S.i1vl7 | OTU domain containing 5 |
| Otud5 | S.c4 | OTU domain containing 5 |
| Ovgp1 | S.i1vl7 | oviductal glycoprotein 1 |
| Ovgp1 | S.c8 | oviductal glycoprotein 1 |
| Ovgp1 | S.i2vl7 | oviductal glycoprotein 1 |
| Ovgp1 | S.i4vl7 | oviductal glycoprotein 1 |
| Oxct1 | S.i2vl7 | 3-oxoacid CoA transferase 1 |
| Oxct1 | S.i1vl7 | 3-oxoacid CoA transferase 1 |
| Oxct1 | S.c7 | 3-oxoacid CoA transferase 1 |
| Oxct1 | S.i3vl7 | 3-oxoacid CoA transferase 1 |
| Oxct1 | S.i4vl7 | 3-oxoacid CoA transferase 1 |
| P2rx4 | S.i1vl7 | purinergic receptor P2X, ligand-gated ion channel 4 |
| P2rx4 | S.c4 | purinergic receptor P2X, ligand-gated ion channel 4 |
| P2rx7 | S.i4vl7 | purinergic receptor P2X, ligand-gated ion channel, 7 |
| P2rx7 | S.c8 | purinergic receptor P2X, ligand-gated ion channel, 7 |
| Pabpc4 | S.c6 | poly(A) binding protein, cytoplasmic 4 |
| Pabpc4 | S.i1vl7 | poly(A) binding protein, cytoplasmic 4 |
| Paip2 | S.i1vl7 | polyadenylate-binding protein-interacting protein 2 |
| Paip2 | S.c6 | polyadenylate-binding protein-interacting protein 2 |
| Pak2 | S.c6 | p21 protein (Cdc42/Rac)-activated kinase 2 |
| Pak4 | S.c8 | p21 protein (Cdc42/Rac)-activated kinase 4 |
| Pald1 | S.c4 | phosphatase domain containing, paladin 1 |
| Palm | S.c1 | paralemmin |
| Papola | S.c8 | poly (A) polymerase alpha |
| Parl | S.c4 | presenilin associated, rhomboid-like |
| Parm1 | S.c2 | prostate androgen-regulated mucin-like protein 1 |
| Parp2 | S.c4 | poly (ADP-ribose) polymerase family, member 2 |
| Pax4 | S.i2vl7 | paired box 4 |
| Pax4 | S.i4vl7 | paired box 4 |
| Pax4 | S.i3vl7 | paired box 4 |
| Pax4 | S.c8 | paired box 4 |
| Paxbp1 | S.i3vl7 | PAX3 and PAX7 binding protein 1 |
| Paxbp1 | S.i1vl7 | PAX3 and PAX7 binding protein 1 |
| Paxbp1 | c5.i3vl7 | PAX3 and PAX7 binding protein 1 |
| Paxbp1 | S.i2vl7 | PAX3 and PAX7 binding protein 1 |
| Paxbp1 | S.i4vl7 | PAX3 and PAX7 binding protein 1 |
| Pbx3 | S.i4vl7 | pre B cell leukemia homeobox 3 |
| Pcbd2 | S.c6 | pterin 4 alpha carbinolamine dehydratase/dimerization cofactor of hepatocyte nuclear factor 1 alpha (TCF1) 2 |
| Pcbp2 | S.i1vl7 | poly(rC) binding protein 2 |
| Pcbp2 | c5.i3vl7 | poly(rC) binding protein 2 |
| Pcbp2 | S.i2vl7 | poly(rC) binding protein 2 |
| Pcbp2 | S.i3vl7 | poly(rC) binding protein 2 |
| Pcdha1 | S.c8 | protocadherin alpha 1 |
| Pcdha10 | S.c8 | protocadherin alpha 10 |
| Pcdha11 | S.c8 | protocadherin alpha 11 |
| Pcdha12 | S.c8 | protocadherin alpha 12 |
| Pcdha2 | S.c8 | protocadherin alpha 2 |
| Pcdha4 | S.c8 | protocadherin alpha 4 |
| Pcdha9 | S.c8 | protocadherin alpha 9 |
| Pcdhac1 | S.c8 | protocadherin alpha subfamily C, 1 |
| Pcdhac2 | S.c8 | protocadherin alpha subfamily C, 2 |
| Pcdhga11 | S.i3vl7 | protocadherin gamma subfamily A, 11 |
| Pcdhga11 | S.c2 | protocadherin gamma subfamily A, 11 |
| Pcdhga11 | S.i4vl7 | protocadherin gamma subfamily A, 11 |
| Pcdhga12 | S.i4vl7 | protocadherin gamma subfamily A, 12 |
| Pcdhga12 | S.c2 | protocadherin gamma subfamily A, 12 |
| Pcdhga12 | S.i3vl7 | protocadherin gamma subfamily A, 12 |
| Pcdhga3 | S.c2 | protocadherin gamma subfamily A, 3 |
| Pcdhga3 | S.i3vl7 | protocadherin gamma subfamily A, 3 |
| Pcdhga3 | S.i4vl7 | protocadherin gamma subfamily A, 3 |
| Pcdhga8 | S.i4vl7 | protocadherin gamma subfamily A, 8 |
| Pcdhga8 | S.c2 | protocadherin gamma subfamily A, 8 |
| Pcdhga8 | S.i3vl7 | protocadherin gamma subfamily A, 8 |
| Pcdhga9 | S.i4vl7 | protocadherin gamma subfamily A, 9 |
| Pcdhga9 | S.c2 | protocadherin gamma subfamily A, 9 |
| Pcdhga9 | S.i3vl7 | protocadherin gamma subfamily A, 9 |
| Pcdhgb6 | S.c2 | protocadherin gamma subfamily B, 6 |
| Pcdhgb6 | S.i4vl7 | protocadherin gamma subfamily B, 6 |
| Pcdhgb6 | S.i3vl7 | protocadherin gamma subfamily B, 6 |
| Pcdhgc3 | S.i3vl7 | protocadherin gamma subfamily C, 3 |
| Pcdhgc3 | S.i4vl7 | protocadherin gamma subfamily C, 3 |
| Pcdhgc3 | S.c2 | protocadherin gamma subfamily C, 3 |
| Pcdhgc4 | S.i3vl7 | protocadherin gamma subfamily C, 4 |
| Pcdhgc4 | S.i4vl7 | protocadherin gamma subfamily C, 4 |
| Pcdhgc4 | S.c2 | protocadherin gamma subfamily C, 4 |
| Pcdhgc5 | S.i3vl7 | protocadherin gamma subfamily C, 5 |
| Pcdhgc5 | S.c2 | protocadherin gamma subfamily C, 5 |
| Pcdhgc5 | S.i4vl7 | protocadherin gamma subfamily C, 5 |
| Pck1 | S.c2 | phosphoenolpyruvate carboxykinase 1, cytosolic |
| Pcmt1 | S.i2vl7 | protein-L-isoaspartate (D-aspartate) O-methyltransferase 1 |
| Pcmt1 | S.i4vl7 | protein-L-isoaspartate (D-aspartate) O-methyltransferase 1 |
| Pcnt | S.c7 | pericentrin (kendrin) |
| Pcolce | S.i4vl7 | procollagen C-endopeptidase enhancer protein |
| Pcolce | S.i2vl7 | procollagen C-endopeptidase enhancer protein |
| Pcolce | S.c2 | procollagen C-endopeptidase enhancer protein |
| Pcolce | S.i1vl7 | procollagen C-endopeptidase enhancer protein |
| Pcolce | S.i3vl7 | procollagen C-endopeptidase enhancer protein |
| Pcx | S.i1vl7 | pyruvate carboxylase |
| Pcx | S.i2vl7 | pyruvate carboxylase |
| Pcx | S.i3vl7 | pyruvate carboxylase |
| Pcx | S.c7 | pyruvate carboxylase |
| Pcyt2 | S.i1vl7 | phosphate cytidylyltransferase 2, ethanolamine |
| Pcyt2 | S.i3vl7 | phosphate cytidylyltransferase 2, ethanolamine |
| Pcyt2 | S.c7 | phosphate cytidylyltransferase 2, ethanolamine |
| Pcyt2 | S.i2vl7 | phosphate cytidylyltransferase 2, ethanolamine |
| Pdc | S.i3vl7 | phosducin |
| Pdc | S.i2vl7 | phosducin |
| Pdc | S.c8 | phosducin |
| Pdc | S.i4vl7 | phosducin |
| Pdcd2l | S.c9 | programmed cell death 2-like |
| Pdcd4 | S.i2vl7 | programmed cell death 4 |
| Pdcd4 | S.i1vl7 | programmed cell death 4 |
| Pdcd4 | S.i4vl7 | programmed cell death 4 |
| Pdcd4 | S.c7 | programmed cell death 4 |
| Pdcd4 | S.i3vl7 | programmed cell death 4 |
| Pdcd5 | S.i1vl7 | programmed cell death 5 |
| Pdcd5 | S.i2vl7 | programmed cell death 5 |
| Pdcd5 | S.c4 | programmed cell death 5 |
| Pdgfra | S.i4vl7 | platelet derived growth factor receptor, alpha polypeptide |
| Pdgfra | S.c3 | platelet derived growth factor receptor, alpha polypeptide |
| Pdha1 | S.c8 | pyruvate dehydrogenase E1 alpha 1 |
| Pdha1 | S.i3vl7 | pyruvate dehydrogenase E1 alpha 1 |
| Pdhb | S.i3vl7 | pyruvate dehydrogenase (lipoamide) beta |
| Pdhb | S.i4vl7 | pyruvate dehydrogenase (lipoamide) beta |
| Pdhb | S.c7 | pyruvate dehydrogenase (lipoamide) beta |
| Pdhb | S.i1vl7 | pyruvate dehydrogenase (lipoamide) beta |
| Pdhb | S.i2vl7 | pyruvate dehydrogenase (lipoamide) beta |
| Pdia3 | S.i2vl7 | protein disulfide isomerase associated 3 |
| Pdia4 | S.i3vl7 | protein disulfide isomerase associated 4 |
| Pdia4 | S.c2 | protein disulfide isomerase associated 4 |
| Pdia6 | S.i3vl7 | protein disulfide isomerase associated 6 |
| Pdia6 | S.c2 | protein disulfide isomerase associated 6 |
| Pdia6 | S.i4vl7 | protein disulfide isomerase associated 6 |
| Pdk1 | S.i4vl7 | pyruvate dehydrogenase kinase, isoenzyme 1 |
| Pdk1 | S.c9 | pyruvate dehydrogenase kinase, isoenzyme 1 |
| Pdk3 | S.c1 | pyruvate dehydrogenase kinase, isoenzyme 3 |
| Pdk4 | S.i3vl7 | pyruvate dehydrogenase kinase, isoenzyme 4 |
| Pdk4 | S.i4vl7 | pyruvate dehydrogenase kinase, isoenzyme 4 |
| Pdk4 | S.i1vl7 | pyruvate dehydrogenase kinase, isoenzyme 4 |
| Pdk4 | c5.i3vl7 | pyruvate dehydrogenase kinase, isoenzyme 4 |
| Pdk4 | S.i2vl7 | pyruvate dehydrogenase kinase, isoenzyme 4 |
| Pdlim1 | S.c6 | PDZ and LIM domain 1 (elfin) |
| Pdlim1 | S.i3vl7 | PDZ and LIM domain 1 (elfin) |
| Pdlim1 | S.i2vl7 | PDZ and LIM domain 1 (elfin) |
| Pdlim1 | S.i4vl7 | PDZ and LIM domain 1 (elfin) |
| Pdlim1 | S.i1vl7 | PDZ and LIM domain 1 (elfin) |
| Pdlim3 | S.i4vl7 | PDZ and LIM domain 3 |
| Pdlim3 | S.c1 | PDZ and LIM domain 3 |
| Pdlim7 | S.i3vl7 | PDZ and LIM domain 7 |
| Pdlim7 | S.c2 | PDZ and LIM domain 7 |
| Pdpk1 | S.i1vl7 | 3-phosphoinositide dependent protein kinase 1 |
| Pdpn | S.c1 | podoplanin |
| Pdpn | S.i4vl7 | podoplanin |
| Pdrg1 | S.i2vl7 | p53 and DNA damage regulated 1 |
| Pdrg1 | S.c3 | p53 and DNA damage regulated 1 |
| Pdrg1 | S.i3vl7 | p53 and DNA damage regulated 1 |
| Pdxk | S.c8 | pyridoxal (pyridoxine, vitamin B6) kinase |
| Pdzd11 | S.c6 | PDZ domain containing 11 |
| Pdzd11 | S.i1vl7 | PDZ domain containing 11 |
| Pdzk1ip1 | S.i4vl7 | PDZK1 interacting protein 1 |
| Pdzk1ip1 | S.c9 | PDZK1 interacting protein 1 |
| Pecam1 | S.i4vl7 | platelet/endothelial cell adhesion molecule 1 |
| Pecam1 | S.c8 | platelet/endothelial cell adhesion molecule 1 |
| Pecam1 | S.i3vl7 | platelet/endothelial cell adhesion molecule 1 |
| Penk | S.i2vl7 | preproenkephalin |
| Penk | S.c8 | preproenkephalin |
| Penk | S.c1 | preproenkephalin |
| Penk | S.i4vl7 | preproenkephalin |
| Pepd | S.i4vl7 | peptidase D |
| Pepd | S.c3 | peptidase D |
| Pex6 | S.i3vl7 | peroxisomal biogenesis factor 6 |
| Pex6 | S.i2vl7 | peroxisomal biogenesis factor 6 |
| Pex6 | S.c2 | peroxisomal biogenesis factor 6 |
| Pfdn2 | S.c3 | prefoldin 2 |
| Pfkl | S.i1vl7 | phosphofructokinase, liver, B-type |
| Pfkl | S.c7 | phosphofructokinase, liver, B-type |
| Pfkp | S.c1 | phosphofructokinase, platelet |
| Pfkp | S.i4vl7 | phosphofructokinase, platelet |
| Pfkp | S.i3vl7 | phosphofructokinase, platelet |
| Pfn2 | S.i4vl7 | profilin 2 |
| Pfn2 | S.i3vl7 | profilin 2 |
| Pgam1 | S.c7 | phosphoglycerate mutase 1 |
| Pgam1 | S.i3vl7 | phosphoglycerate mutase 1 |
| Pgam1 | S.i2vl7 | phosphoglycerate mutase 1 |
| Pgm2 | S.i4vl7 | phosphoglucomutase 2 |
| Pgm2 | S.c1 | phosphoglucomutase 2 |
| Pgp | S.c7 | phosphoglycolate phosphatase |
| Pgrmc1 | S.i1vl7 | progesterone receptor membrane component 1 |
| Pgrmc1 | S.c3 | progesterone receptor membrane component 1 |
| Pgrmc1 | S.i4vl7 | progesterone receptor membrane component 1 |
| Pgrmc1 | S.i2vl7 | progesterone receptor membrane component 1 |
| Pgrmc1 | S.i3vl7 | progesterone receptor membrane component 1 |
| Phb | S.i1vl7 | prohibitin |
| Phb | S.c7 | prohibitin |
| Phlda1 | S.i3vl7 | pleckstrin homology-like domain, family A, member 1 |
| Phlda1 | S.c4 | pleckstrin homology-like domain, family A, member 1 |
| Phlda1 | S.i4vl7 | pleckstrin homology-like domain, family A, member 1 |
| Phlda3 | S.c3 | pleckstrin homology-like domain, family A, member 3 |
| Phlda3 | S.i4vl7 | pleckstrin homology-like domain, family A, member 3 |
| Phlda3 | S.i3vl7 | pleckstrin homology-like domain, family A, member 3 |
| Phtf2 | S.c8 | putative homeodomain transcription factor 2 |
| Phyh | S.c3 | phytanoyl-CoA hydroxylase |
| Phyh | S.i3vl7 | phytanoyl-CoA hydroxylase |
| Phyh | S.i4vl7 | phytanoyl-CoA hydroxylase |
| Pigk | S.i2vl7 | phosphatidylinositol glycan anchor biosynthesis, class K |
| Pigk | S.i4vl7 | phosphatidylinositol glycan anchor biosynthesis, class K |
| Pigk | S.c8 | phosphatidylinositol glycan anchor biosynthesis, class K |
| Pigr | S.i4vl7 | polymeric immunoglobulin receptor |
| Pigr | S.c9 | polymeric immunoglobulin receptor |
| Pigs | S.i4vl7 | phosphatidylinositol glycan anchor biosynthesis, class S |
| Pigx | S.i3vl7 | phosphatidylinositol glycan anchor biosynthesis, class X |
| Pigx | S.i2vl7 | phosphatidylinositol glycan anchor biosynthesis, class X |
| Pigx | c5.i3vl7 | phosphatidylinositol glycan anchor biosynthesis, class X |
| Pigx | S.i1vl7 | phosphatidylinositol glycan anchor biosynthesis, class X |
| Pik3r1 | S.i4vl7 | phosphatidylinositol 3-kinase, regulatory subunit, polypeptide 1 (p85 alpha) |
| Pik3r1 | S.i2vl7 | phosphatidylinositol 3-kinase, regulatory subunit, polypeptide 1 (p85 alpha) |
| Pik3r1 | S.i1vl7 | phosphatidylinositol 3-kinase, regulatory subunit, polypeptide 1 (p85 alpha) |
| Pik3r1 | c5.i3vl7 | phosphatidylinositol 3-kinase, regulatory subunit, polypeptide 1 (p85 alpha) |
| Pik3r1 | S.i3vl7 | phosphatidylinositol 3-kinase, regulatory subunit, polypeptide 1 (p85 alpha) |
| Pim1 | S.c7 | proviral integration site 1 |
| Pim3 | S.c2 | proviral integration site 3 |
| Pip | S.c8 | prolactin induced protein |
| Pip | S.i4vl7 | prolactin induced protein |
| Pip | S.i2vl7 | prolactin induced protein |
| Pip | S.i3vl7 | prolactin induced protein |
| Pip4k2c | S.c6 | phosphatidylinositol-5-phosphate 4-kinase, type II, gamma |
| Pip4k2c | S.i1vl7 | phosphatidylinositol-5-phosphate 4-kinase, type II, gamma |
| Pip5k1b | S.c7 | phosphatidylinositol-4-phosphate 5-kinase, type 1 beta |
| Pira1 | S.c2 | paired-Ig-like receptor A1 |
| Pira1 | S.i3vl7 | paired-Ig-like receptor A1 |
| Pira11 | S.i3vl7 | paired-Ig-like receptor A11 |
| Pira11 | S.c2 | paired-Ig-like receptor A11 |
| Pira2 | S.i3vl7 | paired-Ig-like receptor A2 |
| Pira2 | S.c2 | paired-Ig-like receptor A2 |
| Pira4 | S.i3vl7 | paired-Ig-like receptor A4 |
| Pira4 | S.c2 | paired-Ig-like receptor A4 |
| Pira6 | S.c2 | paired-Ig-like receptor A6 |
| Pira6 | S.i3vl7 | paired-Ig-like receptor A6 |
| Pira7 | S.i3vl7 | paired-Ig-like receptor A7 |
| Pira7 | S.c2 | paired-Ig-like receptor A7 |
| Pirb | S.c2 | paired Ig-like receptor B |
| Pirb | S.i3vl7 | paired Ig-like receptor B |
| Pkd2 | S.i3vl7 | polycystic kidney disease 2 |
| Pkd2 | S.i2vl7 | polycystic kidney disease 2 |
| Pkd2 | S.i4vl7 | polycystic kidney disease 2 |
| Pkd2 | S.c3 | polycystic kidney disease 2 |
| Pkig | S.c3 | protein kinase inhibitor, gamma |
| Pkp4 | S.i4vl7 | plakophilin 4 |
| Pkp4 | S.c3 | plakophilin 4 |
| Pla2g12a | S.i4vl7 | phospholipase A2, group XIIA |
| Pla2g12a | S.c9 | phospholipase A2, group XIIA |
| Pla2g4f | S.i4vl7 | phospholipase A2, group IVF |
| Pla2g4f | S.c8 | phospholipase A2, group IVF |
| Pla2g6 | S.i1vl7 | phospholipase A2, group VI |
| Pla2g6 | S.c7 | phospholipase A2, group VI |
| Pla2g7 | S.c1 | phospholipase A2, group VII (platelet-activating factor acetylhydrolase, plasma) |
| Pla2g7 | S.i4vl7 | phospholipase A2, group VII (platelet-activating factor acetylhydrolase, plasma) |
| Plac1 | S.i4vl7 | placental specific protein 1 |
| Plac1 | S.c8 | placental specific protein 1 |
| Plac8 | S.c2 | placenta-specific 8 |
| Plac8 | S.i4vl7 | placenta-specific 8 |
| Plac9a | S.c2 | placenta specific 9a |
| Plac9b | S.c2 | placenta specific 9b |
| Plbd1 | S.i3vl7 | phospholipase B domain containing 1 |
| Plbd1 | S.c1 | phospholipase B domain containing 1 |
| Plbd1 | S.i4vl7 | phospholipase B domain containing 1 |
| Plcg2 | S.i3vl7 | phospholipase C, gamma 2 |
| Pld3 | S.i4vl7 | phospholipase D family, member 3 |
| Pld4 | S.i4vl7 | phospholipase D family, member 4 |
| Pld4 | S.c1 | phospholipase D family, member 4 |
| Plekhb2 | S.c1 | pleckstrin homology domain containing, family B (evectins) member 2 |
| Plin4 | S.i2vl7 | perilipin 4 |
| Plin4 | S.i3vl7 | perilipin 4 |
| Plin4 | S.c3 | perilipin 4 |
| Plin4 | S.i4vl7 | perilipin 4 |
| Plk2 | S.c1 | polo-like kinase 2 |
| Plk3 | S.c8 | polo-like kinase 3 |
| Plk3 | S.i4vl7 | polo-like kinase 3 |
| Plk3 | S.i3vl7 | polo-like kinase 3 |
| Plk3 | S.i2vl7 | polo-like kinase 3 |
| Plp1 | S.c8 | proteolipid protein (myelin) 1 |
| Plp2 | S.i4vl7 | proteolipid protein 2 |
| Plp2 | S.i2vl7 | proteolipid protein 2 |
| Plp2 | S.i1vl7 | proteolipid protein 2 |
| Plp2 | S.i3vl7 | proteolipid protein 2 |
| Plp2 | S.c4 | proteolipid protein 2 |
| Pls3 | S.i2vl7 | plastin 3 (T-isoform) |
| Pls3 | S.c4 | plastin 3 (T-isoform) |
| Pls3 | S.i1vl7 | plastin 3 (T-isoform) |
| Pls3 | S.i3vl7 | plastin 3 (T-isoform) |
| Pls3 | S.i4vl7 | plastin 3 (T-isoform) |
| Plscr2 | S.c4 | phospholipid scramblase 2 |
| Plscr2 | S.i2vl7 | phospholipid scramblase 2 |
| Plscr2 | S.i1vl7 | phospholipid scramblase 2 |
| Pltp | S.c2 | phospholipid transfer protein |
| Pltp | S.i4vl7 | phospholipid transfer protein |
| Pltp | S.i2vl7 | phospholipid transfer protein |
| Pltp | S.i3vl7 | phospholipid transfer protein |
| Plxnb2 | c5.i3vl7 | plexin B2 |
| Plxnb2 | S.i2vl7 | plexin B2 |
| Plxnb2 | S.i3vl7 | plexin B2 |
| Plxnb2 | S.i4vl7 | plexin B2 |
| Plxnd1 | S.i4vl7 | plexin D1 |
| Plxnd1 | S.c1 | plexin D1 |
| Pmm2 | S.c7 | phosphomannomutase 2 |
| Pmp22 | S.c3 | peripheral myelin protein 22 |
| Pmp22 | S.i4vl7 | peripheral myelin protein 22 |
| Pnp | S.i2vl7 | purine-nucleoside phosphorylase |
| Pnp | S.i4vl7 | purine-nucleoside phosphorylase |
| Pnp | S.c3 | purine-nucleoside phosphorylase |
| Pnrc2 | S.i4vl7 | proline-rich nuclear receptor coactivator 2 |
| Polb | S.c7 | polymerase (DNA directed), beta |
| Polb | S.i1vl7 | polymerase (DNA directed), beta |
| Polb | S.i4vl7 | polymerase (DNA directed), beta |
| Pold2 | S.i2vl7 | polymerase (DNA directed), delta 2, regulatory subunit |
| Pold2 | S.i1vl7 | polymerase (DNA directed), delta 2, regulatory subunit |
| Pold2 | S.i4vl7 | polymerase (DNA directed), delta 2, regulatory subunit |
| Pold2 | S.c7 | polymerase (DNA directed), delta 2, regulatory subunit |
| Pold3 | S.c7 | polymerase (DNA-directed), delta 3, accessory subunit |
| Poln | S.c1 | DNA polymerase N |
| Polr1c | S.c6 | polymerase (RNA) I polypeptide C |
| Polr2e | S.c6 | polymerase (RNA) II (DNA directed) polypeptide E |
| Pon2 | S.c3 | paraoxonase 2 |
| Pon2 | S.i4vl7 | paraoxonase 2 |
| Pon2 | S.i1vl7 | paraoxonase 2 |
| Pon2 | S.i3vl7 | paraoxonase 2 |
| Pon2 | S.i2vl7 | paraoxonase 2 |
| Pon3 | S.c1 | paraoxonase 3 |
| Por | S.i3vl7 | P450 (cytochrome) oxidoreductase |
| Por | S.i4vl7 | P450 (cytochrome) oxidoreductase |
| Por | S.i2vl7 | P450 (cytochrome) oxidoreductase |
| Por | S.c2 | P450 (cytochrome) oxidoreductase |
| Postn | S.i4vl7 | periostin, osteoblast specific factor |
| Postn | S.c3 | periostin, osteoblast specific factor |
| Postn | S.i2vl7 | periostin, osteoblast specific factor |
| Ppa1 | S.c1 | pyrophosphatase (inorganic) 1 |
| Ppa2 | S.i2vl7 | pyrophosphatase (inorganic) 2 |
| Ppa2 | S.c4 | pyrophosphatase (inorganic) 2 |
| Ppa2 | S.i1vl7 | pyrophosphatase (inorganic) 2 |
| Ppap2a | S.c1 | phosphatidic acid phosphatase type 2A |
| Ppap2b | S.c3 | phosphatidic acid phosphatase type 2B |
| Ppap2b | S.i4vl7 | phosphatidic acid phosphatase type 2B |
| Pparg | S.i4vl7 | peroxisome proliferator activated receptor gamma |
| Pparg | S.c1 | peroxisome proliferator activated receptor gamma |
| Ppic | S.i3vl7 | peptidylprolyl isomerase C |
| Ppic | S.c2 | peptidylprolyl isomerase C |
| Ppic | S.i2vl7 | peptidylprolyl isomerase C |
| Ppic | S.i4vl7 | peptidylprolyl isomerase C |
| Ppid | S.c3 | peptidylprolyl isomerase D (cyclophilin D) |
| Ppox | S.c8 | protoporphyrinogen oxidase |
| Ppox | S.i2vl7 | protoporphyrinogen oxidase |
| Ppox | S.i4vl7 | protoporphyrinogen oxidase |
| Ppp1r14b | S.c4 | protein phosphatase 1, regulatory (inhibitor) subunit 14B |
| Ppp1r15b | S.i4vl7 | protein phosphatase 1, regulatory (inhibitor) subunit 15b |
| Ppp1r15b | S.c7 | protein phosphatase 1, regulatory (inhibitor) subunit 15b |
| Ppp1r18 | S.c1 | protein phosphatase 1, regulatory subunit 18 |
| Ppp1r2 | S.c2 | protein phosphatase 1, regulatory (inhibitor) subunit 2 |
| Ppp1r2 | S.c3 | protein phosphatase 1, regulatory (inhibitor) subunit 2 |
| Ppp1r2 | S.i4vl7 | protein phosphatase 1, regulatory (inhibitor) subunit 2 |
| Ppp1r21 | S.i3vl7 | protein phosphatase 1, regulatory subunit 21 |
| Ppp1r21 | S.i4vl7 | protein phosphatase 1, regulatory subunit 21 |
| Ppp1r21 | S.c3 | protein phosphatase 1, regulatory subunit 21 |
| Ppp2cb | S.i3vl7 | protein phosphatase 2 (formerly 2A), catalytic subunit, beta isoform |
| Ppp2cb | S.c4 | protein phosphatase 2 (formerly 2A), catalytic subunit, beta isoform |
| Ppp2cb | S.i1vl7 | protein phosphatase 2 (formerly 2A), catalytic subunit, beta isoform |
| Ppp2r1a | S.i2vl7 | protein phosphatase 2, regulatory subunit A, alpha |
| Ppp2r1a | S.i1vl7 | protein phosphatase 2, regulatory subunit A, alpha |
| Ppp2r1a | S.i3vl7 | protein phosphatase 2, regulatory subunit A, alpha |
| Ppp2r1a | c5.i3vl7 | protein phosphatase 2, regulatory subunit A, alpha |
| Ppp2r5a | S.c3 | protein phosphatase 2, regulatory subunit B', alpha |
| Ppp2r5a | S.i4vl7 | protein phosphatase 2, regulatory subunit B', alpha |
| Ppp2r5c | S.i1vl7 | protein phosphatase 2, regulatory subunit B', gamma |
| Ppp4c | S.i2vl7 | protein phosphatase 4, catalytic subunit |
| Pqlc3 | S.i3vl7 | PQ loop repeat containing |
| Pqlc3 | S.i1vl7 | PQ loop repeat containing |
| Pqlc3 | S.i2vl7 | PQ loop repeat containing |
| Pqlc3 | S.i4vl7 | PQ loop repeat containing |
| Pqlc3 | S.c3 | PQ loop repeat containing |
| Pradc1 | S.i1vl7 | protease-associated domain containing 1 |
| Pradc1 | S.i3vl7 | protease-associated domain containing 1 |
| Pradc1 | S.i4vl7 | protease-associated domain containing 1 |
| Pradc1 | S.c7 | protease-associated domain containing 1 |
| Prdx1 | S.i1vl7 | peroxiredoxin 1 |
| Prdx4 | S.i4vl7 | peroxiredoxin 4 |
| Prdx4 | S.i1vl7 | peroxiredoxin 4 |
| Prdx4 | S.i2vl7 | peroxiredoxin 4 |
| Prdx4 | S.c8 | peroxiredoxin 4 |
| Prdx6 | S.i1vl7 | peroxiredoxin 6 |
| Prdx6 | S.i2vl7 | peroxiredoxin 6 |
| Prg2 | S.i3vl7 | proteoglycan 2, bone marrow |
| Prg2 | S.i2vl7 | proteoglycan 2, bone marrow |
| Prg2 | S.i4vl7 | proteoglycan 2, bone marrow |
| Prg2 | S.c8 | proteoglycan 2, bone marrow |
| Prkacb | S.i3vl7 | protein kinase, cAMP dependent, catalytic, beta |
| Prkacb | S.i2vl7 | protein kinase, cAMP dependent, catalytic, beta |
| Prkacb | S.c3 | protein kinase, cAMP dependent, catalytic, beta |
| Prkacb | S.i1vl7 | protein kinase, cAMP dependent, catalytic, beta |
| Prkacb | S.i4vl7 | protein kinase, cAMP dependent, catalytic, beta |
| Prkar1a | S.i4vl7 | protein kinase, cAMP dependent regulatory, type I, alpha |
| Prkar1a | S.i3vl7 | protein kinase, cAMP dependent regulatory, type I, alpha |
| Prkar1a | S.c4 | protein kinase, cAMP dependent regulatory, type I, alpha |
| Prkar1a | S.i2vl7 | protein kinase, cAMP dependent regulatory, type I, alpha |
| Prkcb | S.c1 | protein kinase C, beta |
| Prkcdbp | S.i2vl7 | protein kinase C, delta binding protein |
| Prkcdbp | S.c3 | protein kinase C, delta binding protein |
| Prkcdbp | S.i4vl7 | protein kinase C, delta binding protein |
| Prkcsh | S.i2vl7 | protein kinase C substrate 80K-H |
| Prkcsh | S.i3vl7 | protein kinase C substrate 80K-H |
| Prkcsh | c5.i3vl7 | protein kinase C substrate 80K-H |
| Prkcz | S.i2vl7 | protein kinase C, zeta |
| Prkcz | S.c6 | protein kinase C, zeta |
| Prkd3 | S.c1 | protein kinase D3 |
| Prlr | S.c7 | prolactin receptor |
| Prmt1 | S.c3 | protein arginine N-methyltransferase 1 |
| Prmt1 | S.i4vl7 | protein arginine N-methyltransferase 1 |
| Prmt1 | S.i2vl7 | protein arginine N-methyltransferase 1 |
| Prmt1 | S.i3vl7 | protein arginine N-methyltransferase 1 |
| Prnp | S.i2vl7 | prion protein |
| Prnp | S.i1vl7 | prion protein |
| Prnp | S.i3vl7 | prion protein |
| Prnp | S.c3 | prion protein |
| Prnp | S.i4vl7 | prion protein |
| Procr | S.i4vl7 | protein C receptor, endothelial |
| Procr | S.c1 | protein C receptor, endothelial |
| Pros1 | S.i4vl7 | protein S (alpha) |
| Pros1 | S.i3vl7 | protein S (alpha) |
| Pros1 | S.c3 | protein S (alpha) |
| Prps1 | S.c4 | phosphoribosyl pyrophosphate synthetase 1 |
| Prss23 | S.c1 | protease, serine 23 |
| Prss23 | S.i4vl7 | protease, serine 23 |
| Prss8 | S.c9 | protease, serine 8 (prostasin) |
| Prss8 | S.i4vl7 | protease, serine 8 (prostasin) |
| Prune | S.c4 | prune homolog (Drosophila) |
| Prune | S.i4vl7 | prune homolog (Drosophila) |
| Prune | S.i3vl7 | prune homolog (Drosophila) |
| Prune | S.i1vl7 | prune homolog (Drosophila) |
| Psap | S.i4vl7 | prosaposin |
| Psap | S.c2 | prosaposin |
| Psat1 | S.i2vl7 | phosphoserine aminotransferase 1 |
| Psat1 | S.i4vl7 | phosphoserine aminotransferase 1 |
| Psat1 | S.c7 | phosphoserine aminotransferase 1 |
| Psen1 | S.i1vl7 | presenilin 1 |
| Psen2 | S.c3 | presenilin 2 |
| Psen2 | S.i4vl7 | presenilin 2 |
| Psma1 | S.i2vl7 | proteasome (prosome, macropain) subunit, alpha type 1 |
| Psma1 | S.i1vl7 | proteasome (prosome, macropain) subunit, alpha type 1 |
| Psma1 | S.i4vl7 | proteasome (prosome, macropain) subunit, alpha type 1 |
| Psma1 | S.i3vl7 | proteasome (prosome, macropain) subunit, alpha type 1 |
| Psma1 | S.c4 | proteasome (prosome, macropain) subunit, alpha type 1 |
| Psma5 | S.i1vl7 | proteasome (prosome, macropain) subunit, alpha type 5 |
| Psma5 | S.i4vl7 | proteasome (prosome, macropain) subunit, alpha type 5 |
| Psma5 | S.i3vl7 | proteasome (prosome, macropain) subunit, alpha type 5 |
| Psma5 | S.c4 | proteasome (prosome, macropain) subunit, alpha type 5 |
| Psma7 | S.i2vl7 | proteasome (prosome, macropain) subunit, alpha type 7 |
| Psma7 | S.i1vl7 | proteasome (prosome, macropain) subunit, alpha type 7 |
| Psma7 | S.i3vl7 | proteasome (prosome, macropain) subunit, alpha type 7 |
| Psma7 | c5.i3vl7 | proteasome (prosome, macropain) subunit, alpha type 7 |
| Psmb10 | S.i4vl7 | proteasome (prosome, macropain) subunit, beta type 10 |
| Psmb10 | S.c1 | proteasome (prosome, macropain) subunit, beta type 10 |
| Psmb8 | S.i4vl7 | proteasome (prosome, macropain) subunit, beta type 8 (large multifunctional peptidase 7) |
| Psmb8 | S.i3vl7 | proteasome (prosome, macropain) subunit, beta type 8 (large multifunctional peptidase 7) |
| Psmb8 | S.c3 | proteasome (prosome, macropain) subunit, beta type 8 (large multifunctional peptidase 7) |
| Psmb8 | S.i2vl7 | proteasome (prosome, macropain) subunit, beta type 8 (large multifunctional peptidase 7) |
| Psmb9 | S.c1 | proteasome (prosome, macropain) subunit, beta type 9 (large multifunctional peptidase 2) |
| Psmc1 | S.i3vl7 | protease (prosome, macropain) 26S subunit, ATPase 1 |
| Psmc1 | c5.i3vl7 | protease (prosome, macropain) 26S subunit, ATPase 1 |
| Psmc1 | S.i1vl7 | protease (prosome, macropain) 26S subunit, ATPase 1 |
| Psmc2 | S.i2vl7 | proteasome (prosome, macropain) 26S subunit, ATPase 2 |
| Psmc3 | S.i4vl7 | proteasome (prosome, macropain) 26S subunit, ATPase 3 |
| Psmc3 | S.i3vl7 | proteasome (prosome, macropain) 26S subunit, ATPase 3 |
| Psmc3 | c5.i3vl7 | proteasome (prosome, macropain) 26S subunit, ATPase 3 |
| Psmc3 | S.i2vl7 | proteasome (prosome, macropain) 26S subunit, ATPase 3 |
| Psmd1 | S.i3vl7 | proteasome (prosome, macropain) 26S subunit, non-ATPase, 1 |
| Psmd1 | S.i4vl7 | proteasome (prosome, macropain) 26S subunit, non-ATPase, 1 |
| Psmd1 | S.c4 | proteasome (prosome, macropain) 26S subunit, non-ATPase, 1 |
| Psmd11 | S.i3vl7 | proteasome (prosome, macropain) 26S subunit, non-ATPase, 11 |
| Psmd11 | c5.i3vl7 | proteasome (prosome, macropain) 26S subunit, non-ATPase, 11 |
| Psmd11 | S.c4 | proteasome (prosome, macropain) 26S subunit, non-ATPase, 11 |
| Psmd11 | S.i2vl7 | proteasome (prosome, macropain) 26S subunit, non-ATPase, 11 |
| Psmd11 | S.i4vl7 | proteasome (prosome, macropain) 26S subunit, non-ATPase, 11 |
| Psmd14 | S.c4 | proteasome (prosome, macropain) 26S subunit, non-ATPase, 14 |
| Psmd4 | S.i1vl7 | proteasome (prosome, macropain) 26S subunit, non-ATPase, 4 |
| Psmd4 | S.i3vl7 | proteasome (prosome, macropain) 26S subunit, non-ATPase, 4 |
| Psmd4 | S.c4 | proteasome (prosome, macropain) 26S subunit, non-ATPase, 4 |
| Psmd6 | S.i1vl7 | proteasome (prosome, macropain) 26S subunit, non-ATPase, 6 |
| Psmd6 | S.c4 | proteasome (prosome, macropain) 26S subunit, non-ATPase, 6 |
| Psmd7 | S.c4 | proteasome (prosome, macropain) 26S subunit, non-ATPase, 7 |
| Psmd8 | S.c6 | proteasome (prosome, macropain) 26S subunit, non-ATPase, 8 |
| Psmd8 | S.i1vl7 | proteasome (prosome, macropain) 26S subunit, non-ATPase, 8 |
| Psme1 | S.c3 | proteasome (prosome, macropain) activator subunit 1 (PA28 alpha) |
| Psme1 | S.i4vl7 | proteasome (prosome, macropain) activator subunit 1 (PA28 alpha) |
| Psme1 | S.i2vl7 | proteasome (prosome, macropain) activator subunit 1 (PA28 alpha) |
| Psme1 | S.i3vl7 | proteasome (prosome, macropain) activator subunit 1 (PA28 alpha) |
| Psme3 | S.c7 | proteaseome (prosome, macropain) activator subunit 3 (PA28 gamma, Ki) |
| Ptges | S.i4vl7 | prostaglandin E synthase |
| Ptges | S.c2 | prostaglandin E synthase |
| Ptges3 | S.c3 | prostaglandin E synthase 3 (cytosolic) |
| Ptgr1 | S.c6 | prostaglandin reductase 1 |
| Ptgr1 | S.i1vl7 | prostaglandin reductase 1 |
| Pthlh | S.i1vl7 | parathyroid hormone-like peptide |
| Pthlh | S.c7 | parathyroid hormone-like peptide |
| Pthlh | S.i2vl7 | parathyroid hormone-like peptide |
| Pthlh | S.i3vl7 | parathyroid hormone-like peptide |
| Pthlh | S.i4vl7 | parathyroid hormone-like peptide |
| Ptk2 | S.c8 | PTK2 protein tyrosine kinase 2 |
| Ptk2 | S.i4vl7 | PTK2 protein tyrosine kinase 2 |
| Ptma | S.i4vl7 | prothymosin alpha |
| Ptma | S.c3 | prothymosin alpha |
| Ptma | S.i1vl7 | prothymosin alpha |
| Ptma | S.i3vl7 | prothymosin alpha |
| Ptma | S.i2vl7 | prothymosin alpha |
| Ptp4a1 | S.i1vl7 | protein tyrosine phosphatase 4a1 |
| Ptp4a1 | S.c4 | protein tyrosine phosphatase 4a1 |
| Ptp4a3 | S.c7 | protein tyrosine phosphatase 4a3 |
| Ptplad1 | S.c2 | protein tyrosine phosphatase-like A domain containing 1 |
| Ptplad1 | c5.i3vl7 | protein tyrosine phosphatase-like A domain containing 1 |
| Ptplad1 | S.i2vl7 | protein tyrosine phosphatase-like A domain containing 1 |
| Ptplad1 | S.i3vl7 | protein tyrosine phosphatase-like A domain containing 1 |
| Ptplad1 | S.i4vl7 | protein tyrosine phosphatase-like A domain containing 1 |
| Ptpn2 | S.c6 | protein tyrosine phosphatase, non-receptor type 2 |
| Ptprc | S.c1 | protein tyrosine phosphatase, receptor type, C |
| Ptprf | S.i4vl7 | protein tyrosine phosphatase, receptor type, F |
| Ptprs | S.i4vl7 | protein tyrosine phosphatase, receptor type, S |
| Ptrf | S.i4vl7 | polymerase I and transcript release factor |
| Ptrf | S.c3 | polymerase I and transcript release factor |
| Ptrf | S.i2vl7 | polymerase I and transcript release factor |
| Ptrf | S.i3vl7 | polymerase I and transcript release factor |
| Pttg1ip | S.i3vl7 | pituitary tumor-transforming 1 interacting protein |
| Pttg1ip | S.i4vl7 | pituitary tumor-transforming 1 interacting protein |
| Pttg1ip | S.c3 | pituitary tumor-transforming 1 interacting protein |
| Pvrl2 | S.c6 | poliovirus receptor-related 2 |
| Pvrl2 | S.i1vl7 | poliovirus receptor-related 2 |
| Pxmp2 | S.c7 | peroxisomal membrane protein 2 |
| Pycr2 | S.c8 | pyrroline-5-carboxylate reductase family, member 2 |
| Pycr2 | S.i3vl7 | pyrroline-5-carboxylate reductase family, member 2 |
| Pycr2 | S.i4vl7 | pyrroline-5-carboxylate reductase family, member 2 |
| Pycr2 | S.i2vl7 | pyrroline-5-carboxylate reductase family, member 2 |
| Qk | S.c3 | quaking |
| Qk | S.i3vl7 | quaking |
| Qk | S.c1 | quaking |
| Qk | S.i2vl7 | quaking |
| Qk | S.i4vl7 | quaking |
| Qsox1 | S.i4vl7 | quiescin Q6 sulfhydryl oxidase 1 |
| Qsox1 | S.i2vl7 | quiescin Q6 sulfhydryl oxidase 1 |
| Qsox1 | S.c8 | quiescin Q6 sulfhydryl oxidase 1 |
| R3hcc1 | S.c6 | R3H domain and coiled-coil containing 1 |
| R3hcc1 | S.i4vl7 | R3H domain and coiled-coil containing 1 |
| R3hcc1 | S.i2vl7 | R3H domain and coiled-coil containing 1 |
| R3hcc1 | S.i1vl7 | R3H domain and coiled-coil containing 1 |
| R3hcc1 | S.c7 | R3H domain and coiled-coil containing 1 |
| R3hcc1 | S.i3vl7 | R3H domain and coiled-coil containing 1 |
| Rab1 | S.c8 | RAB1, member RAS oncogene family |
| Rab1 | S.i4vl7 | RAB1, member RAS oncogene family |
| Rab11a | S.i1vl7 | RAB11a, member RAS oncogene family |
| Rab11a | S.c6 | RAB11a, member RAS oncogene family |
| Rab11b | S.c4 | RAB11B, member RAS oncogene family |
| Rab11fip5 | S.c6 | RAB11 family interacting protein 5 (class I) |
| Rab11fip5 | S.i1vl7 | RAB11 family interacting protein 5 (class I) |
| Rab12 | S.c3 | RAB12, member RAS oncogene family |
| Rab12 | S.i3vl7 | RAB12, member RAS oncogene family |
| Rab12 | S.i2vl7 | RAB12, member RAS oncogene family |
| Rab12 | S.i1vl7 | RAB12, member RAS oncogene family |
| Rab12 | S.i4vl7 | RAB12, member RAS oncogene family |
| Rab14 | S.c3 | RAB14, member RAS oncogene family |
| Rab18 | S.i1vl7 | RAB18, member RAS oncogene family |
| Rab18 | S.i3vl7 | RAB18, member RAS oncogene family |
| Rab18 | S.i2vl7 | RAB18, member RAS oncogene family |
| Rab18 | S.c7 | RAB18, member RAS oncogene family |
| Rab18 | S.i4vl7 | RAB18, member RAS oncogene family |
| Rab24 | c5.i3vl7 | RAB24, member RAS oncogene family |
| Rab24 | S.i3vl7 | RAB24, member RAS oncogene family |
| Rab31 | S.i3vl7 | RAB31, member RAS oncogene family |
| Rab31 | S.i2vl7 | RAB31, member RAS oncogene family |
| Rab31 | S.i1vl7 | RAB31, member RAS oncogene family |
| Rab31 | S.c3 | RAB31, member RAS oncogene family |
| Rab34 | S.c3 | RAB34, member of RAS oncogene family |
| Rab34 | S.i3vl7 | RAB34, member of RAS oncogene family |
| Rab34 | S.i4vl7 | RAB34, member of RAS oncogene family |
| Rab3ip | S.i1vl7 | RAB3A interacting protein |
| Rab3ip | S.c8 | RAB3A interacting protein |
| Rab4a | S.i4vl7 | RAB4A, member RAS oncogene family |
| Rab4a | S.c9 | RAB4A, member RAS oncogene family |
| Rab4a | S.i3vl7 | RAB4A, member RAS oncogene family |
| Rac2 | S.i4vl7 | RAS-related C3 botulinum substrate 2 |
| Rac2 | S.c1 | RAS-related C3 botulinum substrate 2 |
| Rad21 | S.c3 | RAD21 homolog (S. pombe) |
| Rad21 | S.i4vl7 | RAD21 homolog (S. pombe) |
| Rad23a | S.c7 | RAD23a homolog (S. cerevisiae) |
| Rad50 | S.i4vl7 | RAD50 homolog (S. cerevisiae) |
| Raf1 | S.c3 | v-raf-leukemia viral oncogene 1 |
| Ralb | S.i3vl7 | v-ral simian leukemia viral oncogene homolog B (ras related) |
| Ralb | S.c4 | v-ral simian leukemia viral oncogene homolog B (ras related) |
| Ralb | S.i1vl7 | v-ral simian leukemia viral oncogene homolog B (ras related) |
| Ramp2 | S.c2 | receptor (calcitonin) activity modifying protein 2 |
| Ran | S.c4 | RAN, member RAS oncogene family |
| Ran | S.i4vl7 | RAN, member RAS oncogene family |
| Ranbp10 | S.i4vl7 | RAN binding protein 10 |
| Ranbp10 | S.c7 | RAN binding protein 10 |
| Ranbp10 | S.i2vl7 | RAN binding protein 10 |
| Ranbp10 | S.i3vl7 | RAN binding protein 10 |
| Ranbp3 | S.c3 | RAN binding protein 3 |
| Ranbp3 | S.i4vl7 | RAN binding protein 3 |
| Rangap1 | S.i4vl7 | RAN GTPase activating protein 1 |
| Rangap1 | S.c8 | RAN GTPase activating protein 1 |
| Rarres2 | S.c1 | retinoic acid receptor responder (tazarotene induced) 2 |
| Rarres2 | S.i4vl7 | retinoic acid receptor responder (tazarotene induced) 2 |
| Rars | S.c9 | arginyl-tRNA synthetase |
| Rasa1 | S.c4 | RAS p21 protein activator 1 |
| Rasa1 | S.i4vl7 | RAS p21 protein activator 1 |
| Rasa1 | S.i1vl7 | RAS p21 protein activator 1 |
| Rasa3 | S.c2 | RAS p21 protein activator 3 |
| Rasa3 | S.i4vl7 | RAS p21 protein activator 3 |
| Rasa4 | S.c1 | RAS p21 protein activator 4 |
| Rasd1 | S.i4vl7 | RAS, dexamethasone-induced 1 |
| Rasd1 | S.c2 | RAS, dexamethasone-induced 1 |
| Rasd1 | S.i2vl7 | RAS, dexamethasone-induced 1 |
| Rasip1 | S.c2 | Ras interacting protein 1 |
| Rasip1 | S.i2vl7 | Ras interacting protein 1 |
| Rasip1 | S.i3vl7 | Ras interacting protein 1 |
| Rassf1 | S.i3vl7 | Ras association (RalGDS/AF-6) domain family member 1 |
| Rassf1 | S.i2vl7 | Ras association (RalGDS/AF-6) domain family member 1 |
| Rassf1 | c5.i3vl7 | Ras association (RalGDS/AF-6) domain family member 1 |
| Rb1 | c5.i3vl7 | retinoblastoma 1 |
| Rb1 | S.i4vl7 | retinoblastoma 1 |
| Rb1 | S.i1vl7 | retinoblastoma 1 |
| Rb1 | S.i3vl7 | retinoblastoma 1 |
| Rb1 | S.i2vl7 | retinoblastoma 1 |
| Rbbp7 | S.c4 | retinoblastoma binding protein 7 |
| Rbbp7 | S.i3vl7 | retinoblastoma binding protein 7 |
| Rbbp7 | S.i4vl7 | retinoblastoma binding protein 7 |
| Rbbp9 | S.i4vl7 | retinoblastoma binding protein 9 |
| Rbck1 | S.c2 | RanBP-type and C3HC4-type zinc finger containing 1 |
| Rbck1 | S.i4vl7 | RanBP-type and C3HC4-type zinc finger containing 1 |
| Rbfox2 | S.i4vl7 | RNA binding protein, fox-1 homolog (C. elegans) 2 |
| Rbfox2 | S.i3vl7 | RNA binding protein, fox-1 homolog (C. elegans) 2 |
| Rbfox2 | S.c8 | RNA binding protein, fox-1 homolog (C. elegans) 2 |
| Rbfox2 | S.i2vl7 | RNA binding protein, fox-1 homolog (C. elegans) 2 |
| Rbm17 | S.c6 | RNA binding motif protein 17 |
| Rbm18 | S.c6 | RNA binding motif protein 18 |
| Rbm3 | S.i1vl7 | RNA binding motif protein 3 |
| Rbm42 | S.i4vl7 | RNA binding motif protein 42 |
| Rbm42 | S.i2vl7 | RNA binding motif protein 42 |
| Rbm42 | S.c3 | RNA binding motif protein 42 |
| Rbms1 | S.i3vl7 | RNA binding motif, single stranded interacting protein 1 |
| Rbms1 | S.c4 | RNA binding motif, single stranded interacting protein 1 |
| Rbms1 | S.i2vl7 | RNA binding motif, single stranded interacting protein 1 |
| Rbms1 | S.i4vl7 | RNA binding motif, single stranded interacting protein 1 |
| Rbmxl1 | S.c4 | RNA binding motif protein, X linked-like-1 |
| Rbp1 | S.c2 | retinol binding protein 1, cellular |
| Rbp1 | S.i2vl7 | retinol binding protein 1, cellular |
| Rbp1 | S.i4vl7 | retinol binding protein 1, cellular |
| Rbp1 | S.i3vl7 | retinol binding protein 1, cellular |
| Rbp4 | S.i4vl7 | retinol binding protein 4, plasma |
| Rbp4 | S.i2vl7 | retinol binding protein 4, plasma |
| Rbp4 | S.i3vl7 | retinol binding protein 4, plasma |
| Rbp4 | S.c2 | retinol binding protein 4, plasma |
| Rbpj | S.c2 | recombination signal binding protein for immunoglobulin kappa J region |
| Rbpms | S.c3 | RNA binding protein gene with multiple splicing |
| Rbpms | S.i4vl7 | RNA binding protein gene with multiple splicing |
| Rbpms | S.i1vl7 | RNA binding protein gene with multiple splicing |
| Rbpms | S.i3vl7 | RNA binding protein gene with multiple splicing |
| Rbpms | S.i2vl7 | RNA binding protein gene with multiple splicing |
| Rcan1 | S.i2vl7 | regulator of calcineurin 1 |
| Rcan1 | S.i1vl7 | regulator of calcineurin 1 |
| Rcan1 | S.i4vl7 | regulator of calcineurin 1 |
| Rcan1 | S.i3vl7 | regulator of calcineurin 1 |
| Rcan1 | c5.i3vl7 | regulator of calcineurin 1 |
| Rcan3 | S.c6 | regulator of calcineurin 3 |
| Rcn1 | S.i2vl7 | reticulocalbin 1 |
| Rcn1 | S.c1 | reticulocalbin 1 |
| Rcn1 | S.i4vl7 | reticulocalbin 1 |
| Rcn2 | S.c6 | reticulocalbin 2 |
| Rcn2 | S.i1vl7 | reticulocalbin 2 |
| Rdh11 | S.i4vl7 | retinol dehydrogenase 11 |
| Rdh11 | S.i2vl7 | retinol dehydrogenase 11 |
| Rdh11 | S.i1vl7 | retinol dehydrogenase 11 |
| Rdh11 | S.c7 | retinol dehydrogenase 11 |
| Rdh11 | S.i3vl7 | retinol dehydrogenase 11 |
| Reep6 | S.i2vl7 | receptor accessory protein 6 |
| Reep6 | S.i4vl7 | receptor accessory protein 6 |
| Reep6 | S.i3vl7 | receptor accessory protein 6 |
| Reep6 | S.i1vl7 | receptor accessory protein 6 |
| Reep6 | S.c7 | receptor accessory protein 6 |
| Reg2 | S.c8 | regenerating islet-derived 2 |
| Reg2 | S.i4vl7 | regenerating islet-derived 2 |
| Rela | c5.i3vl7 | v-rel reticuloendotheliosis viral oncogene homolog A (avian) |
| Rela | S.i3vl7 | v-rel reticuloendotheliosis viral oncogene homolog A (avian) |
| Relb | S.c1 | avian reticuloendotheliosis viral (v-rel) oncogene related B |
| Reln | S.c8 | reelin |
| Renbp | S.c4 | renin binding protein |
| Renbp | S.i4vl7 | renin binding protein |
| Repin1 | S.i4vl7 | replication initiator 1 |
| Ret | S.i1vl7 | ret proto-oncogene |
| Ret | S.i2vl7 | ret proto-oncogene |
| Ret | S.i4vl7 | ret proto-oncogene |
| Retn | S.i4vl7 | resistin |
| Retn | S.c2 | resistin |
| Retsat | S.c2 | retinol saturase (all trans retinol 13,14 reductase) |
| Retsat | S.i4vl7 | retinol saturase (all trans retinol 13,14 reductase) |
| Retsat | S.i3vl7 | retinol saturase (all trans retinol 13,14 reductase) |
| Rfk | S.c7 | riboflavin kinase |
| Rfk | S.i4vl7 | riboflavin kinase |
| Rfk | S.i2vl7 | riboflavin kinase |
| Rfk | S.i3vl7 | riboflavin kinase |
| Rfk | S.c8 | riboflavin kinase |
| Rgcc | S.c1 | regulator of cell cycle |
| Rgs10 | S.c1 | regulator of G-protein signalling 10 |
| Rgs19 | S.i4vl7 | regulator of G-protein signaling 19 |
| Rgs19 | S.c1 | regulator of G-protein signaling 19 |
| Rgs2 | S.i4vl7 | regulator of G-protein signaling 2 |
| Rgs2 | S.c1 | regulator of G-protein signaling 2 |
| Rgs3 | S.c3 | regulator of G-protein signaling 3 |
| Rho | S.i4vl7 | rhodopsin |
| Rho | S.c7 | rhodopsin |
| Rho | S.i3vl7 | rhodopsin |
| Rho | S.i2vl7 | rhodopsin |
| Rhob | S.i1vl7 | ras homolog gene family, member B |
| Rhob | c5.i3vl7 | ras homolog gene family, member B |
| Rhob | S.i2vl7 | ras homolog gene family, member B |
| Rhob | S.i4vl7 | ras homolog gene family, member B |
| Rhob | S.i3vl7 | ras homolog gene family, member B |
| Rhoc | c5.i3vl7 | ras homolog gene family, member C |
| Rhoc | S.i1vl7 | ras homolog gene family, member C |
| Rhoc | S.i3vl7 | ras homolog gene family, member C |
| Rhod | S.i3vl7 | ras homolog gene family, member D |
| Rhoj | S.i2vl7 | ras homolog gene family, member J |
| Rhoq | S.i1vl7 | ras homolog gene family, member Q |
| Rhoq | S.i3vl7 | ras homolog gene family, member Q |
| Rhoq | S.c6 | ras homolog gene family, member Q |
| Rhou | S.c6 | ras homolog gene family, member U |
| Rhpn2 | S.c9 | rhophilin, Rho GTPase binding protein 2 |
| Rin2 | S.i2vl7 | Ras and Rab interactor 2 |
| Rin2 | S.c4 | Ras and Rab interactor 2 |
| Rin2 | S.i1vl7 | Ras and Rab interactor 2 |
| Rin2 | S.i3vl7 | Ras and Rab interactor 2 |
| Rin2 | S.i4vl7 | Ras and Rab interactor 2 |
| Rmnd5a | S.c4 | required for meiotic nuclear division 5 homolog A (S. cerevisiae) |
| Rnase1 | c5.i3vl7 | ribonuclease, RNase A family, 1 (pancreatic) |
| Rnase1 | S.i1vl7 | ribonuclease, RNase A family, 1 (pancreatic) |
| Rnase1 | S.i3vl7 | ribonuclease, RNase A family, 1 (pancreatic) |
| Rnase1 | S.i2vl7 | ribonuclease, RNase A family, 1 (pancreatic) |
| Rnase4 | S.c2 | ribonuclease, RNase A family 4 |
| Rnase4 | S.i4vl7 | ribonuclease, RNase A family 4 |
| Rnasek | S.c3 | ribonuclease, RNase K |
| Rnasek | S.i4vl7 | ribonuclease, RNase K |
| Rnasek | S.c4 | ribonuclease, RNase K |
| Rnasek | S.i3vl7 | ribonuclease, RNase K |
| Rnasek | S.i1vl7 | ribonuclease, RNase K |
| Rnasek | S.i2vl7 | ribonuclease, RNase K |
| Rnf103 | S.c4 | ring finger protein 103 |
| Rnf103 | S.i3vl7 | ring finger protein 103 |
| Rnf103 | S.i2vl7 | ring finger protein 103 |
| Rnf103 | S.i4vl7 | ring finger protein 103 |
| Rnf103 | S.i1vl7 | ring finger protein 103 |
| Rnf11 | S.i1vl7 | ring finger protein 11 |
| Rnf11 | S.c6 | ring finger protein 11 |
| Rnf145 | S.i4vl7 | ring finger protein 145 |
| Rnf145 | S.c7 | ring finger protein 145 |
| Rnf145 | S.i3vl7 | ring finger protein 145 |
| Rnf145 | S.i2vl7 | ring finger protein 145 |
| Rnf149 | S.c6 | ring finger protein 149 |
| Rnf149 | S.i1vl7 | ring finger protein 149 |
| Rnf167 | S.c9 | ring finger protein 167 |
| Rnf19a | S.i1vl7 | ring finger protein 19A |
| Rnf19a | S.i4vl7 | ring finger protein 19A |
| Rnf19a | S.c4 | ring finger protein 19A |
| Rnf4 | S.c4 | ring finger protein 4 |
| Rnf44 | S.c4 | ring finger protein 44 |
| Rnf44 | S.i1vl7 | ring finger protein 44 |
| Rnf5 | S.i4vl7 | ring finger protein 5 |
| Rnf5 | S.c7 | ring finger protein 5 |
| Rnf5 | S.i3vl7 | ring finger protein 5 |
| Rnf5 | S.i1vl7 | ring finger protein 5 |
| Rnh1 | S.c3 | ribonuclease/angiogenin inhibitor 1 |
| Rnh1 | S.i3vl7 | ribonuclease/angiogenin inhibitor 1 |
| Rnh1 | S.i4vl7 | ribonuclease/angiogenin inhibitor 1 |
| Rnh1 | S.i2vl7 | ribonuclease/angiogenin inhibitor 1 |
| Rnh1 | S.i1vl7 | ribonuclease/angiogenin inhibitor 1 |
| Rnmtl1 | S.c8 | RNA methyltransferase like 1 |
| Rnps1 | S.c3 | ribonucleic acid binding protein S1 |
| Rnps1 | S.i3vl7 | ribonucleic acid binding protein S1 |
| Rogdi | S.c9 | rogdi homolog (Drosophila) |
| Rora | S.i4vl7 | RAR-related orphan receptor alpha |
| Rora | S.i3vl7 | RAR-related orphan receptor alpha |
| Rora | S.i1vl7 | RAR-related orphan receptor alpha |
| Rora | S.i2vl7 | RAR-related orphan receptor alpha |
| Rora | S.c7 | RAR-related orphan receptor alpha |
| Rpl22l1 | S.c7 | ribosomal protein L22 like 1 |
| Rpl22l1 | S.i2vl7 | ribosomal protein L22 like 1 |
| Rpl22l1 | S.i4vl7 | ribosomal protein L22 like 1 |
| Rpl22l1 | S.i1vl7 | ribosomal protein L22 like 1 |
| Rpl22l1 | S.i3vl7 | ribosomal protein L22 like 1 |
| Rpl34 | S.i4vl7 | ribosomal protein L34 |
| Rpl34 | S.c8 | ribosomal protein L34 |
| Rpl34 | S.i2vl7 | ribosomal protein L34 |
| Rpl34-ps1 | S.c8 | ribosomal protein L34, pseudogene 1 |
| Rpl34-ps1 | S.i4vl7 | ribosomal protein L34, pseudogene 1 |
| Rpl34-ps1 | S.i2vl7 | ribosomal protein L34, pseudogene 1 |
| Rpl7 | S.c7 | ribosomal protein L7 |
| Rpl7 | S.i4vl7 | ribosomal protein L7 |
| Rpl7 | S.i2vl7 | ribosomal protein L7 |
| Rpl7 | S.i3vl7 | ribosomal protein L7 |
| Rpl7 | S.i1vl7 | ribosomal protein L7 |
| Rpp14 | S.c7 | ribonuclease P 14 subunit |
| Rpp14 | S.i2vl7 | ribonuclease P 14 subunit |
| Rpp14 | S.i3vl7 | ribonuclease P 14 subunit |
| Rpp21 | S.i2vl7 | ribonuclease P 21 subunit |
| Rpp30 | S.i1vl7 | ribonuclease P/MRP 30 subunit |
| Rpp30 | S.c4 | ribonuclease P/MRP 30 subunit |
| Rps13 | S.c7 | ribosomal protein S13 |
| Rps28 | S.i4vl7 | ribosomal protein S28 |
| Rps28 | S.i2vl7 | ribosomal protein S28 |
| Rps28 | S.c8 | ribosomal protein S28 |
| Rraga | S.c7 | Ras-related GTP binding A |
| Rragc | S.c4 | Ras-related GTP binding C |
| Rragc | S.i4vl7 | Ras-related GTP binding C |
| Rragc | S.i2vl7 | Ras-related GTP binding C |
| Rragc | S.i3vl7 | Ras-related GTP binding C |
| Rragc | S.i1vl7 | Ras-related GTP binding C |
| Rras | S.c4 | Harvey rat sarcoma oncogene, subgroup R |
| Rrp1 | S.i3vl7 | ribosomal RNA processing 1 homolog (S. cerevisiae) |
| Rrp1 | S.i2vl7 | ribosomal RNA processing 1 homolog (S. cerevisiae) |
| Rrp1 | S.c3 | ribosomal RNA processing 1 homolog (S. cerevisiae) |
| Rsu1 | S.i2vl7 | Ras suppressor protein 1 |
| Rsu1 | S.c4 | Ras suppressor protein 1 |
| Rsu1 | S.i4vl7 | Ras suppressor protein 1 |
| Rsu1 | S.i1vl7 | Ras suppressor protein 1 |
| Rtn3 | S.c8 | reticulon 3 |
| Rtn3 | S.i2vl7 | reticulon 3 |
| Rtn4 | S.i2vl7 | reticulon 4 |
| Rtn4 | S.i3vl7 | reticulon 4 |
| Rtn4 | S.i1vl7 | reticulon 4 |
| Rtn4 | S.c4 | reticulon 4 |
| Rtn4 | S.i4vl7 | reticulon 4 |
| Rufy1 | S.c3 | RUN and FYVE domain containing 1 |
| Rundc3a | S.i2vl7 | RUN domain containing 3A |
| Rundc3a | S.i4vl7 | RUN domain containing 3A |
| Rundc3a | S.c8 | RUN domain containing 3A |
| Rundc3a | S.i3vl7 | RUN domain containing 3A |
| Runx2 | S.c7 | runt related transcription factor 2 |
| Ryk | S.i2vl7 | receptor-like tyrosine kinase |
| Ryk | S.c4 | receptor-like tyrosine kinase |
| Ryk | S.i4vl7 | receptor-like tyrosine kinase |
| Ryk | S.i3vl7 | receptor-like tyrosine kinase |
| Ryk | S.i1vl7 | receptor-like tyrosine kinase |
| S100a1 | S.i1vl7 | S100 calcium binding protein A1 |
| S100a10 | S.i1vl7 | S100 calcium binding protein A10 (calpactin) |
| S100a10 | S.i2vl7 | S100 calcium binding protein A10 (calpactin) |
| S100a10 | S.c4 | S100 calcium binding protein A10 (calpactin) |
| S100a10 | S.i4vl7 | S100 calcium binding protein A10 (calpactin) |
| S100a11 | S.i2vl7 | S100 calcium binding protein A11 (calgizzarin) |
| S100a11 | S.i4vl7 | S100 calcium binding protein A11 (calgizzarin) |
| S100a11 | S.i1vl7 | S100 calcium binding protein A11 (calgizzarin) |
| S100a11 | S.i3vl7 | S100 calcium binding protein A11 (calgizzarin) |
| S100a11 | S.c4 | S100 calcium binding protein A11 (calgizzarin) |
| S100a13 | S.i4vl7 | S100 calcium binding protein A13 |
| S100a13 | S.i3vl7 | S100 calcium binding protein A13 |
| S100a13 | S.i1vl7 | S100 calcium binding protein A13 |
| S100a13 | S.c4 | S100 calcium binding protein A13 |
| S100a13 | c5.i3vl7 | S100 calcium binding protein A13 |
| S100a13 | S.i2vl7 | S100 calcium binding protein A13 |
| S100a6 | S.c3 | S100 calcium binding protein A6 (calcyclin) |
| S100a6 | S.i4vl7 | S100 calcium binding protein A6 (calcyclin) |
| S100a8 | S.i4vl7 | S100 calcium binding protein A8 (calgranulin A) |
| S100a8 | S.c2 | S100 calcium binding protein A8 (calgranulin A) |
| Samhd1 | S.i4vl7 | SAM domain and HD domain, 1 |
| Samhd1 | S.c4 | SAM domain and HD domain, 1 |
| Sap30l | S.c4 | SAP30-like |
| Sap30l | S.i1vl7 | SAP30-like |
| Sap30l | S.i2vl7 | SAP30-like |
| Sarnp | S.i4vl7 | SAP domain containing ribonucleoprotein |
| Sarnp | S.c3 | SAP domain containing ribonucleoprotein |
| Sash1 | S.c3 | SAM and SH3 domain containing 1 |
| Sat1 | S.c6 | spermidine/spermine N1-acetyl transferase 1 |
| Sat1 | S.i1vl7 | spermidine/spermine N1-acetyl transferase 1 |
| Sat1 | S.i2vl7 | spermidine/spermine N1-acetyl transferase 1 |
| Sbds | S.c4 | Shwachman-Bodian-Diamond syndrome homolog (human) |
| Sbds | S.i1vl7 | Shwachman-Bodian-Diamond syndrome homolog (human) |
| Sbf2 | S.c3 | SET binding factor 2 |
| Sc4mol | S.c7 | sterol-C4-methyl oxidase-like |
| Scarb1 | S.i1vl7 | scavenger receptor class B, member 1 |
| Scarb1 | S.c7 | scavenger receptor class B, member 1 |
| Scarb2 | c5.i3vl7 | scavenger receptor class B, member 2 |
| Scarb2 | S.i3vl7 | scavenger receptor class B, member 2 |
| Scarb2 | S.i1vl7 | scavenger receptor class B, member 2 |
| Scarb2 | S.i4vl7 | scavenger receptor class B, member 2 |
| Scarb2 | S.i2vl7 | scavenger receptor class B, member 2 |
| Scd1 | S.i4vl7 | stearoyl-Coenzyme A desaturase 1 |
| Scd1 | S.c8 | stearoyl-Coenzyme A desaturase 1 |
| Scd2 | S.i2vl7 | stearoyl-Coenzyme A desaturase 2 |
| Scd2 | S.i1vl7 | stearoyl-Coenzyme A desaturase 2 |
| Scd2 | S.i3vl7 | stearoyl-Coenzyme A desaturase 2 |
| Scd2 | S.c7 | stearoyl-Coenzyme A desaturase 2 |
| Scn1b | S.c1 | sodium channel, voltage-gated, type I, beta |
| Scn1b | S.i4vl7 | sodium channel, voltage-gated, type I, beta |
| Scp2 | S.c1 | sterol carrier protein 2, liver |
| Scp2 | S.i4vl7 | sterol carrier protein 2, liver |
| Scpep1 | S.c3 | serine carboxypeptidase 1 |
| Sdcbp | S.i1vl7 | syndecan binding protein |
| Sdcbp | S.c6 | syndecan binding protein |
| Sdha | S.i4vl7 | succinate dehydrogenase complex, subunit A, flavoprotein (Fp) |
| Sdhc | S.c2 | succinate dehydrogenase complex, subunit C, integral membrane protein |
| Sdpr | S.i4vl7 | serum deprivation response |
| Sdpr | S.i2vl7 | serum deprivation response |
| Sdpr | S.i3vl7 | serum deprivation response |
| Sdpr | S.c3 | serum deprivation response |
| Sec11a | S.i3vl7 | SEC11 homolog A (S. cerevisiae) |
| Sec11a | c5.i3vl7 | SEC11 homolog A (S. cerevisiae) |
| Sec11a | S.i1vl7 | SEC11 homolog A (S. cerevisiae) |
| Sec11a | S.i2vl7 | SEC11 homolog A (S. cerevisiae) |
| Sec14l1 | S.i4vl7 | SEC14-like 1 (S. cerevisiae) |
| Sec14l1 | S.c7 | SEC14-like 1 (S. cerevisiae) |
| Sec14l2 | S.i4vl7 | SEC14-like 2 (S. cerevisiae) |
| Sec14l2 | S.c8 | SEC14-like 2 (S. cerevisiae) |
| Sec23b | S.i4vl7 | SEC23B (S. cerevisiae) |
| Sec23b | S.c7 | SEC23B (S. cerevisiae) |
| Sec23b | S.i1vl7 | SEC23B (S. cerevisiae) |
| Sec61b | S.i4vl7 | Sec61 beta subunit |
| Sec61b | S.c8 | Sec61 beta subunit |
| Sec61b | S.i3vl7 | Sec61 beta subunit |
| Seh1l | S.i4vl7 | SEH1-like (S. cerevisiae |
| Sel1l | c5.i3vl7 | sel-1 suppressor of lin-12-like (C. elegans) |
| Sel1l | S.i3vl7 | sel-1 suppressor of lin-12-like (C. elegans) |
| Selenbp1 | S.c8 | selenium binding protein 1 |
| Selenbp1 | S.i4vl7 | selenium binding protein 1 |
| Selenbp2 | S.i4vl7 | selenium binding protein 2 |
| Selenbp2 | S.c8 | selenium binding protein 2 |
| Selp | S.c1 | selectin, platelet |
| Selplg | S.c1 | selectin, platelet (p-selectin) ligand |
| Sema6c | S.i3vl7 | sema domain, transmembrane domain (TM), and cytoplasmic domain, (semaphorin) 6C |
| Sema6c | S.i2vl7 | sema domain, transmembrane domain (TM), and cytoplasmic domain, (semaphorin) 6C |
| Sema6c | S.i4vl7 | sema domain, transmembrane domain (TM), and cytoplasmic domain, (semaphorin) 6C |
| Sema6c | S.i1vl7 | sema domain, transmembrane domain (TM), and cytoplasmic domain, (semaphorin) 6C |
| Sema7a | S.c7 | sema domain, immunoglobulin domain (Ig), and GPI membrane anchor, (semaphorin) 7A |
| 10-Sep | S.i2vl7 | septin 10 |
| 10-Sep | S.i1vl7 | septin 10 |
| 10-Sep | S.i4vl7 | septin 10 |
| 10-Sep | S.c3 | septin 10 |
| 2-Sep | S.i2vl7 | septin 2 |
| 2-Sep | S.i1vl7 | septin 2 |
| 2-Sep | S.i4vl7 | septin 2 |
| 2-Sep | S.c4 | septin 2 |
| 4-Sep | S.i3vl7 | septin 4 |
| 4-Sep | S.c1 | septin 4 |
| 4-Sep | S.i4vl7 | septin 4 |
| 9-Sep | S.c2 | septin 9 |
| 9-Sep | S.i1vl7 | septin 9 |
| 9-Sep | S.i4vl7 | septin 9 |
| 9-Sep | S.i2vl7 | septin 9 |
| 9-Sep | S.c4 | septin 9 |
| 9-Sep | S.i3vl7 | septin 9 |
| Serp1 | S.c8 | stress-associated endoplasmic reticulum protein 1 |
| Serp1 | S.i1vl7 | stress-associated endoplasmic reticulum protein 1 |
| Serp1 | S.i2vl7 | stress-associated endoplasmic reticulum protein 1 |
| Serp1 | S.i3vl7 | stress-associated endoplasmic reticulum protein 1 |
| Serp1 | S.i4vl7 | stress-associated endoplasmic reticulum protein 1 |
| Serpina3c | S.c1 | serine (or cysteine) peptidase inhibitor, clade A, member 3C |
| Serpina3c | S.i4vl7 | serine (or cysteine) peptidase inhibitor, clade A, member 3C |
| Serpina3n | S.c2 | serine (or cysteine) peptidase inhibitor, clade A, member 3N |
| Serpina3n | S.i4vl7 | serine (or cysteine) peptidase inhibitor, clade A, member 3N |
| Serpinb5 | S.i2vl7 | serine (or cysteine) peptidase inhibitor, clade B, member 5 |
| Serpinb5 | S.i1vl7 | serine (or cysteine) peptidase inhibitor, clade B, member 5 |
| Serpinb5 | S.i4vl7 | serine (or cysteine) peptidase inhibitor, clade B, member 5 |
| Serpinb5 | S.i3vl7 | serine (or cysteine) peptidase inhibitor, clade B, member 5 |
| Serpinb5 | S.c6 | serine (or cysteine) peptidase inhibitor, clade B, member 5 |
| Serpinb6a | S.i4vl7 | serine (or cysteine) peptidase inhibitor, clade B, member 6a |
| Serpinb6a | S.c4 | serine (or cysteine) peptidase inhibitor, clade B, member 6a |
| Serpinb9 | S.i2vl7 | serine (or cysteine) peptidase inhibitor, clade B, member 9 |
| Serpinb9 | S.i3vl7 | serine (or cysteine) peptidase inhibitor, clade B, member 9 |
| Serpinb9 | S.c3 | serine (or cysteine) peptidase inhibitor, clade B, member 9 |
| Serpinf1 | S.i3vl7 | serine (or cysteine) peptidase inhibitor, clade F, member 1 |
| Serpinf1 | S.i4vl7 | serine (or cysteine) peptidase inhibitor, clade F, member 1 |
| Serpinf1 | S.c2 | serine (or cysteine) peptidase inhibitor, clade F, member 1 |
| Serpinf2 | S.i4vl7 | serine (or cysteine) peptidase inhibitor, clade F, member 2 |
| Serpinf2 | S.c8 | serine (or cysteine) peptidase inhibitor, clade F, member 2 |
| Serping1 | S.i4vl7 | serine (or cysteine) peptidase inhibitor, clade G, member 1 |
| Serping1 | S.i3vl7 | serine (or cysteine) peptidase inhibitor, clade G, member 1 |
| Serping1 | S.i2vl7 | serine (or cysteine) peptidase inhibitor, clade G, member 1 |
| Serping1 | S.c2 | serine (or cysteine) peptidase inhibitor, clade G, member 1 |
| Serpinh1 | S.i4vl7 | serine (or cysteine) peptidase inhibitor, clade H, member 1 |
| Serpinh1 | S.c3 | serine (or cysteine) peptidase inhibitor, clade H, member 1 |
| Sesn1 | S.i2vl7 | sestrin 1 |
| Sesn1 | S.c2 | sestrin 1 |
| Sesn1 | S.i4vl7 | sestrin 1 |
| Set | S.i2vl7 | SET nuclear oncogene |
| Set | S.i3vl7 | SET nuclear oncogene |
| Set | S.c3 | SET nuclear oncogene |
| Setd8 | S.c8 | SET domain containing (lysine methyltransferase) 8 |
| Sf1 | S.c8 | splicing factor 1 |
| Sf1 | S.i2vl7 | splicing factor 1 |
| Sf1 | S.i4vl7 | splicing factor 1 |
| Sf3a2 | S.i4vl7 | splicing factor 3a, subunit 2 |
| Sf3a3 | S.i3vl7 | splicing factor 3a, subunit 3 |
| Sf3a3 | S.i4vl7 | splicing factor 3a, subunit 3 |
| Sf3a3 | S.c3 | splicing factor 3a, subunit 3 |
| Sfn | S.c3 | stratifin |
| Sfn | S.i3vl7 | stratifin |
| Sfpq | S.i3vl7 | splicing factor proline/glutamine rich (polypyrimidine tract binding protein associated) |
| Sfpq | S.c2 | splicing factor proline/glutamine rich (polypyrimidine tract binding protein associated) |
| Sfrp1 | S.i4vl7 | secreted frizzled-related protein 1 |
| Sftpb | S.c7 | surfactant associated protein B |
| Sftpb | S.i3vl7 | surfactant associated protein B |
| Sftpb | S.i1vl7 | surfactant associated protein B |
| Sftpb | S.i4vl7 | surfactant associated protein B |
| Sftpb | S.i2vl7 | surfactant associated protein B |
| Sfxn1 | S.i4vl7 | sideroflexin 1 |
| Sfxn1 | S.c3 | sideroflexin 1 |
| Sfxn2 | S.c7 | sideroflexin 2 |
| Sgce | S.c2 | sarcoglycan, epsilon |
| Sgip1 | S.c7 | SH3-domain GRB2-like (endophilin) interacting protein 1 |
| Sgk1 | S.c1 | serum/glucocorticoid regulated kinase 1 |
| Sgpl1 | S.c2 | sphingosine phosphate lyase 1 |
| Sgpl1 | S.i2vl7 | sphingosine phosphate lyase 1 |
| Sgpl1 | S.c3 | sphingosine phosphate lyase 1 |
| Sgpl1 | S.i3vl7 | sphingosine phosphate lyase 1 |
| Sh3bgrl | S.c3 | SH3-binding domain glutamic acid-rich protein like |
| Sh3bgrl | S.i2vl7 | SH3-binding domain glutamic acid-rich protein like |
| Sh3bgrl | S.i4vl7 | SH3-binding domain glutamic acid-rich protein like |
| Sh3bgrl | S.i3vl7 | SH3-binding domain glutamic acid-rich protein like |
| Sh3bgrl2 | S.i4vl7 | SH3 domain binding glutamic acid-rich protein like 2 |
| Sh3bgrl2 | S.c8 | SH3 domain binding glutamic acid-rich protein like 2 |
| Sh3bp2 | S.c8 | SH3-domain binding protein 2 |
| Sh3bp2 | S.i4vl7 | SH3-domain binding protein 2 |
| Sh3bp5 | S.i4vl7 | SH3-domain binding protein 5 (BTK-associated) |
| Sh3bp5 | S.c7 | SH3-domain binding protein 5 (BTK-associated) |
| Shf | S.c8 | Src homology 2 domain containing F |
| Shfm1 | S.i2vl7 | split hand/foot malformation (ectrodactyly) type 1 |
| Shisa5 | S.i2vl7 | shisa homolog 5 (Xenopus laevis) |
| Shisa5 | S.i1vl7 | shisa homolog 5 (Xenopus laevis) |
| Shisa5 | c5.i3vl7 | shisa homolog 5 (Xenopus laevis) |
| Shisa5 | S.i4vl7 | shisa homolog 5 (Xenopus laevis) |
| Shisa5 | S.i3vl7 | shisa homolog 5 (Xenopus laevis) |
| Shkbp1 | S.c2 | Sh3kbp1 binding protein 1 |
| Shoc2 | S.c9 | soc-2 (suppressor of clear) homolog (C. elegans) |
| Shoc2 | S.i4vl7 | soc-2 (suppressor of clear) homolog (C. elegans) |
| Shroom3 | S.i4vl7 | shroom family member 3 |
| Shroom3 | S.c3 | shroom family member 3 |
| Sidt2 | S.i4vl7 | SID1 transmembrane family, member 2 |
| Sidt2 | S.c8 | SID1 transmembrane family, member 2 |
| Sidt2 | S.i3vl7 | SID1 transmembrane family, member 2 |
| Sigmar1 | S.i1vl7 | sigma non-opioid intracellular receptor 1 |
| Sigmar1 | S.c7 | sigma non-opioid intracellular receptor 1 |
| Sipa1 | S.c1 | signal-induced proliferation associated gene 1 |
| Sirpa | S.i3vl7 | signal-regulatory protein alpha |
| Sirpa | S.i1vl7 | signal-regulatory protein alpha |
| Sirpa | S.i4vl7 | signal-regulatory protein alpha |
| Sirpa | S.c4 | signal-regulatory protein alpha |
| Sirpa | S.i2vl7 | signal-regulatory protein alpha |
| Skap2 | S.i3vl7 | src family associated phosphoprotein 2 |
| Skap2 | S.c4 | src family associated phosphoprotein 2 |
| Skap2 | S.i1vl7 | src family associated phosphoprotein 2 |
| Skap2 | S.i2vl7 | src family associated phosphoprotein 2 |
| Skap2 | S.i4vl7 | src family associated phosphoprotein 2 |
| Ski | S.i4vl7 | ski sarcoma viral oncogene homolog (avian) |
| Ski | c5.i3vl7 | ski sarcoma viral oncogene homolog (avian) |
| Ski | S.i3vl7 | ski sarcoma viral oncogene homolog (avian) |
| Ski | S.i2vl7 | ski sarcoma viral oncogene homolog (avian) |
| Ski | S.i1vl7 | ski sarcoma viral oncogene homolog (avian) |
| Skp1a | S.c3 | S-phase kinase-associated protein 1A |
| Skp1a | S.i4vl7 | S-phase kinase-associated protein 1A |
| Skp1a | S.i2vl7 | S-phase kinase-associated protein 1A |
| Skp1a | S.i3vl7 | S-phase kinase-associated protein 1A |
| Slc11a1 | S.i4vl7 | solute carrier family 11 (proton-coupled divalent metal ion transporters), member 1 |
| Slc11a1 | S.c1 | solute carrier family 11 (proton-coupled divalent metal ion transporters), member 1 |
| Slc12a2 | S.c4 | solute carrier family 12, member 2 |
| Slc12a2 | S.i4vl7 | solute carrier family 12, member 2 |
| Slc12a2 | S.i2vl7 | solute carrier family 12, member 2 |
| Slc12a2 | S.i3vl7 | solute carrier family 12, member 2 |
| Slc12a2 | S.i1vl7 | solute carrier family 12, member 2 |
| Slc12a7 | S.i4vl7 | solute carrier family 12, member 7 |
| Slc12a7 | S.c3 | solute carrier family 12, member 7 |
| Slc12a7 | S.i2vl7 | solute carrier family 12, member 7 |
| Slc12a7 | S.i3vl7 | solute carrier family 12, member 7 |
| Slc16a2 | S.i2vl7 | solute carrier family 16 (monocarboxylic acid transporters), member 2 |
| Slc16a2 | c5.i3vl7 | solute carrier family 16 (monocarboxylic acid transporters), member 2 |
| Slc16a2 | S.i3vl7 | solute carrier family 16 (monocarboxylic acid transporters), member 2 |
| Slc1a5 | S.i4vl7 | solute carrier family 1 (neutral amino acid transporter), member 5 |
| Slc1a5 | S.c2 | solute carrier family 1 (neutral amino acid transporter), member 5 |
| Slc1a5 | S.i3vl7 | solute carrier family 1 (neutral amino acid transporter), member 5 |
| Slc20a1 | S.i1vl7 | solute carrier family 20, member 1 |
| Slc20a1 | S.c9 | solute carrier family 20, member 1 |
| Slc20a1 | S.i4vl7 | solute carrier family 20, member 1 |
| Slc22a18 | S.i2vl7 | solute carrier family 22 (organic cation transporter), member 18 |
| Slc23a3 | S.c8 | solute carrier family 23 (nucleobase transporters), member 3 |
| Slc23a3 | S.i4vl7 | solute carrier family 23 (nucleobase transporters), member 3 |
| Slc25a1 | S.i2vl7 | solute carrier family 25 (mitochondrial carrier, citrate transporter), member 1 |
| Slc25a1 | S.c7 | solute carrier family 25 (mitochondrial carrier, citrate transporter), member 1 |
| Slc25a10 | S.c7 | solute carrier family 25 (mitochondrial carrier, dicarboxylate transporter), member 10 |
| Slc25a17 | S.i4vl7 | solute carrier family 25 (mitochondrial carrier, peroxisomal membrane protein), member 17 |
| Slc25a17 | S.c8 | solute carrier family 25 (mitochondrial carrier, peroxisomal membrane protein), member 17 |
| Slc25a17 | S.i3vl7 | solute carrier family 25 (mitochondrial carrier, peroxisomal membrane protein), member 17 |
| Slc25a17 | S.i2vl7 | solute carrier family 25 (mitochondrial carrier, peroxisomal membrane protein), member 17 |
| Slc25a20 | S.i3vl7 | solute carrier family 25 (mitochondrial carnitine/acylcarnitine translocase), member 20 |
| Slc25a20 | S.i2vl7 | solute carrier family 25 (mitochondrial carnitine/acylcarnitine translocase), member 20 |
| Slc25a20 | c5.i3vl7 | solute carrier family 25 (mitochondrial carnitine/acylcarnitine translocase), member 20 |
| Slc25a20 | S.i4vl7 | solute carrier family 25 (mitochondrial carnitine/acylcarnitine translocase), member 20 |
| Slc25a4 | S.i2vl7 | solute carrier family 25 (mitochondrial carrier, adenine nucleotide translocator), member 4 |
| Slc25a4 | S.i1vl7 | solute carrier family 25 (mitochondrial carrier, adenine nucleotide translocator), member 4 |
| Slc25a4 | S.i4vl7 | solute carrier family 25 (mitochondrial carrier, adenine nucleotide translocator), member 4 |
| Slc25a4 | S.i3vl7 | solute carrier family 25 (mitochondrial carrier, adenine nucleotide translocator), member 4 |
| Slc25a4 | S.c3 | solute carrier family 25 (mitochondrial carrier, adenine nucleotide translocator), member 4 |
| Slc27a1 | S.i2vl7 | solute carrier family 27 (fatty acid transporter), member 1 |
| Slc27a1 | S.i3vl7 | solute carrier family 27 (fatty acid transporter), member 1 |
| Slc27a1 | S.c2 | solute carrier family 27 (fatty acid transporter), member 1 |
| Slc27a3 | S.i3vl7 | solute carrier family 27 (fatty acid transporter), member 3 |
| Slc27a3 | S.i4vl7 | solute carrier family 27 (fatty acid transporter), member 3 |
| Slc27a3 | S.c8 | solute carrier family 27 (fatty acid transporter), member 3 |
| Slc27a4 | S.i4vl7 | solute carrier family 27 (fatty acid transporter), member 4 |
| Slc29a1 | S.c7 | solute carrier family 29 (nucleoside transporters), member 1 |
| Slc2a1 | S.i1vl7 | solute carrier family 2 (facilitated glucose transporter), member 1 |
| Slc2a1 | S.c7 | solute carrier family 2 (facilitated glucose transporter), member 1 |
| Slc2a1 | S.i4vl7 | solute carrier family 2 (facilitated glucose transporter), member 1 |
| Slc2a4 | S.c2 | solute carrier family 2 (facilitated glucose transporter), member 4 |
| Slc30a4 | S.c8 | solute carrier family 30 (zinc transporter), member 4 |
| Slc30a4 | S.i2vl7 | solute carrier family 30 (zinc transporter), member 4 |
| Slc30a4 | S.i3vl7 | solute carrier family 30 (zinc transporter), member 4 |
| Slc30a4 | S.i4vl7 | solute carrier family 30 (zinc transporter), member 4 |
| Slc31a1 | S.i4vl7 | solute carrier family 31, member 1 |
| Slc31a1 | S.c9 | solute carrier family 31, member 1 |
| Slc34a2 | S.c8 | solute carrier family 34 (sodium phosphate), member 2 |
| Slc34a2 | S.i4vl7 | solute carrier family 34 (sodium phosphate), member 2 |
| Slc34a2 | S.i2vl7 | solute carrier family 34 (sodium phosphate), member 2 |
| Slc35b1 | S.c7 | solute carrier family 35, member B1 |
| Slc35b2 | S.c8 | solute carrier family 35, member B2 |
| Slc35b2 | S.i4vl7 | solute carrier family 35, member B2 |
| Slc35c2 | S.i1vl7 | solute carrier family 35, member C2 |
| Slc35c2 | S.c7 | solute carrier family 35, member C2 |
| Slc35e1 | S.c7 | solute carrier family 35, member E1 |
| Slc38a10 | S.c7 | solute carrier family 38, member 10 |
| Slc38a2 | S.c7 | solute carrier family 38, member 2 |
| Slc38a2 | S.i1vl7 | solute carrier family 38, member 2 |
| Slc39a6 | S.c8 | solute carrier family 39 (metal ion transporter), member 6 |
| Slc39a8 | S.c9 | solute carrier family 39 (metal ion transporter), member 8 |
| Slc3a2 | S.i3vl7 | solute carrier family 3 (activators of dibasic and neutral amino acid transport), member 2 |
| Slc3a2 | S.i4vl7 | solute carrier family 3 (activators of dibasic and neutral amino acid transport), member 2 |
| Slc3a2 | S.i1vl7 | solute carrier family 3 (activators of dibasic and neutral amino acid transport), member 2 |
| Slc3a2 | S.i2vl7 | solute carrier family 3 (activators of dibasic and neutral amino acid transport), member 2 |
| Slc3a2 | c5.i3vl7 | solute carrier family 3 (activators of dibasic and neutral amino acid transport), member 2 |
| Slc41a1 | S.c8 | solute carrier family 41, member 1 |
| Slc41a1 | S.i4vl7 | solute carrier family 41, member 1 |
| Slc4a7 | S.c7 | solute carrier family 4, sodium bicarbonate cotransporter, member 7 |
| Slc4a7 | S.i1vl7 | solute carrier family 4, sodium bicarbonate cotransporter, member 7 |
| Slc4a7 | S.i2vl7 | solute carrier family 4, sodium bicarbonate cotransporter, member 7 |
| Slc4a7 | S.i4vl7 | solute carrier family 4, sodium bicarbonate cotransporter, member 7 |
| Slc4a7 | S.i3vl7 | solute carrier family 4, sodium bicarbonate cotransporter, member 7 |
| Slc50a1 | S.i4vl7 | solute carrier family 50 (sugar transporter), member 1 |
| Slc50a1 | S.i3vl7 | solute carrier family 50 (sugar transporter), member 1 |
| Slc50a1 | S.i1vl7 | solute carrier family 50 (sugar transporter), member 1 |
| Slc50a1 | S.i2vl7 | solute carrier family 50 (sugar transporter), member 1 |
| Slc50a1 | S.c7 | solute carrier family 50 (sugar transporter), member 1 |
| Slc52a2 | S.i1vl7 | solute carrier protein 52, member 2 |
| Slc52a2 | S.i4vl7 | solute carrier protein 52, member 2 |
| Slc5a1 | S.c9 | solute carrier family 5 (sodium/glucose cotransporter), member 1 |
| Slc5a6 | S.i2vl7 | solute carrier family 5 (sodium-dependent vitamin transporter), member 6 |
| Slc5a6 | S.i4vl7 | solute carrier family 5 (sodium-dependent vitamin transporter), member 6 |
| Slc5a6 | S.i1vl7 | solute carrier family 5 (sodium-dependent vitamin transporter), member 6 |
| Slc5a6 | S.i3vl7 | solute carrier family 5 (sodium-dependent vitamin transporter), member 6 |
| Slc5a6 | S.c7 | solute carrier family 5 (sodium-dependent vitamin transporter), member 6 |
| Slc6a6 | S.i4vl7 | solute carrier family 6 (neurotransmitter transporter, taurine), member 6 |
| Slc6a6 | S.c2 | solute carrier family 6 (neurotransmitter transporter, taurine), member 6 |
| Slc6a8 | S.i4vl7 | solute carrier family 6 (neurotransmitter transporter, creatine), member 8 |
| Slc7a5 | S.c7 | solute carrier family 7 (cationic amino acid transporter, y+ system), member 5 |
| Slc7a5 | S.i1vl7 | solute carrier family 7 (cationic amino acid transporter, y+ system), member 5 |
| Slc7a7 | S.i1vl7 | solute carrier family 7 (cationic amino acid transporter, y+ system), member 7 |
| Slc7a7 | S.i3vl7 | solute carrier family 7 (cationic amino acid transporter, y+ system), member 7 |
| Slc7a7 | S.i2vl7 | solute carrier family 7 (cationic amino acid transporter, y+ system), member 7 |
| Slc7a7 | S.c7 | solute carrier family 7 (cationic amino acid transporter, y+ system), member 7 |
| Slc9a3r1 | S.i3vl7 | solute carrier family 9 (sodium/hydrogen exchanger), member 3 regulator 1 |
| Slc9a3r1 | S.i4vl7 | solute carrier family 9 (sodium/hydrogen exchanger), member 3 regulator 1 |
| Slc9a3r1 | S.i2vl7 | solute carrier family 9 (sodium/hydrogen exchanger), member 3 regulator 1 |
| Slc9a3r1 | S.i1vl7 | solute carrier family 9 (sodium/hydrogen exchanger), member 3 regulator 1 |
| Slc9a3r1 | S.c8 | solute carrier family 9 (sodium/hydrogen exchanger), member 3 regulator 1 |
| Slpi | S.i4vl7 | secretory leukocyte peptidase inhibitor |
| Slpi | S.i2vl7 | secretory leukocyte peptidase inhibitor |
| Slpi | S.i3vl7 | secretory leukocyte peptidase inhibitor |
| Slpi | c5.i3vl7 | secretory leukocyte peptidase inhibitor |
| Slpi | S.i1vl7 | secretory leukocyte peptidase inhibitor |
| Sltm | S.i1vl7 | SAFB-like, transcription modulator |
| Sltm | S.i3vl7 | SAFB-like, transcription modulator |
| Sltm | S.i4vl7 | SAFB-like, transcription modulator |
| Sltm | S.i2vl7 | SAFB-like, transcription modulator |
| Sltm | c5.i3vl7 | SAFB-like, transcription modulator |
| Slu7 | S.c4 | SLU7 splicing factor homolog (S. cerevisiae) |
| Smad1 | S.i3vl7 | SMAD family member 1 |
| Smad1 | S.c4 | SMAD family member 1 |
| Smad1 | S.i2vl7 | SMAD family member 1 |
| Smad1 | S.i1vl7 | SMAD family member 1 |
| Smad1 | S.i4vl7 | SMAD family member 1 |
| Smad4 | S.c3 | SMAD family member 4 |
| Smad4 | S.i4vl7 | SMAD family member 4 |
| Smad4 | S.i2vl7 | SMAD family member 4 |
| Smad4 | S.i3vl7 | SMAD family member 4 |
| Smap1 | S.i2vl7 | small ArfGAP 1 |
| Smap1 | S.c3 | small ArfGAP 1 |
| Smap1 | S.i3vl7 | small ArfGAP 1 |
| Smap1 | S.i4vl7 | small ArfGAP 1 |
| Smarca2 | S.c3 | SWI/SNF related, matrix associated, actin dependent regulator of chromatin, subfamily a, member 2 |
| Smarca2 | S.i4vl7 | SWI/SNF related, matrix associated, actin dependent regulator of chromatin, subfamily a, member 2 |
| Smarca4 | S.i4vl7 | SWI/SNF related, matrix associated, actin dependent regulator of chromatin, subfamily a, member 4 |
| Smarca4 | S.i2vl7 | SWI/SNF related, matrix associated, actin dependent regulator of chromatin, subfamily a, member 4 |
| Smarca4 | S.c3 | SWI/SNF related, matrix associated, actin dependent regulator of chromatin, subfamily a, member 4 |
| Smarca4 | S.i3vl7 | SWI/SNF related, matrix associated, actin dependent regulator of chromatin, subfamily a, member 4 |
| Smarcb1 | S.i4vl7 | SWI/SNF related, matrix associated, actin dependent regulator of chromatin, subfamily b, member 1 |
| Smarcb1 | S.c3 | SWI/SNF related, matrix associated, actin dependent regulator of chromatin, subfamily b, member 1 |
| Smarcb1 | S.i3vl7 | SWI/SNF related, matrix associated, actin dependent regulator of chromatin, subfamily b, member 1 |
| Smarcd2 | S.c8 | SWI/SNF related, matrix associated, actin dependent regulator of chromatin, subfamily d, member 2 |
| Smarcd2 | S.i3vl7 | SWI/SNF related, matrix associated, actin dependent regulator of chromatin, subfamily d, member 2 |
| Smarcd2 | S.i2vl7 | SWI/SNF related, matrix associated, actin dependent regulator of chromatin, subfamily d, member 2 |
| Smarcd2 | S.i4vl7 | SWI/SNF related, matrix associated, actin dependent regulator of chromatin, subfamily d, member 2 |
| Smim1 | S.c7 | small integral membrane protein 1 |
| Smim1 | S.i3vl7 | small integral membrane protein 1 |
| Smim7 | S.c4 | small integral membrane protein 7 |
| Smn1 | S.c3 | survival motor neuron 1 |
| Smndc1 | S.i4vl7 | survival motor neuron domain containing 1 |
| Smndc1 | S.c3 | survival motor neuron domain containing 1 |
| Smpdl3a | S.c9 | sphingomyelin phosphodiesterase, acid-like 3A |
| Smpdl3a | S.i4vl7 | sphingomyelin phosphodiesterase, acid-like 3A |
| Snai2 | S.c1 | snail homolog 2 (Drosophila) |
| Snapin | S.i3vl7 | SNAP-associated protein |
| Snapin | S.i1vl7 | SNAP-associated protein |
| Snapin | S.c4 | SNAP-associated protein |
| Snapin | S.i2vl7 | SNAP-associated protein |
| Snapin | S.i4vl7 | SNAP-associated protein |
| Sncg | S.c1 | synuclein, gamma |
| Sncg | S.i4vl7 | synuclein, gamma |
| Snd1 | S.c7 | staphylococcal nuclease and tudor domain containing 1 |
| Snd1 | S.i1vl7 | staphylococcal nuclease and tudor domain containing 1 |
| Snrk | S.c2 | SNF related kinase |
| Snrnp40 | S.c3 | small nuclear ribonucleoprotein 40 (U5) |
| Snrpa | S.c2 | small nuclear ribonucleoprotein polypeptide A |
| Snrpb2 | S.i4vl7 | U2 small nuclear ribonucleoprotein B |
| Snx10 | S.c4 | sorting nexin 10 |
| Snx12 | S.c4 | sorting nexin 12 |
| Snx12 | S.i3vl7 | sorting nexin 12 |
| Snx12 | S.i4vl7 | sorting nexin 12 |
| Snx12 | S.i2vl7 | sorting nexin 12 |
| Snx4 | S.c4 | sorting nexin 4 |
| Snx5 | S.c3 | sorting nexin 5 |
| Snx5 | S.i4vl7 | sorting nexin 5 |
| Snx6 | S.i1vl7 | sorting nexin 6 |
| Snx6 | S.i2vl7 | sorting nexin 6 |
| Snx6 | S.c4 | sorting nexin 6 |
| Snx6 | S.i3vl7 | sorting nexin 6 |
| Snx6 | S.i4vl7 | sorting nexin 6 |
| Snx9 | S.c3 | sorting nexin 9 |
| Snx9 | S.i4vl7 | sorting nexin 9 |
| Socs3 | S.i1vl7 | suppressor of cytokine signaling 3 |
| Socs3 | S.i2vl7 | suppressor of cytokine signaling 3 |
| Socs3 | S.i4vl7 | suppressor of cytokine signaling 3 |
| Sod1 | S.c2 | superoxide dismutase 1, soluble |
| Sod1 | S.i4vl7 | superoxide dismutase 1, soluble |
| Sod3 | S.i2vl7 | superoxide dismutase 3, extracellular |
| Sod3 | S.c2 | superoxide dismutase 3, extracellular |
| Sod3 | S.i3vl7 | superoxide dismutase 3, extracellular |
| Sorbs1 | S.i3vl7 | sorbin and SH3 domain containing 1 |
| Sorbs1 | S.i4vl7 | sorbin and SH3 domain containing 1 |
| Sorbs1 | S.i2vl7 | sorbin and SH3 domain containing 1 |
| Sorbs1 | S.c3 | sorbin and SH3 domain containing 1 |
| Sowahc | S.c3 | sosondowah ankyrin repeat domain family member C |
| Sowahc | S.i2vl7 | sosondowah ankyrin repeat domain family member C |
| Sowahc | S.i4vl7 | sosondowah ankyrin repeat domain family member C |
| Sox10 | S.c9 | SRY (sex determining region Y)-box 10 |
| Sox18 | S.c2 | SRY (sex determining region Y)-box 18 |
| Sox18 | S.c1 | SRY (sex determining region Y)-box 18 |
| Sox18 | S.i4vl7 | SRY (sex determining region Y)-box 18 |
| Sox4 | S.i1vl7 | SRY (sex determining region Y)-box 4 |
| Sox4 | c5.i3vl7 | SRY (sex determining region Y)-box 4 |
| Sox4 | S.i2vl7 | SRY (sex determining region Y)-box 4 |
| Sox4 | S.i3vl7 | SRY (sex determining region Y)-box 4 |
| Sp1 | S.c7 | trans-acting transcription factor 1 |
| Sp3 | S.c3 | trans-acting transcription factor 3 |
| Sparc | S.c3 | secreted acidic cysteine rich glycoprotein |
| Sparcl1 | S.i3vl7 | SPARC-like 1 |
| Sparcl1 | S.i1vl7 | SPARC-like 1 |
| Sparcl1 | S.i4vl7 | SPARC-like 1 |
| Sparcl1 | S.c3 | SPARC-like 1 |
| Specc1 | S.c4 | sperm antigen with calponin homology and coiled-coil domains 1 |
| Specc1 | S.i1vl7 | sperm antigen with calponin homology and coiled-coil domains 1 |
| Speg | S.i2vl7 | SPEG complex locus |
| Speg | S.c8 | SPEG complex locus |
| Spg20 | S.c6 | spastic paraplegia 20, spartin (Troyer syndrome) homolog (human) |
| Spg21 | S.i3vl7 | spastic paraplegia 21 homolog (human) |
| Spg21 | S.i1vl7 | spastic paraplegia 21 homolog (human) |
| Spg21 | S.i4vl7 | spastic paraplegia 21 homolog (human) |
| Spg21 | S.c3 | spastic paraplegia 21 homolog (human) |
| Spg21 | S.i2vl7 | spastic paraplegia 21 homolog (human) |
| Spi1 | S.c1 | spleen focus forming virus (SFFV) proviral integration oncogene |
| Spin1 | S.c2 | spindlin 1 |
| Spin1 | S.i2vl7 | spindlin 1 |
| Spint1 | S.i2vl7 | serine protease inhibitor, Kunitz type 1 |
| Spint1 | S.i3vl7 | serine protease inhibitor, Kunitz type 1 |
| Spint1 | S.i1vl7 | serine protease inhibitor, Kunitz type 1 |
| Spint1 | c5.i3vl7 | serine protease inhibitor, Kunitz type 1 |
| Spns1 | S.i2vl7 | spinster homolog 1 |
| Sppl3 | S.c3 | signal peptide peptidase 3 |
| Sppl3 | S.i4vl7 | signal peptide peptidase 3 |
| Sppl3 | S.i2vl7 | signal peptide peptidase 3 |
| Sppl3 | S.i3vl7 | signal peptide peptidase 3 |
| Spred2 | S.i4vl7 | sprouty-related, EVH1 domain containing 2 |
| Spred2 | S.c1 | sprouty-related, EVH1 domain containing 2 |
| Spsb1 | S.c1 | splA/ryanodine receptor domain and SOCS box containing 1 |
| Sptan1 | S.i2vl7 | spectrin alpha, non-erythrocytic 1 |
| Sptan1 | S.c3 | spectrin alpha, non-erythrocytic 1 |
| Sptan1 | S.i4vl7 | spectrin alpha, non-erythrocytic 1 |
| Sptan1 | S.i3vl7 | spectrin alpha, non-erythrocytic 1 |
| Sptbn1 | S.i4vl7 | spectrin beta, non-erythrocytic 1 |
| Sptbn1 | S.c3 | spectrin beta, non-erythrocytic 1 |
| Sptbn1 | S.i2vl7 | spectrin beta, non-erythrocytic 1 |
| Sptlc1 | S.c6 | serine palmitoyltransferase, long chain base subunit 1 |
| Sptlc1 | S.i1vl7 | serine palmitoyltransferase, long chain base subunit 1 |
| Sptlc2 | S.c4 | serine palmitoyltransferase, long chain base subunit 2 |
| Sptlc2 | S.i3vl7 | serine palmitoyltransferase, long chain base subunit 2 |
| Sptlc2 | S.i1vl7 | serine palmitoyltransferase, long chain base subunit 2 |
| Sptlc2 | S.i4vl7 | serine palmitoyltransferase, long chain base subunit 2 |
| Sptlc2 | S.i2vl7 | serine palmitoyltransferase, long chain base subunit 2 |
| Sqle | S.i1vl7 | squalene epoxidase |
| Sqle | S.c7 | squalene epoxidase |
| Sqle | S.i2vl7 | squalene epoxidase |
| Sqle | S.i3vl7 | squalene epoxidase |
| Sqle | S.i4vl7 | squalene epoxidase |
| Sqstm1 | S.i1vl7 | sequestosome 1 |
| Sqstm1 | S.c4 | sequestosome 1 |
| Sqstm1 | S.i2vl7 | sequestosome 1 |
| Sqstm1 | S.i3vl7 | sequestosome 1 |
| Srebf1 | S.c7 | sterol regulatory element binding transcription factor 1 |
| Sri | S.i1vl7 | sorcin |
| Sri | S.i2vl7 | sorcin |
| Sri | S.i4vl7 | sorcin |
| Sri | S.c4 | sorcin |
| Srm | S.i1vl7 | spermidine synthase |
| Srm | S.i3vl7 | spermidine synthase |
| Srm | S.i4vl7 | spermidine synthase |
| Srm | S.c7 | spermidine synthase |
| Srp54b | S.c7 | signal recognition particle 54B |
| Srpk2 | S.i4vl7 | serine/arginine-rich protein specific kinase 2 |
| Srpk2 | S.i2vl7 | serine/arginine-rich protein specific kinase 2 |
| Srpk2 | S.c3 | serine/arginine-rich protein specific kinase 2 |
| Srpk2 | S.i3vl7 | serine/arginine-rich protein specific kinase 2 |
| Srpr | S.c7 | signal recognition particle receptor ('docking protein') |
| Srpr | S.i4vl7 | signal recognition particle receptor ('docking protein') |
| Srpr | S.c8 | signal recognition particle receptor ('docking protein') |
| Srsf2 | S.i1vl7 | serine/arginine-rich splicing factor 2 |
| Srsf2 | S.c4 | serine/arginine-rich splicing factor 2 |
| Srsf9 | S.c3 | serine/arginine-rich splicing factor 9 |
| Srxn1 | S.c6 | sulfiredoxin 1 homolog (S. cerevisiae) |
| Ss18 | S.i4vl7 | synovial sarcoma translocation, Chromosome 18 |
| Ss18 | S.i2vl7 | synovial sarcoma translocation, Chromosome 18 |
| Ss18 | S.c3 | synovial sarcoma translocation, Chromosome 18 |
| Ss18 | S.i3vl7 | synovial sarcoma translocation, Chromosome 18 |
| Ssbp4 | S.c2 | single stranded DNA binding protein 4 |
| Ssbp4 | S.i2vl7 | single stranded DNA binding protein 4 |
| Sspn | S.c3 | sarcospan |
| Ssr1 | S.i1vl7 | signal sequence receptor, alpha |
| Ssr1 | S.i4vl7 | signal sequence receptor, alpha |
| Ssr1 | S.c7 | signal sequence receptor, alpha |
| Ssr2 | S.c8 | signal sequence receptor, beta |
| Ssr2 | S.i4vl7 | signal sequence receptor, beta |
| Ssr3 | S.c7 | signal sequence receptor, gamma |
| Ssrp1 | S.i4vl7 | structure specific recognition protein 1 |
| Ssrp1 | S.c4 | structure specific recognition protein 1 |
| Ssrp1 | S.i3vl7 | structure specific recognition protein 1 |
| Sssca1 | S.c4 | Sjogren's syndrome/scleroderma autoantigen 1 homolog (human) |
| Sssca1 | S.i1vl7 | Sjogren's syndrome/scleroderma autoantigen 1 homolog (human) |
| Ssx2ip | S.c9 | synovial sarcoma, X breakpoint 2 interacting protein |
| Ssx2ip | S.i4vl7 | synovial sarcoma, X breakpoint 2 interacting protein |
| St3gal1 | S.i4vl7 | ST3 beta-galactoside alpha-2,3-sialyltransferase 1 |
| St3gal4 | S.c7 | ST3 beta-galactoside alpha-2,3-sialyltransferase 4 |
| St3gal4 | S.i3vl7 | ST3 beta-galactoside alpha-2,3-sialyltransferase 4 |
| St5 | S.i1vl7 | suppression of tumorigenicity 5 |
| St5 | S.c6 | suppression of tumorigenicity 5 |
| St6gal1 | S.i2vl7 | beta galactoside alpha 2,6 sialyltransferase 1 |
| St6gal1 | S.i4vl7 | beta galactoside alpha 2,6 sialyltransferase 1 |
| St6gal1 | S.i3vl7 | beta galactoside alpha 2,6 sialyltransferase 1 |
| St6gal1 | S.i1vl7 | beta galactoside alpha 2,6 sialyltransferase 1 |
| St6gal1 | S.c7 | beta galactoside alpha 2,6 sialyltransferase 1 |
| St6gal1 | S.c8 | beta galactoside alpha 2,6 sialyltransferase 1 |
| St6galnac2 | S.c9 | ST6 (alpha-N-acetyl-neuraminyl-2,3-beta-galactosyl-1,3)-N-acetylgalactosaminide alpha-2,6-sialyltransferase 2 |
| St7 | S.i1vl7 | suppression of tumorigenicity 7 |
| St7 | S.c7 | suppression of tumorigenicity 7 |
| St7 | S.i2vl7 | suppression of tumorigenicity 7 |
| St7 | S.i4vl7 | suppression of tumorigenicity 7 |
| St7 | S.i3vl7 | suppression of tumorigenicity 7 |
| St8sia4 | S.c1 | ST8 alpha-N-acetyl-neuraminide alpha-2,8-sialyltransferase 4 |
| Stab1 | S.i4vl7 | stabilin 1 |
| Stab1 | S.c1 | stabilin 1 |
| Stag2 | S.c3 | stromal antigen 2 |
| Stam | S.c6 | signal transducing adaptor molecule (SH3 domain and ITAM motif) 1 |
| Stam2 | S.c6 | signal transducing adaptor molecule (SH3 domain and ITAM motif) 2 |
| Stard10 | S.i4vl7 | START domain containing 10 |
| Stard10 | S.c8 | START domain containing 10 |
| Stard3 | S.i4vl7 | START domain containing 3 |
| Stard3 | S.i2vl7 | START domain containing 3 |
| Stard3 | c5.i3vl7 | START domain containing 3 |
| Stard3 | S.i3vl7 | START domain containing 3 |
| Stard3 | S.i1vl7 | START domain containing 3 |
| Stat1 | S.c2 | signal transducer and activator of transcription 1 |
| Stat1 | S.i4vl7 | signal transducer and activator of transcription 1 |
| Stat3 | c5.i3vl7 | signal transducer and activator of transcription 3 |
| Stat3 | S.i3vl7 | signal transducer and activator of transcription 3 |
| Stat3 | S.i4vl7 | signal transducer and activator of transcription 3 |
| Stat3 | S.i2vl7 | signal transducer and activator of transcription 3 |
| Stat3 | S.i1vl7 | signal transducer and activator of transcription 3 |
| Stat5b | S.c7 | signal transducer and activator of transcription 5B |
| Stat6 | c5.i3vl7 | signal transducer and activator of transcription 6 |
| Stat6 | S.i3vl7 | signal transducer and activator of transcription 6 |
| Stim1 | S.c1 | stromal interaction molecule 1 |
| Stk10 | S.c1 | serine/threonine kinase 10 |
| Stk10 | S.i4vl7 | serine/threonine kinase 10 |
| Stk40 | S.i2vl7 | serine/threonine kinase 40 |
| Stmn1 | S.i4vl7 | stathmin 1 |
| Stmn1 | S.i2vl7 | stathmin 1 |
| Stmn1 | S.i1vl7 | stathmin 1 |
| Stmn1 | S.c6 | stathmin 1 |
| Stmn1 | S.i3vl7 | stathmin 1 |
| Stx12 | S.c4 | syntaxin 12 |
| Stx2 | S.c1 | syntaxin 2 |
| Stx3 | S.c9 | syntaxin 3 |
| Stx3 | S.i2vl7 | syntaxin 3 |
| Stx4a | S.i1vl7 | syntaxin 4A (placental) |
| Stx4a | S.c4 | syntaxin 4A (placental) |
| Stx4a | S.i3vl7 | syntaxin 4A (placental) |
| Stx4a | S.i4vl7 | syntaxin 4A (placental) |
| Stx4a | S.i2vl7 | syntaxin 4A (placental) |
| Stxbp2 | S.c8 | syntaxin binding protein 2 |
| Stxbp2 | S.i4vl7 | syntaxin binding protein 2 |
| Stxbp2 | S.i2vl7 | syntaxin binding protein 2 |
| Stxbp2 | S.i3vl7 | syntaxin binding protein 2 |
| Sucla2 | S.c3 | succinate-Coenzyme A ligase, ADP-forming, beta subunit |
| Sucla2 | S.i4vl7 | succinate-Coenzyme A ligase, ADP-forming, beta subunit |
| Suclg2 | S.i3vl7 | succinate-Coenzyme A ligase, GDP-forming, beta subunit |
| Suclg2 | S.c7 | succinate-Coenzyme A ligase, GDP-forming, beta subunit |
| Suclg2 | S.i2vl7 | succinate-Coenzyme A ligase, GDP-forming, beta subunit |
| Sugt1 | S.c4 | SGT1, suppressor of G2 allele of SKP1 (S. cerevisiae) |
| Sugt1 | S.i1vl7 | SGT1, suppressor of G2 allele of SKP1 (S. cerevisiae) |
| Sumf1 | S.i3vl7 | sulfatase modifying factor 1 |
| Sumf1 | c5.i3vl7 | sulfatase modifying factor 1 |
| Sumf1 | S.i4vl7 | sulfatase modifying factor 1 |
| Sun1 | S.i1vl7 | Sad1 and UNC84 domain containing 1 |
| Sun2 | S.i1vl7 | Sad1 and UNC84 domain containing 2 |
| Supt4a | S.i3vl7 | suppressor of Ty 4A |
| Supt4a | S.i4vl7 | suppressor of Ty 4A |
| Supt4a | S.i2vl7 | suppressor of Ty 4A |
| Supt4a | c5.i3vl7 | suppressor of Ty 4A |
| Supt4a | S.i1vl7 | suppressor of Ty 4A |
| Surf4 | S.c9 | surfeit gene 4 |
| Surf4 | S.i4vl7 | surfeit gene 4 |
| Syf2 | S.c4 | SYF2 homolog, RNA splicing factor (S. cerevisiae) |
| Syf2 | S.i4vl7 | SYF2 homolog, RNA splicing factor (S. cerevisiae) |
| Syf2 | S.i1vl7 | SYF2 homolog, RNA splicing factor (S. cerevisiae) |
| Syk | S.c1 | spleen tyrosine kinase |
| Syne4 | S.c8 | spectrin repeat containing, nuclear envelope family member 4 |
| Syne4 | S.i2vl7 | spectrin repeat containing, nuclear envelope family member 4 |
| Syne4 | S.i4vl7 | spectrin repeat containing, nuclear envelope family member 4 |
| Syngr1 | S.i1vl7 | synaptogyrin 1 |
| Syngr1 | S.i2vl7 | synaptogyrin 1 |
| Syngr1 | S.c7 | synaptogyrin 1 |
| Synpo | S.c3 | synaptopodin |
| Synpo | S.i4vl7 | synaptopodin |
| Synpo | S.i2vl7 | synaptopodin |
| Taf11 | S.c6 | TAF11 RNA polymerase II, TATA box binding protein (TBP)-associated factor |
| Taf1d | S.c3 | TATA box binding protein (Tbp)-associated factor, RNA polymerase I, D |
| Tagln2 | S.i4vl7 | transgelin 2 |
| Tagln2 | S.c4 | transgelin 2 |
| Tagln2 | S.i1vl7 | transgelin 2 |
| Tagln2 | S.i3vl7 | transgelin 2 |
| Tagln2 | S.i2vl7 | transgelin 2 |
| Tap1 | S.c4 | transporter 1, ATP-binding cassette, sub-family B (MDR/TAP) |
| Tap1 | S.i1vl7 | transporter 1, ATP-binding cassette, sub-family B (MDR/TAP) |
| Tap1 | S.i4vl7 | transporter 1, ATP-binding cassette, sub-family B (MDR/TAP) |
| Tap2 | S.c2 | transporter 2, ATP-binding cassette, sub-family B (MDR/TAP) |
| Tapbp | S.c2 | TAP binding protein |
| Tax1bp1 | S.c8 | Tax1 (human T cell leukemia virus type I) binding protein 1 |
| Tax1bp1 | S.i2vl7 | Tax1 (human T cell leukemia virus type I) binding protein 1 |
| Tax1bp3 | c5.i3vl7 | Tax1 (human T cell leukemia virus type I) binding protein 3 |
| Tax1bp3 | S.i1vl7 | Tax1 (human T cell leukemia virus type I) binding protein 3 |
| Tax1bp3 | S.i2vl7 | Tax1 (human T cell leukemia virus type I) binding protein 3 |
| Tax1bp3 | S.i3vl7 | Tax1 (human T cell leukemia virus type I) binding protein 3 |
| Tax1bp3 | S.i4vl7 | Tax1 (human T cell leukemia virus type I) binding protein 3 |
| Tbc1d10a | S.i4vl7 | TBC1 domain family, member 10a |
| Tbc1d23 | S.c1 | TBC1 domain family, member 23 |
| Tbc1d24 | S.i2vl7 | TBC1 domain family, member 24 |
| Tbc1d24 | S.c8 | TBC1 domain family, member 24 |
| Tbc1d24 | S.i4vl7 | TBC1 domain family, member 24 |
| Tbc1d24 | S.i3vl7 | TBC1 domain family, member 24 |
| Tbc1d24 | S.i1vl7 | TBC1 domain family, member 24 |
| Tbca | S.c6 | tubulin cofactor A |
| Tbca | S.i1vl7 | tubulin cofactor A |
| Tbl1xr1 | S.c4 | transducin (beta)-like 1X-linked receptor 1 |
| Tbl3 | S.i4vl7 | transducin (beta)-like 3 |
| Tbl3 | S.c7 | transducin (beta)-like 3 |
| Tbl3 | S.i1vl7 | transducin (beta)-like 3 |
| Tcea1 | S.i2vl7 | transcription elongation factor A (SII) 1 |
| Tcea1 | S.i3vl7 | transcription elongation factor A (SII) 1 |
| Tcea1 | S.c2 | transcription elongation factor A (SII) 1 |
| Tcea1 | S.i4vl7 | transcription elongation factor A (SII) 1 |
| Tcea2 | S.c8 | transcription elongation factor A (SII), 2 |
| Tceb1 | S.c4 | transcription elongation factor B (SIII), polypeptide 1 |
| Tceb3 | S.c8 | transcription elongation factor B (SIII), polypeptide 3 |
| Tcf12 | S.c4 | transcription factor 12 |
| Tcf4 | S.i4vl7 | transcription factor 4 |
| Tcf4 | S.c1 | transcription factor 4 |
| Tcf4 | S.i3vl7 | transcription factor 4 |
| Tcirg1 | S.c2 | T cell, immune regulator 1, ATPase, H+ transporting, lysosomal V0 protein A3 |
| Tcirg1 | S.i4vl7 | T cell, immune regulator 1, ATPase, H+ transporting, lysosomal V0 protein A3 |
| Tcn2 | S.i4vl7 | transcobalamin 2 |
| Tcn2 | S.c8 | transcobalamin 2 |
| Tcta | S.c4 | T cell leukemia translocation altered gene |
| Tcta | S.c8 | T cell leukemia translocation altered gene |
| Tdrp | S.c2 | testis development related protein |
| Tdrp | S.i3vl7 | testis development related protein |
| Tead2 | S.i2vl7 | TEA domain family member 2 |
| Tead2 | S.i1vl7 | TEA domain family member 2 |
| Tead4 | S.i2vl7 | TEA domain family member 4 |
| Tead4 | S.c6 | TEA domain family member 4 |
| Tek | S.i3vl7 | endothelial-specific receptor tyrosine kinase |
| Tek | S.c1 | endothelial-specific receptor tyrosine kinase |
| Ten1 | S.c7 | TEN1 telomerase capping complex subunit |
| Tesk1 | S.c2 | testis specific protein kinase 1 |
| Tfip11 | S.i4vl7 | tuftelin interacting protein 11 |
| Tfrc | S.i3vl7 | transferrin receptor |
| Tfrc | S.i1vl7 | transferrin receptor |
| Tfrc | S.c7 | transferrin receptor |
| Tfrc | S.i4vl7 | transferrin receptor |
| Tfrc | S.i2vl7 | transferrin receptor |
| Tgfb1 | S.i3vl7 | transforming growth factor, beta 1 |
| Tgfb1 | S.c2 | transforming growth factor, beta 1 |
| Tgfb3 | c5.i3vl7 | transforming growth factor, beta 3 |
| Tgfb3 | S.i3vl7 | transforming growth factor, beta 3 |
| Tgfb3 | S.i2vl7 | transforming growth factor, beta 3 |
| Tgfb3 | S.i4vl7 | transforming growth factor, beta 3 |
| Tgfb3 | S.i1vl7 | transforming growth factor, beta 3 |
| Tgfbi | S.c2 | transforming growth factor, beta induced |
| Tgfbr3 | S.c2 | transforming growth factor, beta receptor III |
| Tgif1 | S.i2vl7 | TGFB-induced factor homeobox 1 |
| Tgif1 | S.i3vl7 | TGFB-induced factor homeobox 1 |
| Tgif1 | S.c4 | TGFB-induced factor homeobox 1 |
| Tgif1 | S.i4vl7 | TGFB-induced factor homeobox 1 |
| Thbd | S.i4vl7 | thrombomodulin |
| Thbd | S.c1 | thrombomodulin |
| Thbs1 | S.c4 | thrombospondin 1 |
| Thbs2 | S.i4vl7 | thrombospondin 2 |
| Thbs2 | S.c1 | thrombospondin 2 |
| Thra | S.c1 | thyroid hormone receptor alpha |
| Thrap3 | S.i2vl7 | thyroid hormone receptor associated protein 3 |
| Thrap3 | S.i4vl7 | thyroid hormone receptor associated protein 3 |
| Thrsp | S.c7 | thyroid hormone responsive |
| Thrsp | S.i1vl7 | thyroid hormone responsive |
| Thumpd3 | S.c4 | THUMP domain containing 3 |
| Thy1 | S.c3 | thymus cell antigen 1, theta |
| Thy1 | S.i2vl7 | thymus cell antigen 1, theta |
| Thy1 | S.i3vl7 | thymus cell antigen 1, theta |
| Thy1 | S.i4vl7 | thymus cell antigen 1, theta |
| Tie1 | S.c2 | tyrosine kinase with immunoglobulin-like and EGF-like domains 1 |
| Tie1 | S.c1 | tyrosine kinase with immunoglobulin-like and EGF-like domains 1 |
| Tie1 | S.i4vl7 | tyrosine kinase with immunoglobulin-like and EGF-like domains 1 |
| Tie1 | S.i3vl7 | tyrosine kinase with immunoglobulin-like and EGF-like domains 1 |
| Timm10b | S.i1vl7 | translocase of inner mitochondrial membrane 10B |
| Timm17b | S.c7 | translocase of inner mitochondrial membrane 17b |
| Timm17b | S.i4vl7 | translocase of inner mitochondrial membrane 17b |
| Timm17b | S.i1vl7 | translocase of inner mitochondrial membrane 17b |
| Timm17b | S.i3vl7 | translocase of inner mitochondrial membrane 17b |
| Timp2 | S.c2 | tissue inhibitor of metalloproteinase 2 |
| Timp3 | S.i4vl7 | tissue inhibitor of metalloproteinase 3 |
| Timp3 | S.c1 | tissue inhibitor of metalloproteinase 3 |
| Tinagl1 | S.i4vl7 | tubulointerstitial nephritis antigen-like 1 |
| Tinagl1 | S.c7 | tubulointerstitial nephritis antigen-like 1 |
| Tinagl1 | S.i3vl7 | tubulointerstitial nephritis antigen-like 1 |
| Tinagl1 | S.i2vl7 | tubulointerstitial nephritis antigen-like 1 |
| Tiprl | S.i2vl7 | TIP41, TOR signalling pathway regulator-like (S. cerevisiae) |
| Tiprl | S.c4 | TIP41, TOR signalling pathway regulator-like (S. cerevisiae) |
| Tiprl | S.i3vl7 | TIP41, TOR signalling pathway regulator-like (S. cerevisiae) |
| Tiprl | S.i4vl7 | TIP41, TOR signalling pathway regulator-like (S. cerevisiae) |
| Tjp1 | S.c4 | tight junction protein 1 |
| Tjp1 | S.i2vl7 | tight junction protein 1 |
| Tjp1 | S.i4vl7 | tight junction protein 1 |
| Tjp1 | S.i3vl7 | tight junction protein 1 |
| Tln1 | S.c9 | talin 1 |
| Tln1 | S.i2vl7 | talin 1 |
| Tln1 | S.i3vl7 | talin 1 |
| Tln1 | S.i4vl7 | talin 1 |
| Tm2d3 | S.i4vl7 | TM2 domain containing 3 |
| Tm2d3 | S.i2vl7 | TM2 domain containing 3 |
| Tm2d3 | S.c7 | TM2 domain containing 3 |
| Tm2d3 | S.i3vl7 | TM2 domain containing 3 |
| Tm2d3 | S.i1vl7 | TM2 domain containing 3 |
| Tm7sf2 | S.i3vl7 | transmembrane 7 superfamily member 2 |
| Tm7sf2 | S.i1vl7 | transmembrane 7 superfamily member 2 |
| Tm7sf2 | S.c7 | transmembrane 7 superfamily member 2 |
| Tm7sf2 | S.i2vl7 | transmembrane 7 superfamily member 2 |
| Tm7sf2 | S.i4vl7 | transmembrane 7 superfamily member 2 |
| Tm7sf3 | S.c4 | transmembrane 7 superfamily member 3 |
| Tmc6 | S.c7 | transmembrane channel-like gene family 6 |
| Tmco1 | S.i4vl7 | transmembrane and coiled-coil domains 1 |
| Tmco1 | S.i2vl7 | transmembrane and coiled-coil domains 1 |
| Tmed3 | S.i4vl7 | transmembrane emp24 domain containing 3 |
| Tmed3 | S.c7 | transmembrane emp24 domain containing 3 |
| Tmed3 | S.i1vl7 | transmembrane emp24 domain containing 3 |
| Tmem106c | S.c4 | transmembrane protein 106C |
| Tmem119 | S.i3vl7 | transmembrane protein 119 |
| Tmem119 | S.i4vl7 | transmembrane protein 119 |
| Tmem119 | S.i2vl7 | transmembrane protein 119 |
| Tmem119 | S.i1vl7 | transmembrane protein 119 |
| Tmem119 | c5.i3vl7 | transmembrane protein 119 |
| Tmem131 | S.i3vl7 | transmembrane protein 131 |
| Tmem131 | S.c4 | transmembrane protein 131 |
| Tmem160 | S.c7 | transmembrane protein 160 |
| Tmem160 | S.i1vl7 | transmembrane protein 160 |
| Tmem165 | S.c7 | transmembrane protein 165 |
| Tmem165 | S.i2vl7 | transmembrane protein 165 |
| Tmem165 | S.i1vl7 | transmembrane protein 165 |
| Tmem165 | S.i4vl7 | transmembrane protein 165 |
| Tmem165 | S.i3vl7 | transmembrane protein 165 |
| Tmem214 | S.i4vl7 | transmembrane protein 214 |
| Tmem214 | S.c8 | transmembrane protein 214 |
| Tmem230 | S.c4 | transmembrane protein 230 |
| Tmem230 | S.i4vl7 | transmembrane protein 230 |
| Tmem30b | S.c9 | transmembrane protein 30B |
| Tmem43 | S.c4 | transmembrane protein 43 |
| Tmem43 | S.i1vl7 | transmembrane protein 43 |
| Tmem45a | S.c1 | transmembrane protein 45a |
| Tmem45a | S.i4vl7 | transmembrane protein 45a |
| Tmem56 | S.i1vl7 | transmembrane protein 56 |
| Tmem56 | S.c6 | transmembrane protein 56 |
| Tmem66 | S.i3vl7 | transmembrane protein 66 |
| Tmem66 | S.c3 | transmembrane protein 66 |
| Tmem66 | S.i2vl7 | transmembrane protein 66 |
| Tmem97 | S.i3vl7 | transmembrane protein 97 |
| Tmem97 | S.i4vl7 | transmembrane protein 97 |
| Tmem97 | S.c7 | transmembrane protein 97 |
| Tmsb10 | S.i3vl7 | thymosin, beta 10 |
| Tmsb10 | S.i1vl7 | thymosin, beta 10 |
| Tmsb10 | S.i2vl7 | thymosin, beta 10 |
| Tmsb10 | S.i4vl7 | thymosin, beta 10 |
| Tmsb10 | S.c4 | thymosin, beta 10 |
| Tmsb4x | S.c4 | thymosin, beta 4, X chromosome |
| Tmsb4x | S.i3vl7 | thymosin, beta 4, X chromosome |
| Tmsb4x | S.i2vl7 | thymosin, beta 4, X chromosome |
| Tmsb4x | S.i1vl7 | thymosin, beta 4, X chromosome |
| Tmsb4x | S.i4vl7 | thymosin, beta 4, X chromosome |
| Tnfrsf1a | S.i3vl7 | tumor necrosis factor receptor superfamily, member 1a |
| Tnfrsf1a | S.c3 | tumor necrosis factor receptor superfamily, member 1a |
| Tnfrsf1a | S.i2vl7 | tumor necrosis factor receptor superfamily, member 1a |
| Tnfrsf1b | S.c1 | tumor necrosis factor receptor superfamily, member 1b |
| Tnip1 | S.c2 | TNFAIP3 interacting protein 1 |
| Tnip1 | S.i4vl7 | TNFAIP3 interacting protein 1 |
| Tnk2 | S.c7 | tyrosine kinase, non-receptor, 2 |
| Tnnc2 | S.c1 | troponin C2, fast |
| Tnp1 | S.i4vl7 | transition protein 1 |
| Tnp1 | S.c8 | transition protein 1 |
| Tnpo1 | S.c4 | transportin 1 |
| Tnxb | S.i3vl7 | tenascin XB |
| Tnxb | S.c2 | tenascin XB |
| Tnxb | S.i4vl7 | tenascin XB |
| Tob1 | S.i3vl7 | transducer of ErbB-2.1 |
| Tob1 | S.c7 | transducer of ErbB-2.1 |
| Tob1 | S.i4vl7 | transducer of ErbB-2.1 |
| Tom1l1 | S.c6 | target of myb1-like 1 (chicken) |
| Top3b | S.i4vl7 | topoisomerase (DNA) III beta |
| Tor1b | S.c9 | torsin family 1, member B |
| Tor1b | S.i4vl7 | torsin family 1, member B |
| Tor3a | S.c7 | torsin family 3, member A |
| Tpd52 | S.i4vl7 | tumor protein D52 |
| Tpd52 | S.i2vl7 | tumor protein D52 |
| Tpd52 | S.i3vl7 | tumor protein D52 |
| Tpd52 | S.c7 | tumor protein D52 |
| Tpd52 | S.i1vl7 | tumor protein D52 |
| Tpd52l1 | S.i4vl7 | tumor protein D52-like 1 |
| Tpd52l1 | S.c8 | tumor protein D52-like 1 |
| Tpd52l1 | S.i2vl7 | tumor protein D52-like 1 |
| Tpm1 | S.i4vl7 | tropomyosin 1, alpha |
| Tpm1 | S.i3vl7 | tropomyosin 1, alpha |
| Tpm1 | S.i1vl7 | tropomyosin 1, alpha |
| Tpm1 | S.c4 | tropomyosin 1, alpha |
| Tpm4 | S.i3vl7 | tropomyosin 4 |
| Tpm4 | S.i2vl7 | tropomyosin 4 |
| Tpm4 | S.i1vl7 | tropomyosin 4 |
| Tpm4 | S.c4 | tropomyosin 4 |
| Tpm4 | S.i4vl7 | tropomyosin 4 |
| Tpp1 | S.c3 | tripeptidyl peptidase I |
| Tpp2 | S.c2 | tripeptidyl peptidase II |
| Tprgl | S.c4 | transformation related protein 63 regulated like |
| Tprgl | S.i3vl7 | transformation related protein 63 regulated like |
| Tpst1 | S.c1 | protein-tyrosine sulfotransferase 1 |
| Trafd1 | S.i4vl7 | TRAF type zinc finger domain containing 1 |
| Trafd1 | S.c3 | TRAF type zinc finger domain containing 1 |
| Tram1 | S.i1vl7 | translocating chain-associating membrane protein 1 |
| Tram1 | S.i4vl7 | translocating chain-associating membrane protein 1 |
| Tram1 | S.c7 | translocating chain-associating membrane protein 1 |
| Tram1 | S.i3vl7 | translocating chain-associating membrane protein 1 |
| Trappc12 | S.i4vl7 | trafficking protein particle complex 12 |
| Trappc12 | S.c4 | trafficking protein particle complex 12 |
| Trappc12 | S.i1vl7 | trafficking protein particle complex 12 |
| Trib3 | S.c8 | tribbles homolog 3 (Drosophila) |
| Trim11 | S.i3vl7 | tripartite motif-containing 11 |
| Trim11 | S.i1vl7 | tripartite motif-containing 11 |
| Trim11 | S.i2vl7 | tripartite motif-containing 11 |
| Trim11 | c5.i3vl7 | tripartite motif-containing 11 |
| Trim25 | S.c6 | tripartite motif-containing 25 |
| Trim27 | S.c8 | tripartite motif-containing 27 |
| Trnt1 | S.c4 | tRNA nucleotidyl transferase, CCA-adding, 1 |
| Trp53 | S.c2 | transformation related protein 53 |
| Trp53 | S.i3vl7 | transformation related protein 53 |
| Tsc2 | S.c2 | tuberous sclerosis 2 |
| Tsc22d1 | S.i1vl7 | TSC22 domain family, member 1 |
| Tsc22d1 | S.i4vl7 | TSC22 domain family, member 1 |
| Tsc22d1 | S.i3vl7 | TSC22 domain family, member 1 |
| Tsc22d1 | c5.i3vl7 | TSC22 domain family, member 1 |
| Tsc22d1 | S.i2vl7 | TSC22 domain family, member 1 |
| Tsnax | S.i3vl7 | translin-associated factor X |
| Tsnax | S.i2vl7 | translin-associated factor X |
| Tsnax | S.c3 | translin-associated factor X |
| Tspan12 | S.i2vl7 | tetraspanin 12 |
| Tspan12 | S.c1 | tetraspanin 12 |
| Tspan33 | S.i3vl7 | tetraspanin 33 |
| Tspan33 | S.i2vl7 | tetraspanin 33 |
| Tspan33 | c5.i3vl7 | tetraspanin 33 |
| Tspan33 | S.i1vl7 | tetraspanin 33 |
| Tspan4 | S.c2 | tetraspanin 4 |
| Tspan4 | S.i2vl7 | tetraspanin 4 |
| Tspan4 | S.i4vl7 | tetraspanin 4 |
| Tspan7 | S.i3vl7 | tetraspanin 7 |
| Tspan7 | S.i1vl7 | tetraspanin 7 |
| Tspan7 | S.c8 | tetraspanin 7 |
| Tspan7 | S.i4vl7 | tetraspanin 7 |
| Tspan7 | S.i2vl7 | tetraspanin 7 |
| Tspo | S.i3vl7 | translocator protein |
| Tspo | S.i4vl7 | translocator protein |
| Tspo | S.c2 | translocator protein |
| Tspo | S.i2vl7 | translocator protein |
| Tst | S.c1 | thiosulfate sulfurtransferase, mitochondrial |
| Tsta3 | S.i2vl7 | tissue specific transplantation antigen P35B |
| Ttc3 | S.i4vl7 | tetratricopeptide repeat domain 3 |
| Ttc3 | S.i3vl7 | tetratricopeptide repeat domain 3 |
| Ttc3 | S.c4 | tetratricopeptide repeat domain 3 |
| Ttc3 | S.i2vl7 | tetratricopeptide repeat domain 3 |
| Ttc3 | S.i1vl7 | tetratricopeptide repeat domain 3 |
| Ttc33 | S.c4 | tetratricopeptide repeat domain 33 |
| Ttc7b | S.c9 | tetratricopeptide repeat domain 7B |
| Tuba1a | S.i2vl7 | tubulin, alpha 1A |
| Tuba1a | S.i4vl7 | tubulin, alpha 1A |
| Tuba1a | S.c3 | tubulin, alpha 1A |
| Tuba1a | S.i3vl7 | tubulin, alpha 1A |
| Tubb2a | S.c4 | tubulin, beta 2A class IIA |
| Tubb5 | S.i3vl7 | tubulin, beta 5 class I |
| Tubb5 | S.i4vl7 | tubulin, beta 5 class I |
| Tubb5 | S.c4 | tubulin, beta 5 class I |
| Tubb5 | S.c3 | tubulin, beta 5 class I |
| Tubb5 | S.i1vl7 | tubulin, beta 5 class I |
| Tubb5 | S.i2vl7 | tubulin, beta 5 class I |
| Tubgcp4 | S.i4vl7 | tubulin, gamma complex associated protein 4 |
| Tufm | S.c8 | Tu translation elongation factor, mitochondrial |
| Tufm | S.i4vl7 | Tu translation elongation factor, mitochondrial |
| Tufm | S.i3vl7 | Tu translation elongation factor, mitochondrial |
| Tufm | S.i2vl7 | Tu translation elongation factor, mitochondrial |
| Twist1 | S.c1 | twist basic helix-loop-helix transcription factor 1 |
| Twsg1 | S.i4vl7 | twisted gastrulation homolog 1 (Drosophila) |
| Twsg1 | S.i2vl7 | twisted gastrulation homolog 1 (Drosophila) |
| Twsg1 | S.i1vl7 | twisted gastrulation homolog 1 (Drosophila) |
| Twsg1 | S.i3vl7 | twisted gastrulation homolog 1 (Drosophila) |
| Txndc12 | S.i1vl7 | thioredoxin domain containing 12 (endoplasmic reticulum) |
| Txndc12 | S.c6 | thioredoxin domain containing 12 (endoplasmic reticulum) |
| Txndc9 | S.c6 | thioredoxin domain containing 9 |
| Txndc9 | S.i1vl7 | thioredoxin domain containing 9 |
| Txnip | c5.i3vl7 | thioredoxin interacting protein |
| Txnip | S.i1vl7 | thioredoxin interacting protein |
| Txnip | S.i4vl7 | thioredoxin interacting protein |
| Txnip | S.i2vl7 | thioredoxin interacting protein |
| Txnip | S.i3vl7 | thioredoxin interacting protein |
| Tyro3 | S.i1vl7 | TYRO3 protein tyrosine kinase 3 |
| Tyro3 | S.c6 | TYRO3 protein tyrosine kinase 3 |
| Tyro3 | S.i3vl7 | TYRO3 protein tyrosine kinase 3 |
| Tyro3 | S.i2vl7 | TYRO3 protein tyrosine kinase 3 |
| Tyrobp | S.i4vl7 | TYRO protein tyrosine kinase binding protein |
| Tyrobp | S.i2vl7 | TYRO protein tyrosine kinase binding protein |
| Tyrobp | S.c2 | TYRO protein tyrosine kinase binding protein |
| Tyrobp | S.i3vl7 | TYRO protein tyrosine kinase binding protein |
| Uba2 | S.i4vl7 | ubiquitin-like modifier activating enzyme 2 |
| Uba5 | S.i4vl7 | ubiquitin-like modifier activating enzyme 5 |
| Uba5 | S.c7 | ubiquitin-like modifier activating enzyme 5 |
| Uba5 | S.i1vl7 | ubiquitin-like modifier activating enzyme 5 |
| Uba5 | S.i2vl7 | ubiquitin-like modifier activating enzyme 5 |
| Uba5 | S.i3vl7 | ubiquitin-like modifier activating enzyme 5 |
| Uba7 | S.c4 | ubiquitin-like modifier activating enzyme 7 |
| Uba7 | S.i2vl7 | ubiquitin-like modifier activating enzyme 7 |
| Uba7 | S.i4vl7 | ubiquitin-like modifier activating enzyme 7 |
| Uba7 | S.i1vl7 | ubiquitin-like modifier activating enzyme 7 |
| Uba7 | S.i3vl7 | ubiquitin-like modifier activating enzyme 7 |
| Ubald2 | S.c6 | UBA-like domain containing 2 |
| Ubc | S.c2 | ubiquitin C |
| Ube2b | S.i2vl7 | ubiquitin-conjugating enzyme E2B |
| Ube2b | S.i4vl7 | ubiquitin-conjugating enzyme E2B |
| Ube2b | S.c4 | ubiquitin-conjugating enzyme E2B |
| Ube2d2a | S.i1vl7 | ubiquitin-conjugating enzyme E2D 2A |
| Ube2n | S.c4 | ubiquitin-conjugating enzyme E2N |
| Ube2r2 | S.c4 | ubiquitin-conjugating enzyme E2R 2 |
| Ube2r2 | S.i1vl7 | ubiquitin-conjugating enzyme E2R 2 |
| Ubr4 | S.i1vl7 | ubiquitin protein ligase E3 component n-recognin 4 |
| Ubr4 | S.i3vl7 | ubiquitin protein ligase E3 component n-recognin 4 |
| Ubr4 | S.i4vl7 | ubiquitin protein ligase E3 component n-recognin 4 |
| Ubr4 | S.i2vl7 | ubiquitin protein ligase E3 component n-recognin 4 |
| Ubr4 | c5.i3vl7 | ubiquitin protein ligase E3 component n-recognin 4 |
| Ubtf | S.c2 | upstream binding transcription factor, RNA polymerase I |
| Ubtf | S.i4vl7 | upstream binding transcription factor, RNA polymerase I |
| Ubtf | S.i3vl7 | upstream binding transcription factor, RNA polymerase I |
| Ubtf | S.i2vl7 | upstream binding transcription factor, RNA polymerase I |
| Ubxn1 | S.i2vl7 | UBX domain protein 1 |
| Ubxn4 | c5.i3vl7 | UBX domain protein 4 |
| Ubxn4 | S.i3vl7 | UBX domain protein 4 |
| Ubxn4 | S.i2vl7 | UBX domain protein 4 |
| Ubxn8 | S.i1vl7 | UBX domain protein 8 |
| Ubxn8 | S.c6 | UBX domain protein 8 |
| Uck1 | S.c1 | uridine-cytidine kinase 1 |
| Uck2 | S.c9 | uridine-cytidine kinase 2 |
| Uck2 | S.i4vl7 | uridine-cytidine kinase 2 |
| Ucp1 | S.c1 | uncoupling protein 1 (mitochondrial, proton carrier) |
| Ucp3 | S.c6 | uncoupling protein 3 (mitochondrial, proton carrier) |
| Ufm1 | S.c9 | ubiquitin-fold modifier 1 |
| Ufm1 | S.i3vl7 | ubiquitin-fold modifier 1 |
| Ufm1 | S.i4vl7 | ubiquitin-fold modifier 1 |
| Ugdh | S.i1vl7 | UDP-glucose dehydrogenase |
| Ugdh | S.i3vl7 | UDP-glucose dehydrogenase |
| Ugdh | S.i4vl7 | UDP-glucose dehydrogenase |
| Ugdh | S.c4 | UDP-glucose dehydrogenase |
| Ugt1a1 | S.i2vl7 | UDP glucuronosyltransferase 1 family, polypeptide A1 |
| Ugt1a1 | S.c1 | UDP glucuronosyltransferase 1 family, polypeptide A1 |
| Ugt1a1 | S.i4vl7 | UDP glucuronosyltransferase 1 family, polypeptide A1 |
| Ugt1a10 | S.c1 | UDP glycosyltransferase 1 family, polypeptide A10 |
| Ugt1a10 | S.i2vl7 | UDP glycosyltransferase 1 family, polypeptide A10 |
| Ugt1a10 | S.i4vl7 | UDP glycosyltransferase 1 family, polypeptide A10 |
| Ugt1a2 | S.i4vl7 | UDP glucuronosyltransferase 1 family, polypeptide A2 |
| Ugt1a2 | S.i2vl7 | UDP glucuronosyltransferase 1 family, polypeptide A2 |
| Ugt1a2 | S.c1 | UDP glucuronosyltransferase 1 family, polypeptide A2 |
| Ugt1a5 | S.i4vl7 | UDP glucuronosyltransferase 1 family, polypeptide A5 |
| Ugt1a5 | S.i2vl7 | UDP glucuronosyltransferase 1 family, polypeptide A5 |
| Ugt1a5 | S.c1 | UDP glucuronosyltransferase 1 family, polypeptide A5 |
| Ugt1a6a | S.i4vl7 | UDP glucuronosyltransferase 1 family, polypeptide A6A |
| Ugt1a6a | S.c1 | UDP glucuronosyltransferase 1 family, polypeptide A6A |
| Ugt1a6a | S.i2vl7 | UDP glucuronosyltransferase 1 family, polypeptide A6A |
| Ugt1a6b | S.i4vl7 | UDP glucuronosyltransferase 1 family, polypeptide A6B |
| Ugt1a6b | S.c1 | UDP glucuronosyltransferase 1 family, polypeptide A6B |
| Ugt1a6b | S.i2vl7 | UDP glucuronosyltransferase 1 family, polypeptide A6B |
| Ugt1a7c | S.i4vl7 | UDP glucuronosyltransferase 1 family, polypeptide A7C |
| Ugt1a7c | S.i2vl7 | UDP glucuronosyltransferase 1 family, polypeptide A7C |
| Ugt1a7c | S.c1 | UDP glucuronosyltransferase 1 family, polypeptide A7C |
| Ugt1a9 | S.i2vl7 | UDP glucuronosyltransferase 1 family, polypeptide A9 |
| Ugt1a9 | S.c1 | UDP glucuronosyltransferase 1 family, polypeptide A9 |
| Ugt1a9 | S.i4vl7 | UDP glucuronosyltransferase 1 family, polypeptide A9 |
| Uhmk1 | S.i4vl7 | U2AF homology motif (UHM) kinase 1 |
| Uhmk1 | S.i3vl7 | U2AF homology motif (UHM) kinase 1 |
| Uhmk1 | S.i2vl7 | U2AF homology motif (UHM) kinase 1 |
| Uhmk1 | S.c4 | U2AF homology motif (UHM) kinase 1 |
| Ulk1 | S.c6 | unc-51 like kinase 1 |
| Unc119 | S.i4vl7 | unc-119 homolog (C. elegans) |
| Unc119 | S.c3 | unc-119 homolog (C. elegans) |
| Unc119 | S.i3vl7 | unc-119 homolog (C. elegans) |
| Uqcr11 | S.i1vl7 | ubiquinol-cytochrome c reductase, complex III subunit XI |
| Uqcrq | S.i3vl7 | ubiquinol-cytochrome c reductase, complex III subunit VII |
| Uqcrq | S.i2vl7 | ubiquinol-cytochrome c reductase, complex III subunit VII |
| Uqcrq | S.c7 | ubiquinol-cytochrome c reductase, complex III subunit VII |
| Urod | S.i3vl7 | uroporphyrinogen decarboxylase |
| Urod | S.c8 | uroporphyrinogen decarboxylase |
| Use1 | S.c3 | unconventional SNARE in the ER 1 homolog (S. cerevisiae) |
| Use1 | S.i4vl7 | unconventional SNARE in the ER 1 homolog (S. cerevisiae) |
| Use1 | S.i3vl7 | unconventional SNARE in the ER 1 homolog (S. cerevisiae) |
| Usp14 | S.c6 | ubiquitin specific peptidase 14 |
| Usp18 | S.c6 | ubiquitin specific peptidase 18 |
| Usp2 | S.i2vl7 | ubiquitin specific peptidase 2 |
| Usp2 | S.c8 | ubiquitin specific peptidase 2 |
| Usp2 | S.i4vl7 | ubiquitin specific peptidase 2 |
| Usp24 | S.c9 | ubiquitin specific peptidase 24 |
| Usp24 | S.i4vl7 | ubiquitin specific peptidase 24 |
| Usp25 | S.i2vl7 | ubiquitin specific peptidase 25 |
| Usp25 | S.i1vl7 | ubiquitin specific peptidase 25 |
| Usp25 | S.c7 | ubiquitin specific peptidase 25 |
| Usp25 | S.i3vl7 | ubiquitin specific peptidase 25 |
| Usp3 | S.c6 | ubiquitin specific peptidase 3 |
| Usp48 | S.i1vl7 | ubiquitin specific peptidase 48 |
| Usp48 | S.c3 | ubiquitin specific peptidase 48 |
| Usp48 | S.i2vl7 | ubiquitin specific peptidase 48 |
| Usp48 | S.i3vl7 | ubiquitin specific peptidase 48 |
| Vamp8 | S.c6 | vesicle-associated membrane protein 8 |
| Vars | S.c7 | valyl-tRNA synthetase |
| Vav1 | S.c8 | vav 1 oncogene |
| Vav1 | S.i2vl7 | vav 1 oncogene |
| Vav1 | S.i4vl7 | vav 1 oncogene |
| Vcam1 | S.c1 | vascular cell adhesion molecule 1 |
| Vcam1 | S.i4vl7 | vascular cell adhesion molecule 1 |
| Vcl | S.c4 | vinculin |
| Vcl | S.i4vl7 | vinculin |
| Vcl | S.i2vl7 | vinculin |
| Vcl | S.i3vl7 | vinculin |
| Vcl | S.i1vl7 | vinculin |
| Vcp | S.c8 | valosin containing protein |
| Vdr | S.c7 | vitamin D receptor |
| Vdr | S.c9 | vitamin D receptor |
| Vegfb | S.i4vl7 | vascular endothelial growth factor B |
| Vegfb | S.i3vl7 | vascular endothelial growth factor B |
| Vegfb | S.c2 | vascular endothelial growth factor B |
| Vkorc1 | S.c2 | vitamin K epoxide reductase complex, subunit 1 |
| Vopp1 | S.c9 | vesicular, overexpressed in cancer, prosurvival protein 1 |
| Vps37b | S.c8 | vacuolar protein sorting 37B (yeast) |
| Vps4b | S.c4 | vacuolar protein sorting 4b (yeast) |
| Vps72 | S.c2 | vacuolar protein sorting 72 (yeast) |
| Vrk3 | S.c4 | vaccinia related kinase 3 |
| Vwf | S.i4vl7 | Von Willebrand factor homolog |
| Vwf | S.c1 | Von Willebrand factor homolog |
| Wbp1 | S.i4vl7 | WW domain binding protein 1 |
| Wbp1l | S.c3 | WW domain binding protein 1 like |
| Wbp1l | S.i4vl7 | WW domain binding protein 1 like |
| Wbp1l | S.i3vl7 | WW domain binding protein 1 like |
| Wbp5 | S.c6 | WW domain binding protein 5 |
| Wdr20 | S.c8 | WD repeat domain 20 |
| Wdr20 | S.i4vl7 | WD repeat domain 20 |
| Wdr26 | c5.i3vl7 | WD repeat domain 26 |
| Wdr26 | S.i3vl7 | WD repeat domain 26 |
| Wdr45 | S.c4 | WD repeat domain 45 |
| Wdr81 | S.i1vl7 | WD repeat domain 81 |
| Wdr81 | S.c4 | WD repeat domain 81 |
| Wdr81 | S.i2vl7 | WD repeat domain 81 |
| Wdr81 | S.i4vl7 | WD repeat domain 81 |
| Wdr92 | S.i1vl7 | WD repeat domain 92 |
| Wdr92 | S.i4vl7 | WD repeat domain 92 |
| Wdr92 | S.c8 | WD repeat domain 92 |
| Wdr92 | S.i3vl7 | WD repeat domain 92 |
| Wfdc12 | S.i3vl7 | WAP four-disulfide core domain 12 |
| Wfdc12 | S.i1vl7 | WAP four-disulfide core domain 12 |
| Wfdc12 | S.i2vl7 | WAP four-disulfide core domain 12 |
| Wfdc12 | S.c6 | WAP four-disulfide core domain 12 |
| Wfdc2 | S.i2vl7 | WAP four-disulfide core domain 2 |
| Wipf1 | S.c6 | WAS/WASL interacting protein family, member 1 |
| Wls | S.i3vl7 | wntless homolog (Drosophila) |
| Wls | S.i4vl7 | wntless homolog (Drosophila) |
| Wls | S.c7 | wntless homolog (Drosophila) |
| Wnk2 | S.c2 | WNK lysine deficient protein kinase 2 |
| Wnt5b | S.i4vl7 | wingless-related MMTV integration site 5B |
| Wnt5b | S.i3vl7 | wingless-related MMTV integration site 5B |
| Wnt5b | c5.i3vl7 | wingless-related MMTV integration site 5B |
| Wnt5b | S.i1vl7 | wingless-related MMTV integration site 5B |
| Wnt5b | S.i2vl7 | wingless-related MMTV integration site 5B |
| Wtap | S.i4vl7 | Wilms tumour 1-associating protein |
| Wwtr1 | S.c6 | WW domain containing transcription regulator 1 |
| Xdh | S.i4vl7 | xanthine dehydrogenase |
| Xdh | S.c9 | xanthine dehydrogenase |
| Xpa | S.c4 | xeroderma pigmentosum, complementation group A |
| Xpc | S.i1vl7 | xeroderma pigmentosum, complementation group C |
| Xpc | S.c4 | xeroderma pigmentosum, complementation group C |
| Xpot | S.c7 | exportin, tRNA (nuclear export receptor for tRNAs) |
| Xrcc1 | S.i4vl7 | X-ray repair complementing defective repair in Chinese hamster cells 1 |
| Xrcc5 | S.c9 | X-ray repair complementing defective repair in Chinese hamster cells 5 |
| Yipf5 | S.i3vl7 | Yip1 domain family, member 5 |
| Yipf5 | S.c7 | Yip1 domain family, member 5 |
| Ypel3 | S.c3 | yippee-like 3 (Drosophila) |
| Ywhag | S.c3 | tyrosine 3-monooxygenase/tryptophan 5-monooxygenase activation protein, gamma polypeptide |
| Ywhah | S.i1vl7 | tyrosine 3-monooxygenase/tryptophan 5-monooxygenase activation protein, eta polypeptide |
| Ywhah | S.i2vl7 | tyrosine 3-monooxygenase/tryptophan 5-monooxygenase activation protein, eta polypeptide |
| Ywhah | S.c4 | tyrosine 3-monooxygenase/tryptophan 5-monooxygenase activation protein, eta polypeptide |
| Ywhah | S.i3vl7 | tyrosine 3-monooxygenase/tryptophan 5-monooxygenase activation protein, eta polypeptide |
| Ywhah | S.i4vl7 | tyrosine 3-monooxygenase/tryptophan 5-monooxygenase activation protein, eta polypeptide |
| Ywhaq | S.c4 | tyrosine 3-monooxygenase/tryptophan 5-monooxygenase activation protein, theta polypeptide |
| Ywhaq | S.i4vl7 | tyrosine 3-monooxygenase/tryptophan 5-monooxygenase activation protein, theta polypeptide |
| Ywhaq | S.i3vl7 | tyrosine 3-monooxygenase/tryptophan 5-monooxygenase activation protein, theta polypeptide |
| Ywhaq | S.i1vl7 | tyrosine 3-monooxygenase/tryptophan 5-monooxygenase activation protein, theta polypeptide |
| Ywhaq | S.i2vl7 | tyrosine 3-monooxygenase/tryptophan 5-monooxygenase activation protein, theta polypeptide |
| Zbed6 | S.i4vl7 | zinc finger, BED domain containing 6 |
| Zbed6 | S.c3 | zinc finger, BED domain containing 6 |
| Zbtb16 | S.c2 | zinc finger and BTB domain containing 16 |
| Zbtb22 | S.i4vl7 | zinc finger and BTB domain containing 22 |
| Zbtb22 | S.c8 | zinc finger and BTB domain containing 22 |
| Zdhhc3 | S.i2vl7 | zinc finger, DHHC domain containing 3 |
| Zdhhc3 | c5.i3vl7 | zinc finger, DHHC domain containing 3 |
| Zdhhc3 | S.i3vl7 | zinc finger, DHHC domain containing 3 |
| Zdhhc9 | S.c9 | zinc finger, DHHC domain containing 9 |
| Zeb1 | S.i4vl7 | zinc finger E-box binding homeobox 1 |
| Zeb1 | S.i2vl7 | zinc finger E-box binding homeobox 1 |
| Zeb1 | S.i3vl7 | zinc finger E-box binding homeobox 1 |
| Zeb1 | S.c8 | zinc finger E-box binding homeobox 1 |
| Zfand2a | S.i3vl7 | zinc finger, AN1-type domain 2A |
| Zfand2a | S.c6 | zinc finger, AN1-type domain 2A |
| Zfand5 | S.i3vl7 | zinc finger, AN1-type domain 5 |
| Zfand5 | c5.i3vl7 | zinc finger, AN1-type domain 5 |
| Zfand5 | S.i4vl7 | zinc finger, AN1-type domain 5 |
| Zfp207 | S.c4 | zinc finger protein 207 |
| Zfp207 | S.i4vl7 | zinc finger protein 207 |
| Zfp281 | S.i4vl7 | zinc finger protein 281 |
| Zfp281 | S.i3vl7 | zinc finger protein 281 |
| Zfp281 | S.c3 | zinc finger protein 281 |
| Zfp36 | S.i2vl7 | zinc finger protein 36 |
| Zfp36 | S.c7 | zinc finger protein 36 |
| Zfp36 | S.i4vl7 | zinc finger protein 36 |
| Zfp36l1 | S.i4vl7 | zinc finger protein 36, C3H type-like 1 |
| Zfp36l1 | S.c3 | zinc finger protein 36, C3H type-like 1 |
| Zfp644 | S.i2vl7 | zinc finger protein 644 |
| Zfp644 | S.c4 | zinc finger protein 644 |
| Zfp644 | S.i4vl7 | zinc finger protein 644 |
| Zfp644 | S.i3vl7 | zinc finger protein 644 |
| Zfp703 | S.c2 | zinc finger protein 703 |
| Zfp703 | S.i4vl7 | zinc finger protein 703 |
| Zfp771 | S.i2vl7 | zinc finger protein 771 |
| Zfp771 | S.i1vl7 | zinc finger protein 771 |
| Zfp771 | S.i4vl7 | zinc finger protein 771 |
| Zfpm1 | S.c7 | zinc finger protein, multitype 1 |
| Zfr | S.c3 | zinc finger RNA binding protein |
| Zfr | S.i4vl7 | zinc finger RNA binding protein |
| Zhx1 | S.c7 | zinc fingers and homeoboxes 1 |
| Zic2 | S.i2vl7 | zinc finger protein of the cerebellum 2 |
| Zic2 | S.c8 | zinc finger protein of the cerebellum 2 |
| Zic2 | S.i3vl7 | zinc finger protein of the cerebellum 2 |
| Zic2 | S.i4vl7 | zinc finger protein of the cerebellum 2 |
| Zmiz2 | S.i4vl7 | zinc finger, MIZ-type containing 2 |
| Zmiz2 | S.c3 | zinc finger, MIZ-type containing 2 |
| Zmiz2 | S.i3vl7 | zinc finger, MIZ-type containing 2 |
| Zmiz2 | S.i1vl7 | zinc finger, MIZ-type containing 2 |
| Zmynd11 | S.c4 | zinc finger, MYND domain containing 11 |
| Znrf2 | S.i4vl7 | zinc and ring finger 2 |
| Znrf2 | S.c9 | zinc and ring finger 2 |
| Zranb2 | S.i3vl7 | zinc finger, RAN-binding domain containing 2 |
| Zranb2 | S.i2vl7 | zinc finger, RAN-binding domain containing 2 |
| Zranb2 | S.c3 | zinc finger, RAN-binding domain containing 2 |
| Zranb2 | S.i4vl7 | zinc finger, RAN-binding domain containing 2 |
| Zscan21 | S.i2vl7 | zinc finger and SCAN domain containing 21 |
| Zscan21 | S.i4vl7 | zinc finger and SCAN domain containing 21 |
| Zscan21 | S.c8 | zinc finger and SCAN domain containing 21 |
| Zwint | S.i2vl7 | ZW10 interactor |
| Zwint | S.i3vl7 | ZW10 interactor |
| Zwint | S.i4vl7 | ZW10 interactor |
| Zwint | S.c4 | ZW10 interactor |
| Zwint | S.i1vl7 | ZW10 interactor |

S1: List of orthologous genes identified in Clarkson et al. [3] and Stein et al. [4].
