# Supplementary material for: Gene set analysis of post-lactational mammary gland involution gene signatures in inflammatory and triple-negative breast cancer
Source: PLoS One. 2018 Apr 4;13(4):e0192689. doi: 10.1371/journal.pone.0192689 (PMC5884491; doi:10.1371/journal.pone.0192689)
Supplement: S2 Table — BP, biological process; CC; cellular components. (DOCX) [file pone.0192689.s002.docx]

| Type | Category ID | Category Name | # Genes Category | STEM Clusters | | | | | | | | | |
| --- | --- | --- | --- | --- | --- | --- | --- | --- | --- | --- | --- | --- | --- |
|  |  |  |  | #1 | #2 | #3 | #4 | #5 | #6 | #7 | #8 | #9 | #10 |
| BP | GO:0001568 | Blood vessel development | 394 | 0.002 | NS | NS | NS | NS | NS | NS | NS | NS | NS |
| BP | GO:0001944 | Vasculature development | 415 | 0.004 | NS | NS | NS | NS | NS | NS | NS | NS | NS |
| BP | GO:0002455 | Humoral immune response mediated by circulating immunoglobulin | 37 | 0.038 | NS | NS | NS | NS | NS | NS | NS | NS | NS |
| BP | GO:0006082 | Organic acid metabolic process | 517 | NS | NS | NS | NS | NS | NS | 0.054 | NS | NS | NS |
| BP | GO:0006631 | Fatty acid metabolic process | 190 | 0.038 | NS | NS | NS | NS | 0.048 | NS | NS | NS | NS |
| BP | GO:0006635 | Fatty acid beta-oxidation | 37 | 0.038 | NS | NS | NS | NS | NS | NS | NS | NS | NS |
| BP | GO:0006909 | Phagocytosis | 84 | 0.008 | NS | NS | NS | NS | NS | NS | NS | NS | NS |
| BP | GO:0006950 | Response to stress | 1539 | 0.004 | NS | NS | NS | NS | NS | NS | NS | NS | NS |
| BP | GO:0006952 | Defense response | 575 | <0.001 | NS | NS | NS | NS | NS | NS | NS | NS | NS |
| BP | GO:0006954 | Inflammatory response | 322 | 0.002 | NS | NS | NS | NS | NS | NS | NS | NS | NS |
| BP | GO:0006955 | Immune response | 529 | <0.001 | NS | NS | NS | NS | NS | NS | NS | NS | NS |
| BP | GO:0009062 | Fatty acid catabolic process | 47 | 0.036 | NS | NS | NS | NS | NS | NS | NS | NS | NS |
| BP | GO:0009605 | Response to external stimulus | 932 | 0.004 | NS | NS | NS | NS | NS | NS | NS | NS | NS |
| BP | GO:0016042 | Lipid catabolic process | 130 | 0.018 | NS | NS | NS | NS | NS | NS | NS | NS | NS |
| BP | GO:0019395 | Fatty acid oxidation | 54 | 0.026 | NS | NS | NS | NS | NS | NS | NS | NS | NS |
| BP | GO:0019752 | Carboxylic acid metabolic process | 490 | NS | NS | NS | NS | NS | NS | 0.036 | NS | NS | NS |
| BP | GO:0019882 | Antigen processing and presentation | 60 | 0.008 | NS | NS | NS | NS | NS | NS | NS | NS | NS |
| BP | GO:0030036 | Actin cytoskeleton organization | 279 | 0.038 | NS | NS | NS | NS | NS | NS | NS | NS | NS |
| BP | GO:0030198 | Extracellular matrix organization | 129 | 0.004 | NS | NS | NS | NS | NS | NS | NS | NS | NS |
| BP | GO:0032101 | Regulation of response to external stimulus | 344 | 0.014 | NS | NS | NS | NS | NS | NS | NS | NS | NS |
| BP | GO:0032103 | Positive regulation of response to external stimulus | 144 | 0.046 | NS | NS | NS | NS | NS | NS | NS | NS | NS |
| BP | GO:0032787 | Monocarboxylic acid metabolic process | 286 | NS | NS | NS | NS | NS | 0.05 | NS | NS | NS | NS |
| BP | GO:0034097 | Response to cytokine | 298 | 0.004 | NS | NS | NS | NS | NS | NS | NS | NS | NS |
| BP | GO:0034341 | Response to interferon-gamma | 39 | 0.008 | NS | NS | NS | NS | NS | NS | NS | NS | NS |
| BP | GO:0034440 | Lipid oxidation | 56 | 0.004 | NS | NS | NS | NS | NS | NS | NS | NS | NS |
| BP | GO:0040012 | Regulation of locomotion | 435 | 0.048 | NS | NS | NS | NS | NS | NS | NS | NS | NS |
| BP | GO:0042127 | Regulation of cell proliferation | 869 | 0.052 | NS | NS | NS | NS | NS | NS | NS | NS | NS |
| BP | GO:0042221 | Response to chemical | 1573 | NS | NS | NS | NS | NS | NS | NS | NS | 0.004 | NS |
| BP | GO:0043062 | Extracellular structure organization | 129 | 0.004 | NS | NS | NS | NS | NS | NS | NS | NS | NS |
| BP | GO:0043436 | Oxoacid metabolic process | 511 | NS | NS | NS | NS | NS | NS | 0.05 | NS | NS | NS |
| BP | GO:0044281 | Small molecule metabolic process | 1299 | NS | NS | NS | NS | NS | 0.006 | 0.012 | NS | NS | NS |
| BP | GO:0044711 | Single-organism biosynthetic process | 700 | NS | NS | NS | NS | NS | 0.02 | NS | NS | NS | NS |
| BP | GO:0045087 | Innate immune response | 233 | 0.002 | NS | NS | NS | NS | NS | NS | NS | NS | NS |
| BP | GO:0048514 | Blood vessel morphogenesis | 344 | 0.032 | NS | NS | NS | NS | NS | NS | NS | NS | NS |
| BP | GO:0070887 | Cellular response to chemical stimulus | 1033 | NS | NS | NS | NS | NS | NS | NS | NS | 0.024 | NS |
| BP | GO:1901605 | Alpha-amino acid metabolic process | 117 | NS | NS | 0.052 | NS | NS | NS | NS | NS | NS | NS |
| BP | GO:2000145 | Regulation of cell motility | 396 | 0.048 | NS | NS | NS | NS | NS | NS | NS | NS | NS |
| CC | GO:0000502 | Proteasome complex | 53 | NS | <0.001 | NS | NS | NS | NS | NS | NS | NS | NS |
| CC | GO:0000932 | Cytoplasmic mrna processing body | 26 | NS | 0.02 | NS | NS | NS | NS | NS | NS | NS | NS |
| CC | GO:0005578 | Proteinaceous extracellular matrix | 183 | <0.001 | NS | NS | NS | NS | NS | NS | NS | NS | NS |
| CC | GO:0005581 | Collagen trimer | 51 | 0.004 | NS | NS | NS | NS | NS | NS | NS | NS | NS |
| CC | GO:0005615 | Extracellular space | 694 | <0.001 | NS | NS | NS | NS | NS | NS | NS | NS | NS |
| CC | GO:0005783 | Endoplasmic reticulum | 747 | NS | NS | NS | NS | NS | 0.002 | NS | NS | NS | NS |
| CC | GO:0005838 | Proteasome regulatory particle | 11 | NS | <0.001 | NS | NS | NS | NS | NS | NS | NS | NS |
| CC | GO:0009986 | Cell surface | 473 | 0.026 | NS | NS | NS | NS | NS | NS | NS | NS | NS |
| CC | GO:0012505 | Endomembrane system | 1633 | NS | NS | NS | NS | NS | 0.006 | NS | NS | NS | NS |
| CC | GO:0016021 | Integral component of membrane | 1967 | NS | NS | NS | NS | NS | 0.008 | NS | NS | NS | NS |
| CC | GO:0022624 | Proteasome accessory complex | 18 | NS | <0.001 | NS | NS | NS | NS | NS | NS | NS | NS |
| CC | GO:0031224 | Intrinsic component of membrane | 2045 | NS | NS | NS | NS | NS | 0.006 | NS | NS | NS | NS |
| CC | GO:0031982 | Vesicle | 1944 | <0.001 | <0.001 | NS | NS | NS | NS | NS | NS | NS | NS |
| CC | GO:0031988 | Membrane-bounded vesicle | 1828 | <0.001 | <0.001 | NS | NS | NS | NS | NS | NS | NS | 0.052 |
| CC | GO:0043227 | Membrane-bounded organelle | 5489 | NS | NS | NS | NS | NS | NS | NS | 0.038 | NS | NS |
| CC | GO:0043230 | Extracellular organelle | 1534 | <0.001 | <0.001 | NS | NS | NS | NS | NS | NS | NS | 0.04 |
| CC | GO:0044420 | Extracellular matrix part | 89 | 0.004 | NS | NS | NS | NS | NS | NS | NS | NS | NS |
| CC | GO:0044421 | Extracellular region part | 1992 | <0.001 | <0.001 | NS | NS | NS | NS | NS | NS | NS | NS |
| CC | GO:0044444 | Cytoplasmic part | 3297 | NS | NS | NS | NS | NS | 0.048 | NS | NS | NS | NS |
| CC | GO:0065010 | Extracellular membrane-bounded organelle | 1534 | <0.001 | <0.001 | NS | NS | NS | NS | NS | NS | NS | 0.04 |
| CC | GO:0070062 | Extracellular vesicular exosome | 1528 | <0.001 | <0.001 | NS | NS | NS | NS | NS | NS | NS | 0.038 |

S2: Results of ontology analysis for the STEM significant clusters. BP, biological process; CC; cellular components.
