# Supplementary material for: Gene set analysis of post-lactational mammary gland involution gene signatures in inflammatory and triple-negative breast cancer
Source: PLoS One. 2018 Apr 4;13(4):e0192689. doi: 10.1371/journal.pone.0192689 (PMC5884491; doi:10.1371/journal.pone.0192689)
Supplement: S3 Table — (DOCX) [file pone.0192689.s003.docx]

|  | **Involution signature** | **# Genes total** | **# Genes overlapped with IBC-like signature** | **Symbols of Genes overlapped with IBC-like signature** |
| --- | --- | --- | --- | --- |
| Clarkson et al. 2004 | Inv1 | 256 | 1 | MARCKS |
|  | Inv2 | 118 | 2 | ARPC2, HSP90B1 |
|  | Inv3 | 69 | 2 | FOLR1, TMC6 |
|  | Inv4 | 47 | 0 |  |
|  | Inv5 | 43 | 0 |  |
|  | Inv6 | 76 | 1 | DAB2 |
|  | Inv7 | 39 | 0 |  |
|  | Inv8 | 31 | 0 |  |
|  | Inv9 | 37 | 0 |  |
|  | Inv10 | 26 | 0 |  |
| Stein et al. 2009 | S.c1 | 242 | 1 | PRKCB |
|  | S.c2 | 262 | 1 | HSP90B1 |
|  | S.c3 | 325 | 4 | MARCKS, PNP, SP3, ZFR |
|  | S.c4 | 343 | 7 | ACTG1, ARPC2, ATF2, IQGAP1, RYK, TBL1XR1, TNPO1 |
|  | S.c5.i3vl7 | 146 | 4 | ACOX1, CTBP2, CTSA, IQGAP1 |
|  | S.c6 | 137 | 1 | PAK2 |
|  | S.c7 | 282 | 2 | DAB2, TMC6 |
|  | S.c8 | 212 | 3 | BCKDK, DAB2, FOLR1 |
|  | S.c9 | 81 | 1 | JMJD6 |
|  | S.i1vl7 | 643 | 7 | ACOX1, ARPC2, CTBP2, CTSA, FOLR1, IQGAP1, RYK |
|  | S.i2vl7 | 790 | 10 | ACOX1, ARPC2, CTBP2, CTSA, DAB2, FOLR1, IQGAP1, PNP, RYK, TMCO1 |
|  | S.i3vl7 | 834 | 9 | ACOX1, ARPC2, CTBP2, CTSA, DAB2, FOLR1, HSP90B1, IQGAP1, RYK |
|  | S.i4vl7 | 1160 | 14 | ACOX1, ARPC2, ATF2, BCKDK, CTBP2, CTSA, DAB2, FOLR1, IQGAP1, MARCKS, PNP, RYK, TMCO1, ZFR |

S3: Overlap of genes between involution-specific signatures and IBC-like signature.
