# Supplementary material for: Gene set analysis of post-lactational mammary gland involution gene signatures in inflammatory and triple-negative breast cancer
Source: PLoS One. 2018 Apr 4;13(4):e0192689. doi: 10.1371/journal.pone.0192689 (PMC5884491; doi:10.1371/journal.pone.0192689)
Supplement: S4 Table — (DOCX) [file pone.0192689.s004.docx]

| Gene Signature | Enriched in IBC vs Normal | Size | ES | NES | Nominal p-value | FDR q-value |
| --- | --- | --- | --- | --- | --- | --- |
| Involution specific signatures developed through STEM clustering using data from Clarkson et al. 2004 | | | | | | |
| Inv1 | IBC | 205 | 0.46 | 1.29 | 0.127 | 0.202 |
| Inv2 | Normal | 100 | -0.24 | -0.85 | 0.635 | 0.864 |
| Inv3 | Normal | 57 | -0.57 | -1.36 | 0.016 | 0.251 |
| Inv4 | IBC | 33 | 0.43 | 1.1 | 0.327 | 0.281 |
| Inv5 | Normal | 30 | -0.48 | -1.3 | 0.123 | 0.249 |
| Inv6 | Normal | 62 | -0.3 | -0.94 | 0.571 | 0.851 |
| Inv7 | IBC | 36 | 0.46 | 1.15 | 0.111 | 0.315 |
| Inv8 | Normal | 24 | -0.29 | -0.71 | 0.923 | 0.898 |
| Inv9 | IBC | 33 | 0.67 | 1.34 | 0.055 | 0.27 |
| Inv10 | Normal | 21 | -0.43 | -1.08 | 0.412 | 0.715 |
| Involution specific signatures reported in Stein et al. 2009 | | | | | | |
| S.C1 | IBC | 182 | 0.41 | 1.02 | 0.481 | 0.389 |
| S.C2 | IBC | 205 | 0.54 | 1.26 | 0.08 | 0.56 |
| S.C3 | IBC | 252 | 0.39 | 1.25 | 0.114 | 0.31 |
| S.C4 | Normal | 258 | -0.23 | -0.77 | 0.833 | 0.859 |
| S.C5.I3VL7 | Normal | 117 | -0.36 | -1.15 | 0.2 | 0.44 |
| S.C6 | Normal | 100 | -0.41 | -1.13 | 0.339 | 0.362 |
| S.C7 | IBC | 225 | 0.33 | 1.11 | 0.244 | 0.35 |
| S.C8 | Normal | 153 | -0.41 | -1.28 | 0.091 | 0.493 |
| S.C9 | Normal | 66 | -0.55 | -1.24 | 0.24 | 0.397 |
| S.I1VL7 | Normal | 495 | -0.29 | -1.06 | 0.31 | 0.403 |
| S.I2VL7 | IBC | 612 | 0.31 | 1.14 | 0.186 | 0.367 |
| S.I3VL7 | IBC | 648 | 0.28 | 1.03 | 0.3 | 0.417 |
| S.I4VL7 | IBC | 894 | 0.34 | 1.19 | 0.128 | 0.357 |

S4: Signature analysis of the breast parenchyma from 19 IBC patients and 25 non-IBC patients.
